# Nontrigonal constraint enhances 1,2-addition reactivity of phosphazenes†
†Electronic supplementary information (ESI) available. CCDC 1821116–1821122. For ESI and crystallographic data in CIF or other electronic format see DOI: 10.1039/c8sc00929e


**DOI:** 10.1039/c8sc00929e

**Published:** 2018-04-06

**Authors:** Yi-Chun Lin, James C. Gilhula, Alexander T. Radosevich

**Affiliations:** a Department of Chemistry , Massachusetts Institute of Technology , Cambridge , MA 02139 , USA . Email: radosevich@mit.edu

## Abstract

The syntheses and 1,2-addition reactivities of nontrigonal phosphazenes supported by trianionic tricoordinating chelates of the type L_3_P=Ndipp (**3**: L_3_ = N[CHC(^*t*^Bu)O]_2_^3–^; **4**: L_3_ = N(*o*-NMeC_6_H_4_)_2_^3–^; dipp = 2,6-diisopropylphenyl) are reported. These compounds are characterized by multinuclear NMR and single-crystal X-ray diffraction experiments. Distorted phosphazenes **3** and **4** are shown to add B–H, B–O, and Si–H bonds across the formal P=N double bond, and their reactivities are contrasted with acyclic analogues. Derivatives of phosphazene **3** bearing sterically unencumbered *N*-substitutents readily dimerize to form the corresponding cyclodiphosphazanes; compounds with sterically demanding *N*-substituents are interconvertible between their monomeric and dimeric forms. The enhanced electrophilicity of the phosphorus center in nontrigonal phosphazenes **3** and **4** is rationalized by DFT calculations. Gas phase fluoride ion affinities are computed to be markedly higher for distorted phosphazenes, while proton affinities are largely unaffected by geometric distortion. These results are interpreted to suggest that distortion from pseudotetrahedral geometry results in stabilization of the P-based LUMO, while HOMO energies are essentially unchanged.

## Abstract 2

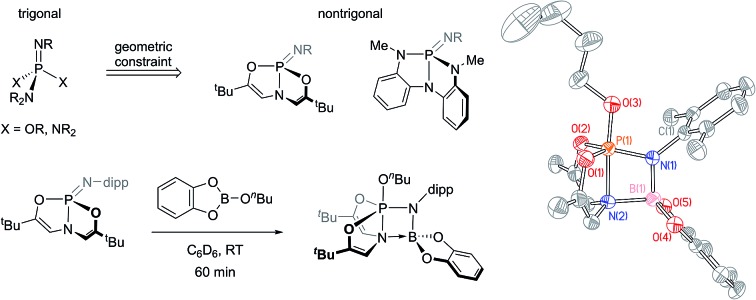
The syntheses and 1,2-addition reactivities of nontrigonal phosphazenes supported by trianionic tricoordinating chelates of the type L_3_P=Ndipp (**3**: L_3_ = N[CHC(^*t*^Bu)O]_2_^3–^; **4**: L_3_ = N(*o*-NMeC_6_H_4_)_2_^3–^; dipp = 2,6-diisopropylphenyl) are reported.

## 1. Introduction

Phosphazenes, σ^4^,λ^5^-phosphorus compounds of the form R_3_P=NR′, comprise a broad class of functionally diverse main group inorganic molecules.^1^ The properties and reactivities of phosphazenes can be modulated over a wide range depending on substitution at the two heteroatom positions. In many circumstances, the P=N unit proves quite inert; indeed, the robustness of the P=N moiety forms the basis for the many remarkable applications of polyphosphazene inorganic/organic hybrid materials.^2^

As might be expected on the basis of the differing electronegativities of phosphorus and nitrogen, however, the polarization of the formal P=N double bond (*i.e.*, contributing Lewis structures R_3_P=NR′ ↔ R_3_P^+^–N^–^R′) also gives rise to numerous applications for phosphazenes as nitrogen-based electron pair donors. For instance, phosphazenes are known to be strong donor ligands for transition metals.^3^ Phosphazenes have found synthetic use as strong, non-ionic organic superbases; the tetrameric triaminophosphazene superbase *t*-Bu-P_4_ displays exceptionally high Brønsted–Lowry basicity and has been investigated for a variety of base-mediated transformations.^4^

Apart from this nitrogen-based reactivity, phosphazenes also have been employed in a number of transformations that leverage the vicinal ambiphilic character of the P=N unit. Chief among this class of reactions are metathetical transformations stemming from formal (2 + 2) addition/elimination of unsaturated organic compounds at the phosphazene P=N moiety, of which the aza-Wittig^5^ and related imine metathesis^6^ reaction are representative. An increasingly important use of phosphazenes comes in the Staudinger ligation reaction, which has become an important tool in bioconjugation chemistry.^7^

**Fig. 1:**
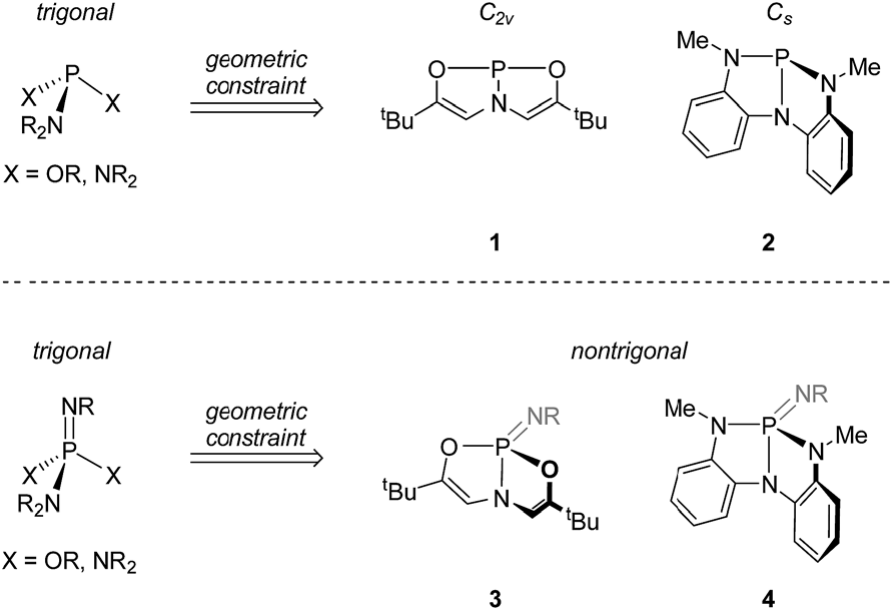
(top) Trianionic *O*,*N*,*O*- and *N*,*N*,*N*-chelating ligands enforce nontrigonal geometries in P(iii) compounds. (bottom) These ligand frameworks may be further extended to form nontrigonal phosphazenes.

Recently, we have been investigating the connection between equilibrium ground-state structure and reactivity in a class of nontrigonal phosphabicyclic compounds. We have documented that the distorted molecular geometries for these tricoordinate phosphorus compounds have a significant impact on both their electronic structure and reactivity. For instance, we have reported that Arduengo's T-shaped phosphorus compound^8^**1** (Fig. 1 top) supports transfer hydrogenation catalysis^9^ and N–H oxidative addition reactivity.^10^

In addition, we reported the synthesis of *C*_s_-symmetric phosphorus compound **2** (Fig. 1, top) and its ability to undergo E–H bond activation reactivity (E = –OR, –NHR, –BR_2_) at phosphorus through a ligand-cooperative mechanism.^11^,^12^

Based on this precedent, we wished to ascertain the extent to which the distinctive reactivity traits of nontrigonal phosphorus compounds **1** and **2** might range beyond the tricoordinate state to support interesting chemical properties of their distorted σ^4^,λ^5^-phosphazene derivatives. In this study, we report a combined theoretical and experimental treatment of phosphazenes based on supporting structures **1** and **2** which validate the hypothesis that imposition of a geometric constraint at positions ancillary to the P=N unit enhances vicinal ditopic ambiphilicity of these phosphazenes. We also show that 1,2-reactivity of the P=N unit leads to facile addition of σ-bonded B–H, B–O, and Si–H reagents across the P=N moiety. In total, the results establish a rational framework for the design of bespoke phosphazenes with novel properties and reactivities that expand the functional role of this important class of main group compounds.

## 2. Results and discussion

### 2.1. Computational model of the phosphazene distortion coordinate

**Fig. 2:**
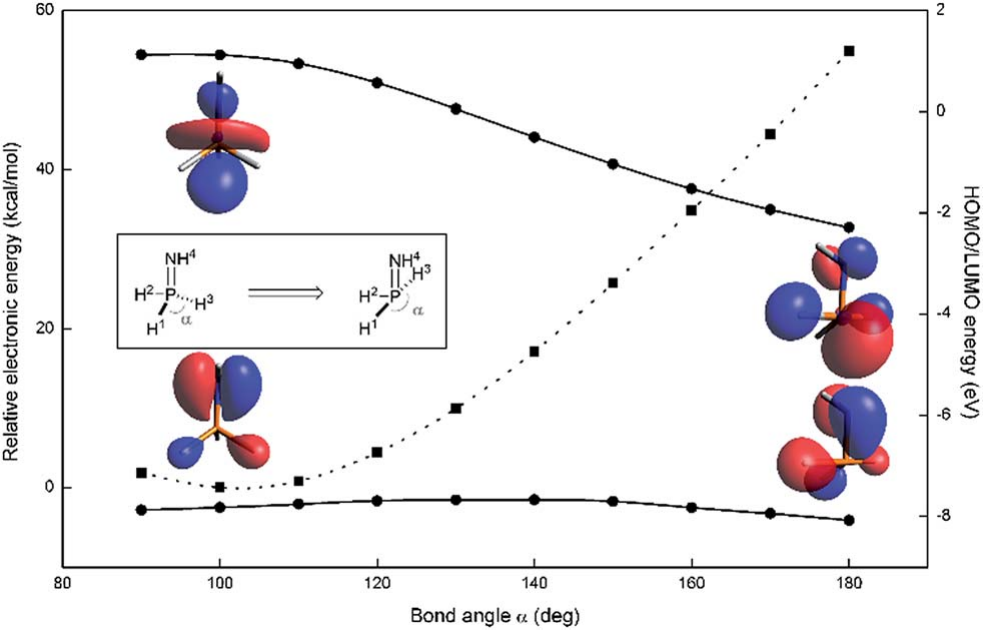
Relative electronic energy (dashed, square) and HOMO/LUMO energies (solid, circle) in H_3_P=NH as a function of bond angle, calculated at the M06-2X/def2-TZVP level of theory. HOMO and LUMO Kohn–Sham orbitals are shown for *α* = 100° and 180°.

A computational appraisal of the consequence of molecular distortion on phosphazene frontier electronic structure illustrates the theoretical framework underlying our experimental study. With the parent phosphazene H_3_P=NH (Fig. 2, inset) as a minimalistic model system, orbital and overall electronic energies were computed as a function of internal bond angle ∠H_1_–P–H_3_ (*α*) in the range 90° < *α* < 180° at the M06-2X/def2-TZVP level of theory^13^,^14^ as implemented in the ORCA 4.0.0 software package.^15^,^16^ For each structure scanned, the dihedral angle ∠H_2_–P–N–H_4_ was relaxed to minimize energy associated with rotation about the P=N formal double bond; bond lengths were held constant at values obtained from the equilibrium geometry of H_3_P=NH (*d*_P–H_ = 1.44 Å, *d*_P–N_ = 1.61 Å, *d*_N–H_ = 1.02 Å); these parameters are in good agreement with those found in previous computational studies of H_3_P=NH.^17^

Deviations from the equilibrium geometry (*α* = 97.9°) predictably lead to increases in overall energy (Fig. 2, dotted line). Notwithstanding this fact, the energy of the highest occupied orbital (*ε*_HOMO_) remains more or less constant across the scanned coordinate. Visual inspection of the relevant orbitals provides for a qualitative interpretation of this observation; highest occupied molecular orbitals are found to be mostly nitrogen-based, corresponding largely to the N lone pair (*n*_N_) with minor contributions from the ancillary phosphorus substituents.

By contrast, the energy of the lowest unoccupied orbital (*ε*_LUMO_) decreases as the bond angle *α* increases, ultimately resulting in stabilization of more than 3 eV as LUMO takes on increasing s-orbital character in the distortion to seesaw geometry (*α* = 180°). This electronic picture suggests that the nontrigonal distortion of phosphazenes should retain the Lewis basicity of the N position but dramatically increase the Lewis acidity of the P position. We posit that the juxtaposition of donor and acceptor character at adjacent atoms should lead to an increase in 1,2-ambiphilic reactivity of the P=N unit, in analogy to well-established chemistry of early transition metal imido (M=NR) complexes.^18^ We sought to confirm these theoretical predictions experimentally with a suite of phosphazene compounds.

### 2.2. Phosphazene synthesis and characterization

#### 2.2.1 Synthesis and spectroscopy

**Scheme 1:**
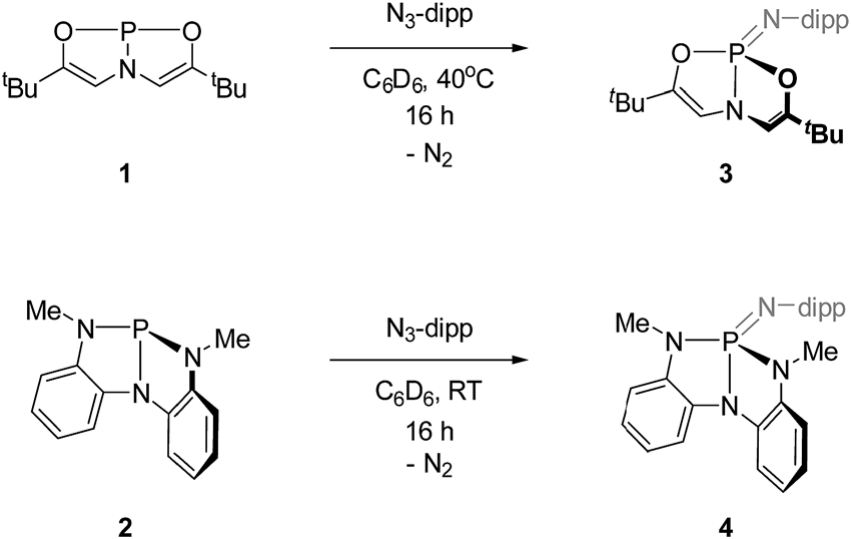
Synthesis of phosphazenes **3** and **4** (dipp = 2,6-diisopropylphenyl).

The requisite phosphazenes are easily prepared by Staudinger imination^19^ of tricoordinate phosphorus platforms. Reaction of *C*_2v_-symmetric phosphorus compound **1** with 2,6-diisopropylphenyl azide (C_6_D_6_, 40 °C, 16 h), followed by removal of volatiles and trituration of the residue in pentane, produced a white solid whose ^31^P{^1^H} NMR spectrum displayed a resonance at *δ* 7.3 ppm; the observed chemical shift is consistent with compositionally similar *O*,*N*,*O*-substituted phosphazenes previously reported in the literature.^20^ In the ^1^H NMR spectrum, both vinylic protons (*δ* 5.49 ppm) and ^*t*^Bu groups (*δ* 0.91 ppm) on the *O*,*N*,*O*-support scaffold of **3** give rise to a single resonance, respectively, suggesting a time-averaged molecular geometry of *C*_s_ molecular symmetry or higher. In contrast to the planar starting material **1**, the ^3^*J*_P–H_ scalar coupling constant between phosphorus and the vinylic hydrogen nuclei for **3** (^3^*J*_P–H_ = 29 Hz) is discernible and modest in magnitude. Arduengo previously noted^21^ an empirical relationship between the magnitude of this ^3^*J*_P–H_ value and the extent of molecular folding of the *O*,*N*,*O*-supporting ligand in related σ^4^-P compounds. On this basis, we infer that the bicyclic *O*,*N*,*O* ligand backbone is distorted from planarity by folding along the P–N axis. We therefore assigned the structure of **3** as in Scheme 1.

Similarly, compound **4** was prepared by the reaction of **2** with 1 equiv. of 2,6-diisopropylphenyl azide (C_6_D_6_, RT, 12 h) and subsequent recrystallization from a solution of pentane and dichloromethane. The ^31^P{^1^H} NMR spectrum of this compound showed a resonance at *δ* 14.5 ppm, consistent with typical values for tetraazaphosphazenes. The spectroscopic equivalence of both ligand *N*-methyl substituents in the ^1^H NMR spectrum suggest time-averaged *C*_s_ or higher symmetry, as with compound **3**.

**Scheme 2:**
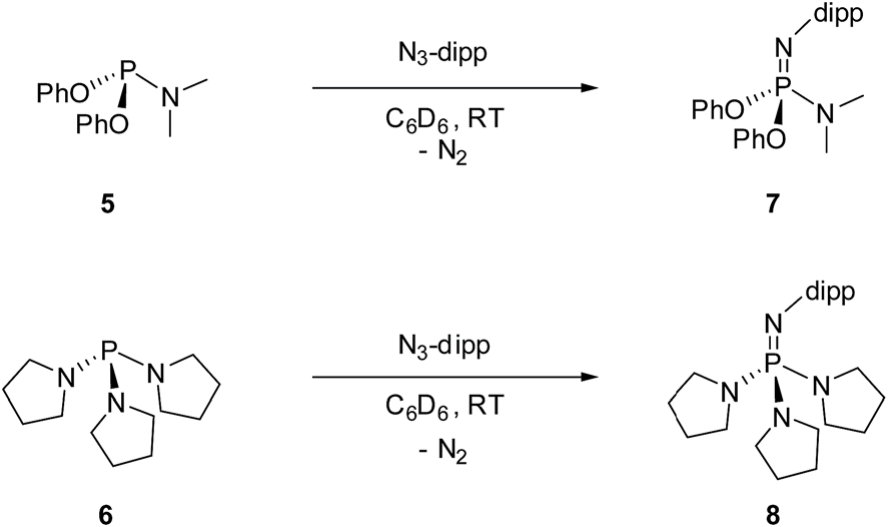
Synthesis of phosphazene **7** and **8** (dipp = 2,6-diisopropylphenyl).

For the purpose of comparison, acyclic phosphazene analogues **7** and **8** were likewise synthesized from the corresponding phosphorus compounds **5** and **6** (Scheme 2).

#### 2.2.2 Solid state structures

**Fig. 3:**
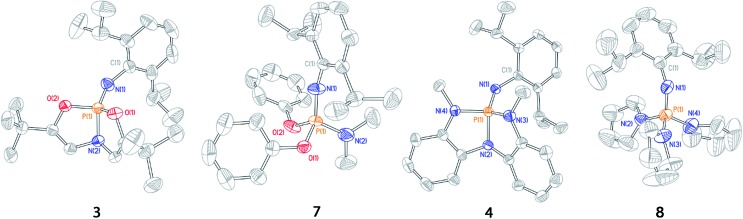
Thermal ellipsoid plots of phosphazenes **3**, **7**, **4**, and **8** rendered at 50% probability level. Hydrogen atoms omitted for clarity.

Crystalline solids of **3** and **4** suitable for X-ray diffraction were grown by vapor diffusion from a dichloromethane/pentane system, and their solid state structures were determined (Fig. 3, Table 1). As expected, both structures exhibit nontrigonal distortion enforced by the ligand. Acyclic *O*,*N*,*O*- (**7**) and *N*,*N*,*N*- (**8**)^22^ phosphazene analogues also were analyzed by X-ray diffraction of single crystalline samples obtained from dichloromethane/pentane solution.

**Table 1:** Selected bond distances (Å) and angles (°) for phosphazenes **3**, **4**, **7**, and **8**^*a*^

Metric	**3**	**7**	**4**	**8**
*d*(P_1_–N_1_)	1.5069(14)	1.4810(17)	1.5309(11)	1.525(3)
*d*(P_1_–N_2_)	1.6903(13)	1.613(2)	1.7095(10)	1.644(3)
*d*(P_1_–O_1_)	1.6044(12)	1.6044(15)		
*d*(P_1_–O_2_)	1.5962(11)	1.5985(15)		
*d*(P_1_–N_3_)			1.6723(12)	1.639(3)
*d*(P_1_–N_4_)			1.6591(11)	1.632(3)
∠**O**_**1**_**–P**_**1**_**–O**_**2**_	**112.62(6)**	**96.70(8)**		
∠O_1_–P_1_–N_1_	115.30(7)	120.04(9)		
∠O_1_–P_1_–N_2_	96.68(7)	101.79(9)		
∠O_2_–P_1_–N_1_	109.40(7)	112.35(9)		
∠O_2_–P_1_–N_2_	96.19(6)	107.06(11)		
∠**N**_**1**_**–P**_**1**_**–N**_**2**_	**124.96(7)**	**116.46(11)**	**129.22(6)**	**117.32(15)**
∠N_1_–P_1_–N_3_			114.72(6)	104.41(16)
∠N_1_–P_1_–N_4_			107.24(6)	108.23(14)
∠N_2_–P_1_–N_3_			92.35(5)	104.41(16)
∠N_2_–P_1_–N_4_			92.84(5)	102.58(15)
∠**N**_**3**_**–P**_**1**_**–N**_**4**_			**119.73(6)**	**108.45(16)**
∠P_1_–N_1_–C_1_	135.72(10)	153.82(15)	137.19(10)	137.3(2)

^*a*^See ESI for full crystallographic details. Bond angles most relevant to nontrigonal distortion in bold.

The solid state structure of **3** displays substantial opening of the O_1_–P_1_–O_2_ bond angle relative to the acyclic phosphazene **7**; the bond angle in **3** (112.62(6)°) is 16° wider than that of **7** (96.70(8)°). The increase in O_1_–P_1_–O_2_ bond angle in **3** is complemented by a moderate (*ca.* 5–10°) decrease in internal bond angles O_1_–P_1_–O_2_. Additionally, the N_1_–P_1_–N_2_ angle likewise increases in **3** (124.96(7)°) relative to **7** (116.46(11)°) in order to restrict unfavorable steric interactions between the *N*-dipp substituent and the ligand backbone. Thus, the solid state structure of phosphazene **3** corresponds to a deviation from idealized pseudo-tetrahedral geometry along the distortion coordinate illustrated in Fig. 2.

Similarly, *N*,*N*,*N*-phosphabicyclic phosphazene **4** has an expanded N_3_–P_1_–N_4_ bond angle of **4** (119.73(6)°), 14° greater than the average angle in **8** (105.15(15)°). Furthermore, the N_2_–P_1_–N_3_ and N_3_–P_1_–N_4_ endocyclic angles were generally smaller relative to **8**. As with constrained phosphazene **3**, there was also an expansion of the N_1_–P_1_–N_2_ bond angle in **4** (129.22(6)°) *versus***8** (109.99(15)°, average of 3 angles), constituting a difference of 19°; this difference is greater than that of the **3**/**7** pair and is likely a result of increased steric congestion imposed by the *N*-methylanilides of **4**.

These solid state structures confirm our hypothesis that imposing geometric constraints *via* a bicyclic ligand framework results in a nontrigonal geometry along the distortion coordinate toward see-saw molecular structures.

#### 2.2.3 Computational comparison of constrained and unconstrained compounds

We performed gas phase fluoride ion affinity (FIA) and proton affinity (PA) calculations on **3**, **4**, **7**, and **8** at the M06-2X/def2-TZVP level of theory as a way of quantifying their respective Lewis acidities and basicities (see ESI† for full details). FIAs were calculated *via* Christe's method,^23^ summarized in eqn (1), and PAs were similarly computed according to eqn (2). The collected values are reported in Table 2.

1 CF_2_O + [R_3_P(F)

<svg xmlns="http://www.w3.org/2000/svg" version="1.0" width="16.000000pt" height="16.000000pt" viewBox="0 0 16.000000 16.000000" preserveAspectRatio="xMidYMid meet"><metadata>
Created by potrace 1.16, written by Peter Selinger 2001-2019
</metadata><g transform="translate(1.000000,15.000000) scale(0.005147,-0.005147)" fill="currentColor" stroke="none"><path d="M0 1440 l0 -80 1360 0 1360 0 0 80 0 80 -1360 0 -1360 0 0 -80z M0 960 l0 -80 1360 0 1360 0 0 80 0 80 -1360 0 -1360 0 0 -80z"/></g></svg>

NR′]^–^ → CF_3_O^–^ + R_3_P

<svg xmlns="http://www.w3.org/2000/svg" version="1.0" width="16.000000pt" height="16.000000pt" viewBox="0 0 16.000000 16.000000" preserveAspectRatio="xMidYMid meet"><metadata>
Created by potrace 1.16, written by Peter Selinger 2001-2019
</metadata><g transform="translate(1.000000,15.000000) scale(0.005147,-0.005147)" fill="currentColor" stroke="none"><path d="M0 1440 l0 -80 1360 0 1360 0 0 80 0 80 -1360 0 -1360 0 0 -80z M0 960 l0 -80 1360 0 1360 0 0 80 0 80 -1360 0 -1360 0 0 -80z"/></g></svg>

NR′ Δ*H*_rxn_ = FIA

2 [R_3_P

<svg xmlns="http://www.w3.org/2000/svg" version="1.0" width="16.000000pt" height="16.000000pt" viewBox="0 0 16.000000 16.000000" preserveAspectRatio="xMidYMid meet"><metadata>
Created by potrace 1.16, written by Peter Selinger 2001-2019
</metadata><g transform="translate(1.000000,15.000000) scale(0.005147,-0.005147)" fill="currentColor" stroke="none"><path d="M0 1440 l0 -80 1360 0 1360 0 0 80 0 80 -1360 0 -1360 0 0 -80z M0 960 l0 -80 1360 0 1360 0 0 80 0 80 -1360 0 -1360 0 0 -80z"/></g></svg>

N(H)R′]^+^ → H^+^ + R_3_P

<svg xmlns="http://www.w3.org/2000/svg" version="1.0" width="16.000000pt" height="16.000000pt" viewBox="0 0 16.000000 16.000000" preserveAspectRatio="xMidYMid meet"><metadata>
Created by potrace 1.16, written by Peter Selinger 2001-2019
</metadata><g transform="translate(1.000000,15.000000) scale(0.005147,-0.005147)" fill="currentColor" stroke="none"><path d="M0 1440 l0 -80 1360 0 1360 0 0 80 0 80 -1360 0 -1360 0 0 -80z M0 960 l0 -80 1360 0 1360 0 0 80 0 80 -1360 0 -1360 0 0 -80z"/></g></svg>

NR′ Δ*H*_rxn_ = PA

**Table 2:** Gas phase fluoride ion affinities and proton affinities of phosphazenes **3**, **4**, **7**, and **8** calculated at the M06-2X/def2-TZVP level of theory

Compound	FIA^*a*^	PA^*a*^
**3**	70.4	238
**7**	47.6	239
**4**	57.3	246
**8**	20.3	258

^*a*^Values in kcal mol^–1^.

Fluoride ion affinities for constrained phosphazenes **3** and **4** are significantly greater (>20 kcal mol^–1^ difference in each case) than their unconstrained analogues **7** and **8**. This result conforms with qualitative predictions from the model system H_3_P=NH (*vide supra*, Section 2.1.); upon distortion from pseudo-tetrahedral geometry, the phosphorus-based LUMOs decrease in energy, allowing for stronger interactions of exogenous anions like F^–^ with nontrigonal **3** and **4** than with trigonal compounds **7** and **8**.

Differences in proton affinities, on the other hand, are much smaller in magnitude. For instance, the computed PAs for compounds **3** and **7** are 238 kcal mol^–1^ and 239 kcal mol^–1^, respectively. For compounds **4** and **8**, the PAs are 246 kcal mol^–1^ and 258 kcal mol^–1^, respectively. Given the magnitude of the calculated proton affinities, the difference in calculated PAs for **3**/**7** and **4**/**8** does not exceed 6% overall. This outcome is in accord with the interpretation from calculations in Section 2.1. in which molecular distortion would not be expected to affect significantly the N-based HOMOs responsible for Lewis basicity.

### 2.3. Monomer–dimer speciation of nontrigonal phosphazenes

#### 2.3.1 Effect of trianionic chelate on monomer–dimer equilibrium of phosphazenes

**Scheme 3:**
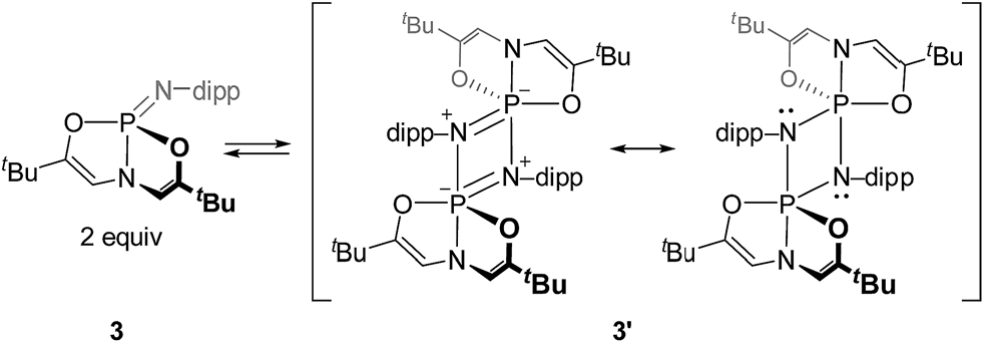
Interconversion of monomer and dimer forms of *O*,*N*,*O*-phosphazenes.

Although the monomeric phosphazene **3** could be prepared and characterized as indicated above, prolonged standing of solutions (*ca.* 3 d) at 60 °C led to the emergence of a new species with a low-field ^31^P NMR resonance (*δ* = –26.9 ppm). The formation and intensity of this new signal followed a concentration dependence in [**3**], suggesting a possible bimolecular origin. In accord with this observation, the identity of the new species was ultimately confirmed by single crystal X-ray diffraction analysis to be that of a head-to-tail homodimeric 1,3-diaza-2,4-diphosphetidine (cyclodiphosphazane) **3′**, which could be selectively crystallized from benzene solutions of **3**/**3′** (Scheme 3).

**Fig. 4:**
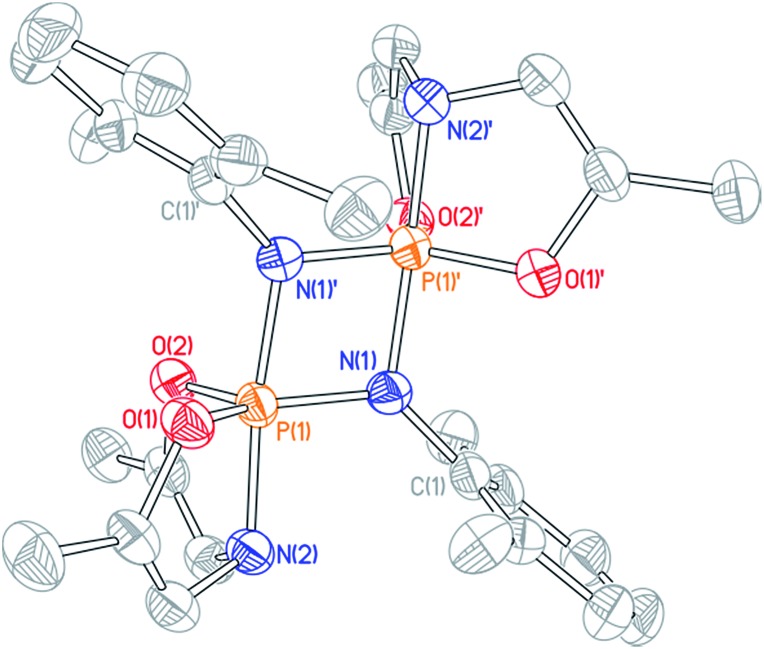
Molecular structure of **3′** with 50% probability level rendered thermal ellipsoid plot. One half of the molecule was present in the asymmetric unit; the other half was generated by the relevant symmetry operations. All hydrogen and methyl carbon atoms removed for clarity. Selected bond lengths [Å] and angles [°]: P(1)–N(1) 1.665(3), P(1)–N(1)′ 1.783(3), P(1)–N(2) 1.782(3), P(1)–O(1) 1.640(2), P(1)–O(2) 1.651(2); N(1)–P(1)–N(2) 95.17(13), N(1)′–P(1)–N(2) 172.04(13), O(1)–P(1)–O(2) 112.90(13).

Cyclophosphazane **3′** is found to crystallize in the centrosymmetric monoclinic space group *C*2/*c*, with one phosphazene monomer fragment in the asymmetric unit and the dimer generated by symmetry. The structural data indicate local trigonal bipyramidal geometry about each phosphorus center, where the formerly imino nitrogen atoms occupy one equatorial and one apical site (Fig. 4). The most distinctive feature of this stereochemical arrangement is the dissymmetric P–N bond distances within a planar diamond-like P_2_N_2_ core. As might be expected on the basis of the Rundle–Pimentel model of bonding in pentacoordinate p-block compounds,^24^ the axial P–N bond is significantly longer (*d*_P–N_ = 1.783(3) Å) than the equatorial P–N bonds (*d*_P–N_ = 1.665(3) Å). Both bond distances are elongated as compared to monomeric **3** (*d*_P–N_ = 1.5069(14) Å). In short, the structural metrics are consistent with a superposition of the Lewis structures for **3′**, displayed in Scheme 3.

Dissolution of a single-crystalline sample of **3′** in C_6_D_6_ at ambient temperature resulted in repopulation of the mixture containing both dimer **3′** and monomer **3** as judged by ^1^H and ^31^P NMR spectroscopy. Consequently, we conclude that the formal 2 + 2 dimerization of **3** to **3′** is reversible, as illustrated in Scheme 3. Notwithstanding the apparent dynamic nature of the monomer–dimer equilibrium, the appearance of two distinct resonances for **3** and **3′** in the ^31^P NMR spectra is most consistent with a slow interconversion relative to the NMR timescale.

In contrast to the deformed *O*,*N*,*O*-phosphazene **3**, dimerization of the constrained *N*,*N*,*N*-phosphazene **4** was not observed under any conditions.^25^ We posit that in this case the steric crowding by N–Me substituents of the bicyclic ligand framework and imino *N*-substituent prohibits close approach of a second phosphazene as would be necessary for dimer formation. Additionally, the phosphorus center of **4** is less Lewis acidic than that of **3** (as revealed by FIA analysis above), so it is possible that decreased electrophilicity at P precludes sufficient driving force for phosphazene dimerization.

#### 2.3.2 Effect of imino *N*-substituent on monomer–dimer equilibrium of *O*,*N*,*O* substituted phosphazenes

**Scheme 4:**
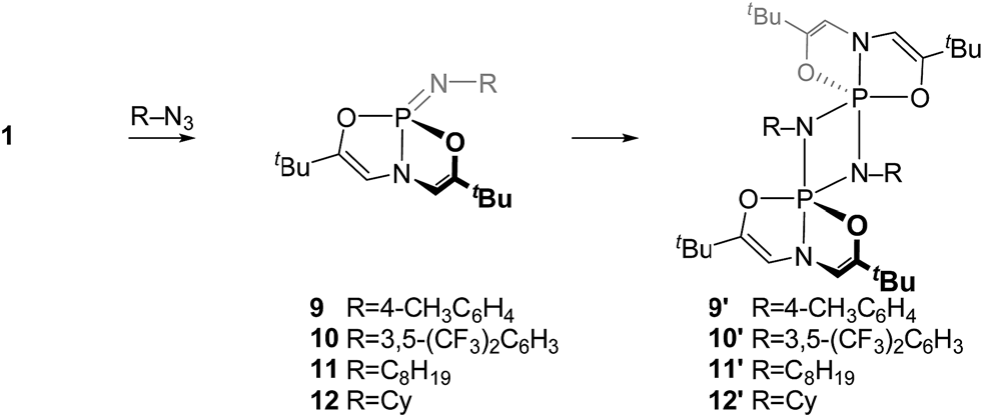
Synthesis of dimeric *O*,*N*,*O*-phosphazenes.

The identity of the imino *N*-substituent has a controlling effect on the rate and position of monomer–dimer equilibrium for phosphazenes based on *O*,*N*,*O*-platform **1**. Whereas the *N*-dipp substituted phosphazene could be isolated in either monomeric (**3**) or dimeric (**3′**) forms, *O*,*N*,*O*-phosphazenes bearing less sterically encumbered *N*-substituents could be isolated only in their dimeric cyclodiphosphazane forms (Scheme 4). In a typical preparation, treatment of **1** with 1 equiv. of organoazide in C_6_D_6_ at 60 °C for 16 h gave only dimeric cyclodiphosphazanes with ^31^P NMR chemical shifts characteristic of five-coordinate phosphorus (Table 3). *In situ*^31^P NMR experiments show that monomeric phosphazenes bearing *N*-tolyl (**9**, *δ* 9.9 ppm) moieties are detected as transient intermediates in the overall process, converting ultimately to the dimeric cyclodiphosphazane (**9′**, *δ* –44.6 ppm). By contrast, phosphazenes with electron-withdrawing (3,5-bis(trifluoromethyl)phenyl, **10′**, *δ* –44.0 ppm) and alkyl (*n*-octyl, **11′**, *δ* –39.6 ppm; cyclohexyl, **12′**, *δ* –38.1 ppm) *N*-substituents were observed only in their dimeric cyclodiphosphazane forms.

**Table 3:** ^31^P NMR chemical shifts of phosphazene monomers and dimers^*a*^

Compound	*δ* (monomer)^*b*^	*δ* (dimer)^*b*^
**3**	7.3	–26.9
**9**	9.9	–44.6
**10**	—^*c*^	–44.0
**11**	—^*c*^	–39.6
**12**	—^*c*^	–38.1

^*a*^Chemical shift referenced to 85% H_3_PO_4_ (*δ* = 0.0 ppm).

^*b*^Spectra recorded in C_6_D_6_ at 293 K.

^*c*^Monomer not observed.

**Fig. 5:**
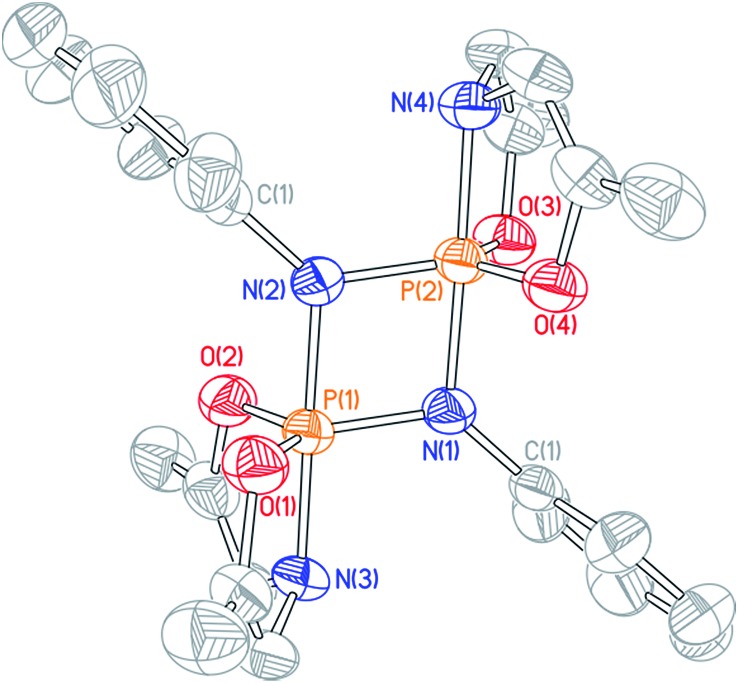
Molecular structure of **9′** with 50% probability level rendered thermal ellipsoid plot. All hydrogen and methyl carbon atoms removed for clarity. Selected bond lengths [Å] and angles [°]: P(1)–N(1) 1.635(3), P(1)–N(2) 1.744(3), P(1)–N(3) 1.746(3), P(1)–O(1) 1.637(3), P(1)–O(2) 1.641(3), N(2)–P(1)–N(3) 179.47(17), N(1)–P(1)–N(3) 99.64(16), O(1)–P(1)––O(2) 123.82(16).

The ^31^P NMR resonances of cyclodiphosphazanes **9′–12′** appear at noticeably lower field compared to **3′**, suggesting a geometric distinction between the very bulky 2,6-diisopropyl-substituted cyclodiphosphazane and less sterically demanding congeners. Single crystals of dimeric *N-p*-tolyl derivative **9′** were obtained by slow evaporation of a benzene solution, and the structure was interrogated by X-ray diffraction (Fig. 5). Globally, compound **9′** adopts the same planar 1,3-diaza-2,4-diphosphetidine P_2_N_2_ diamond core structure as **3′**. There are, however, notable metrical differences; the axial P–N (*d*_P–N_ = 1.744(3) Å) and equatorial P–N (*d*_P–N_ = 1.635(3) Å) bond lengths for *N-p*-tolyl cyclodiphosphazane **9′** are approximately 0.04 Å shorter than for *N*-dipp cyclodiphosphazane **3′**. We infer that the reduced steric congestion about the P_2_N_2_ core for **9′** permits tighter association of the monomer subunits in a head-to-tail fashion that is manifest in the ^31^P isotropic chemical shielding differences. Congruent with this assessment, attempts to access monomeric phosphazenes **9–12** by heating of benzene solutions of **9′–12′** were unsuccessful; formal (2 + 2) dimerization appears to be prohibitively downhill in enthalpy and, therefore, irreversible for these less sterically congested cyclodiphosphazanes.

Formal [2 + 2]-cyclodimerization of phosphazene P=N units is well-precedented in the literature, specifically for phosphazenes bearing strongly electron-withdrawing *P*-substituents or where relief of ring strain provides a driving force.^26^ That geometrically constrained *O*,*N*,*O*-phosphazenes (**9′–12′**) readily dimerize is thus a qualitative indication of the marked Lewis acidity of these phosphorus centers as compared to their acyclic congeners. Furthermore, the sum of the observations concerning the monomer–dimer speciation of distorted phosphazenes **3**/**3’** evidences a propensity for 1,2-ambiphilic reactivity of the P=N unit, suggesting that intermolecular 1,2-additions of exogenous reagents might be feasible.

### 2.4. 1,2 addition reactivity of nontrigonal phosphazenes

#### 2.4.1 B–H addition reactions

To investigate reactivity of the P=N unit in deformed phosphazenes **3** and **4**, we elected to attempt the 1,2-addition of σ-bonded E–H small molecules. To this end, treatment of *O*,*N*,*O*-phosphazene **3** with 1 equiv. of pinacolborane (HBpin) in C_6_D_6_ at room temperature resulted in rapid consumption of the starting materials and formation of a single new compound with a ^31^P NMR resonance at *δ* –43.7 ppm presenting as a doublet of triplets (*J* = 837, 33 Hz). The magnitude of the larger coupling constant is indicative of a direct P–H linkage; existence of a P–H moiety was confirmed in ^1^H NMR spectra by the appearance of a doublet centered at *δ* 9.37 ppm with complementary coupling (^1^*J*_P–H_ = 837 Hz). Although attempts to obtain a solid state structure of **13** from X-ray diffraction analysis were unsuccessful, these spectroscopic signatures are consistent with formation of hydrido amido phosphorane **13** in which the *O*,*N*,*O*-chelate spans two apical and one equatorial position about a phosphorus-centered trigonal bipyramid, and the hydride and borylamide substituents reside in the equatorial plane. Further support for this assignment comes by way of analogy to previous results from our group. We reported previously that hydrido amido phosphoranes related to **13** are accessible *via* intermolecular N–H oxidative addition to **3**.^10^ More specifically, the addition of 2,6-diisopropylaniline to **3** gave a crystallographically characterized oxidative addition product (*i.e.* the *des*-boryl congener of **13**) that exhibits spectroscopic features (^31^P *δ* –51.4 ppm; ^1^*J*_P–H_ = 841 Hz) in close agreement with those obtained for **13**. In short, the combined data lend strong evidence to the structural assignment of the B–H addition product **13**.

**Scheme 5:**
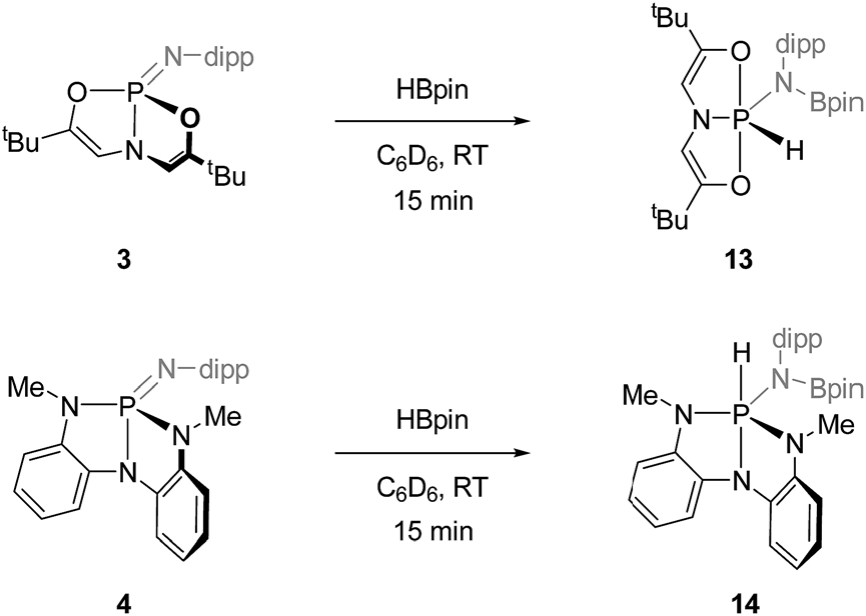
B–H additions to **3** and **4** (dipp = 2,6-diisopropylphenyl).

Likewise, treating *N*,*N*,*N*-phosphazene **4** with 1 equiv. of pinacolborane in C_6_D_6_ at ambient temperature quickly consumed starting materials to yield a species **14** with a doublet ^31^P NMR signal at *δ* –37.6 ppm (doublet, ^1^*J*_P–H_ = 579 Hz). Complementary coupling was observed in the ^1^H NMR spectrum with a doublet resonance at *δ* 6.36 ppm. As with the reaction of **3** and HBpin, these spectral data are characteristic of pentacoordinated hydrido amido phosphorane featuring a direct P–H bond. By analogy to previous results on the intermolecular N–H oxidative addition of 2,6-diisopropylaniline to **4**,^11^ we posit the assignment of **14** as in Scheme 5 with a folded non-meridional *N*,*N*,*N*-ligand, equatorial *N*-dipp substituent, and an axial hydride.

By contrast to **3** and **4**, acyclic *O*,*N*,*O*- and *N*,*N*,*N*-phosphazenes **7** and **8** were unreactive with respect to pinacolborane under identical reaction conditions. Additionally, none of the dimeric cyclodiphosphazanes **9′–12′** were found to undergo reaction with HBpin. We conclude, therefore, that the ability of the phosphazenes **3** and **4** to undergo 1,2-addition of H–Bpin is dependent on the distorted structure enforced by the trianionic heteroatom supporting structures, and that access to the monomeric form of the phosphazene is critical for intermolecular addition.

#### 2.4.2 B–O addition reactions

**Scheme 6:**
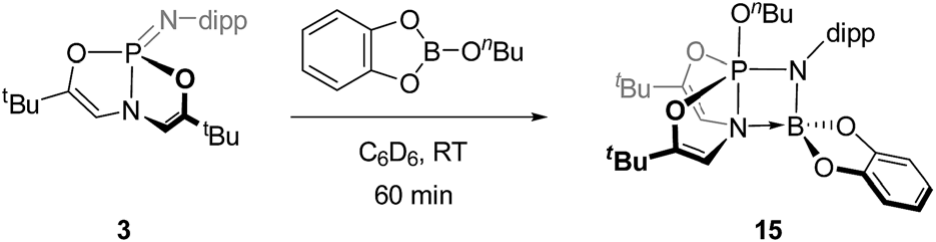
B–O addition to **3** (dipp = 2,6-diisopropylphenyl).

The deformed *O*,*N*,*O*-phosphazene **3** was also able to add B–O σ bonds across the P=N unit. Addition of 1 equiv. of *n*-butoxy catecholborane to a C_6_D_6_ solution of **3** at room temperature (Scheme 6) resulted in consumption of **3** with concomitant generation of a new ^31^P NMR singlet resonance at *δ* –31.4 ppm indicative of pentacoordinated phosphorus species **15**; scalar coupling could not be resolved. The corresponding ^1^H NMR spectrum featured a resonance at *δ* 5.33 ppm (doublet, ^3^*J*_P–H_ = 17 Hz), which can be attributed to coupling between the phosphorus center and the vinylic protons of the *O*,*N*,*O*-ligand. The magnitude of this coupling is smaller than would be expected for σ^5^-phosphorus compounds with a planar, meridional *O*,*N*,*O*-chelate. This result suggests that antiperiplanarity between P and H has been lost; that is, the dihedral angle between the phosphorus and vinylic hydrogen atoms has decreased from 180° in accordance with the Karplus equation for ^3^*J* scalar coupling,^27^ implying that the *O*,*N*,*O*-framework adopts a folded geometry.

**Fig. 6:**
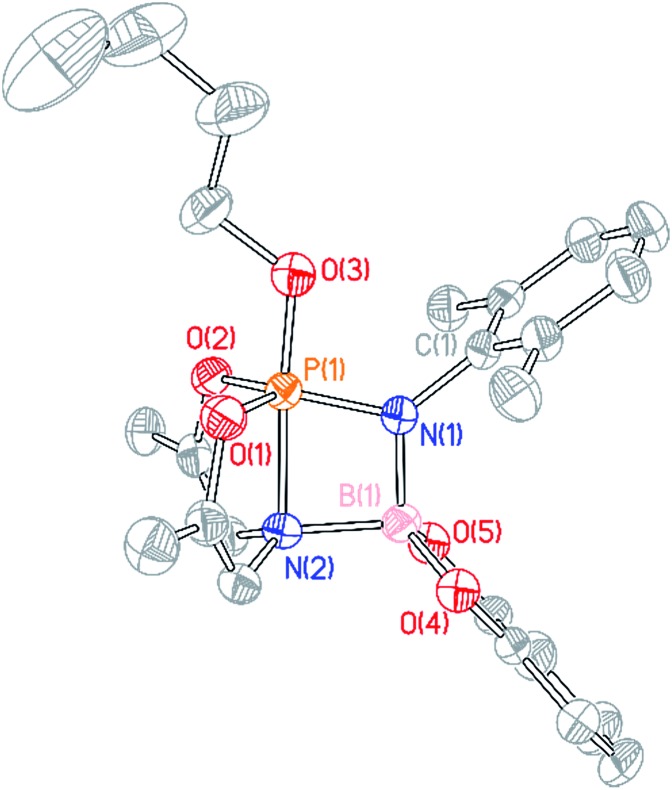
Molecular structure of **15** with 50% probability level rendered thermal ellipsoid plot. Hydrogen and methyl carbon atoms omitted for clarity. Selected bond lengths [Å] and angles [°]: P(1)–N(1) 1.6195(18), P(1)–N(2) 1.9392(19), P(1)–O(1) 1.6242(16), P(1)–O(2) 1.6252(16), P(1)–O(3) 1.5906(16), N(1)–B(1) 1.515(3), N(2)–B(1) 1.637(3); N(1)–P(1)–O(3) 98.16(9), N(1)–P(1)–N(2) 77.59(8), N(2)–P(1)–O(3) 175.75(8), O(1)–P(1)–O(2) 113.38(9), N(1)–B(1)–N(2) 90.68(15).

A single crystal suitable for X-ray diffraction was grown from benzene solution, and the solid state structure corroborates the above conclusion regarding *O*,*N*,*O*-folding (Fig. 6). A distinguishing feature of compound **15** is the unexpectedly short distance between the ligand amido N atom and the *N*-dipp-bound boron (*d*_N2–B1_ = 1.637(3) Å), consistent with the presence of a dative interaction N2 → B1 in **15**; pyramidalization of the boron atom evident in the solid state structure further evidences this conclusion. By consequence of this interaction, compound **15** may be viewed as a trigonal bipyramidal phosphorus compound supported by a tetracoordinating boroazaphosphatrane ligand, where the fifth apical binding site of the trigonal bipyramid is occupied by the *n*-butoxy substituent. Indeed, the apical bond distance *d*_P1–N2_ is quite long (1.9392(19) Å), conforming to precedent from cationic phosphatranes^28^ (*cf. d*_P–N_ = 1.986(5) Å for [HP(OCH_2_CH_2_)_3_N][BF_4_];29*a**d*_P–N_ = 1.967(8) Å for [HP(MeNCH_2_CH_2_)_3_N][BF_4_]29*b*). Additionally, the apical P1–N2 bond in **15** is substantially longer than the equatorial P1–N1 bond (1.6195(18) Å), comprising a difference of more than 0.3 Å. As a consequence, the *trans* apical P1–O3 bond is found to be quite short (1.5906(16) Å); in fact, the apical P–O bond is shorter than the equatorial P–O bonds (1.6242(16) Å and 1.6252(16) Å). This observation runs counter to typical trigonal bipyramidal geometries, where the 3-center, 4-electron apical bonds are usually longer than the 2-center, 2-electron equatorial bonds. These unusual structural features of the B–O adduct of phosphazene **3** likely arise from molecular constraint imposed by the *O*,*N*,*O*-ligand and by the Lewis acidic boron atom.

Unlike phosphazene **3**, *N*,*N*,*N*-ligated phosphazene **4** did not react with *n*-butoxy catecholborane, perhaps again due to increased steric congestion about the P=N unit. Likewise, acyclic analogues **7** and **8** were unreactive to B–O bonds.

#### 2.4.3 Si–H addition reactions

**Scheme 7:**
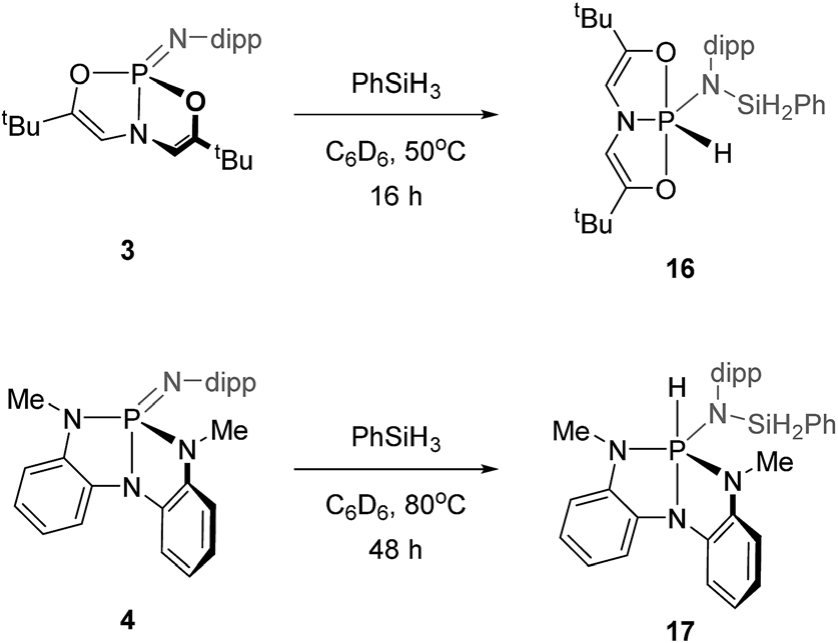
Si–H addition to **3** and **4** (dipp = 2,6-diisopropylphenyl).

In addition to activating B–H and B–O bonds, phosphazene **3** was reactive toward the Si–H bond of phenylsilane. Treating phosphazene **3** with phenylsilane in C_6_D_6_ at 50 °C for 16 h (Scheme 7) produced compound **16**, which in the ^31^P NMR spectrum displayed a doublet of triplets centered at *δ* –44.2 ppm (*J* = 802 Hz, 31 Hz). Likewise, a doublet resonance with a large coupling constant centered at *δ* 9.44 ppm (^1^*J*_P–H_ = 802 Hz) was observed in the ^1^H NMR spectrum. Another complementary doublet signal, corresponding to ligand vinylic protons, appeared at *δ* 5.34 ppm (^3^*J*_P–H_ = 31 Hz). Analogous to other σ^5^,λ^5^-phosphorus compounds synthesized here (*vide supra*), the coupling constant of 31 Hz is indicative of a planar *O*,*N*,*O*-chelate, as in Scheme 7. Thus, these NMR spectra indicate that the Si–H bond of phenylsilane added across the P=N bond of **3** to give a hydrido phosphorane with a *N*-silyl substituent.

*N*,*N*,*N*-Phosphazene **4** also reacted with phenylsilane, but at a slower rate than **3**. The conversion of **4** and phenylsilane to hydrido phosphorane **17** was completed after 48 h of heating at 80 °C in C_6_D_6_. The resulting ^31^P NMR spectrum showed one signal centered at *δ* –29.1 ppm (d, ^1^*J*_P–H_ = 535 Hz); the magnitude of the scalar coupling constant is characteristic of a P–H bond, consistent with the structure of the σ^5^-phosphorus Si–H addition product **17**. By analogy to other *N*,*N*,*N*-ligated compounds synthesized earlier, we expect the trianionic chelate to adopt a folded structure, as in Scheme 7.

Acyclic phosphazenes **7** and **8** were found to be unreactive to Si–H addition. The fact that compounds of this type are robust to silane addition has been exploited by Fontaine, who has shown that phosphazenes similar to **8** can be used as Lewis base-catalysts for catalysed hydrosilylation of CO_2_.^30^

The 1,2-addition of hydrosilane across the P=N unit of a phosphazene has often been invoked, albeit usually in tandem with subsequent elimination from P(v) and without direct observation of the elementary step. Kawashima has studied the intramolecular addition of a Si–H bond to a functionalized phosphazene.^31^ Phosphine-catalyzed variants of the Staudinger reduction of azides are believed to rely on the addition of hydrosilane to phosphazene intermediates to close the catalytic cycle.^32^ Relatedly, Denton has demonstrated that a phosphine-catalyzed Staudinger amidation reaction relies on the *in situ* reduction of a phosphazene, presumably initiated by 1,2-addition of hydrosilane.^33^ In each of these chemistries the silane reduction step likely proceeds *via* 1,2-addition *via* a trigonal bipyramidal hydrido amido phosphorane intermediate, but despite the prevalence of the 1,2-addition proposal, there do not exist discrete, well-characterized analogues of this key step. Our current results represent a rare well-defined addition reaction giving rise to stable pentacoordinate adducts that substantiate the notion of 1,2-addition of hydrosilanes to phosphazenes.

## 3. Conclusions

Constrained phosphabicyclic phosphazenes supported by trianionic scaffolding *O*,*N*,*O*- and *N*,*N*,*N*-ligands exhibit increased cyclodimerization and 1,2-addition reactions of B–H, B–O, and Si–H σ bonds as compared to acyclic congeners. The results of the combined experimental and theoretical studies above support the conclusion that nontrigonal distortion of phosphazenes leads to an enhancement of the 1,2-ambiphilic reactivity of the P=N unit. In view of the multivarious roles of phosphazenes, the ability to master electronic structure and reactivity as a function of a modifiable parameter contributes to the discovery of novel applications. In conjunction with established approaches to synthetic tuning through substituent effects, the current results establish a rational geometry-based framework for modulating the reactivity of this important class of main group compounds, which may be leveraged in the design of functionally novel entities.

## 4. Experimental section

Full experimental details are available in the online ESI.†

### 4.1. Synthesis of **3**

A solution of 1 (100 mg, 0.42 mmol) with 2,6-diisopropylphenyl azide (85 mg, 0.42 mmol) in C_6_D_6_ (1 mL) was stirred at 40 °C for 16 h. All volatiles were removed *in vacuo*, and the resulting residue was triturated with pentane. The crude product was obtained after filtration, and pure 3 was isolated as a white solid by recrystallization from a 10 : 1 dichloromethane/pentane solution (131 mg, 75% yield). ^1^H NMR (C_6_D_6_, 400 MHz): *δ* 7.23–7.21 (m, 2H), 7.12–7.07 (m, 1H), 7.07 (d, 1H, *J* = 7.7 Hz), 5.49 (d, 2H, *J* = 28.8 Hz), 3.83 (hept, 1H, *J* = 6.9 Hz), 1.40 (d, 12H, *J* = 6.8 Hz), 0.91 (s, 18H) ppm. ^13^C NMR (C_6_D_6_, 126 MHz): *δ* 154.19, 141.29 (d, *J* = 8.7 Hz), 123.03, 121.98, 112.43 (d, *J* = 10.1 Hz), 32.64 (d, *J* = 8.6 Hz), 29.35, 26.89, 23.75 ppm. ^31^P NMR (C_6_D_6_, 162 MHz): *δ* 7.21 (t, *J* = 28.8 Hz) ppm. MS (ESI) calc'd for C_24_H_37_N_2_O_2_P (M^+^) 416.2593, found 416.2596.

### 4.2. Synthesis of **4**

A solution of 2 (100 mg, 0.39 mmol) with 2,6-diisopropylphenyl azide (80 mg, 0.39 mmol) in C_6_D_6_ (1 mL) was stirred under ambient temperature for 12 h. The solvent was removed *in vacuo*, and the resulting solid product was recrystallized from a 10 : 1 dichloromethane/pentane solution (121 mg, 72% yield). ^1^H NMR (C_6_D_6_, 400 MHz): *δ* 7.26 (d, *J* = 7.7 Hz, 2H), 7.16 (d, *J* = 7.4 Hz, 2H), 7.09–7.02 (m, 1H), 6.98–6.85 (m, 3H), 6.77 (d, *J* = 7.6 Hz, 3H), 6.23 (d, *J* = 7.7 Hz, 2H), 3.65 (hept, *J* = 6.9 Hz, 2H), 2.73 (d, *J* = 9.2 Hz, 6H), 1.22 (d, *J* = 6.9 Hz, 12H) ppm. ^13^C NMR (C_6_D_6_, 125 MHz): *δ* 141.38, 141.00 (d, *J* = 11.7 Hz), 138.09 (d, *J* = 17.6 Hz), 134.58 (d, *J* = 13.0 Hz), 124.56, 123.26, 121.34, 120.14, 116.34 (d, *J* = 10.2 Hz), 108.66 (d, *J* = 10.0 Hz), 29.29 (d, *J* = 18.9 Hz), 23.75 ppm. ^31^P NMR (C_6_D_6_, 162 MHz): *δ* 14.54 ppm. MS (ESI) calc'd for C_26_H_31_N_4_P (M^+^) 430.2286, found 430.2290.

### 4.3. Representative B–H addition

A solution of 3 (30 mg, 0.07 mmol) and HBpin (9 mg, 0.07 mmol) in C_6_D_6_ (0.3 mL) was stirred at ambient temperature for 15 min. The solvent was removed *in vacuo* to afford the crude product (37 mg, 95% crude yield). ^1^H NMR (C_6_D_6_, 500 MHz): *δ* 9.38 (d, 1H, *J* = 837.7 Hz), 7.27–7.25 (m, 1H), 7.08–7.04 (m, 2H), 5.49 (d, 2H, *J* = 33.2 Hz), 3.62–3.56 (m, 2H), 1.53 (d, 6H, *J* = 6.6 Hz, 1.45) (d, 6H, *J* = 6.6 Hz, 6H), 1.19 (s, 12H), 1.15 (s, 18H) ppm. ^13^C NMR (C_6_D_6_, 126 MHz): *δ* 150.73 (d, *J* = 6.4 Hz), 147.14, 126.64, 124.52 (d, *J* = 4.4 Hz), 124.02, 123.42, 100.51 (d, *J* = 18.8 Hz), 82.52, 31.69, 28.25, 27.51, 24.66, 23.62, 23.26 ppm. ^31^P NMR (C_6_D_6_, 203 MHz): *δ* –43.02 (dt, *J* = 837.0, 31.5 Hz) ppm. MS (ESI) calc'd for C_30_H_49_BN_2_O_4_P (M–H^+^) 543.3518, found 543.3523.

### 4.4. Representative Si–H addition

A solution of 4 (50 mg, 0.12 mmol) and phenylsilane (13 mg, 0.12 mmol) in C_6_D_6_ (0.3 mL) was stirred at 80 °C for 48 h. All volatiles were removed *in vacuo* to afford 20 as a yellow oil (62 mg, 99% crude yield). ^1^H NMR (C_6_D_6_, 500 MHz): *δ* 7.32 (d, *J* = 7.1 Hz, 2H), 7.14–6.99 (m, 7H), 6.95 (t, *J* = 7.2 Hz, 3H), 6.87 (t, *J* = 7.7 Hz, 2H), 6.73 (t, *J* = 7.8 Hz, 2H), 6.37 (d, *J* = 7.5 Hz, 2H), 5.75 (d, *J* = 534.0 Hz, 1H), 5.05 (d, *J* = 5.8 Hz, 2H), 3.45–3.33 (m, 2H), 2.49 (d, *J* = 15.9 Hz, 6H), 1.15 (d, *J* = 6.8 Hz, 6H), 1.06 (d, *J* = 6.7 Hz, 6H) ppm. ^13^C NMR (C_6_D_6_, 126 MHz): *δ* 148.30 (d, *J* = 5.8 Hz), 137.93, 135.77 (d, *J* = 10.4 Hz), 134.65 (d, *J* = 14.5 Hz), 134.25, 133.46, 129.01, 127.16 (d, *J* = 3.6 Hz), 123.90 (d, *J* = 4.0 Hz), 121.42, 119.38, 113.89 (d, *J* = 10.8 Hz), 108.72 (d, *J* = 7.6 Hz), 29.69 (d, *J* = 15.5 Hz), 27.61, 26.24, 23.84 ppm. ^31^P NMR (C_6_D_6_, 203 MHz): *δ* –28.54 (dt, *J* = 534.5, 17.2 Hz) ppm. MS (ESI) calc'd for C_32_H_40_N_4_PSi (M + H^+^) 539.2760, found 539.2763.

## Conflicts of interest

There are no conflicts to declare.

## Supplementary Material

Supplementary information

Crystal structure data
