# Supplementary material for: Nontrigonal constraint enhances 1,2-addition reactivity of phosphazenes
Source: Chem Sci. 2018 Apr 6;9(18):4338–47. doi: 10.1039/c8sc00929e (PMC5944378; doi:10.1039/c8sc00929e)
Supplement: Supplementary file 1 [file SC-009-C8SC00929E-s001.pdf]

Electronic Supplementary Material (ESI) for Chemical Science.
This journal is © The Royal Society of Chemistry 2018

Nontrigonal Constraint Enhances 1,2-Addition Reactivity of Phosphazenes

Yi-Chun Lin†, James C. Gilhula†, and Alexander T. Radosevich*

Department of Chemistry, Massachusetts Institute of Technology, Cambridge, MA 02139

Contents

I. General Materials and Methods ... S2
II. Synthesis of **1** ... S3
III. Synthesis of **2** ... S4
IV. Synthesis of Distorted and Acyclic Phosphazenes ... S6
V. General Synthetic Procedure for Azides ... S12
VI. Crystallographic Procedures and Data ... S13
VII. Multinuclear NMR Spectra ... S123
VIII. DFT Calculations ... S170
IX. References ... S219

I. General Materials and Methods

All reagents were purchased from Sigma-Aldrich, Alfa Aesar, ACROS, TCI, or Oakwood Chemical, and used as received unless otherwise noted. Diethyl ether (Et_2O), methylene chloride (CH_2Cl_2), tetrahydrofuran (THF), and pentane were dried according to the method of Grubbs[1] as modified by Bergman[2] using a Glass Contour Solvent Purification System. All glassware was oven-dried at 120°C prior to use. All reactions were carried out under dry nitrogen atmosphere (Schlenk line or glovebox) unless otherwise noted. NMR spectra were recorded on a Bruker AV-360 (360MHz), a Bruker AV-400 (400 MHz), a Bruker AV-500 (500MHz) or a VARIAN Inova-500 (500MHz) spectrometer. ^{1}H NMR chemical shifts are given in ppm with respect to solvent residual peak (C_6D_6, δ 7.16 ppm; $CDCl_3$, δ 7.26 ppm; CD_2Cl_2, δ 5.32 ppm), ^{13}C{^{1}H} NMR shifts are given in ppm with respect to (C_6D_6 δ 128.06 ppm; $CDCl_3$ δ 77.16 ppm; CD_2Cl_2 δ 53.84 ppm). Coupling constants are reported as *J*-values in Hz. High resolution EI and ESI mass spectra were obtained from the Mass Spectrometry Laboratory at the School of Chemical Sciences, University of Illinois at Urbana-Champaign, or Department of Chemistry Instrumentation Facility, Massachusetts Institute of Technology. X-ray diffraction data was collected on a Bruker SMART APEX CCD area detector system equipped with a graphite monochromator and a MoKα fine-focus sealed tube (λ = 0.71073 Å). Raw data integration and reduction were performed with the SAINT[3] and SADABS[4] programs. Structures were solved by direct methods using SHELXS[5] and refined by least-squares methods on F^2 using SHELXL-2018 with the WinGX[6] software package. All non-hydrogen atoms were refined with anisotropic displacement parameters. Hydrogen atoms were fixed in their ideal geometries. XP was used for graphical representations.

II. Synthesis of 1

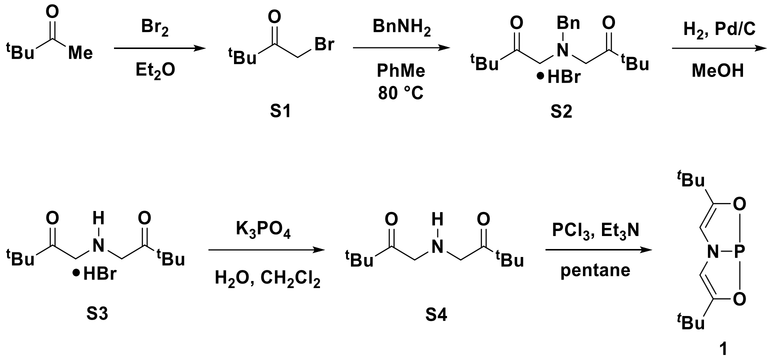

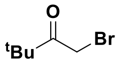

S1

The following procedure is adapted from the literature.[7] To a diethyl ether of solution (80 ml) of pinacolone (50.0 g, 499 mmol) was added several drops of bromine at ambient temperature. The orange solution became colorless and was subsequently cooled in an ice bath. Bromine (26 mL, 499 mmol) was added dropwise over an hour, maintaining the internal temperature below 10°C. The reaction mixture was stirred an additional 30 min, then 200 mL of water were added and stirred for 15 min. The reaction mixture was warmed to room temperature and treated with solid sodium bicarbonate (42 g, 499 mmol), and the resulting phasic layers were separated. The ether layer was washed with brine (2x50 mL), dried (Na_2SO_4), and concentrated *in vacuo*. Purification by vacuum distillation yielded **S1** as a light yellow oil (78 g, 87%). ^{1}H NMR (300 MHz, $CDCl_3$): δ 4.18 (2H, s), 1.23 (9H, s) ppm.

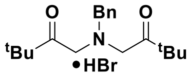

S2

To a toluene (900 ml) solution of **S1** (70.3 g, 393 mmol) was added benzylamine (43 mL, 339 mmol) in one portion. The reaction mixture was stirred at 80°C for 48 h, during which time a voluminous white solid precipitated. The solid was collected on a Buchner funnel, washed with water (2x100 mL), and recrystallized in ethanol to give **S2** as a white crystalline solid (62 g, 82%). ^{1}H NMR (360 MHz, $CDCl_3$): δ 7.66 (2H, m), 7.46 (3H, m), 4.94 (2H, m), 4.72 (4H, m), 1.14 (18H, s) ppm.

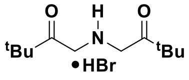

S3

To a suspension of **S2** (60.0 g, 150 mmol) in nitrogen-sparged methanol (500 mL) was added 10% palladium on carbon (600 mg, 0.6 mmol). The atmosphere was exchanged for hydrogen via three evacuation/backfill cycles. The heterogeneous mixture was stirred under H_2 (1 atm) for 24 hours. The reaction mixture was then filtered over celite and the filtrate was

concentrated. Recrystallization of the crude solid product from ethanol gave **S3** as a white solid (40.5 g, 90%). ^{1}H NMR (360 MHz, $CDCl_3$): δ 4.31 (4H, s), 1.23 (18H, s) ppm.

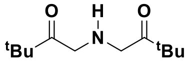
S4

A mixture of **S3** (30.0 g, 102 mmol), water (400 mL) and dichloromethane (300 mL) was cooled in an ice bath. An aqueous solution of tribasic potassium phosphate (26.0 g, 122 mmol, 270 mL, 0.46 M) was added dropwise over 30 minutes, then the solution was stirred for an additional 2 hours in an ice bath. The aqueous layer was extracted with dichloromethane (3x100 mL), then the dichloromethane extracts were washed with water (3x200 mL). The organic layer was dried (Na_2SO_4) and concentrated *in vacuo* to give **S4** as a yellow solid (16.0 g, 90%), which was stored at –30 °C prior to further use. ^{1}H NMR (400 MHz, $CDCl_3$): δ3.62 (4H, s), 2.83 (1H, br s), 1.15 (18H, s) ppm.

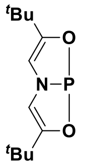
1

At –78°C, to a pentane solution (150 mL) of phosphorus (III) chloride (4.1 mL, 47 mmol) under nitrogen was added **S4** (10.0 g, 48 mmol) in pentane (130 mL) dropwise. After a pentane solution (100 mL) of triethylamine (20.5 mL, 147 mmol) was added dropwise, the resulting mixture was stirred at -78°C for 4 hours. The mixture was then warmed to room temperature and stirred for an additional 2 hours. The reaction mixture was concentrated to a residue *in vacuo* and transferred to a glovebox. The crude reaction residue was then triturated with pentane (200 mL) and filtered over celite. The filtrate was concentrated to give crude **1** and further recrystallization from pentane at –35°C afforded pure **1** as a yellow solid (4.6 g, 73%). ^{1}H NMR (400 MHz, $CDCl_3$): δ 7.40 (2H, d, *J*=9.6 Hz), 1.27 (18H, s) ppm. ^{31}P{^{1}H} NMR (145 MHz, $CDCl_3$): 187.7 ppm.

III. Synthesis of 2

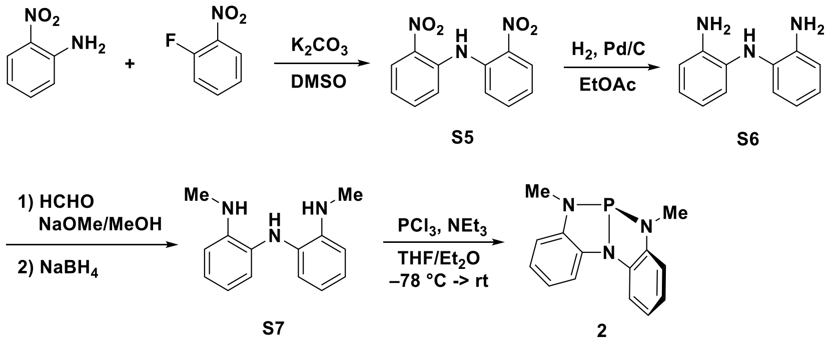

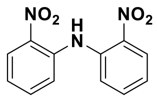

S5

Synthesized according to literature procedure.[8] 1-Fluoro-nitrobenzene (20.0 mL, 190 mmol), 2-nitroaniline (26.2 g, 190 mmol) and K_2CO_3 (31.4 g, 228 mmol) were mixed in DMSO (300 mL). The reaction mixture was stirred at 120°C for 36 h. H_2O was then added (300 mL) and the mixture was extracted with dichloromethane (3 x 500 mL). The combined organic layers were washed with a saturated aqueous solution of NaCl (6 x 300 mL, 15%), dried over anhydrous magnesium sulfate, and concentrated *in vacuo*. The product was obtained as an orange solid (46.5 g, 95%) and was used in the next step without further purification. ^{1}H NMR (360 MHz, $CDCl_3$): δ 10.98 (s, 1H), 8.16 (dd, 2H, J = 8.4 Hz, J = 1.3 Hz), 7.58-7.48 (m, 4H), 7.09-7.04 (m, 2H) ppm.

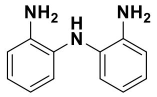

S6

Bis(2-nitrophenyl)amine **S1** (10 g, 38.5 mmol) was suspended in EtOAc (100 mL). Palladium on carbon (790 mg, 10 wt%) was then added to the solution. The reaction mixture was transferred to a high pressure reactor, charged with hydrogen (400 psi), and stirred for 10 h. Upon completion, the solution was filtered through Celite and concentrated to a dark residue under vacuum, which was used in the next step without further purification (5.43 g, 94%). ^{1}H NMR (360 MHz, $CDCl_3$): δ 6.97-6.92 (m, 2H), 6.80-6.74 (m, 6H), 5.00 (br s, 1H), 3.74 (br s, 4H) ppm.

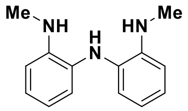

S7

To a mixture of bis(2-aminophenyl)amine **S6** (10 g, 50.1 mmol) and paraformaldehyde (14.8 g, 494 mmol) in anhydrous methanol (500 mL) was added a solution of NaOMe in methanol (34.5 mL, 25 wt. %) slowly at 0°C. The mixture was then stirred under reflux for 1 h. After being cooled to 0 °C, $NaBH_4$ (20.6 g, 545 mmol) was added in small portions. The solution was again stirred with heating to reflux for 1 h. The reaction mixture was then cooled to room temperature. To this mixture, 1 M NaOH (150 mL) was added followed by extraction with dichloromethane (3 × 300 mL). The combined organic phases were dried over magnesium sulfate and filtered. The crude mixture was concentrated under vacuum and flushed through a short column of silica gel with dichloromethane. After concentration, the resulting crude solid was recrystallized from ethanol to give product as purple crystals (9.8 g, 87%). ^{1}H NMR (360 MHz, $CDCl_3$): δ 7.07 (m, 2H), 6.77-6.71 (m, 6H), 4.91 (s, 1H), 3.76 (br s, 2H), 2.88 (s, 6H) ppm.

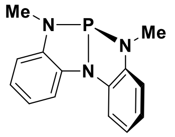

2

Phosphorus trichloride (1.15 mL, 13.2 mmol) was dissolved in ether (40 mL) and cooled to –78°C. A solution of triamine **S7** (3.00 g, 13.2 mmol) in THF (10 mL) was added dropwise via syringe. Upon completion, an ether solution (36 mL) of triethylamine (5.60 mL, 40.2 mmol) was added slowly via syringe. The reaction mixture was stirred at –78°C for 1h and then warmed to room temperature. After 4h of stirring at room temperature, all volatiles were removed *in vacuo,* and the resulting solid mixture was brought into a nitrogen-filled glovebox. The solid mixture was stirred in pentane and filtered through Celite to remove all insoluble solids. Pentane was removed *in vacuo* and the resulting crude product was recrystallized from pentane in a freezer (ca. –35°C) to yield **1** as light-pink crystals (2.5 g, 75 %). ^{1}H NMR (C_6D_6, 400 MHz): δ 7.38 (dd, 1H, *J* = 7.7, 1.3 Hz), 6.94 (td, 1H, *J* = 7.6, 1.2 Hz), 6.77 (td, 1H, *J* = 7.6, 1.3 Hz), 6.34 – 6.29 (m, 1H), 2.52 (d, 3H, *J* = 8.3 Hz). ^{31}P{^{1}H} NMR (C_6D_6, 162 MHz): δ 159.8 ppm.

IV. Synthesis of Distorted and Acyclic Phosphazenes

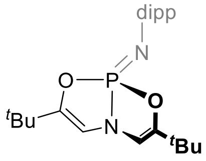

3

To a C_6D_6 solution of **1** (100 mg, 0.42 mmol) was added 2,6-diisopropylphenyl azide (85 mg, 0.42 mmol) and the reaction was stirred at 60°C for 16 h. All volatiles were removed *in vacuo* and the resulting residue was triturated with pentane. The crude product was obtained after filtration, and pure **3** was isolated as a white solid by recrystallization from a 10:1 dichloromethane/pentane solution (131 mg, 75% yield). ^{1}H NMR (C_6D_6, 400 MHz): δ 7.23-7.21 (m, 2H), 7.12-7.07 (m, 1H), 7.07 (d, 1H, *J* = 7.7 Hz), 5.49 (d, 2H, *J* = 28.8 Hz), 3.83 (hept, 1H, *J* = 6.9 Hz), 1.40 (d, 12H, *J* = 6.8 Hz), 0.91 (s, 18H) ppm. ^{13}C NMR (C_6D_6, 126 MHz): δ 154.19, 141.29 (d, *J* = 8.7 Hz), 123.03, 121.98, 112.43 (d, *J* = 10.1 Hz), 32.64 (d, *J* = 8.6 Hz), 29.35, 26.89, 23.75 ppm. ^{31}P NMR (C_6D_6, 162 MHz): δ 7.21 (t, *J* = 28.8 Hz) ppm. MS (ESI) calc'd for $C_{24}H_{37}N_2O_2P$ (M^+) 416.2593, found 416.2596.

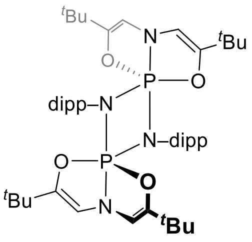

3'

A C_6D_6 solution of phosphine **1** (50 mg, 0.21 mmol) was charged to a J. Young NMR tube followed by addition of 2,6-diisopropylphenyl azide (43 mg, 0.21 mmol) and the reaction was held at 60°C for 3 d. Pure 3' was never observed in solution by NMR, but single crystals of 3' for X-ray crystallography were isolated by crystallization from a benzene solution.

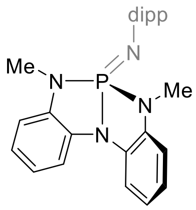

4

Treatment of **2** (100 mg, 0.39 mmol) with 1 equiv. pf 2,6-diisopropylphenyl azide (80 mg, 0.39 mmol) in C_6D_6 (1 ml) at ambient temperature for 12 h, followed by removal of solvent *in vacuo* afforded a solid which was recrystallized from a 10:1 dichloromethane/pentane solution to give pure product (121 mg, 72 % yield). ^{1}H NMR (C_6D_6, 400 MHz): δ 7.26 (d, *J* = 7.7 Hz, 2H), 7.16 (d, *J* = 7.4 Hz, 2H), 7.09 – 7.02 (m, 1H), 6.98 – 6.85 (m, 3H), 6.77 (d, *J* = 7.6 Hz, 3H), 6.23 (d, *J* = 7.7 Hz, 2H), 3.65 (hept, *J* = 6.9 Hz, 2H), 2.73 (d, *J* = 9.2 Hz, 6H), 1.22 (d, *J* = 6.9 Hz, 12H) ppm. ^{13}C NMR (C_6D_6, 125 MHz): δ 141.38, 141.00 (d, *J* = 11.7 Hz), 138.09 (d, *J* = 17.6 Hz), 134.58 (d, *J* = 13.0 Hz), 124.56, 123.26, 121.34, 120.14, 116.34 (d, *J* = 10.2 Hz), 108.66 (d, *J* = 10.0 Hz), 29.29 (d, *J* = 18.9 Hz), 23.75 ppm. ^{31}P NMR (C_6D_6, 162 MHz): δ 14.54 ppm. MS (ESI) calc'd for $C_{26}H_{31}N_4P$ (M^+) 430.2286, found 430.2290.

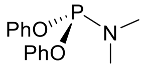

5

A THF solution of tris(dimethylamino)phosphine (0.5 g, 3 mmol) and phenol (0.29 g, 3 mmol) was refluxed for 20 hours. The solvent was removed from the reaction mixture to afford the product (0.78 mg, 99% yield). Product was used without further purification. ^{1}H NMR (C_6D_6, 400 MHz): δ 7.15 (d, *J* = 8.6 Hz, 4H), 7.07 (t, *J* = 7.9 Hz, 4H), 6.86 (t, *J* = 7.3 Hz, 2H), 2.53 (d, *J* = 9.4 Hz, 6H). ^{13}C NMR (C_6D_6, 101 MHz): δ 154.13 (d, *J* = 6.5 Hz), 129.54, 122.91, 120.15 (d, *J* = 8.5 Hz), 34.34 (d, *J* = 20.5 Hz). ^{31}P NMR (C_6D_6, 162 MHz): δ 139.7 (m) ppm.

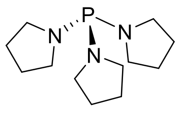

6

Compound **6** was prepared according to the literature method.[9] All spectral data were consistent with previously reported values.

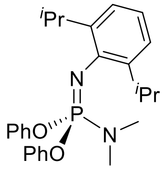

7

A CH_2Cl_2 solution of **5** (100 mg, 0.38 mmol) was treated with 2,6-diisopropylphenyl azide (78 mg, 0.38 mmol), and the reaction was stirred at room temperature for 16 h. All volatiles were removed *in vacuo,* and the resulting residue was triturated with pentane. The crude product was obtained after filtration, and the pure product was isolated as a white solid by recrystallization from a 10:1 dichloromethane/pentane solution (120 mg, 71% yield). ^{1}H NMR (C_6D_6, 400 MHz): δ 7.21 – 6.73 (m, 13H), 3.49 (hept, *J* = 6.9 Hz, 2H), 2.54 (d, *J* = 10.9 Hz, 6H), 1.15 (d, *J* = 6.9 Hz, 12H). ^{13}C

NMR (C_6D_6, 101 MHz): δ 151.95 (d, *J* = 8.1 Hz), 141.46 (d, *J* = 7.7 Hz), 129.39, 124.06, 122.89, 120.77, 120.15 (d, *J* = 5.2 Hz), 36.70 (d, *J* = 4.3 Hz), 28.17, 23.80. ^{31}P NMR (C_6D_6, 162 MHz): δ 8.22 (t, *J* = 10.8 Hz) ppm. MS (ESI) calc'd for $C_{26}H_{34}N_2O_2P$ (M+H$^+$) 437.2358, found 437.2363.

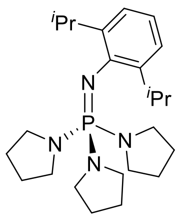

8

To a CH_2Cl_2 solution of tris(1-pyrrolidinyl)phosphine (60 mg, 0.25 mmol) was added 2,6-diisopropylphenyl azide (50 mg, 0.25 mmol), and the mixture was stirred at room temperature for 16 h. All volatiles were removed *in vacuo* and the resulting residue was triturated with pentane. The crude product was obtained after filtration, and the pure product was isolated as a yellow solid by recrystallization from a 10:1 dichloromethane/pentane solution (80 mg, 78 % yield). ^{1}H NMR (C_6D_6, 400 MHz): δ 7.26 (d, *J* = 7.5 Hz, 2H), 7.06 (t, *J* = 7.5 Hz, 1H), 3.92 (hept, *J* = 6.9 Hz, 2H), 3.12 – 2.93 (m, 12H), 1.56 – 1.45 (m, 12H), 1.39 (d, *J* = 6.9 Hz, 12H). ^{13}C NMR (C_6D_6, 126 MHz): δ 145.52 (d, *J* = 7.1 Hz), 141.35 (d, *J* = 7.0 Hz), 122.49, 118.57, 46.55 (d, *J* = 4.3 Hz), 27.93, 26.34 (d, *J* = 8.4 Hz), 24.24. ^{31}P NMR (C_6D_6, 162 MHz): δ 29.67 ppm. MS (ESI) calc'd for $C_{24}H_{42}N_4P$ (M+H$^+$) 417.3147, found 417.3145.

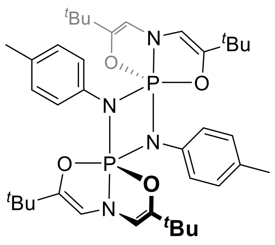

9'

A C_6D_6 solution of phosphine **1** (50 mg, 0.21 mmol) was charged to a J. Young NMR tube followed by addition of 2,4,6-trimethylphenyl azide (34 mg, 0.21 mmol), and the reaction was heated at 60°C for 16 h. All volatiles were removed in vacuo, and the resulting solid residue was washed three times with pentane to obtain **9'** (62 mg, 86% yield). Crystalline **9'** for X-ray crystallography was obtained by recrystallization from a benzene solution. ^{1}H NMR (C_6D_6, 500 MHz): δ 7.65 (d, 4H, J = 8.0 Hz), 7.11 (d, 2H, J = 8.0 Hz), 5.56-5.50 (m, 4H), 2.15 (s, 6H), 1.18 (s, 36H) ppm. ^{13}C NMR (C_6D_6, 126 MHz): δ 146.19, 138.32, 134.54, 130.27, 128.55, 111.38, 32.01, 27.53, 20.79 ppm. ^{31}P NMR (C_6D_6, 202 MHz): δ -44.62 (t, J= 14.1 Hz) ppm. MS (EI) calc'd for $C_{38}H_{55}N_4O_4P_2$ (M+H$^+$) 693.3693, found 693.3699.

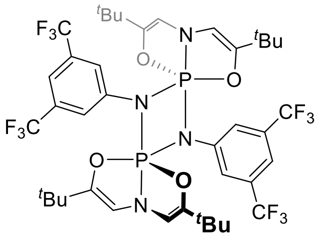

10'

A C_6D_6 solution of phosphine **1** (50 mg, 0.21 mmol) was charged to a J. Young NMR tube followed by addition of 3,5-bis(trifluoromethyl)phenyl azide (54 mg, 0.21 mmol), and the mixture was allowed to react at ambient temperature for 16 hours. **10'** was not isolated but was observed spectroscopically by ^{31}P. All of the starting phosphazene **3** was consumed, and the final reaction mixture contained excess azide. ^{31}P NMR (C_6D_6, 146 MHz): δ -44.04 (t, J= 14.1 Hz) ppm.

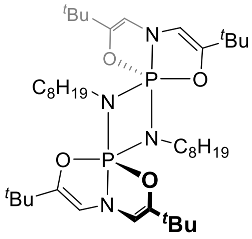

11'

A C_6D_6 solution of phosphine **1** (50 mg, 0.21 mmol) was charged to a J. Young NMR tube followed by addition of *n*-octyl azide (42 mg, 0.27 mmol), and the reaction was heated at 60°C for 60 h. **11'** was not isolated but was observed spectroscopically by ^{31}P and ^{1}H NMR. The final reaction mixture contained a small amount of starting phosphazene **3** in addition to excess azide. ^{31}P NMR (C_6D_6, 146 MHz): δ -39.61 (m) ppm.

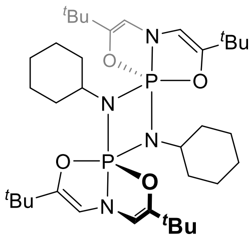

12'

A C_6D_6 solution of phosphine **1** (50 mg, 0.21 mmol) was charged to a J. Young NMR tube followed by addition of cyclohexyl azide (31 mg, 0.25 mmol), and the reaction was heated at 60°C for 60 h. **12'** was not isolated but was observed spectroscopically by ^{31}P NMR. ^{1}H NMR data displayed a complex reaction mixture. ^{31}P NMR (C_6D_6, 146 MHz): δ -38.05 (m) ppm.

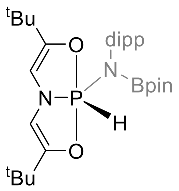

13

Treatment of **3** (30 mg, 0.07 mmol) with a C_6D_6 solution (0.3ml) of HBpin (9 mg, 0.07 mmol) at ambient temperature for 15 min afforded product after removal of solvent (37 mg, 95% yield). ^{1}H NMR (C_6D_6, 500 MHz): δ 9.38 (d, 1H, *J* = 837.7 Hz), 7.27 – 7.25 (m, 1H), 7.08 – 7.04 (m, 2H), 5.49 (d, 2H, *J* = 33.2 Hz), 3.62-3.56 (m, 2H), 1.53 (d, 6H, *J* = 6.6 Hz, 1.45 (d, 6H, *J* = 6.6 Hz, 6H), 1.19 (s, 12H), 1.15 (s, 18H) ppm. ^{13}C NMR (C_6D_6, 126 MHz): δ 150.73 (d, *J* = 6.4 Hz), 147.14, 126.64, 124.52 (d, *J* = 4.4 Hz), 124.02, 123.42, 100.51 (d, *J* = 18.8 Hz), 82.52, 31.69, 28.25, 27.51, 24.66, 23.62, 23.26 ppm. ^{31}P NMR (C_6D_6, 203 MHz): δ -43.02 (dt, *J* = 837.0, 31.5 Hz) ppm. MS (ESI) calc'd for $C_{30}H_{49}BN_2O_4P$ (M-H^+) 543.3518, found 543.3523.

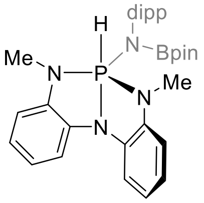

14

Treating **4** (35 mg, 0.08 mmol) with a C_6D_6 solution (0.3ml) of HBpin (10 mg, 0.08 mmol) at ambient temperature for 15 min followed by removal of solvent afforded product (42 mg, 92% yield). ^{1}H NMR (C_6D_6, 500 MHz): δ 7.44 (d, *J* = 7.5 Hz, 2H), 7.10 – 6.85 (m, 8H), 6.56 (d, *J* = 7.4 Hz, 1H), 6.36 (d, *J* = 578.8 Hz, 1H), 3.11 – 2.91 (m, 2H), 2.60 (d, *J* = 16.7 Hz, 6H), 1.15 (d, *J* = 6.9 Hz, 6H), 1.01 (d, *J* = 6.7 Hz, 6H), 0.83 (s, 12H) ppm. ^{13}C NMR (C_6D_6, 126 MHz): δ 147.97 (d, *J* = 5.0 Hz), 135.98, 134.16 (d, *J* = 12.9 Hz), 133.81 (d, *J* = 14.5 Hz), 126.90, 124.52 (d, *J* = 5.7 Hz), 123.11, 119.83, 118.86, 110.92 (d, *J* = 11.4 Hz), 108.55 (d, *J* = 7.2 Hz), 81.95, 28.15, 25.94, 24.61, 23.97, 23.07 ppm. ^{31}P NMR (C_6D_6, MHz): δ -37.57 (d, *J* = 578.8) ppm. MS (ESI) calc'd for $C_{32}H_{43}BN_4O_2P$ (M-H^+) 557.3217, found 557.3212.

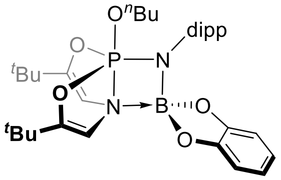

15

Treating **3** (60 mg, 0.14 mmol) with a C_6D_6 solution (0.3ml) of butoxy catecholborane (26 mg, 0.14 mmol) at ambient temperature for 1 hr, followed by removal of solvent, afforded crude solids. Recrystallization from a benzene solution gave the pure product (58 mg, 65% yield). ^{1}H NMR (C_6D_6, 400 MHz): δ 7.15 (d, *J* = 1.5 Hz, 3H), 6.90 (dd, *J* = 5.6, 3.4 Hz, 2H), 6.67 (dd, *J* = 5.6, 3.3 Hz, 2H), 5.33 (d, *J* = 16.6 Hz, 2H), 4.05 – 3.90 (m, 2H), 3.84 – 3.71 (m, 2H), 1.52 (d, *J* = 6.8 Hz, 6H), 1.41 (d, *J* = 7.1 Hz, 6H), 1.24 – 1.10 (m, 4H), 1.04 (s, 9H), 0.87 (t, *J* = 7.4 Hz, 3H) ppm. ^{13}C NMR (C_6D_6, 126 MHz): δ 157.24, 151.97, 147.15 (d, *J* = 6.0 Hz), 134.07 (d, *J* = 8.2 Hz), 126.78, 124.02, 123.38, 119.33, 109.54, 109.33 (d, *J* = 4.7 Hz), 65.24 (d, *J* = 9.2 Hz), 32.56 (d, *J* = 10.1 Hz), 27.98, 26.40, 24.85, 18.46, 15.29, 13.34 ppm. ^{31}P NMR (C_6D_6, 203 MHz): δ -31.41 ppm. MS (ESI) calc'd for $C_{34}H_{50}BN_2O_5P$ (M^+) 608.3550, found 608.3555.

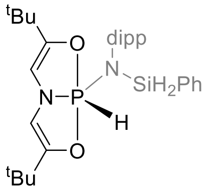

16

Treating **3** (60 mg, 0.14 mmol) with a C_6D_6 solution (0.3ml) of phenylsilane (16 mg, 0.14 mmol) at 50°C for 16 h gave a colorless oil after removal of solvent *in vacuo* (74 mg, 98% yield). ^{1}H NMR (C_6D_6, 500 MHz): δ 9.25 (d, *J* = 801.8 Hz, 1H), 7.85 (dd, *J* = 7.9, 1.7 Hz, 2H), 7.17 – 6.85 (m, 6H), 5.34 (d, *J* = 30.7 Hz, 2H), 5.22 (s, 2H), 3.63 (hept, *J* = 6.8 Hz, 2H), 1.29 (d, *J* = 6.9 Hz, 6H), 1.08 (d, *J* = 6.8 Hz, 6H), 0.85 (s, 18H) ppm. ^{13}C NMR (C_6D_6, 126 MHz): δ 150.26, 146.86, 140.12, 134.80, 129.54, 126.36, 123.82, 101.61 (d, *J* = 18.5 Hz), 31.85, 28.70, 28.22, 27.42 ppm. ^{31}P NMR (C_6D_6, 203 MHz): δ -43.52 (dt, *J* = 801.8, 30.7 Hz) ppm. MS (ESI) calc'd for $C_{30}H_{46}N_2O_2PSi$ ($M+H^+$) 525.3061, found 525.3065.

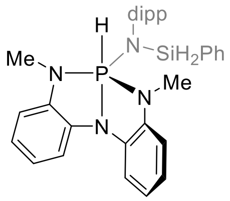

17

Reacting **4** (50 mg, 0.12 mmol) with a C_6D_6 solution (0.3ml) of phenylsilane (13 mg, 0.12 mmol) at 80°C for 48 h afforded yellow oil after solvent removal (62 mg, 99% crude yield). ^{1}H NMR (C_6D_6, 500 MHz): δ 7.32 (d, *J* = 7.1 Hz, 2H), 7.14 – 6.99 (m, 7H), 6.95 (t, *J* = 7.2 Hz, 3H), 6.87 (t, *J* = 7.7 Hz, 2H), 6.73 (t, *J* = 7.8 Hz, 2H), 6.37 (d, *J* = 7.5 Hz, 2H), 5.75 (d, *J* = 534.0 Hz, 1H), 5.05 (d, *J* = 5.8 Hz, 2H), 3.45 – 3.33 (m, 2H), 2.49 (d, *J* = 15.9 Hz, 6H), 1.15 (d, *J* = 6.8 Hz, 6H), 1.06 (d, *J* = 6.7 Hz, 6H) ppm. ^{13}C NMR (C_6D_6, 126 MHz): δ 148.30 (d, *J* = 5.8 Hz), 137.93, 135.77 (d, *J* = 10.4 Hz), 134.65 (d, *J* = 14.5 Hz), 134.25, 133.46, 129.01, 127.16 (d, *J* = 3.6 Hz), 123.90 (d, *J* = 4.0 Hz), 121.42, 119.38, 113.89 (d, *J* = 10.8 Hz), 108.72 (d, *J* = 7.6 Hz), 29.69 (d, *J* = 15.5 Hz), 27.61, 26.24, 23.84 ppm. ^{31}P NMR (C_6D_6, 203 MHz): δ -28.54 (dt, *J* = 534.5, 17.2 Hz) ppm. MS (ESI) calc'd for $C_{32}H_{40}N_4PSi$ (M+H$^+$) 539.2760, found 539.2763.

V. General Synthetic Procedure for Azides

The following procedure was adapted from the literature.[10] A mixture of amine (5 mmol) and $NaNO_2$ (5.5 mmol) were added to a cooled (−30°C) acidic (5 mL of concentrated HCl and 5 mL of distilled H_2O) solution of $NaBF_4$ (10 mmol). A yellow precipitate gradually formed after several minutes of stirring. The mixture was then stirred at −30°C for an additional 30 min. The resulting intermediate diazonium tetrafluoroborate salt was filtered quickly in air and washed with cold water. This sticky, yellow powder was added to a cooled (0°C) aqueous solution (10 mL) of NaN_3. After vigorous gas evolution, the orange mixture was stirred overnight at 25°C. The product was extracted from the aqueous layer with diethyl ether and dried with $MgSO_4$. The solution was filtered, and the solvent was removed to give the crude azide product. The crude product was dissolved in 10 ml of hexane and further purified by passing through a plug of silica gel. Hexane was removed *in vacuo* from the collected solution to afford the pure azide product.

VI. Crystallographic Procedures and Data

Compound **3:**

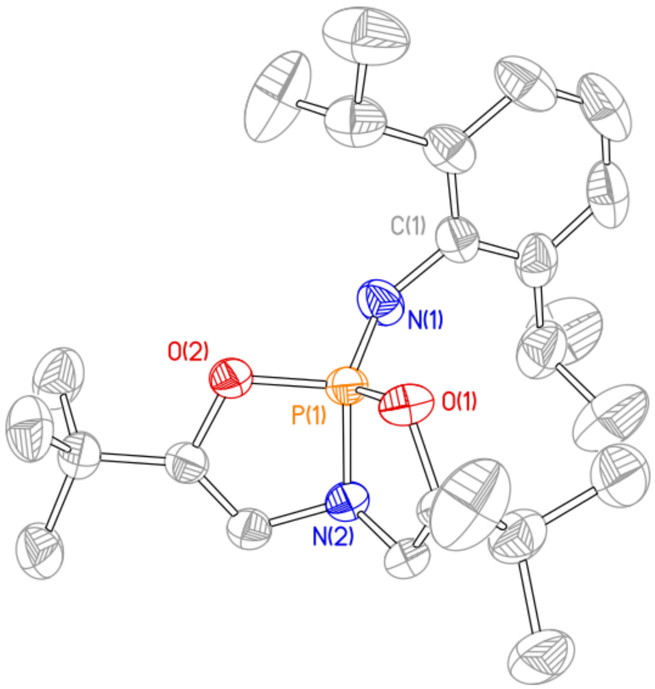

Table S1. Crystal data and structure refinement for **3**.

Identification code	yzl18m	
Empirical formula	C24 H37 N2 O2 P	
Formula weight	416.52	
Temperature	213 K	
Wavelength	0.71073 Å	
Crystal system	Monoclinic	
Space group	C2/c	
Unit cell dimensions	a = 19.931(8) Å	α= 90°.
	b = 12.616(8) Å	β= 109.346(14)°.
	c = 21.565(12) Å	γ = 90°.
Volume	5116(5) Å^3	
Z	8	
Density (calculated)	1.082 Mg/m^3	
Absorption coefficient	0.127 mm^{-1}	
F(000)	1808	
Crystal size	0.200 x 0.150 x 0.120 mm^3	
Theta range for data collection	1.944 to 28.379°.	
Index ranges	-26<=h<=26, -16<=k<=11, -28<=l<=28	
Reflections collected	18786	
Independent reflections	6358 [R(int) = 0.0309]	
Completeness to theta = 25.242°	100.0 %	
Absorption correction	Semi-empirical from equivalents	
Max. and min. transmission	0.9849 and 0.9750	
Refinement method	Full-matrix least-squares on F^2	
Data / restraints / parameters	6358 / 0 / 272	
Goodness-of-fit on F^2	1.033	
Final R indices [I>2sigma(I)]	R1 = 0.0485, wR2 = 0.1339	
R indices (all data)	R1 = 0.0571, wR2 = 0.1426	
Extinction coefficient	n/a	
Largest diff. peak and hole	0.277 and -0.325 e.Å^{-3}	

Table S2. Atomic coordinates (x 10^4) and equivalent isotropic displacement parameters ($Å^2$x 10^3) for **3**. U(eq) is defined as one third of the trace of the orthogonalized U^{ij} tensor.

	x	y	z	U(eq)
C(1)	2822(1)	5049(1)	3630(1)	41(1)
C(2)	2536(1)	5694(1)	4014(1)	47(1)
C(5)	1546(1)	6754(2)	3251(2)	108(1)
C(3)	1748(1)	5932(1)	3794(1)	56(1)
C(4)	1465(1)	6235(2)	4346(1)	92(1)
C(6)	3003(1)	6127(2)	4588(1)	63(1)
C(7)	3721(1)	5949(2)	4778(1)	72(1)
C(8)	3995(1)	5329(2)	4394(1)	66(1)
C(9)	3557(1)	4870(1)	3811(1)	51(1)
C(10)	3865(1)	4196(2)	3388(1)	68(1)
C(12)	4093(2)	3111(2)	3692(2)	112(1)
C(11)	4475(2)	4753(2)	3246(2)	113(1)
C(13)	1513(1)	2715(1)	1738(1)	39(1)
C(14)	1015(1)	3420(1)	1724(1)	37(1)
C(15)	359(1)	3788(1)	1196(1)	44(1)
C(16)	-269(1)	3733(2)	1453(1)	61(1)
C(17)	460(1)	4933(2)	1007(1)	70(1)
C(18)	212(1)	3067(2)	597(1)	69(1)
C(19)	2176(1)	1733(1)	2737(1)	42(1)
C(20)	2067(1)	1857(1)	3305(1)	41(1)
C(21)	2139(1)	1123(1)	3871(1)	54(1)
C(22)	1466(1)	1183(2)	4057(1)	93(1)
C(24)	2271(2)	-7(1)	3667(1)	81(1)
C(23)	2776(1)	1462(2)	4460(1)	74(1)
N(2)	2083(1)	2687(1)	2355(1)	38(1)
N(1)	2363(1)	4649(1)	3035(1)	45(1)
O(1)	1872(1)	2916(1)	3409(1)	41(1)
O(2)	1156(1)	3977(1)	2324(1)	41(1)
P(1)	1920(1)	3663(1)	2823(1)	33(1)

Table S3. Bond lengths [Å] and angles [°] for **3**.

C(1)-N(1)	1.3988(18)
C(1)-C(9)	1.404(2)
C(1)-C(2)	1.409(2)
C(2)-C(6)	1.391(2)
C(2)-C(3)	1.512(2)
C(5)-C(3)	1.516(3)
C(5)-H(5A)	0.9600
C(5)-H(5B)	0.9600
C(5)-H(5C)	0.9600
C(3)-C(4)	1.525(3)
C(3)-H(3)	0.9800
C(4)-H(4A)	0.9600
C(4)-H(4B)	0.9600
C(4)-H(4C)	0.9600
C(6)-C(7)	1.371(3)
C(6)-H(6)	0.9300
C(7)-C(8)	1.375(3)
C(7)-H(7)	0.9300
C(8)-C(9)	1.397(2)
C(8)-H(8)	0.9300
C(9)-C(10)	1.518(3)
C(10)-C(11)	1.520(3)
C(10)-C(12)	1.521(3)
C(10)-H(10)	0.9800
C(12)-H(12A)	0.9600
C(12)-H(12B)	0.9600
C(12)-H(12C)	0.9600
C(11)-H(11A)	0.9600
C(11)-H(11B)	0.9600
C(11)-H(11C)	0.9600
C(13)-C(14)	1.3259(19)
C(13)-N(2)	1.4352(18)
C(13)-H(13)	0.9300
C(14)-O(2)	1.4157(16)

C(14)-C(15)	1.4950(19)
C(15)-C(18)	1.527(2)
C(15)-C(16)	1.528(2)
C(15)-C(17)	1.533(2)
C(16)-H(16A)	0.9600
C(16)-H(16B)	0.9600
C(16)-H(16C)	0.9600
C(17)-H(17A)	0.9600
C(17)-H(17B)	0.9600
C(17)-H(17C)	0.9600
C(18)-H(18A)	0.9600
C(18)-H(18B)	0.9600
C(18)-H(18C)	0.9600
C(19)-C(20)	1.321(2)
C(19)-N(2)	1.4370(19)
C(19)-H(19)	0.9300
C(20)-O(1)	1.4294(17)
C(20)-C(21)	1.500(2)
C(21)-C(22)	1.525(3)
C(21)-C(23)	1.530(3)
C(21)-C(24)	1.539(3)
C(22)-H(22A)	0.9600
C(22)-H(22B)	0.9600
C(22)-H(22C)	0.9600
C(24)-H(24A)	0.9600
C(24)-H(24B)	0.9600
C(24)-H(24C)	0.9600
C(23)-H(23A)	0.9600
C(23)-H(23B)	0.9600
C(23)-H(23C)	0.9600
N(2)-P(1)	1.6903(13)
N(1)-P(1)	1.5069(14)
O(1)-P(1)	1.6044(12)
O(2)-P(1)	1.5962(11)
N(1)-C(1)-C(9)	120.12(14)
N(1)-C(1)-C(2)	118.60(13)

C(9)-C(1)-C(2)	121.12(13)
C(6)-C(2)-C(1)	117.96(16)
C(6)-C(2)-C(3)	121.31(16)
C(1)-C(2)-C(3)	120.70(13)
C(3)-C(5)-H(5A)	109.5
C(3)-C(5)-H(5B)	109.5
H(5A)-C(5)-H(5B)	109.5
C(3)-C(5)-H(5C)	109.5
H(5A)-C(5)-H(5C)	109.5
H(5B)-C(5)-H(5C)	109.5
C(2)-C(3)-C(5)	111.17(14)
C(2)-C(3)-C(4)	114.84(17)
C(5)-C(3)-C(4)	111.0(2)
C(2)-C(3)-H(3)	106.4
C(5)-C(3)-H(3)	106.4
C(4)-C(3)-H(3)	106.4
C(3)-C(4)-H(4A)	109.5
C(3)-C(4)-H(4B)	109.5
H(4A)-C(4)-H(4B)	109.5
C(3)-C(4)-H(4C)	109.5
H(4A)-C(4)-H(4C)	109.5
H(4B)-C(4)-H(4C)	109.5
C(7)-C(6)-C(2)	121.70(19)
C(7)-C(6)-H(6)	119.1
C(2)-C(6)-H(6)	119.1
C(6)-C(7)-C(8)	119.81(16)
C(6)-C(7)-H(7)	120.1
C(8)-C(7)-H(7)	120.1
C(7)-C(8)-C(9)	121.54(17)
C(7)-C(8)-H(8)	119.2
C(9)-C(8)-H(8)	119.2
C(8)-C(9)-C(1)	117.84(17)
C(8)-C(9)-C(10)	121.14(17)
C(1)-C(9)-C(10)	121.02(14)
C(9)-C(10)-C(11)	111.85(19)
C(9)-C(10)-C(12)	111.56(19)

C(11)-C(10)-C(12)	111.0(2)
C(9)-C(10)-H(10)	107.4
C(11)-C(10)-H(10)	107.4
C(12)-C(10)-H(10)	107.4
C(10)-C(12)-H(12A)	109.5
C(10)-C(12)-H(12B)	109.5
H(12A)-C(12)-H(12B)	109.5
C(10)-C(12)-H(12C)	109.5
H(12A)-C(12)-H(12C)	109.5
H(12B)-C(12)-H(12C)	109.5
C(10)-C(11)-H(11A)	109.5
C(10)-C(11)-H(11B)	109.5
H(11A)-C(11)-H(11B)	109.5
C(10)-C(11)-H(11C)	109.5
H(11A)-C(11)-H(11C)	109.5
H(11B)-C(11)-H(11C)	109.5
C(14)-C(13)-N(2)	113.23(11)
C(14)-C(13)-H(13)	123.4
N(2)-C(13)-H(13)	123.4
C(13)-C(14)-O(2)	113.07(12)
C(13)-C(14)-C(15)	133.22(12)
O(2)-C(14)-C(15)	113.61(11)
C(14)-C(15)-C(18)	109.51(12)
C(14)-C(15)-C(16)	108.90(13)
C(18)-C(15)-C(16)	109.24(14)
C(14)-C(15)-C(17)	109.62(13)
C(18)-C(15)-C(17)	109.92(15)
C(16)-C(15)-C(17)	109.63(14)
C(15)-C(16)-H(16A)	109.5
C(15)-C(16)-H(16B)	109.5
H(16A)-C(16)-H(16B)	109.5
C(15)-C(16)-H(16C)	109.5
H(16A)-C(16)-H(16C)	109.5
H(16B)-C(16)-H(16C)	109.5
C(15)-C(17)-H(17A)	109.5
C(15)-C(17)-H(17B)	109.5

H(17A)-C(17)-H(17B)	109.5
C(15)-C(17)-H(17C)	109.5
H(17A)-C(17)-H(17C)	109.5
H(17B)-C(17)-H(17C)	109.5
C(15)-C(18)-H(18A)	109.5
C(15)-C(18)-H(18B)	109.5
H(18A)-C(18)-H(18B)	109.5
C(15)-C(18)-H(18C)	109.5
H(18A)-C(18)-H(18C)	109.5
H(18B)-C(18)-H(18C)	109.5
C(20)-C(19)-N(2)	113.80(12)
C(20)-C(19)-H(19)	123.1
N(2)-C(19)-H(19)	123.1
C(19)-C(20)-O(1)	113.10(12)
C(19)-C(20)-C(21)	132.78(13)
O(1)-C(20)-C(21)	114.05(12)
C(20)-C(21)-C(22)	109.15(14)
C(20)-C(21)-C(23)	109.43(15)
C(22)-C(21)-C(23)	109.09(18)
C(20)-C(21)-C(24)	108.60(14)
C(22)-C(21)-C(24)	111.69(18)
C(23)-C(21)-C(24)	108.85(16)
C(21)-C(22)-H(22A)	109.5
C(21)-C(22)-H(22B)	109.5
H(22A)-C(22)-H(22B)	109.5
C(21)-C(22)-H(22C)	109.5
H(22A)-C(22)-H(22C)	109.5
H(22B)-C(22)-H(22C)	109.5
C(21)-C(24)-H(24A)	109.5
C(21)-C(24)-H(24B)	109.5
H(24A)-C(24)-H(24B)	109.5
C(21)-C(24)-H(24C)	109.5
H(24A)-C(24)-H(24C)	109.5
H(24B)-C(24)-H(24C)	109.5
C(21)-C(23)-H(23A)	109.5
C(21)-C(23)-H(23B)	109.5

H(23A)-C(23)-H(23B)	109.5
C(21)-C(23)-H(23C)	109.5
H(23A)-C(23)-H(23C)	109.5
H(23B)-C(23)-H(23C)	109.5
C(13)-N(2)-C(19)	117.69(11)
C(13)-N(2)-P(1)	106.58(9)
C(19)-N(2)-P(1)	106.29(9)
C(1)-N(1)-P(1)	135.72(10)
C(20)-O(1)-P(1)	109.60(8)
C(14)-O(2)-P(1)	110.41(8)
N(1)-P(1)-O(2)	109.40(7)
N(1)-P(1)-O(1)	115.30(7)
O(2)-P(1)-O(1)	112.62(6)
N(1)-P(1)-N(2)	124.96(7)
O(2)-P(1)-N(2)	96.19(6)
O(1)-P(1)-N(2)	96.68(7)

Symmetry transformations used to generate equivalent atoms:

Table S4. Anisotropic displacement parameters ($Å^2$x 10^3) for **3**. The anisotropic displacement factor exponent takes the form: $-2\pi^2$[h^2 $a^{*2}U^{11}$ + ... + 2 h k a* b* U^{12}]

	U^{11}	U^{22}	U^{33}	U^{23}	U^{13}	U^{12}
C(1)	46(1)	32(1)	39(1)	5(1)	7(1)	-11(1)
C(2)	56(1)	40(1)	45(1)	-1(1)	15(1)	-18(1)
C(5)	70(1)	107(2)	157(3)	64(2)	51(2)	28(1)
C(3)	59(1)	48(1)	68(1)	-6(1)	29(1)	-12(1)
C(4)	90(2)	92(2)	112(2)	-43(1)	57(2)	-24(1)
C(6)	81(1)	59(1)	47(1)	-11(1)	21(1)	-31(1)
C(7)	79(1)	80(1)	43(1)	2(1)	0(1)	-39(1)
C(8)	50(1)	73(1)	56(1)	20(1)	-5(1)	-16(1)
C(9)	47(1)	46(1)	52(1)	14(1)	5(1)	-5(1)
C(10)	49(1)	66(1)	84(1)	9(1)	16(1)	8(1)
C(12)	114(2)	64(1)	165(3)	23(2)	55(2)	21(1)
C(11)	106(2)	102(2)	159(3)	12(2)	83(2)	0(2)
C(13)	43(1)	39(1)	36(1)	-4(1)	14(1)	3(1)
C(14)	37(1)	36(1)	38(1)	-4(1)	13(1)	-2(1)
C(15)	39(1)	46(1)	43(1)	1(1)	10(1)	4(1)
C(16)	36(1)	83(1)	62(1)	2(1)	12(1)	2(1)
C(17)	64(1)	58(1)	82(1)	22(1)	14(1)	7(1)
C(18)	66(1)	82(1)	46(1)	-12(1)	0(1)	17(1)
C(19)	45(1)	32(1)	45(1)	-3(1)	11(1)	8(1)
C(20)	43(1)	30(1)	49(1)	-2(1)	14(1)	2(1)
C(21)	67(1)	41(1)	56(1)	9(1)	23(1)	8(1)
C(22)	89(2)	87(2)	120(2)	51(1)	57(2)	11(1)
C(24)	124(2)	39(1)	68(1)	10(1)	16(1)	14(1)
C(23)	94(2)	75(1)	47(1)	1(1)	16(1)	17(1)
N(2)	38(1)	36(1)	40(1)	-4(1)	14(1)	5(1)
N(1)	50(1)	38(1)	42(1)	-1(1)	9(1)	-11(1)
O(1)	51(1)	31(1)	47(1)	-2(1)	25(1)	1(1)
O(2)	34(1)	41(1)	45(1)	-12(1)	10(1)	5(1)
P(1)	32(1)	29(1)	38(1)	-4(1)	12(1)	0(1)

Table S5. Hydrogen coordinates (x 10^4) and isotropic displacement parameters ($Å^2$x 10^{3}) for **3**.

	x	y	z	U(eq)
H(5A)	1799	7400	3408	162
H(5B)	1043	6886	3119	162
H(5C)	1665	6496	2882	162
H(3)	1505	5276	3602	68
H(4A)	1695	6872	4555	138
H(4B)	1561	5672	4663	138
H(4C)	961	6351	4167	138
H(6)	2823	6549	4850	75
H(7)	4022	6247	5164	87
H(8)	4483	5212	4527	79
H(10)	3487	4085	2966	81
H(12A)	4453	3194	4114	168
H(12B)	4281	2703	3410	168
H(12C)	3690	2751	3742	168
H(11A)	4319	5434	3053	169
H(11B)	4629	4332	2947	169
H(11C)	4864	4845	3648	169
H(13)	1495	2283	1384	47
H(16A)	-306	3029	1608	92
H(16B)	-699	3908	1106	92
H(16C)	-196	4227	1808	92
H(17A)	554	5381	1386	106
H(17B)	37	5170	671	106
H(17C)	854	4965	845	106
H(18A)	609	3088	439	104
H(18B)	-208	3306	257	104
H(18C)	142	2353	718	104
H(19)	2301	1087	2598	50
H(22A)	1387	1902	4161	139
H(22B)	1518	741	4432	139

H(22C)	1067	943	3694	139
H(24A)	1868	-235	3306	122
H(24B)	2341	-479	4032	122
H(24C)	2687	-10	3536	122
H(23A)	3208	1334	4368	111
H(23B)	2780	1061	4840	111
H(23C)	2738	2203	4543	111

Table S6. Torsion angles [°] for **3**.

N(1)-C(1)-C(2)-C(6)	-177.03(13)
C(9)-C(1)-C(2)-C(6)	-1.7(2)
N(1)-C(1)-C(2)-C(3)	1.15(19)
C(9)-C(1)-C(2)-C(3)	176.52(13)
C(6)-C(2)-C(3)-C(5)	101.6(2)
C(1)-C(2)-C(3)-C(5)	-76.5(2)
C(6)-C(2)-C(3)-C(4)	-25.5(2)
C(1)-C(2)-C(3)-C(4)	156.40(16)
C(1)-C(2)-C(6)-C(7)	0.7(2)
C(3)-C(2)-C(6)-C(7)	-177.45(16)
C(2)-C(6)-C(7)-C(8)	0.1(3)
C(6)-C(7)-C(8)-C(9)	0.0(3)
C(7)-C(8)-C(9)-C(1)	-0.9(2)
C(7)-C(8)-C(9)-C(10)	179.32(16)
N(1)-C(1)-C(9)-C(8)	177.06(13)
C(2)-C(1)-C(9)-C(8)	1.8(2)
N(1)-C(1)-C(9)-C(10)	-3.2(2)
C(2)-C(1)-C(9)-C(10)	-178.50(14)
C(8)-C(9)-C(10)-C(11)	-50.5(2)
C(1)-C(9)-C(10)-C(11)	129.7(2)
C(8)-C(9)-C(10)-C(12)	74.4(2)
C(1)-C(9)-C(10)-C(12)	-105.3(2)
N(2)-C(13)-C(14)-O(2)	-0.56(16)
N(2)-C(13)-C(14)-C(15)	175.37(14)

C(13)-C(14)-C(15)-C(18)	9.9(2)
O(2)-C(14)-C(15)-C(18)	-174.19(13)
C(13)-C(14)-C(15)-C(16)	129.29(17)
O(2)-C(14)-C(15)-C(16)	-54.80(16)
C(13)-C(14)-C(15)-C(17)	-110.77(19)
O(2)-C(14)-C(15)-C(17)	65.14(16)
N(2)-C(19)-C(20)-O(1)	0.77(17)
N(2)-C(19)-C(20)-C(21)	-176.14(15)
C(19)-C(20)-C(21)-C(22)	-131.1(2)
O(1)-C(20)-C(21)-C(22)	52.0(2)
C(19)-C(20)-C(21)-C(23)	109.6(2)
O(1)-C(20)-C(21)-C(23)	-67.27(17)
C(19)-C(20)-C(21)-C(24)	-9.1(3)
O(1)-C(20)-C(21)-C(24)	174.03(15)
C(14)-C(13)-N(2)-C(19)	115.04(14)
C(14)-C(13)-N(2)-P(1)	-4.10(14)
C(20)-C(19)-N(2)-C(13)	-115.26(14)
C(20)-C(19)-N(2)-P(1)	4.03(14)
C(9)-C(1)-N(1)-P(1)	94.35(18)
C(2)-C(1)-N(1)-P(1)	-90.23(18)
C(19)-C(20)-O(1)-P(1)	-5.56(14)
C(21)-C(20)-O(1)-P(1)	171.96(11)
C(13)-C(14)-O(2)-P(1)	5.34(14)
C(15)-C(14)-O(2)-P(1)	-171.42(9)
C(1)-N(1)-P(1)-O(2)	136.51(15)
C(1)-N(1)-P(1)-O(1)	8.37(18)
C(1)-N(1)-P(1)-N(2)	-110.80(16)
C(14)-O(2)-P(1)-N(1)	123.74(9)
C(14)-O(2)-P(1)-O(1)	-106.64(10)
C(14)-O(2)-P(1)-N(2)	-6.74(9)
C(20)-O(1)-P(1)-N(1)	-127.00(10)
C(20)-O(1)-P(1)-O(2)	106.48(9)
C(20)-O(1)-P(1)-N(2)	6.90(9)
C(13)-N(2)-P(1)-N(1)	-112.66(10)
C(19)-N(2)-P(1)-N(1)	121.03(10)
C(13)-N(2)-P(1)-O(2)	6.26(9)

C(19)-N(2)-P(1)-O(2)	-120.06(9)
C(13)-N(2)-P(1)-O(1)	119.97(9)
C(19)-N(2)-P(1)-O(1)	-6.34(9)

Symmetry transformations used to generate equivalent atoms:

Compound **4**:

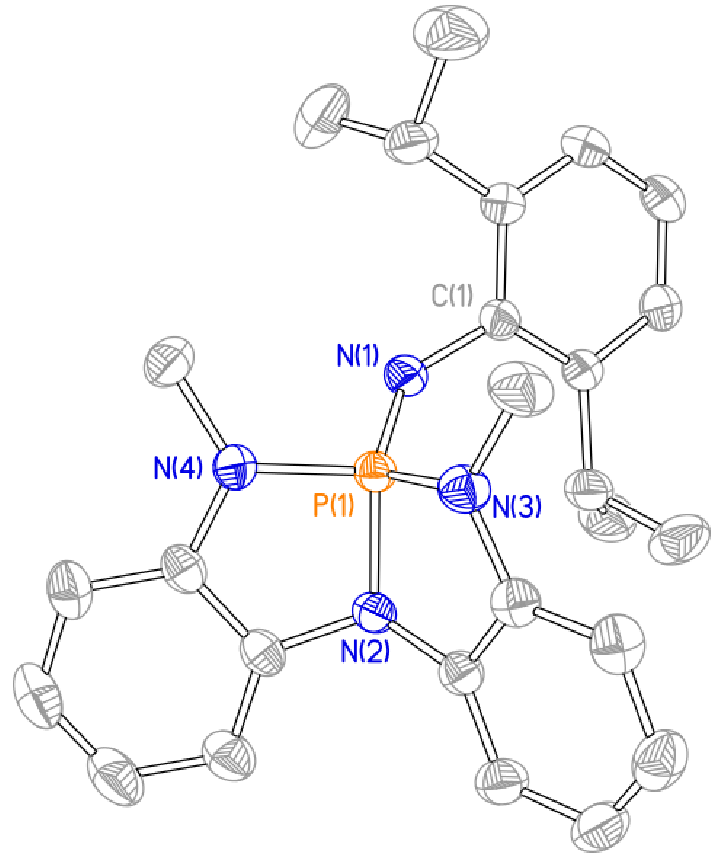

Table S7. Crystal data and structure refinement for **4**.

Identification code	yzl19s	
Empirical formula	C26 H31 N4 P	
Formula weight	430.52	
Temperature	223(2) K	
Wavelength	0.71073 Å	
Crystal system	Monoclinic	
Space group	$P2_1/n$	
Unit cell dimensions	a = 12.8565(18) Å	α= 90°.
	b = 9.4531(13) Å	β= 96.382(4)°.
	c = 19.152(3) Å	γ = 90°.
Volume	2313.2(6) $Å^3$	
Z	4	
Density (calculated)	1.236 Mg/m^3	
Absorption coefficient	0.140 mm^{-1}	
F(000)	920	
Crystal size	0.220 x 0.200 x 0.160 mm^3	
Theta range for data collection	2.016 to 28.298°.	
Index ranges	-11<=h<=17, -12<=k<=12, -25<=l<=21	
Reflections collected	15190	
Independent reflections	5662 [R(int) = 0.0172]	
Completeness to theta = 25.242°	99.6 %	
Refinement method	Full-matrix least-squares on F^2	
Data / restraints / parameters	5662 / 0 / 286	
Goodness-of-fit on F^2	1.035	
Final R indices [I>2sigma(I)]	R1 = 0.0432, wR2 = 0.1188	
R indices (all data)	R1 = 0.0489, wR2 = 0.1248	
Extinction coefficient	n/a	
Largest diff. peak and hole	0.291 and -0.355 e.$Å^{-3}$	

Table S8. Atomic coordinates (x 10^4) and equivalent isotropic displacement parameters (Å^{2}x 10^3) for **4**. U(eq) is defined as one third of the trace of the orthogonalized U^{ij} tensor.

	x	y	z	U(eq)
C(1)	6467(1)	8158(1)	1255(1)	27(1)
C(2)	5609(1)	9024(1)	1386(1)	32(1)
C(3)	4596(1)	8533(2)	1218(1)	38(1)
C(4)	4402(1)	7220(2)	917(1)	41(1)
C(5)	5236(1)	6394(2)	770(1)	39(1)
C(6)	6272(1)	6834(1)	930(1)	32(1)
C(7)	7142(1)	5882(2)	728(1)	43(1)
C(8)	7022(1)	4344(2)	944(1)	58(1)
C(9)	7194(1)	5971(2)	-69(1)	59(1)
C(10)	5796(1)	10458(2)	1729(1)	43(1)
C(11)	4989(2)	11573(2)	1460(1)	66(1)
C(12)	5870(2)	10311(2)	2523(1)	71(1)
C(13)	7488(1)	10474(2)	102(1)	46(1)
C(14)	8901(1)	8741(1)	-48(1)	30(1)
C(15)	8787(1)	8725(2)	-773(1)	38(1)
C(16)	9358(1)	7738(2)	-1112(1)	44(1)
C(17)	10032(1)	6806(2)	-740(1)	45(1)
C(18)	10145(1)	6815(2)	-7(1)	37(1)
C(19)	9575(1)	7779(1)	333(1)	28(1)
C(20)	10398(1)	8154(1)	1563(1)	30(1)
C(21)	11337(1)	7434(2)	1643(1)	41(1)
C(22)	12104(1)	7863(2)	2173(1)	53(1)
C(23)	11918(1)	8959(2)	2615(1)	52(1)
C(24)	10969(1)	9683(2)	2542(1)	41(1)
C(25)	10216(1)	9288(1)	2006(1)	31(1)
C(26)	8823(1)	10981(2)	2269(1)	46(1)
N(1)	7473(1)	8610(1)	1518(1)	30(1)
N(2)	9512(1)	7904(1)	1065(1)	27(1)
N(3)	8350(1)	9574(1)	393(1)	33(1)
N(4)	9226(1)	9888(1)	1836(1)	32(1)
P(1)	8496(1)	8983(1)	1221(1)	26(1)

Table S9. Bond lengths [Å] and angles [°] for **4**.

C(1)-N(1)	1.4021(16)
C(1)-C(6)	1.4076(17)
C(1)-C(2)	1.4189(17)
C(2)-C(3)	1.3870(19)
C(2)-C(10)	1.5133(19)
C(3)-C(4)	1.380(2)
C(3)-H(3)	0.9400
C(4)-C(5)	1.380(2)
C(4)-H(4)	0.9400
C(5)-C(6)	1.3972(18)
C(5)-H(5)	0.9400
C(6)-C(7)	1.5181(18)
C(7)-C(8)	1.524(2)
C(7)-C(9)	1.536(3)
C(7)-H(7)	0.9900
C(8)-H(8A)	0.9700
C(8)-H(8B)	0.9700
C(8)-H(8C)	0.9700
C(9)-H(9A)	0.9700
C(9)-H(9B)	0.9700
C(9)-H(9C)	0.9700
C(10)-C(12)	1.519(3)
C(10)-C(11)	1.527(2)
C(10)-H(10)	0.9900
C(11)-H(11A)	0.9700
C(11)-H(11B)	0.9700
C(11)-H(11C)	0.9700
C(12)-H(12A)	0.9700
C(12)-H(12B)	0.9700
C(12)-H(12C)	0.9700
C(13)-N(3)	1.4584(17)
C(13)-H(13A)	0.9700
C(13)-H(13B)	0.9700
C(13)-H(13C)	0.9700

C(14)-C(15)	1.3809(19)
C(14)-N(3)	1.4016(16)
C(14)-C(19)	1.4037(18)
C(15)-C(16)	1.392(2)
C(15)-H(15)	0.9400
C(16)-C(17)	1.377(2)
C(16)-H(16)	0.9400
C(17)-C(18)	1.395(2)
C(17)-H(17)	0.9400
C(18)-C(19)	1.3769(18)
C(18)-H(18)	0.9400
C(19)-N(2)	1.4187(16)
C(20)-C(21)	1.3790(19)
C(20)-C(25)	1.4019(18)
C(20)-N(2)	1.4222(16)
C(21)-C(22)	1.395(2)
C(21)-H(21)	0.9400
C(22)-C(23)	1.375(3)
C(22)-H(22)	0.9400
C(23)-C(24)	1.392(2)
C(23)-H(23)	0.9400
C(24)-C(25)	1.3815(19)
C(24)-H(24)	0.9400
C(25)-N(4)	1.3997(17)
C(26)-N(4)	1.4555(17)
C(26)-H(26A)	0.9700
C(26)-H(26B)	0.9700
C(26)-H(26C)	0.9700
N(1)-P(1)	1.5309(11)
N(2)-P(1)	1.7095(10)
N(3)-P(1)	1.6723(12)
N(4)-P(1)	1.6591(11)
N(1)-C(1)-C(6)	122.79(11)
N(1)-C(1)-C(2)	117.65(11)
C(6)-C(1)-C(2)	119.27(11)
C(3)-C(2)-C(1)	119.55(12)

C(3)-C(2)-C(10)	120.03(12)
C(1)-C(2)-C(10)	120.39(12)
C(4)-C(3)-C(2)	121.36(13)
C(4)-C(3)-H(3)	119.3
C(2)-C(3)-H(3)	119.3
C(5)-C(4)-C(3)	119.03(13)
C(5)-C(4)-H(4)	120.5
C(3)-C(4)-H(4)	120.5
C(4)-C(5)-C(6)	122.10(13)
C(4)-C(5)-H(5)	119.0
C(6)-C(5)-H(5)	119.0
C(5)-C(6)-C(1)	118.63(12)
C(5)-C(6)-C(7)	118.66(12)
C(1)-C(6)-C(7)	122.68(11)
C(6)-C(7)-C(8)	113.38(13)
C(6)-C(7)-C(9)	109.60(14)
C(8)-C(7)-C(9)	109.78(14)
C(6)-C(7)-H(7)	108.0
C(8)-C(7)-H(7)	108.0
C(9)-C(7)-H(7)	108.0
C(7)-C(8)-H(8A)	109.5
C(7)-C(8)-H(8B)	109.5
H(8A)-C(8)-H(8B)	109.5
C(7)-C(8)-H(8C)	109.5
H(8A)-C(8)-H(8C)	109.5
H(8B)-C(8)-H(8C)	109.5
C(7)-C(9)-H(9A)	109.5
C(7)-C(9)-H(9B)	109.5
H(9A)-C(9)-H(9B)	109.5
C(7)-C(9)-H(9C)	109.5
H(9A)-C(9)-H(9C)	109.5
H(9B)-C(9)-H(9C)	109.5
C(2)-C(10)-C(12)	109.97(14)
C(2)-C(10)-C(11)	113.77(14)
C(12)-C(10)-C(11)	111.42(15)
C(2)-C(10)-H(10)	107.1

C(12)-C(10)-H(10)	107.1
C(11)-C(10)-H(10)	107.1
C(10)-C(11)-H(11A)	109.5
C(10)-C(11)-H(11B)	109.5
H(11A)-C(11)-H(11B)	109.5
C(10)-C(11)-H(11C)	109.5
H(11A)-C(11)-H(11C)	109.5
H(11B)-C(11)-H(11C)	109.5
C(10)-C(12)-H(12A)	109.5
C(10)-C(12)-H(12B)	109.5
H(12A)-C(12)-H(12B)	109.5
C(10)-C(12)-H(12C)	109.5
H(12A)-C(12)-H(12C)	109.5
H(12B)-C(12)-H(12C)	109.5
N(3)-C(13)-H(13A)	109.5
N(3)-C(13)-H(13B)	109.5
H(13A)-C(13)-H(13B)	109.5
N(3)-C(13)-H(13C)	109.5
H(13A)-C(13)-H(13C)	109.5
H(13B)-C(13)-H(13C)	109.5
C(15)-C(14)-N(3)	127.43(13)
C(15)-C(14)-C(19)	120.36(12)
N(3)-C(14)-C(19)	112.06(11)
C(14)-C(15)-C(16)	118.32(14)
C(14)-C(15)-H(15)	120.8
C(16)-C(15)-H(15)	120.8
C(17)-C(16)-C(15)	121.44(13)
C(17)-C(16)-H(16)	119.3
C(15)-C(16)-H(16)	119.3
C(16)-C(17)-C(18)	120.38(14)
C(16)-C(17)-H(17)	119.8
C(18)-C(17)-H(17)	119.8
C(19)-C(18)-C(17)	118.59(14)
C(19)-C(18)-H(18)	120.7
C(17)-C(18)-H(18)	120.7
C(18)-C(19)-C(14)	120.91(12)

C(18)-C(19)-N(2)	127.90(12)
C(14)-C(19)-N(2)	111.06(11)
C(21)-C(20)-C(25)	120.89(12)
C(21)-C(20)-N(2)	128.12(13)
C(25)-C(20)-N(2)	110.98(11)
C(20)-C(21)-C(22)	118.43(15)
C(20)-C(21)-H(21)	120.8
C(22)-C(21)-H(21)	120.8
C(23)-C(22)-C(21)	120.59(15)
C(23)-C(22)-H(22)	119.7
C(21)-C(22)-H(22)	119.7
C(22)-C(23)-C(24)	121.32(15)
C(22)-C(23)-H(23)	119.3
C(24)-C(23)-H(23)	119.3
C(25)-C(24)-C(23)	118.36(15)
C(25)-C(24)-H(24)	120.8
C(23)-C(24)-H(24)	120.8
C(24)-C(25)-N(4)	127.42(13)
C(24)-C(25)-C(20)	120.37(13)
N(4)-C(25)-C(20)	112.20(11)
N(4)-C(26)-H(26A)	109.5
N(4)-C(26)-H(26B)	109.5
H(26A)-C(26)-H(26B)	109.5
N(4)-C(26)-H(26C)	109.5
H(26A)-C(26)-H(26C)	109.5
H(26B)-C(26)-H(26C)	109.5
C(1)-N(1)-P(1)	137.19(10)
C(19)-N(2)-C(20)	123.23(10)
C(19)-N(2)-P(1)	110.55(8)
C(20)-N(2)-P(1)	110.72(8)
C(14)-N(3)-C(13)	120.73(12)
C(14)-N(3)-P(1)	111.86(9)
C(13)-N(3)-P(1)	123.92(10)
C(25)-N(4)-C(26)	121.82(12)
C(25)-N(4)-P(1)	112.56(9)
C(26)-N(4)-P(1)	124.19(10)

N(1)-P(1)-N(4)	107.24(6)
N(1)-P(1)-N(3)	114.72(6)
N(4)-P(1)-N(3)	119.73(6)
N(1)-P(1)-N(2)	129.22(6)
N(4)-P(1)-N(2)	92.84(5)
N(3)-P(1)-N(2)	92.35(5)

Symmetry transformations used to generate equivalent atoms:

Table S10. Anisotropic displacement parameters ($Å^2$x 10^3) for **4**. The anisotropic displacement factor exponent takes the form: $-2\pi^2$[h^2 $a^{*2}U^{11}$ + ... + 2 h k a* b* U^{12}]

	U^{11}	U^{22}	U^{33}	U^{23}	U^{13}	U^{12}
C(1)	24(1)	31(1)	27(1)	-1(1)	3(1)	-1(1)
C(2)	28(1)	34(1)	33(1)	-3(1)	4(1)	2(1)
C(3)	26(1)	45(1)	42(1)	-3(1)	4(1)	5(1)
C(4)	24(1)	50(1)	48(1)	-7(1)	-2(1)	-3(1)
C(5)	29(1)	43(1)	45(1)	-13(1)	0(1)	-5(1)
C(6)	26(1)	35(1)	35(1)	-7(1)	2(1)	0(1)
C(7)	26(1)	38(1)	64(1)	-22(1)	5(1)	-3(1)
C(8)	45(1)	41(1)	87(1)	-15(1)	0(1)	6(1)
C(9)	45(1)	62(1)	74(1)	-33(1)	27(1)	-18(1)
C(10)	35(1)	37(1)	57(1)	-13(1)	9(1)	4(1)
C(11)	59(1)	39(1)	100(2)	-7(1)	8(1)	12(1)
C(12)	86(2)	69(1)	56(1)	-29(1)	0(1)	9(1)
C(13)	45(1)	44(1)	49(1)	10(1)	1(1)	16(1)
C(14)	30(1)	28(1)	34(1)	2(1)	6(1)	-4(1)
C(15)	42(1)	40(1)	34(1)	7(1)	5(1)	-6(1)
C(16)	50(1)	52(1)	32(1)	-3(1)	12(1)	-11(1)
C(17)	45(1)	48(1)	44(1)	-12(1)	17(1)	-3(1)
C(18)	34(1)	35(1)	43(1)	-5(1)	8(1)	2(1)
C(19)	26(1)	28(1)	31(1)	-1(1)	5(1)	-4(1)
C(20)	26(1)	29(1)	33(1)	5(1)	1(1)	-3(1)
C(21)	33(1)	40(1)	49(1)	3(1)	0(1)	5(1)
C(22)	32(1)	55(1)	67(1)	4(1)	-11(1)	5(1)
C(23)	38(1)	58(1)	56(1)	2(1)	-14(1)	-7(1)
C(24)	38(1)	43(1)	41(1)	-2(1)	-1(1)	-11(1)
C(25)	26(1)	32(1)	34(1)	4(1)	2(1)	-6(1)
C(26)	39(1)	48(1)	52(1)	-23(1)	12(1)	-6(1)
N(1)	24(1)	32(1)	36(1)	-5(1)	4(1)	-2(1)
N(2)	23(1)	27(1)	31(1)	0(1)	3(1)	1(1)
N(3)	33(1)	29(1)	35(1)	4(1)	2(1)	6(1)
N(4)	27(1)	32(1)	36(1)	-8(1)	4(1)	-4(1)
P(1)	23(1)	24(1)	31(1)	-2(1)	3(1)	-1(1)

Table S11. Hydrogen coordinates (x 10^4) and isotropic displacement parameters ($Å^2$x 10 ^{3}) for **4**.

	x	y	z	U(eq)
H(3)	4030	9105	1311	45
H(4)	3712	6893	812	49
H(5)	5102	5508	556	47
H(7)	7813	6238	967	52
H(8A)	7010	4290	1448	87
H(8B)	7606	3795	810	87
H(8C)	6373	3966	709	87
H(9A)	6561	5573	-314	88
H(9B)	7796	5443	-190	88
H(9C)	7260	6953	-204	88
H(10)	6485	10795	1614	51
H(11A)	4921	11592	951	99
H(11B)	5216	12494	1641	99
H(11C)	4318	11342	1618	99
H(12A)	5195	10029	2658	106
H(12B)	6073	11211	2740	106
H(12C)	6389	9600	2677	106
H(13A)	7746	11171	-209	69
H(13B)	7190	10953	481	69
H(13C)	6955	9899	-160	69
H(15)	8335	9364	-1032	46
H(16)	9281	7706	-1606	53
H(17)	10419	6161	-981	54
H(18)	10600	6176	249	45
H(21)	11457	6673	1347	49
H(22)	12755	7399	2229	63
H(23)	12441	9224	2973	63
H(24)	10844	10423	2849	49
H(26A)	8769	10608	2735	69
H(26B)	8136	11278	2059	69
H(26C)	9294	11785	2301	69

Table S12. Torsion angles [°] for **4**.

N(1)-C(1)-C(2)-C(3)	-171.58(12)
C(6)-C(1)-C(2)-C(3)	2.43(19)
N(1)-C(1)-C(2)-C(10)	6.53(18)
C(6)-C(1)-C(2)-C(10)	-179.46(13)
C(1)-C(2)-C(3)-C(4)	-0.7(2)
C(10)-C(2)-C(3)-C(4)	-178.85(14)
C(2)-C(3)-C(4)-C(5)	-1.1(2)
C(3)-C(4)-C(5)-C(6)	1.2(2)
C(4)-C(5)-C(6)-C(1)	0.5(2)
C(4)-C(5)-C(6)-C(7)	-177.90(15)
N(1)-C(1)-C(6)-C(5)	171.41(13)
C(2)-C(1)-C(6)-C(5)	-2.28(19)
N(1)-C(1)-C(6)-C(7)	-10.3(2)
C(2)-C(1)-C(6)-C(7)	176.02(13)
C(5)-C(6)-C(7)-C(8)	-48.1(2)
C(1)-C(6)-C(7)-C(8)	133.57(15)
C(5)-C(6)-C(7)-C(9)	74.90(17)
C(1)-C(6)-C(7)-C(9)	-103.39(15)
C(3)-C(2)-C(10)-C(12)	88.88(18)
C(1)-C(2)-C(10)-C(12)	-89.21(17)
C(3)-C(2)-C(10)-C(11)	-36.9(2)
C(1)-C(2)-C(10)-C(11)	145.00(15)
N(3)-C(14)-C(15)-C(16)	-175.22(13)
C(19)-C(14)-C(15)-C(16)	-0.16(19)
C(14)-C(15)-C(16)-C(17)	-0.8(2)
C(15)-C(16)-C(17)-C(18)	1.2(2)
C(16)-C(17)-C(18)-C(19)	-0.5(2)
C(17)-C(18)-C(19)-C(14)	-0.4(2)
C(17)-C(18)-C(19)-N(2)	175.06(12)
C(15)-C(14)-C(19)-C(18)	0.79(19)
N(3)-C(14)-C(19)-C(18)	176.56(12)
C(15)-C(14)-C(19)-N(2)	-175.40(11)
N(3)-C(14)-C(19)-N(2)	0.37(15)
C(25)-C(20)-C(21)-C(22)	-0.2(2)

N(2)-C(20)-C(21)-C(22)	-179.86(14)
C(20)-C(21)-C(22)-C(23)	1.4(3)
C(21)-C(22)-C(23)-C(24)	-0.8(3)
C(22)-C(23)-C(24)-C(25)	-0.8(3)
C(23)-C(24)-C(25)-N(4)	-178.88(14)
C(23)-C(24)-C(25)-C(20)	2.0(2)
C(21)-C(20)-C(25)-C(24)	-1.5(2)
N(2)-C(20)-C(25)-C(24)	178.24(11)
C(21)-C(20)-C(25)-N(4)	179.26(12)
N(2)-C(20)-C(25)-N(4)	-1.04(15)
C(6)-C(1)-N(1)-P(1)	65.28(19)
C(2)-C(1)-N(1)-P(1)	-120.93(13)
C(18)-C(19)-N(2)-C(20)	59.29(18)
C(14)-C(19)-N(2)-C(20)	-124.86(12)
C(18)-C(19)-N(2)-P(1)	-166.50(11)
C(14)-C(19)-N(2)-P(1)	9.35(12)
C(21)-C(20)-N(2)-C(19)	-50.82(19)
C(25)-C(20)-N(2)-C(19)	129.51(12)
C(21)-C(20)-N(2)-P(1)	175.03(12)
C(25)-C(20)-N(2)-P(1)	-4.64(13)
C(15)-C(14)-N(3)-C(13)	5.5(2)
C(19)-C(14)-N(3)-C(13)	-169.95(12)
C(15)-C(14)-N(3)-P(1)	165.16(11)
C(19)-C(14)-N(3)-P(1)	-10.24(14)
C(24)-C(25)-N(4)-C(26)	-5.8(2)
C(20)-C(25)-N(4)-C(26)	173.44(12)
C(24)-C(25)-N(4)-P(1)	-172.69(11)
C(20)-C(25)-N(4)-P(1)	6.53(14)
C(1)-N(1)-P(1)-N(4)	163.08(13)
C(1)-N(1)-P(1)-N(3)	27.48(16)
C(1)-N(1)-P(1)-N(2)	-88.25(15)
C(25)-N(4)-P(1)-N(1)	124.83(9)
C(26)-N(4)-P(1)-N(1)	-41.72(13)
C(25)-N(4)-P(1)-N(3)	-102.22(10)
C(26)-N(4)-P(1)-N(3)	91.23(13)
C(25)-N(4)-P(1)-N(2)	-7.88(9)

C(26)-N(4)-P(1)-N(2)	-174.43(12)
C(14)-N(3)-P(1)-N(1)	-122.31(9)
C(13)-N(3)-P(1)-N(1)	36.63(14)
C(14)-N(3)-P(1)-N(4)	108.00(10)
C(13)-N(3)-P(1)-N(4)	-93.05(13)
C(14)-N(3)-P(1)-N(2)	13.38(10)
C(13)-N(3)-P(1)-N(2)	172.33(12)
C(19)-N(2)-P(1)-N(1)	111.98(9)
C(20)-N(2)-P(1)-N(1)	-107.88(9)
C(19)-N(2)-P(1)-N(4)	-132.96(8)
C(20)-N(2)-P(1)-N(4)	7.17(9)
C(19)-N(2)-P(1)-N(3)	-13.03(9)
C(20)-N(2)-P(1)-N(3)	127.10(9)

Symmetry transformations used to generate equivalent atoms:

Compound **7:**

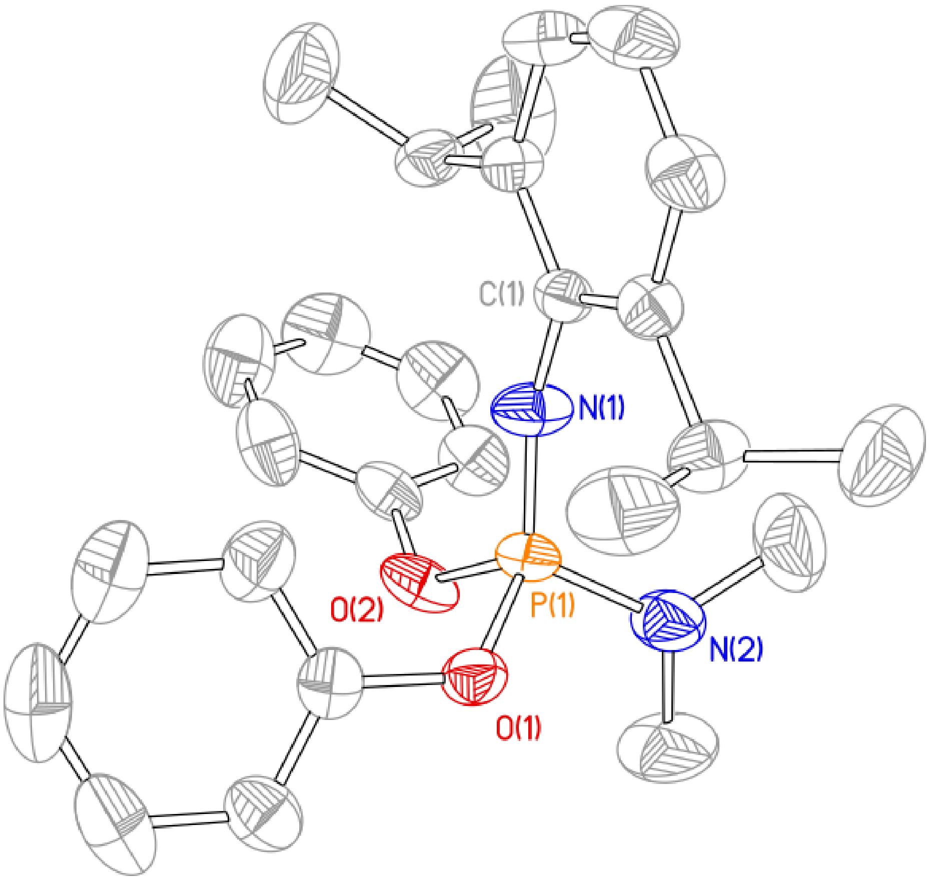

Table S13. Crystal data and structure refinement for **7**.

Identification code	yzl21m	
Empirical formula	C26 H33 N2 O2 P	
Formula weight	436.51	
Temperature	213(2) K	
Wavelength	0.71073 Å	
Crystal system	Monoclinic	
Space group	$P2_1/n$	
Unit cell dimensions	a = 8.587(2) Å	α= 90°.
	b = 17.687(5) Å	β= 99.539(6)°.
	c = 16.430(5) Å	γ = 90°.
Volume	2460.9(12) $Å^3$	
Z	4	
Density (calculated)	1.178 Mg/m^3	
Absorption coefficient	0.136 mm^{-1}	
F(000)	936	
Crystal size	0.200 x 0.170 x 0.080 mm^3	
Theta range for data collection	1.704 to 24.998°.	
Index ranges	-7<=h<=10, -16<=k<=21, -19<=l<=19	
Reflections collected	11025	
Independent reflections	4251 [R(int) = 0.0270]	
Completeness to theta = 24.998°	98.0 %	
Absorption correction	Semi-empirical from equivalents	
Max. and min. transmission	0.9892 and 0.243577	
Refinement method	Full-matrix least-squares on F^2	
Data / restraints / parameters	4251 / 0 / 286	
Goodness-of-fit on F^2	0.997	
Final R indices [I>2sigma(I)]	R1 = 0.0494, wR2 = 0.1325	
R indices (all data)	R1 = 0.0576, wR2 = 0.1385	
Extinction coefficient	n/a	
Largest diff. peak and hole	0.366 and -0.254 e.$Å^{-3}$	

Table S14. Atomic coordinates (x 10^4) and equivalent isotropic displacement parameters (Å^{2}x 10^3) for **7**. U(eq) is defined as one third of the trace of the orthogonalized U^{ij} tensor.

	x	y	z	U(eq)
C(1)	5173(2)	2851(1)	6199(1)	33(1)
C(2)	5929(2)	3558(1)	6214(1)	39(1)
C(3)	6863(3)	3712(1)	5620(2)	53(1)
C(4)	7072(3)	3184(1)	5031(2)	56(1)
C(5)	6364(3)	2491(1)	5033(1)	49(1)
C(6)	5408(2)	2301(1)	5609(1)	38(1)
C(7)	4701(3)	1518(1)	5581(1)	49(1)
C(8)	3831(4)	1305(2)	4731(2)	79(1)
C(9)	5964(4)	930(1)	5885(2)	76(1)
C(10)	5688(3)	4148(1)	6845(1)	49(1)
C(11)	7195(4)	4515(2)	7276(2)	95(1)
C(12)	4518(5)	4743(2)	6486(2)	100(1)
C(13)	5070(3)	1366(1)	8206(1)	40(1)
C(14)	6466(3)	1747(1)	8185(2)	57(1)
C(15)	7693(3)	1653(2)	8820(2)	72(1)
C(16)	7545(4)	1196(2)	9469(2)	79(1)
C(17)	6152(4)	809(2)	9483(2)	75(1)
C(18)	4892(3)	892(1)	8842(1)	52(1)
C(19)	3005(3)	3386(1)	8253(1)	43(1)
C(20)	4234(3)	3680(2)	8786(1)	62(1)
C(21)	4237(4)	4444(2)	8971(2)	80(1)
C(22)	3006(4)	4897(2)	8621(2)	80(1)
C(23)	1795(4)	4596(2)	8094(2)	73(1)
C(24)	1768(3)	3838(1)	7904(2)	56(1)
C(25)	846(4)	2367(2)	5937(2)	86(1)
C(26)	242(3)	1744(2)	7181(2)	91(1)
N(1)	4195(2)	2730(1)	6780(1)	46(1)
N(2)	1436(2)	2072(1)	6748(1)	58(1)
O(1)	3786(2)	1442(1)	7569(1)	40(1)
O(2)	2996(2)	2615(1)	8101(1)	48(1)
P(1)	3188(1)	2260(1)	7229(1)	36(1)

Table S15. Bond lengths [Å] and angles [°] for **7**.

C(1)-N(1)	1.390(2)
C(1)-C(2)	1.408(3)
C(1)-C(6)	1.410(3)
C(2)-C(3)	1.389(3)
C(2)-C(10)	1.510(3)
C(3)-C(4)	1.377(3)
C(3)-H(3)	0.9400
C(4)-C(5)	1.368(3)
C(4)-H(4)	0.9400
C(5)-C(6)	1.392(3)
C(5)-H(5)	0.9400
C(6)-C(7)	1.510(3)
C(7)-C(8)	1.519(4)
C(7)-C(9)	1.526(4)
C(7)-H(7)	0.9900
C(8)-H(8A)	0.9700
C(8)-H(8B)	0.9700
C(8)-H(8C)	0.9700
C(9)-H(9A)	0.9700
C(9)-H(9B)	0.9700
C(9)-H(9C)	0.9700
C(10)-C(12)	1.507(4)
C(10)-C(11)	1.514(4)
C(10)-H(10)	0.9900
C(11)-H(11A)	0.9700
C(11)-H(11B)	0.9700
C(11)-H(11C)	0.9700
C(12)-H(12A)	0.9700
C(12)-H(12B)	0.9700
C(12)-H(12C)	0.9700
C(13)-C(18)	1.366(3)
C(13)-C(14)	1.381(3)
C(13)-O(1)	1.396(3)
C(14)-C(15)	1.365(4)

C(14)-H(14)	0.9400
C(15)-C(16)	1.361(5)
C(15)-H(15)	0.9400
C(16)-C(17)	1.381(5)
C(16)-H(16)	0.9400
C(17)-C(18)	1.388(4)
C(17)-H(17)	0.9400
C(18)-H(18)	0.9400
C(19)-C(20)	1.358(3)
C(19)-C(24)	1.376(3)
C(19)-O(2)	1.387(3)
C(20)-C(21)	1.385(4)
C(20)-H(20)	0.9400
C(21)-C(22)	1.375(4)
C(21)-H(21)	0.9400
C(22)-C(23)	1.347(4)
C(22)-H(22)	0.9400
C(23)-C(24)	1.376(4)
C(23)-H(23)	0.9400
C(24)-H(24)	0.9400
C(25)-N(2)	1.442(3)
C(25)-H(25A)	0.9700
C(25)-H(25B)	0.9700
C(25)-H(25C)	0.9700
C(26)-N(2)	1.462(3)
C(26)-H(26A)	0.9700
C(26)-H(26B)	0.9700
C(26)-H(26C)	0.9700
N(1)-P(1)	1.4810(17)
N(2)-P(1)	1.613(2)
O(1)-P(1)	1.6044(15)
O(2)-P(1)	1.5985(15)
N(1)-C(1)-C(2)	116.99(17)
N(1)-C(1)-C(6)	122.58(17)
C(2)-C(1)-C(6)	120.43(17)
C(3)-C(2)-C(1)	118.66(19)

C(3)-C(2)-C(10)	120.59(19)
C(1)-C(2)-C(10)	120.72(17)
C(4)-C(3)-C(2)	121.2(2)
C(4)-C(3)-H(3)	119.4
C(2)-C(3)-H(3)	119.4
C(5)-C(4)-C(3)	119.7(2)
C(5)-C(4)-H(4)	120.1
C(3)-C(4)-H(4)	120.1
C(4)-C(5)-C(6)	122.0(2)
C(4)-C(5)-H(5)	119.0
C(6)-C(5)-H(5)	119.0
C(5)-C(6)-C(1)	117.91(19)
C(5)-C(6)-C(7)	118.70(19)
C(1)-C(6)-C(7)	123.37(17)
C(6)-C(7)-C(8)	112.9(2)
C(6)-C(7)-C(9)	110.8(2)
C(8)-C(7)-C(9)	110.2(2)
C(6)-C(7)-H(7)	107.6
C(8)-C(7)-H(7)	107.6
C(9)-C(7)-H(7)	107.6
C(7)-C(8)-H(8A)	109.5
C(7)-C(8)-H(8B)	109.5
H(8A)-C(8)-H(8B)	109.5
C(7)-C(8)-H(8C)	109.5
H(8A)-C(8)-H(8C)	109.5
H(8B)-C(8)-H(8C)	109.5
C(7)-C(9)-H(9A)	109.5
C(7)-C(9)-H(9B)	109.5
H(9A)-C(9)-H(9B)	109.5
C(7)-C(9)-H(9C)	109.5
H(9A)-C(9)-H(9C)	109.5
H(9B)-C(9)-H(9C)	109.5
C(12)-C(10)-C(2)	111.8(2)
C(12)-C(10)-C(11)	110.3(3)
C(2)-C(10)-C(11)	114.5(2)
C(12)-C(10)-H(10)	106.6

C(2)-C(10)-H(10)	106.6
C(11)-C(10)-H(10)	106.6
C(10)-C(11)-H(11A)	109.5
C(10)-C(11)-H(11B)	109.5
H(11A)-C(11)-H(11B)	109.5
C(10)-C(11)-H(11C)	109.5
H(11A)-C(11)-H(11C)	109.5
H(11B)-C(11)-H(11C)	109.5
C(10)-C(12)-H(12A)	109.5
C(10)-C(12)-H(12B)	109.5
H(12A)-C(12)-H(12B)	109.5
C(10)-C(12)-H(12C)	109.5
H(12A)-C(12)-H(12C)	109.5
H(12B)-C(12)-H(12C)	109.5
C(18)-C(13)-C(14)	121.8(2)
C(18)-C(13)-O(1)	117.2(2)
C(14)-C(13)-O(1)	121.0(2)
C(15)-C(14)-C(13)	118.9(3)
C(15)-C(14)-H(14)	120.5
C(13)-C(14)-H(14)	120.5
C(16)-C(15)-C(14)	120.7(3)
C(16)-C(15)-H(15)	119.6
C(14)-C(15)-H(15)	119.6
C(15)-C(16)-C(17)	120.1(3)
C(15)-C(16)-H(16)	120.0
C(17)-C(16)-H(16)	120.0
C(16)-C(17)-C(18)	120.2(3)
C(16)-C(17)-H(17)	119.9
C(18)-C(17)-H(17)	119.9
C(13)-C(18)-C(17)	118.2(3)
C(13)-C(18)-H(18)	120.9
C(17)-C(18)-H(18)	120.9
C(20)-C(19)-C(24)	120.7(2)
C(20)-C(19)-O(2)	118.2(2)
C(24)-C(19)-O(2)	121.0(2)
C(19)-C(20)-C(21)	119.2(3)

C(19)-C(20)-H(20)	120.4
C(21)-C(20)-H(20)	120.4
C(22)-C(21)-C(20)	120.3(3)
C(22)-C(21)-H(21)	119.9
C(20)-C(21)-H(21)	119.9
C(23)-C(22)-C(21)	119.7(3)
C(23)-C(22)-H(22)	120.1
C(21)-C(22)-H(22)	120.1
C(22)-C(23)-C(24)	120.9(3)
C(22)-C(23)-H(23)	119.5
C(24)-C(23)-H(23)	119.5
C(19)-C(24)-C(23)	119.2(3)
C(19)-C(24)-H(24)	120.4
C(23)-C(24)-H(24)	120.4
N(2)-C(25)-H(25A)	109.5
N(2)-C(25)-H(25B)	109.5
H(25A)-C(25)-H(25B)	109.5
N(2)-C(25)-H(25C)	109.5
H(25A)-C(25)-H(25C)	109.5
H(25B)-C(25)-H(25C)	109.5
N(2)-C(26)-H(26A)	109.5
N(2)-C(26)-H(26B)	109.5
H(26A)-C(26)-H(26B)	109.5
N(2)-C(26)-H(26C)	109.5
H(26A)-C(26)-H(26C)	109.5
H(26B)-C(26)-H(26C)	109.5
C(1)-N(1)-P(1)	153.82(15)
C(25)-N(2)-C(26)	115.2(2)
C(25)-N(2)-P(1)	122.31(19)
C(26)-N(2)-P(1)	121.0(2)
C(13)-O(1)-P(1)	121.03(12)
C(19)-O(2)-P(1)	123.36(12)
N(1)-P(1)-O(2)	112.35(9)
N(1)-P(1)-O(1)	120.04(9)
O(2)-P(1)-O(1)	96.70(8)
N(1)-P(1)-N(2)	116.46(11)

O(2)-P(1)-N(2)	107.06(11)
O(1)-P(1)-N(2)	101.79(9)

Symmetry transformations used to generate equivalent atoms:

Table 16. Anisotropic displacement parameters ($Å^2$x 10^3) for **7**. The anisotropic displacement factor exponent takes the form: $-2\pi^2[h^2 a^{*2}U^{11} + ... + 2 h k a^* b^* U^{12}]$

	U^{11}	U^{22}	U^{33}	U^{23}	U^{13}	U^{12}
C(1)	32(1)	36(1)	32(1)	6(1)	8(1)	0(1)
C(2)	37(1)	39(1)	41(1)	6(1)	7(1)	-4(1)
C(3)	50(1)	49(1)	64(2)	9(1)	21(1)	-12(1)
C(4)	53(1)	68(2)	56(1)	10(1)	29(1)	-3(1)
C(5)	49(1)	59(1)	43(1)	-2(1)	18(1)	6(1)
C(6)	35(1)	42(1)	38(1)	1(1)	8(1)	3(1)
C(7)	54(1)	41(1)	54(1)	-10(1)	20(1)	-3(1)
C(8)	83(2)	73(2)	78(2)	-25(2)	6(2)	-17(2)
C(9)	87(2)	43(1)	98(2)	6(1)	19(2)	5(1)
C(10)	57(2)	39(1)	50(1)	-2(1)	10(1)	-15(1)
C(11)	87(2)	113(3)	78(2)	-29(2)	-5(2)	-28(2)
C(12)	124(3)	80(2)	89(2)	-35(2)	-2(2)	32(2)
C(13)	42(1)	34(1)	45(1)	-7(1)	4(1)	7(1)
C(14)	47(1)	57(2)	67(2)	-3(1)	2(1)	-2(1)
C(15)	54(2)	76(2)	80(2)	-16(2)	-8(2)	2(1)
C(16)	75(2)	91(2)	60(2)	-22(2)	-20(2)	20(2)
C(17)	98(3)	74(2)	49(2)	5(1)	2(2)	27(2)
C(18)	60(2)	46(1)	50(1)	3(1)	9(1)	11(1)
C(19)	53(1)	46(1)	34(1)	6(1)	20(1)	11(1)
C(20)	67(2)	77(2)	40(1)	-6(1)	4(1)	21(1)
C(21)	92(2)	87(2)	59(2)	-31(2)	7(2)	-1(2)
C(22)	106(3)	54(2)	86(2)	-19(2)	31(2)	11(2)
C(23)	82(2)	53(2)	84(2)	1(1)	18(2)	26(2)
C(24)	54(2)	54(1)	63(2)	4(1)	12(1)	14(1)
C(25)	73(2)	107(3)	69(2)	14(2)	-14(2)	11(2)
C(26)	42(2)	103(2)	127(3)	41(2)	16(2)	-16(2)
N(1)	60(1)	35(1)	51(1)	-4(1)	29(1)	-7(1)
N(2)	40(1)	65(1)	68(1)	25(1)	2(1)	-4(1)
O(1)	39(1)	34(1)	48(1)	5(1)	3(1)	-3(1)
O(2)	65(1)	42(1)	44(1)	11(1)	27(1)	11(1)
P(1)	37(1)	33(1)	39(1)	6(1)	13(1)	-1(1)

Table S17. Hydrogen coordinates (x 10^4) and isotropic displacement parameters ($Å^2$x 10^3) for **7**.

	x	y	z	U(eq)
H(3)	7362	4185	5619	64
H(4)	7699	3299	4630	68
H(5)	6528	2132	4634	59
H(7)	3924	1512	5964	58
H(8A)	4580	1266	4350	118
H(8B)	3055	1691	4537	118
H(8C)	3305	824	4761	118
H(9A)	5484	432	5867	113
H(9B)	6445	1046	6448	113
H(9C)	6767	937	5533	113
H(10)	5218	3884	7277	58
H(11A)	7697	4783	6875	142
H(11B)	7905	4128	7542	142
H(11C)	6948	4867	7689	142
H(12A)	4930	5016	6055	150
H(12B)	4344	5093	6916	150
H(12C)	3527	4503	6255	150
H(14)	6570	2066	7739	69
H(15)	8651	1908	8810	87
H(16)	8392	1142	9908	95
H(17)	6057	489	9929	90
H(18)	3941	629	8844	63
H(20)	5071	3368	9027	74
H(21)	5086	4654	9336	96
H(22)	3008	5415	8749	96
H(23)	958	4908	7854	87
H(24)	914	3631	7540	68
H(25A)	290	1971	5598	129
H(25B)	1723	2546	5685	129
H(25C)	129	2783	5982	129

H(26A)	-580	2114	7213	136
H(26B)	729	1598	7734	136
H(26C)	-215	1301	6884	136

Table S18. Torsion angles [°] for **7**.

N(1)-C(1)-C(2)-C(3)	-177.3(2)
C(6)-C(1)-C(2)-C(3)	2.2(3)
N(1)-C(1)-C(2)-C(10)	0.8(3)
C(6)-C(1)-C(2)-C(10)	-179.76(19)
C(1)-C(2)-C(3)-C(4)	-0.9(3)
C(10)-C(2)-C(3)-C(4)	-178.9(2)
C(2)-C(3)-C(4)-C(5)	-0.7(4)
C(3)-C(4)-C(5)-C(6)	1.0(4)
C(4)-C(5)-C(6)-C(1)	0.3(3)
C(4)-C(5)-C(6)-C(7)	-178.6(2)
N(1)-C(1)-C(6)-C(5)	177.5(2)
C(2)-C(1)-C(6)-C(5)	-1.9(3)
N(1)-C(1)-C(6)-C(7)	-3.6(3)
C(2)-C(1)-C(6)-C(7)	176.92(19)
C(5)-C(6)-C(7)-C(8)	-52.1(3)
C(1)-C(6)-C(7)-C(8)	129.1(2)
C(5)-C(6)-C(7)-C(9)	72.1(3)
C(1)-C(6)-C(7)-C(9)	-106.7(2)
C(3)-C(2)-C(10)-C(12)	77.3(3)
C(1)-C(2)-C(10)-C(12)	-100.7(3)
C(3)-C(2)-C(10)-C(11)	-49.0(3)
C(1)-C(2)-C(10)-C(11)	132.9(2)
C(18)-C(13)-C(14)-C(15)	-0.8(4)
O(1)-C(13)-C(14)-C(15)	-179.7(2)
C(13)-C(14)-C(15)-C(16)	-0.4(4)
C(14)-C(15)-C(16)-C(17)	1.1(5)
C(15)-C(16)-C(17)-C(18)	-0.7(4)

C(14)-C(13)-C(18)-C(17)	1.2(3)
O(1)-C(13)-C(18)-C(17)	-179.9(2)
C(16)-C(17)-C(18)-C(13)	-0.4(4)
C(24)-C(19)-C(20)-C(21)	-0.6(4)
O(2)-C(19)-C(20)-C(21)	-177.6(2)
C(19)-C(20)-C(21)-C(22)	0.4(4)
C(20)-C(21)-C(22)-C(23)	-0.3(5)
C(21)-C(22)-C(23)-C(24)	0.4(5)
C(20)-C(19)-C(24)-C(23)	0.7(4)
O(2)-C(19)-C(24)-C(23)	177.6(2)
C(22)-C(23)-C(24)-C(19)	-0.6(4)
C(2)-C(1)-N(1)-P(1)	-171.7(3)
C(6)-C(1)-N(1)-P(1)	8.9(5)
C(18)-C(13)-O(1)-P(1)	132.35(17)
C(14)-C(13)-O(1)-P(1)	-48.7(2)
C(20)-C(19)-O(2)-P(1)	-112.7(2)
C(24)-C(19)-O(2)-P(1)	70.2(2)
C(1)-N(1)-P(1)-O(2)	158.3(4)
C(1)-N(1)-P(1)-O(1)	45.8(4)
C(1)-N(1)-P(1)-N(2)	-77.7(4)
C(19)-O(2)-P(1)-N(1)	31.5(2)
C(19)-O(2)-P(1)-O(1)	157.88(17)
C(19)-O(2)-P(1)-N(2)	-97.57(18)
C(13)-O(1)-P(1)-N(1)	67.47(18)
C(13)-O(1)-P(1)-O(2)	-53.20(15)
C(13)-O(1)-P(1)-N(2)	-162.24(15)
C(25)-N(2)-P(1)-N(1)	-2.2(3)
C(26)-N(2)-P(1)-N(1)	-167.9(2)
C(25)-N(2)-P(1)-O(2)	124.5(2)
C(26)-N(2)-P(1)-O(2)	-41.2(3)
C(25)-N(2)-P(1)-O(1)	-134.7(2)
C(26)-N(2)-P(1)-O(1)	59.7(2)

Symmetry transformations used to generate equivalent atoms:

Compound **8**:

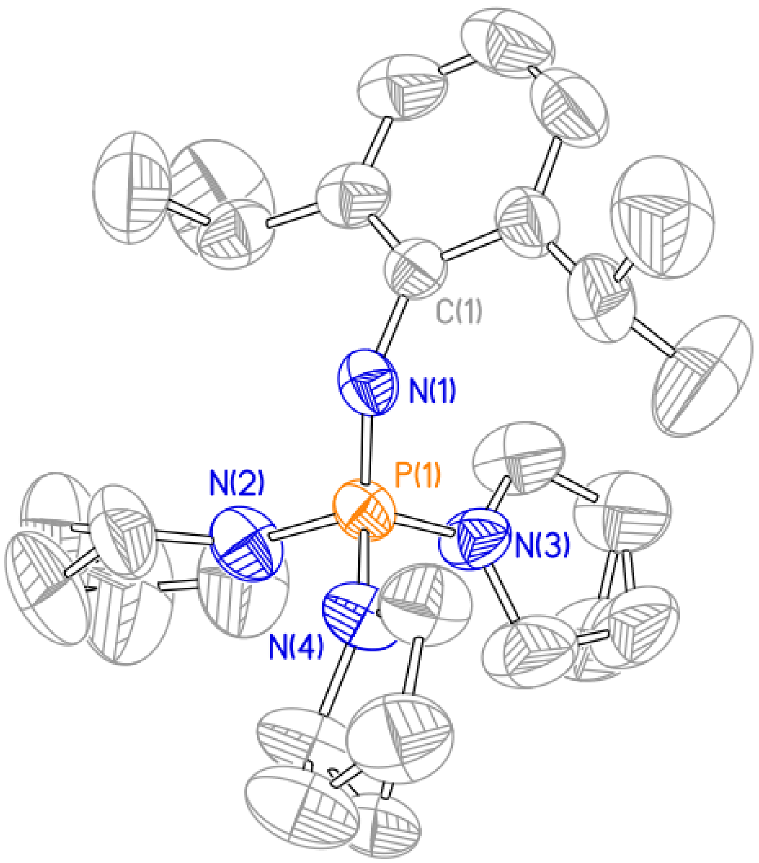

Table S19. Crystal data and structure refinement for **8**.

Identification code	yzl22s	
Empirical formula	C24 H41 N4 P	
Formula weight	416.58	
Temperature	293(2) K	
Wavelength	0.71073 Å	
Crystal system	Monoclinic	
Space group	$P2_1/c$	
Unit cell dimensions	a = 8.3589(17) Å	α= 90°.
	b = 16.491(3) Å	β= 93.77(3)°.
	c = 18.049(4) Å	γ = 90°.
Volume	2482.6(9) $Å^3$	
Z	4	
Density (calculated)	1.115 Mg/m^3	
Absorption coefficient	0.127 mm^{-1}	
F(000)	912	
Crystal size	0.24 x 0.19 x 0.13 mm^3	
Theta range for data collection	1.674 to 24.993°.	
Index ranges	-9<=h<=9, -18<=k<=19, -21<=l<=21	
Reflections collected	15379	
Independent reflections	4358 [R(int) = 0.0304]	
Completeness to theta = 24.993°	99.9 %	
Absorption correction	Empirical	
Max. and min. transmission	0.50 and 0.209649	
Refinement method	Full-matrix least-squares on F^2	
Data / restraints / parameters	4358 / 18 / 291	
Goodness-of-fit on F^2	1.086	
Final R indices [I>2sigma(I)]	R1 = 0.0798, wR2 = 0.1936	
R indices (all data)	R1 = 0.1026, wR2 = 0.2070	
Extinction coefficient	n/a	
Largest diff. peak and hole	0.316 and -0.330 e.$Å^{-3}$	

Table S20. Atomic coordinates (x 10^4) and equivalent isotropic displacement parameters ($Å^2$x 10^3) for **8**. U(eq) is defined as one third of the trace of the orthogonalized U^{ij} tensor.

	x	y	z	U(eq)
C(1)	3155(3)	6017(2)	2301(2)	49(1)
C(2)	3481(4)	6399(2)	2999(2)	61(1)
C(3)	3778(5)	7229(3)	3014(3)	80(1)
C(4)	3799(5)	7688(3)	2386(3)	92(1)
C(5)	3550(5)	7312(3)	1720(3)	84(1)
C(6)	3231(4)	6489(2)	1647(2)	61(1)
C(7)	2997(5)	6095(3)	893(2)	81(1)
C(8)	4145(7)	6385(4)	331(3)	127(2)
C(9)	1305(7)	6163(5)	573(3)	145(3)
C(10)	3586(5)	5901(3)	3704(2)	71(1)
C(11)	5279(7)	5611(4)	3876(3)	135(2)
C(12)	2976(10)	6319(4)	4366(3)	167(3)
C(13)	2704(6)	3721(3)	3498(3)	97(2)
C(14A)	2120(30)	3277(11)	4117(10)	126(6)
C(14B)	2200(40)	3730(18)	4319(11)	146(9)
C(15)	598(12)	3712(8)	4272(5)	214(5)
C(16)	29(7)	4158(4)	3607(3)	131(2)
C(17)	-1672(6)	4601(3)	1782(4)	109(2)
C(18A)	-2950(30)	5260(20)	1607(17)	118(9)
C(18B)	-3000(30)	5090(30)	1870(20)	128(12)
C(19)	-2365(5)	5970(3)	1975(4)	116(2)
C(20)	-797(5)	5809(3)	2383(3)	88(1)
C(21)	2422(5)	3888(3)	1071(2)	77(1)
C(22)	2307(7)	3041(3)	755(3)	96(2)
C(23A)	880(20)	2692(7)	1134(7)	90(4)
C(23B)	1770(30)	2506(9)	1347(12)	100(5)
C(24)	997(7)	3024(2)	1874(3)	100(2)
N(1)	2873(3)	5187(2)	2249(1)	48(1)
N(2)	1334(4)	4168(2)	3140(2)	67(1)
N(3)	-308(3)	5012(2)	2143(2)	60(1)
N(4)	1671(4)	3842(2)	1774(2)	62(1)

P(1)	1467(1)	4610(1)	2330(1)	46(1)

Table S21. Bond lengths [Å] and angles [°] for **8**.

C(1)-N(1)	1.392(4)
C(1)-C(6)	1.418(5)
C(1)-C(2)	1.418(5)
C(2)-C(3)	1.391(5)
C(2)-C(10)	1.513(5)
C(3)-C(4)	1.364(6)
C(3)-H(3)	0.9300
C(4)-C(5)	1.356(6)
C(4)-H(4)	0.9300
C(5)-C(6)	1.389(6)
C(5)-H(5)	0.9300
C(6)-C(7)	1.508(6)
C(7)-C(9)	1.496(7)
C(7)-C(8)	1.519(6)
C(7)-H(7)	0.9800
C(8)-H(8A)	0.9600
C(8)-H(8B)	0.9600
C(8)-H(8C)	0.9600
C(9)-H(9A)	0.9600
C(9)-H(9B)	0.9600
C(9)-H(9C)	0.9600
C(10)-C(12)	1.498(6)
C(10)-C(11)	1.506(7)
C(10)-H(10)	0.9800
C(11)-H(11A)	0.9600
C(11)-H(11B)	0.9600
C(11)-H(11C)	0.9600
C(12)-H(12A)	0.9600

C(12)-H(12B)	0.9600
C(12)-H(12C)	0.9600
C(13)-C(14A)	1.447(16)
C(13)-N(2)	1.475(5)
C(13)-C(14B)	1.57(2)
C(13)-H(13A)	0.9700
C(13)-H(13B)	0.9700
C(13)-H(13C)	0.9700
C(13)-H(13D)	0.9700
C(14A)-C(15)	1.50(2)
C(14A)-H(14A)	0.9700
C(14A)-H(14B)	0.9700
C(14B)-C(15)	1.33(3)
C(14B)-H(14C)	0.9700
C(14B)-H(14D)	0.9700
C(15)-C(16)	1.461(9)
C(15)-H(15A)	0.9700
C(15)-H(15B)	0.9700
C(15)-H(15C)	0.9700
C(15)-H(15D)	0.9700
C(16)-N(2)	1.422(5)
C(16)-H(16A)	0.9700
C(16)-H(16B)	0.9700
C(17)-C(18B)	1.39(3)
C(17)-N(3)	1.444(5)
C(17)-C(18A)	1.54(3)
C(17)-H(17A)	0.9700
C(17)-H(17B)	0.9700
C(17)-H(17C)	0.9700
C(17)-H(17D)	0.9700
C(18A)-C(19)	1.42(3)
C(18A)-H(18A)	0.9700
C(18A)-H(18B)	0.9700
C(18B)-C(19)	1.55(4)
C(18B)-H(18C)	0.9700
C(18B)-H(18D)	0.9700

C(19)-C(20)	1.485(6)
C(19)-H(19A)	0.9700
C(19)-H(19B)	0.9700
C(19)-H(19C)	0.9700
C(19)-H(19D)	0.9700
C(20)-N(3)	1.452(5)
C(20)-H(20A)	0.9700
C(20)-H(20B)	0.9700
C(21)-N(4)	1.455(4)
C(21)-C(22)	1.510(6)
C(21)-H(21A)	0.9700
C(21)-H(21B)	0.9700
C(22)-C(23B)	1.479(18)
C(22)-C(23A)	1.525(16)
C(22)-H(22A)	0.9700
C(22)-H(22B)	0.9700
C(22)-H(22C)	0.9700
C(22)-H(22D)	0.9700
C(23A)-C(24)	1.441(12)
C(23A)-H(23A)	0.9700
C(23A)-H(23B)	0.9700
C(23B)-C(24)	1.458(14)
C(23B)-H(23C)	0.9700
C(23B)-H(23D)	0.9700
C(24)-N(4)	1.478(5)
C(24)-H(24A)	0.9700
C(24)-H(24B)	0.9700
C(24)-H(24C)	0.9700
C(24)-H(24D)	0.9700
N(1)-P(1)	1.525(3)
N(2)-P(1)	1.644(3)
N(3)-P(1)	1.639(3)
N(4)-P(1)	1.632(3)
N(1)-C(1)-C(6)	120.0(3)
N(1)-C(1)-C(2)	121.3(3)
C(6)-C(1)-C(2)	118.6(3)

C(3)-C(2)-C(1)	118.6(4)
C(3)-C(2)-C(10)	121.1(4)
C(1)-C(2)-C(10)	120.2(3)
C(4)-C(3)-C(2)	122.8(4)
C(4)-C(3)-H(3)	118.6
C(2)-C(3)-H(3)	118.6
C(5)-C(4)-C(3)	118.3(4)
C(5)-C(4)-H(4)	120.9
C(3)-C(4)-H(4)	120.9
C(4)-C(5)-C(6)	123.2(4)
C(4)-C(5)-H(5)	118.4
C(6)-C(5)-H(5)	118.4
C(5)-C(6)-C(1)	118.4(4)
C(5)-C(6)-C(7)	121.2(4)
C(1)-C(6)-C(7)	120.3(3)
C(9)-C(7)-C(6)	112.3(4)
C(9)-C(7)-C(8)	110.0(4)
C(6)-C(7)-C(8)	114.7(4)
C(9)-C(7)-H(7)	106.4
C(6)-C(7)-H(7)	106.4
C(8)-C(7)-H(7)	106.4
C(7)-C(8)-H(8A)	109.5
C(7)-C(8)-H(8B)	109.5
H(8A)-C(8)-H(8B)	109.5
C(7)-C(8)-H(8C)	109.5
H(8A)-C(8)-H(8C)	109.5
H(8B)-C(8)-H(8C)	109.5
C(7)-C(9)-H(9A)	109.5
C(7)-C(9)-H(9B)	109.5
H(9A)-C(9)-H(9B)	109.5
C(7)-C(9)-H(9C)	109.5
H(9A)-C(9)-H(9C)	109.5
H(9B)-C(9)-H(9C)	109.5
C(12)-C(10)-C(11)	110.3(5)
C(12)-C(10)-C(2)	114.7(4)
C(11)-C(10)-C(2)	110.3(3)

C(12)-C(10)-H(10)	107.1
C(11)-C(10)-H(10)	107.1
C(2)-C(10)-H(10)	107.1
C(10)-C(11)-H(11A)	109.5
C(10)-C(11)-H(11B)	109.5
H(11A)-C(11)-H(11B)	109.5
C(10)-C(11)-H(11C)	109.5
H(11A)-C(11)-H(11C)	109.5
H(11B)-C(11)-H(11C)	109.5
C(10)-C(12)-H(12A)	109.5
C(10)-C(12)-H(12B)	109.5
H(12A)-C(12)-H(12B)	109.5
C(10)-C(12)-H(12C)	109.5
H(12A)-C(12)-H(12C)	109.5
H(12B)-C(12)-H(12C)	109.5
C(14A)-C(13)-N(2)	107.4(10)
N(2)-C(13)-C(14B)	99.1(12)
C(14A)-C(13)-H(13A)	110.2
N(2)-C(13)-H(13A)	110.2
C(14A)-C(13)-H(13B)	110.2
N(2)-C(13)-H(13B)	110.2
H(13A)-C(13)-H(13B)	108.5
N(2)-C(13)-H(13C)	111.9
C(14B)-C(13)-H(13C)	111.9
N(2)-C(13)-H(13D)	111.9
C(14B)-C(13)-H(13D)	111.9
H(13C)-C(13)-H(13D)	109.6
C(13)-C(14A)-C(15)	103.6(10)
C(13)-C(14A)-H(14A)	111.0
C(15)-C(14A)-H(14A)	111.0
C(13)-C(14A)-H(14B)	111.0
C(15)-C(14A)-H(14B)	111.0
H(14A)-C(14A)-H(14B)	109.0
C(15)-C(14B)-C(13)	105.8(14)
C(15)-C(14B)-H(14C)	110.6
C(13)-C(14B)-H(14C)	110.6

C(15)-C(14B)-H(14D)	110.6
C(13)-C(14B)-H(14D)	110.6
H(14C)-C(14B)-H(14D)	108.7
C(14B)-C(15)-C(16)	108.2(10)
C(16)-C(15)-C(14A)	108.7(8)
C(16)-C(15)-H(15A)	109.9
C(14A)-C(15)-H(15A)	109.9
C(16)-C(15)-H(15B)	109.9
C(14A)-C(15)-H(15B)	109.9
H(15A)-C(15)-H(15B)	108.3
C(14B)-C(15)-H(15C)	110.1
C(16)-C(15)-H(15C)	110.1
C(14B)-C(15)-H(15D)	110.1
C(16)-C(15)-H(15D)	110.1
H(15C)-C(15)-H(15D)	108.4
N(2)-C(16)-C(15)	105.8(5)
N(2)-C(16)-H(16A)	110.6
C(15)-C(16)-H(16A)	110.6
N(2)-C(16)-H(16B)	110.6
C(15)-C(16)-H(16B)	110.6
H(16A)-C(16)-H(16B)	108.7
C(18B)-C(17)-N(3)	106.7(15)
N(3)-C(17)-C(18A)	106.3(11)
N(3)-C(17)-H(17A)	110.5
C(18A)-C(17)-H(17A)	110.5
N(3)-C(17)-H(17B)	110.5
C(18A)-C(17)-H(17B)	110.5
H(17A)-C(17)-H(17B)	108.7
C(18B)-C(17)-H(17C)	110.4
N(3)-C(17)-H(17C)	110.4
C(18B)-C(17)-H(17D)	110.4
N(3)-C(17)-H(17D)	110.4
H(17C)-C(17)-H(17D)	108.6
C(19)-C(18A)-C(17)	105.8(17)
C(19)-C(18A)-H(18A)	110.6
C(17)-C(18A)-H(18A)	110.6

C(19)-C(18A)-H(18B)	110.6
C(17)-C(18A)-H(18B)	110.6
H(18A)-C(18A)-H(18B)	108.7
C(17)-C(18B)-C(19)	106.8(19)
C(17)-C(18B)-H(18C)	110.4
C(19)-C(18B)-H(18C)	110.4
C(17)-C(18B)-H(18D)	110.4
C(19)-C(18B)-H(18D)	110.4
H(18C)-C(18B)-H(18D)	108.6
C(18A)-C(19)-C(20)	110.5(12)
C(20)-C(19)-C(18B)	100.3(12)
C(18A)-C(19)-H(19A)	109.6
C(20)-C(19)-H(19A)	109.6
C(18A)-C(19)-H(19B)	109.6
C(20)-C(19)-H(19B)	109.6
H(19A)-C(19)-H(19B)	108.1
C(20)-C(19)-H(19C)	111.7
C(18B)-C(19)-H(19C)	111.7
C(20)-C(19)-H(19D)	111.7
C(18B)-C(19)-H(19D)	111.7
H(19C)-C(19)-H(19D)	109.5
N(3)-C(20)-C(19)	105.7(4)
N(3)-C(20)-H(20A)	110.6
C(19)-C(20)-H(20A)	110.6
N(3)-C(20)-H(20B)	110.6
C(19)-C(20)-H(20B)	110.6
H(20A)-C(20)-H(20B)	108.7
N(4)-C(21)-C(22)	105.0(3)
N(4)-C(21)-H(21A)	110.7
C(22)-C(21)-H(21A)	110.7
N(4)-C(21)-H(21B)	110.7
C(22)-C(21)-H(21B)	110.7
H(21A)-C(21)-H(21B)	108.8
C(23B)-C(22)-C(21)	107.2(6)
C(21)-C(22)-C(23A)	102.3(6)
C(21)-C(22)-H(22A)	111.3

C(23A)-C(22)-H(22A)	111.3
C(21)-C(22)-H(22B)	111.3
C(23A)-C(22)-H(22B)	111.3
H(22A)-C(22)-H(22B)	109.2
C(23B)-C(22)-H(22C)	110.3
C(21)-C(22)-H(22C)	110.3
C(23B)-C(22)-H(22D)	110.3
C(21)-C(22)-H(22D)	110.3
H(22C)-C(22)-H(22D)	108.5
C(24)-C(23A)-C(22)	105.3(8)
C(24)-C(23A)-H(23A)	110.7
C(22)-C(23A)-H(23A)	110.7
C(24)-C(23A)-H(23B)	110.7
C(22)-C(23A)-H(23B)	110.7
H(23A)-C(23A)-H(23B)	108.8
C(24)-C(23B)-C(22)	106.9(10)
C(24)-C(23B)-H(23C)	110.3
C(22)-C(23B)-H(23C)	110.3
C(24)-C(23B)-H(23D)	110.3
C(22)-C(23B)-H(23D)	110.3
H(23C)-C(23B)-H(23D)	108.6
C(23A)-C(24)-N(4)	103.7(6)
C(23B)-C(24)-N(4)	105.5(7)
C(23A)-C(24)-H(24A)	111.0
N(4)-C(24)-H(24A)	111.0
C(23A)-C(24)-H(24B)	111.0
N(4)-C(24)-H(24B)	111.0
H(24A)-C(24)-H(24B)	109.0
C(23B)-C(24)-H(24C)	110.6
N(4)-C(24)-H(24C)	110.6
C(23B)-C(24)-H(24D)	110.6
N(4)-C(24)-H(24D)	110.6
H(24C)-C(24)-H(24D)	108.8
C(1)-N(1)-P(1)	137.3(2)
C(16)-N(2)-C(13)	109.8(4)
C(16)-N(2)-P(1)	129.2(3)

C(13)-N(2)-P(1)	121.0(3)
C(17)-N(3)-C(20)	109.3(3)
C(17)-N(3)-P(1)	125.5(3)
C(20)-N(3)-P(1)	125.0(3)
C(21)-N(4)-C(24)	110.1(3)
C(21)-N(4)-P(1)	124.3(2)
C(24)-N(4)-P(1)	125.3(3)
N(1)-P(1)-N(4)	108.23(14)
N(1)-P(1)-N(3)	114.91(15)
N(4)-P(1)-N(3)	108.45(16)
N(1)-P(1)-N(2)	117.32(15)
N(4)-P(1)-N(2)	102.58(15)
N(3)-P(1)-N(2)	104.41(16)

Symmetry transformations used to generate equivalent atoms:

Table S22. Anisotropic displacement parameters ($Å^2 x 10^3$) for 8. The anisotropic displacement factor exponent takes the form: $-2\pi^2[h^2 a^{*2}U^{11} + ... + 2 h k a^* b^* U^{12}]$

	U^{11}	U^{22}	U^{33}	U^{23}	U^{13}	U^{12}
C(1)	30(2)	50(2)	66(2)	5(2)	3(1)	2(1)
C(2)	42(2)	62(2)	79(2)	-6(2)	10(2)	2(2)
C(3)	65(3)	68(3)	107(3)	-23(3)	7(2)	-6(2)
C(4)	83(3)	50(2)	143(5)	2(3)	16(3)	-4(2)
C(5)	74(3)	62(3)	116(4)	31(3)	10(3)	5(2)
C(6)	47(2)	60(2)	77(3)	21(2)	2(2)	1(2)
C(7)	94(3)	83(3)	65(3)	28(2)	0(2)	-4(2)
C(8)	127(5)	159(5)	97(4)	27(4)	32(3)	2(4)
C(9)	95(4)	242(8)	96(4)	1(5)	-17(3)	-14(5)
C(10)	73(3)	81(3)	58(2)	-13(2)	4(2)	-10(2)
C(11)	105(4)	170(6)	127(5)	57(4)	-9(3)	9(4)
C(12)	249(9)	171(6)	88(4)	-16(4)	61(5)	35(6)
C(13)	112(4)	88(3)	89(3)	38(3)	3(3)	9(3)
C(14A)	188(15)	104(11)	91(11)	48(8)	45(10)	29(12)

C(14B)	225(19)	150(19)	59(10)	58(12)	-26(11)	-20(20)
C(15)	191(9)	325(14)	131(7)	121(8)	59(7)	6(10)
C(16)	107(4)	193(6)	98(4)	46(4)	53(3)	5(4)
C(17)	71(3)	89(3)	163(5)	-22(3)	-19(3)	-4(3)
C(18A)	85(12)	101(14)	161(16)	-31(13)	-48(11)	25(9)
C(18B)	33(8)	120(18)	230(30)	-13(18)	-13(12)	10(8)
C(19)	55(3)	94(4)	198(6)	-10(4)	-6(3)	18(3)
C(20)	55(2)	80(3)	130(4)	-30(3)	8(2)	11(2)
C(21)	94(3)	78(3)	62(2)	-13(2)	19(2)	-8(2)
C(22)	116(4)	84(3)	88(3)	-31(3)	12(3)	5(3)
C(23A)	129(11)	39(5)	96(7)	-14(4)	-24(7)	-6(6)
C(23B)	118(13)	55(7)	129(14)	-19(8)	39(10)	-1(7)
C(24)	123(4)	52(2)	127(4)	-12(3)	33(3)	-15(3)
N(1)	50(2)	49(2)	47(2)	8(1)	7(1)	3(1)
N(2)	68(2)	69(2)	66(2)	13(2)	24(2)	-2(2)
N(3)	42(2)	60(2)	78(2)	-10(2)	-2(1)	-1(1)
N(4)	70(2)	46(2)	72(2)	-2(1)	17(2)	-2(1)
P(1)	46(1)	43(1)	50(1)	3(1)	10(1)	1(1)

Table S23. Hydrogen coordinates (x 10^4) and isotropic displacement parameters (Å^{2}x 10 ^{3}) for 8.

	x	y	z	U(eq)
H(3)	3971	7482	3472	96
H(4)	3979	8244	2414	110
H(5)	3594	7622	1291	100
H(7)	3208	5516	968	97
H(8A)	5228	6340	541	190
H(8B)	4017	6057	-108	190
H(8C)	3918	6941	206	190
H(9A)	1211	5905	95	218

H(9B)	600	5901	898	218
H(9C)	1017	6724	521	218
H(10)	2920	5418	3610	85
H(11A)	5656	5345	3447	202
H(11B)	5957	6066	4005	202
H(11C)	5303	5238	4284	202
H(12A)	1897	6501	4250	251
H(12B)	2992	5948	4777	251
H(12C)	3646	6777	4497	251
H(13A)	3142	3349	3147	116
H(13B)	3541	4095	3671	116
H(13C)	3711	4001	3448	116
H(13D)	2776	3173	3306	116
H(14A)	2886	3296	4544	151
H(14B)	1908	2715	3986	151
H(14C)	2627	3262	4589	176
H(14D)	2584	4217	4573	176
H(15A)	-207	3324	4405	256
H(15B)	796	4084	4684	256
H(15C)	190	3960	4709	256
H(15D)	224	3155	4242	256
H(16A)	-892	3889	3361	157
H(16B)	-274	4706	3733	157
H(17A)	-2076	4191	2108	131
H(17B)	-1376	4340	1330	131
H(17C)	-1516	4521	1260	131
H(17D)	-1820	4075	2009	131
H(18A)	-3095	5348	1076	142
H(18B)	-3970	5096	1788	142
H(18C)	-3552	4924	2301	153
H(18D)	-3743	5060	1435	153
H(19A)	-3131	6151	2320	139
H(19B)	-2243	6397	1613	139
H(19C)	-2235	6234	1502	139
H(19D)	-3060	6295	2266	139
H(20A)	-15	6215	2264	106

H(20B)	-906	5815	2915	106
H(21A)	3533	4056	1147	93
H(21B)	1861	4271	739	93
H(22A)	3276	2733	882	115
H(22B)	2107	3050	220	115
H(22C)	1546	3026	326	115
H(22D)	3343	2865	604	115
H(23A)	-120	2852	871	107
H(23B)	934	2104	1149	107
H(23C)	1013	2106	1140	119
H(23D)	2673	2225	1592	119
H(24A)	1701	2698	2203	119
H(24B)	-50	3054	2075	119
H(24C)	1231	2835	2378	119
H(24D)	-157	3028	1769	119

Table S24. Torsion angles [°] for **8**.

N(1)-C(1)-C(2)-C(3)	179.0(3)
C(6)-C(1)-C(2)-C(3)	3.4(5)
N(1)-C(1)-C(2)-C(10)	2.2(5)
C(6)-C(1)-C(2)-C(10)	-173.5(3)
C(1)-C(2)-C(3)-C(4)	-1.3(6)
C(10)-C(2)-C(3)-C(4)	175.6(4)
C(2)-C(3)-C(4)-C(5)	-1.3(7)
C(3)-C(4)-C(5)-C(6)	1.8(7)
C(4)-C(5)-C(6)-C(1)	0.3(6)
C(4)-C(5)-C(6)-C(7)	-178.7(4)
N(1)-C(1)-C(6)-C(5)	-178.6(3)
C(2)-C(1)-C(6)-C(5)	-2.9(5)

N(1)-C(1)-C(6)-C(7)	0.4(5)
C(2)-C(1)-C(6)-C(7)	176.1(3)
C(5)-C(6)-C(7)-C(9)	-85.5(5)
C(1)-C(6)-C(7)-C(9)	95.5(5)
C(5)-C(6)-C(7)-C(8)	41.1(6)
C(1)-C(6)-C(7)-C(8)	-137.9(4)
C(3)-C(2)-C(10)-C(12)	37.8(6)
C(1)-C(2)-C(10)-C(12)	-145.5(5)
C(3)-C(2)-C(10)-C(11)	-87.5(5)
C(1)-C(2)-C(10)-C(11)	89.3(5)
N(2)-C(13)-C(14A)-C(15)	-20.3(18)
N(2)-C(13)-C(14B)-C(15)	32(2)
C(13)-C(14B)-C(15)-C(16)	-33(2)
C(13)-C(14A)-C(15)-C(16)	21(2)
C(14B)-C(15)-C(16)-N(2)	21(2)
C(14A)-C(15)-C(16)-N(2)	-14.0(16)
N(3)-C(17)-C(18A)-C(19)	-9(2)
N(3)-C(17)-C(18B)-C(19)	25(3)
C(17)-C(18A)-C(19)-C(20)	-1(2)
C(17)-C(18B)-C(19)-C(20)	-34(3)
C(18A)-C(19)-C(20)-N(3)	10.0(16)
C(18B)-C(19)-C(20)-N(3)	28.9(16)
N(4)-C(21)-C(22)-C(23B)	-10.2(12)
N(4)-C(21)-C(22)-C(23A)	24.2(7)
C(21)-C(22)-C(23A)-C(24)	-36.6(11)
C(21)-C(22)-C(23B)-C(24)	21.0(18)
C(22)-C(23A)-C(24)-N(4)	33.9(11)
C(22)-C(23B)-C(24)-N(4)	-23.3(18)
C(6)-C(1)-N(1)-P(1)	-102.9(4)
C(2)-C(1)-N(1)-P(1)	81.5(4)
C(15)-C(16)-N(2)-C(13)	1.1(8)
C(15)-C(16)-N(2)-P(1)	-177.1(6)
C(14A)-C(13)-N(2)-C(16)	12.7(11)
C(14B)-C(13)-N(2)-C(16)	-18.8(11)
C(14A)-C(13)-N(2)-P(1)	-169.0(10)
C(14B)-C(13)-N(2)-P(1)	159.5(11)

C(18B)-C(17)-N(3)-C(20)	-6(2)
C(18A)-C(17)-N(3)-C(20)	15.6(14)
C(18B)-C(17)-N(3)-P(1)	167.9(19)
C(18A)-C(17)-N(3)-P(1)	-170.3(13)
C(19)-C(20)-N(3)-C(17)	-15.9(5)
C(19)-C(20)-N(3)-P(1)	170.0(3)
C(22)-C(21)-N(4)-C(24)	-4.3(5)
C(22)-C(21)-N(4)-P(1)	-179.1(3)
C(23A)-C(24)-N(4)-C(21)	-18.7(9)
C(23B)-C(24)-N(4)-C(21)	17.3(13)
C(23A)-C(24)-N(4)-P(1)	156.0(8)
C(23B)-C(24)-N(4)-P(1)	-168.0(12)
C(1)-N(1)-P(1)-N(4)	148.9(3)
C(1)-N(1)-P(1)-N(3)	27.5(4)
C(1)-N(1)-P(1)-N(2)	-95.8(4)
C(21)-N(4)-P(1)-N(1)	-31.1(4)
C(24)-N(4)-P(1)-N(1)	154.9(4)
C(21)-N(4)-P(1)-N(3)	94.2(3)
C(24)-N(4)-P(1)-N(3)	-79.8(4)
C(21)-N(4)-P(1)-N(2)	-155.7(3)
C(24)-N(4)-P(1)-N(2)	30.2(4)
C(17)-N(3)-P(1)-N(1)	143.7(4)
C(20)-N(3)-P(1)-N(1)	-43.1(4)
C(17)-N(3)-P(1)-N(4)	22.5(4)
C(20)-N(3)-P(1)-N(4)	-164.3(3)
C(17)-N(3)-P(1)-N(2)	-86.3(4)
C(20)-N(3)-P(1)-N(2)	86.9(4)
C(16)-N(2)-P(1)-N(1)	125.5(5)
C(13)-N(2)-P(1)-N(1)	-52.5(4)
C(16)-N(2)-P(1)-N(4)	-116.1(5)
C(13)-N(2)-P(1)-N(4)	66.0(4)
C(16)-N(2)-P(1)-N(3)	-3.0(5)
C(13)-N(2)-P(1)-N(3)	179.0(3)

Symmetry transformations used to generate equivalent atoms:

Compound **3'**:

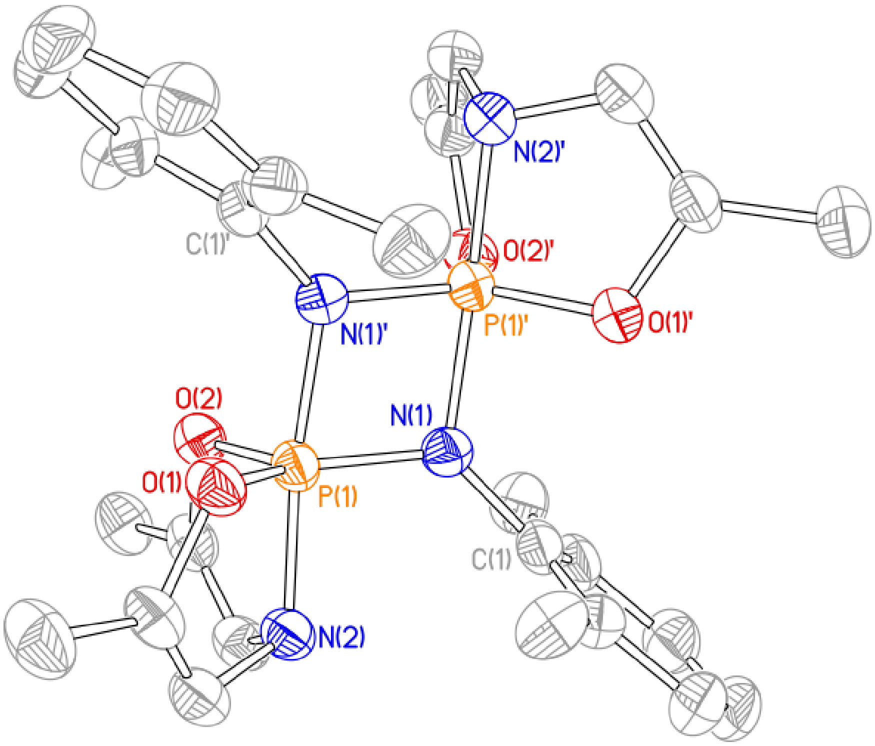

Table S25. Crystal data and structure refinement for **3'**.

Identification code	yzl5s	
Empirical formula	C48 H74 N4 O4 P2	
Formula weight	833.08	
Temperature	293(2) K	
Wavelength	0.71073 Å	
Crystal system	Monoclinic	
Space group	C2/c	
Unit cell dimensions	a = 27.535(6) Å	α= 90°.
	b = 11.390(2) Å	β= 129.45(3)°.
	c = 20.157(4) Å	γ = 90°.
Volume	4882(2) $Å^3$	
Z	4	
Density (calculated)	1.133 Mg/m^3	
Absorption coefficient	0.133 mm^{-1}	
F(000)	1808	
Crystal size	0.21 x 0.18 x 0.15 mm^3	
Theta range for data collection	1.916 to 24.999°.	
Index ranges	-28<=h<=32, -13<=k<=13, -23<=l<=23	
Reflections collected	19206	
Independent reflections	4252 [R(int) = 0.0450]	
Completeness to theta = 24.999°	99.0 %	
Absorption correction	Semi-empirical from equivalents	
Max. and min. transmission	0.9803 and 0.9726	
Refinement method	Full-matrix least-squares on F^2	
Data / restraints / parameters	4252 / 0 / 262	
Goodness-of-fit on F^2	1.111	
Final R indices [I>2sigma(I)]	R1 = 0.0715, wR2 = 0.1978	
R indices (all data)	R1 = 0.0838, wR2 = 0.2046	
Extinction coefficient	n/a	
Largest diff. peak and hole	0.438 and -0.259 e.$Å^{-3}$	

Table S26. Atomic coordinates (x 10^4) and equivalent isotropic displacement parameters (Å^{2}x 10^3) for **3'**. U(eq) is defined as one third of the trace of the orthogonalized U^{ij} tensor.

	x	y	z	U(eq)
C(1)	-758(2)	9779(3)	5890(2)	34(1)
C(2)	-1159(2)	8930(3)	5275(2)	40(1)
C(3)	-1622(2)	9295(4)	4424(2)	51(1)
C(4)	-1684(2)	10436(4)	4184(2)	54(1)
C(5)	-1265(2)	11256(4)	4789(2)	50(1)
C(6)	-796(2)	10951(3)	5643(2)	39(1)
C(7)	-339(2)	11914(3)	6243(2)	48(1)
C(8)	-663(3)	12909(4)	6333(4)	73(1)
C(9)	16(2)	12402(4)	5948(3)	64(1)
C(10)	-1116(2)	7632(4)	5466(2)	48(1)
C(11)	-1716(2)	7209(5)	5291(3)	73(1)
C(12)	-996(3)	6899(4)	4944(3)	74(1)
C(13)	1083(2)	10191(3)	6977(2)	41(1)
C(14)	1354(2)	10656(3)	7741(2)	39(1)
C(15)	1961(2)	11313(4)	8350(3)	53(1)
C(16)	1849(3)	12607(6)	8351(6)	140(3)
C(17)	2296(3)	10847(8)	9242(4)	142(4)
C(18)	2353(3)	11157(8)	8074(5)	138(4)
C(19)	587(2)	8373(3)	6444(2)	40(1)
C(20)	724(2)	7589(3)	7023(2)	38(1)
C(21)	920(2)	6324(3)	7131(3)	48(1)
C(22)	591(3)	5572(4)	7372(4)	71(1)
C(23)	770(2)	5860(4)	6310(3)	68(1)
C(24)	1638(2)	6259(5)	7855(3)	73(1)
N(1)	-322(1)	9462(2)	6788(2)	31(1)
N(2)	531(1)	9540(2)	6644(2)	34(1)
O(1)	1012(1)	10406(2)	8028(2)	36(1)
O(2)	739(1)	8060(2)	7686(1)	36(1)
P(1)	460(1)	9412(1)	7460(1)	29(1)

Table S27. Bond lengths [Å] and angles [°] for **3'**.

C(1)-C(2)	1.398(5)
C(1)-C(6)	1.405(5)
C(1)-N(1)	1.444(4)
C(2)-C(3)	1.401(5)
C(2)-C(10)	1.513(5)
C(3)-C(4)	1.358(6)
C(3)-H(3)	0.9300
C(4)-C(5)	1.380(6)
C(4)-H(4)	0.9300
C(5)-C(6)	1.389(5)
C(5)-H(5)	0.9300
C(6)-C(7)	1.522(5)
C(7)-C(8)	1.521(6)
C(7)-C(9)	1.535(6)
C(7)-H(7)	0.9800
C(8)-H(8A)	0.9600
C(8)-H(8B)	0.9600
C(8)-H(8C)	0.9600
C(9)-H(9A)	0.9600
C(9)-H(9B)	0.9600
C(9)-H(9C)	0.9600
C(10)-C(11)	1.530(6)
C(10)-C(12)	1.537(6)
C(10)-H(10)	0.9800
C(11)-H(11A)	0.9600
C(11)-H(11B)	0.9600
C(11)-H(11C)	0.9600
C(12)-H(12A)	0.9600
C(12)-H(12B)	0.9600
C(12)-H(12C)	0.9600
C(13)-C(14)	1.321(5)
C(13)-N(2)	1.418(4)
C(13)-H(13)	0.9300
C(14)-O(1)	1.413(4)

C(14)-C(15)	1.502(5)
C(15)-C(17)	1.502(8)
C(15)-C(16)	1.506(8)
C(15)-C(18)	1.509(7)
C(16)-H(16A)	0.9600
C(16)-H(16B)	0.9600
C(16)-H(16C)	0.9600
C(17)-H(17A)	0.9600
C(17)-H(17B)	0.9600
C(17)-H(17C)	0.9600
C(18)-H(18A)	0.9600
C(18)-H(18B)	0.9600
C(18)-H(18C)	0.9600
C(19)-C(20)	1.321(5)
C(19)-N(2)	1.425(4)
C(19)-H(19)	0.9300
C(20)-O(2)	1.415(4)
C(20)-C(21)	1.505(5)
C(21)-C(23)	1.525(6)
C(21)-C(22)	1.531(6)
C(21)-C(24)	1.541(6)
C(22)-H(22A)	0.9600
C(22)-H(22B)	0.9600
C(22)-H(22C)	0.9600
C(23)-H(23A)	0.9600
C(23)-H(23B)	0.9600
C(23)-H(23C)	0.9600
C(24)-H(24A)	0.9600
C(24)-H(24B)	0.9600
C(24)-H(24C)	0.9600
N(1)-P(1)	1.665(3)
N(1)-P(1)#1	1.783(3)
N(2)-P(1)	1.782(3)
O(1)-P(1)	1.640(2)
O(2)-P(1)	1.651(2)
P(1)-P(1)#1	2.6392(17)

C(2)-C(1)-C(6)	120.2(3)
C(2)-C(1)-N(1)	119.9(3)
C(6)-C(1)-N(1)	119.8(3)
C(1)-C(2)-C(3)	118.2(4)
C(1)-C(2)-C(10)	124.1(3)
C(3)-C(2)-C(10)	117.7(3)
C(4)-C(3)-C(2)	122.1(4)
C(4)-C(3)-H(3)	119.0
C(2)-C(3)-H(3)	119.0
C(3)-C(4)-C(5)	119.2(4)
C(3)-C(4)-H(4)	120.4
C(5)-C(4)-H(4)	120.4
C(4)-C(5)-C(6)	121.5(4)
C(4)-C(5)-H(5)	119.2
C(6)-C(5)-H(5)	119.2
C(5)-C(6)-C(1)	118.6(3)
C(5)-C(6)-C(7)	116.9(3)
C(1)-C(6)-C(7)	124.4(3)
C(8)-C(7)-C(6)	112.3(4)
C(8)-C(7)-C(9)	110.0(4)
C(6)-C(7)-C(9)	111.2(3)
C(8)-C(7)-H(7)	107.7
C(6)-C(7)-H(7)	107.7
C(9)-C(7)-H(7)	107.7
C(7)-C(8)-H(8A)	109.5
C(7)-C(8)-H(8B)	109.5
H(8A)-C(8)-H(8B)	109.5
C(7)-C(8)-H(8C)	109.5
H(8A)-C(8)-H(8C)	109.5
H(8B)-C(8)-H(8C)	109.5
C(7)-C(9)-H(9A)	109.5
C(7)-C(9)-H(9B)	109.5
H(9A)-C(9)-H(9B)	109.5
C(7)-C(9)-H(9C)	109.5
H(9A)-C(9)-H(9C)	109.5

H(9B)-C(9)-H(9C)	109.5
C(2)-C(10)-C(11)	110.6(4)
C(2)-C(10)-C(12)	111.8(4)
C(11)-C(10)-C(12)	110.0(4)
C(2)-C(10)-H(10)	108.1
C(11)-C(10)-H(10)	108.1
C(12)-C(10)-H(10)	108.1
C(10)-C(11)-H(11A)	109.5
C(10)-C(11)-H(11B)	109.5
H(11A)-C(11)-H(11B)	109.5
C(10)-C(11)-H(11C)	109.5
H(11A)-C(11)-H(11C)	109.5
H(11B)-C(11)-H(11C)	109.5
C(10)-C(12)-H(12A)	109.5
C(10)-C(12)-H(12B)	109.5
H(12A)-C(12)-H(12B)	109.5
C(10)-C(12)-H(12C)	109.5
H(12A)-C(12)-H(12C)	109.5
H(12B)-C(12)-H(12C)	109.5
C(14)-C(13)-N(2)	113.8(3)
C(14)-C(13)-H(13)	123.1
N(2)-C(13)-H(13)	123.1
C(13)-C(14)-O(1)	111.5(3)
C(13)-C(14)-C(15)	131.6(3)
O(1)-C(14)-C(15)	116.8(3)
C(14)-C(15)-C(17)	108.8(4)
C(14)-C(15)-C(16)	111.5(4)
C(17)-C(15)-C(16)	108.4(6)
C(14)-C(15)-C(18)	109.0(4)
C(17)-C(15)-C(18)	111.1(6)
C(16)-C(15)-C(18)	108.0(5)
C(15)-C(16)-H(16A)	109.5
C(15)-C(16)-H(16B)	109.5
H(16A)-C(16)-H(16B)	109.5
C(15)-C(16)-H(16C)	109.5
H(16A)-C(16)-H(16C)	109.5

H(16B)-C(16)-H(16C)	109.5
C(15)-C(17)-H(17A)	109.5
C(15)-C(17)-H(17B)	109.5
H(17A)-C(17)-H(17B)	109.5
C(15)-C(17)-H(17C)	109.5
H(17A)-C(17)-H(17C)	109.5
H(17B)-C(17)-H(17C)	109.5
C(15)-C(18)-H(18A)	109.5
C(15)-C(18)-H(18B)	109.5
H(18A)-C(18)-H(18B)	109.5
C(15)-C(18)-H(18C)	109.5
H(18A)-C(18)-H(18C)	109.5
H(18B)-C(18)-H(18C)	109.5
C(20)-C(19)-N(2)	113.7(3)
C(20)-C(19)-H(19)	123.2
N(2)-C(19)-H(19)	123.2
C(19)-C(20)-O(2)	113.5(3)
C(19)-C(20)-C(21)	130.5(3)
O(2)-C(20)-C(21)	115.7(3)
C(20)-C(21)-C(23)	110.5(3)
C(20)-C(21)-C(22)	110.6(3)
C(23)-C(21)-C(22)	110.1(4)
C(20)-C(21)-C(24)	108.1(3)
C(23)-C(21)-C(24)	108.0(4)
C(22)-C(21)-C(24)	109.6(4)
C(21)-C(22)-H(22A)	109.5
C(21)-C(22)-H(22B)	109.5
H(22A)-C(22)-H(22B)	109.5
C(21)-C(22)-H(22C)	109.5
H(22A)-C(22)-H(22C)	109.5
H(22B)-C(22)-H(22C)	109.5
C(21)-C(23)-H(23A)	109.5
C(21)-C(23)-H(23B)	109.5
H(23A)-C(23)-H(23B)	109.5
C(21)-C(23)-H(23C)	109.5
H(23A)-C(23)-H(23C)	109.5

H(23B)-C(23)-H(23C)	109.5
C(21)-C(24)-H(24A)	109.5
C(21)-C(24)-H(24B)	109.5
H(24A)-C(24)-H(24B)	109.5
C(21)-C(24)-H(24C)	109.5
H(24A)-C(24)-H(24C)	109.5
H(24B)-C(24)-H(24C)	109.5
C(1)-N(1)-P(1)	130.1(2)
C(1)-N(1)-P(1)#1	128.1(2)
P(1)-N(1)-P(1)#1	99.87(14)
C(13)-N(2)-C(19)	110.3(3)
C(13)-N(2)-P(1)	108.5(2)
C(19)-N(2)-P(1)	106.3(2)
C(14)-O(1)-P(1)	114.5(2)
C(20)-O(2)-P(1)	111.3(2)
O(1)-P(1)-O(2)	112.90(13)
O(1)-P(1)-N(1)	133.63(13)
O(2)-P(1)-N(1)	113.05(13)
O(1)-P(1)-N(2)	89.56(12)
O(2)-P(1)-N(2)	91.45(12)
N(1)-P(1)-N(2)	95.17(13)
O(1)-P(1)-N(1)#1	89.26(12)
O(2)-P(1)-N(1)#1	96.25(12)
N(1)-P(1)-N(1)#1	80.00(14)
N(2)-P(1)-N(1)#1	172.04(13)
O(1)-P(1)-P(1)#1	117.15(9)
O(2)-P(1)-P(1)#1	106.28(8)
N(1)-P(1)-P(1)#1	41.71(9)
N(2)-P(1)-P(1)#1	136.83(11)
N(1)#1-P(1)-P(1)#1	38.42(9)

Symmetry transformations used to generate equivalent atoms:

#1 -x,y,-z+3/2

Table S28. Anisotropic displacement parameters ($Å^2 x 10^3$) for **3'**. The anisotropic displacement factor exponent takes the form: $-2\pi^2[h^2 a^{*2}U^{11} + ... + 2 h k a^* b^* U^{12}]$

	U^{11}	U^{22}	U^{33}	U^{23}	U^{13}	U^{12}
C(1)	26(2)	44(2)	32(2)	-1(2)	18(2)	3(1)
C(2)	24(2)	55(2)	35(2)	-5(2)	17(2)	-1(2)
C(3)	35(2)	74(3)	33(2)	-7(2)	16(2)	-1(2)
C(4)	40(2)	82(3)	29(2)	6(2)	16(2)	15(2)
C(5)	46(2)	58(2)	41(2)	13(2)	25(2)	14(2)
C(6)	36(2)	48(2)	34(2)	3(2)	24(2)	5(2)
C(7)	55(2)	41(2)	40(2)	4(2)	27(2)	0(2)
C(8)	92(4)	51(3)	96(4)	-5(3)	69(3)	0(3)
C(9)	60(3)	61(3)	70(3)	7(2)	41(3)	-4(2)
C(10)	40(2)	54(2)	37(2)	-10(2)	19(2)	-10(2)
C(11)	56(3)	88(4)	64(3)	-9(3)	33(3)	-29(3)
C(12)	79(3)	66(3)	68(3)	-22(2)	42(3)	-3(3)
C(13)	38(2)	46(2)	48(2)	0(2)	31(2)	-2(2)
C(14)	33(2)	41(2)	49(2)	-3(2)	29(2)	-5(2)
C(15)	39(2)	61(3)	59(3)	-13(2)	32(2)	-14(2)
C(16)	78(4)	73(4)	232(9)	-58(5)	82(6)	-40(3)
C(17)	62(4)	208(9)	70(4)	11(5)	2(3)	-60(5)
C(18)	79(4)	227(9)	148(6)	-115(6)	90(5)	-95(5)
C(19)	38(2)	47(2)	40(2)	-10(2)	27(2)	-6(2)
C(20)	34(2)	41(2)	42(2)	-7(2)	26(2)	-1(2)
C(21)	48(2)	43(2)	60(2)	-4(2)	37(2)	3(2)
C(22)	91(4)	38(2)	110(4)	1(2)	75(3)	-1(2)
C(23)	80(3)	54(3)	78(3)	-14(2)	55(3)	6(2)
C(24)	56(3)	71(3)	82(3)	3(3)	39(3)	20(2)
N(1)	30(1)	35(2)	28(1)	-1(1)	19(1)	-1(1)
N(2)	31(2)	40(2)	33(2)	-2(1)	22(1)	-4(1)
O(1)	32(1)	42(1)	37(1)	-6(1)	24(1)	-8(1)
O(2)	35(1)	37(1)	38(1)	1(1)	25(1)	4(1)
P(1)	25(1)	32(1)	30(1)	-1(1)	17(1)	0(1)

Table S29. Hydrogen coordinates (x 10^4) and isotropic displacement parameters ($Å^2$x 10^3) for **3'**.

	x	y	z	U(eq)
H(3)	-1896	8740	4010	62
H(4)	-2006	10661	3619	65
H(5)	-1297	12031	4620	60
H(7)	-29	11565	6813	57
H(8A)	-358	13494	6716	110
H(8B)	-978	13254	5780	110
H(8C)	-856	12607	6560	110
H(9A)	219	11770	5893	97
H(9B)	-276	12785	5403	97
H(9C)	326	12957	6364	97
H(10)	-763	7512	6077	57
H(11A)	-1681	6386	5416	110
H(11B)	-1772	7634	5650	110
H(11C)	-2071	7343	4699	110
H(12A)	-972	6083	5081	112
H(12B)	-1334	7015	4341	112
H(12C)	-607	7139	5082	112
H(13)	1239	10284	6685	49
H(16A)	2245	13001	8746	210
H(16B)	1633	12918	7784	210
H(16C)	1598	12726	8523	210
H(17A)	2685	11262	9637	213
H(17B)	2037	10956	9402	213
H(17C)	2381	10026	9256	213
H(18A)	2742	11576	8461	207
H(18B)	2439	10338	8082	207
H(18C)	2128	11459	7502	207
H(19)	533	8182	5952	47
H(22A)	722	4770	7438	106
H(22B)	702	5851	7902	106

H(22C)	144	5625	6925	106
H(23A)	898	5054	6389	102
H(23B)	326	5918	5845	102
H(23C)	993	6316	6178	102
H(24A)	1774	5458	7935	109
H(24B)	1844	6725	7701	109
H(24C)	1743	6553	8378	109

Table S30. Torsion angles [°] for **3'.**

C(6)-C(1)-C(2)-C(3)	-4.0(5)
N(1)-C(1)-C(2)-C(3)	173.0(3)
C(6)-C(1)-C(2)-C(10)	174.6(3)
N(1)-C(1)-C(2)-C(10)	-8.4(5)
C(1)-C(2)-C(3)-C(4)	0.8(6)
C(10)-C(2)-C(3)-C(4)	-177.9(4)
C(2)-C(3)-C(4)-C(5)	2.4(6)
C(3)-C(4)-C(5)-C(6)	-2.6(6)
C(4)-C(5)-C(6)-C(1)	-0.5(6)
C(4)-C(5)-C(6)-C(7)	176.8(4)
C(2)-C(1)-C(6)-C(5)	3.9(5)
N(1)-C(1)-C(6)-C(5)	-173.1(3)
C(2)-C(1)-C(6)-C(7)	-173.3(3)
N(1)-C(1)-C(6)-C(7)	9.7(5)
C(5)-C(6)-C(7)-C(8)	63.8(5)
C(1)-C(6)-C(7)-C(8)	-119.0(4)
C(5)-C(6)-C(7)-C(9)	-59.9(5)
C(1)-C(6)-C(7)-C(9)	117.3(4)
C(1)-C(2)-C(10)-C(11)	117.2(4)
C(3)-C(2)-C(10)-C(11)	-64.2(5)
C(1)-C(2)-C(10)-C(12)	-119.9(4)

C(3)-C(2)-C(10)-C(12)	58.8(5)
N(2)-C(13)-C(14)-O(1)	-1.5(4)
N(2)-C(13)-C(14)-C(15)	174.3(4)
C(13)-C(14)-C(15)-C(17)	-135.1(6)
O(1)-C(14)-C(15)-C(17)	40.5(6)
C(13)-C(14)-C(15)-C(16)	105.4(6)
O(1)-C(14)-C(15)-C(16)	-79.0(6)
C(13)-C(14)-C(15)-C(18)	-13.8(7)
O(1)-C(14)-C(15)-C(18)	161.8(5)
N(2)-C(19)-C(20)-O(2)	4.4(4)
N(2)-C(19)-C(20)-C(21)	-169.2(3)
C(19)-C(20)-C(21)-C(23)	-16.7(6)
O(2)-C(20)-C(21)-C(23)	169.7(3)
C(19)-C(20)-C(21)-C(22)	-138.9(4)
O(2)-C(20)-C(21)-C(22)	47.6(5)
C(19)-C(20)-C(21)-C(24)	101.2(5)
O(2)-C(20)-C(21)-C(24)	-72.3(4)
C(2)-C(1)-N(1)-P(1)	113.3(3)
C(6)-C(1)-N(1)-P(1)	-69.6(4)
C(2)-C(1)-N(1)-P(1)#1	-85.8(4)
C(6)-C(1)-N(1)-P(1)#1	91.3(4)
C(14)-C(13)-N(2)-C(19)	-124.0(3)
C(14)-C(13)-N(2)-P(1)	-8.0(4)
C(20)-C(19)-N(2)-C(13)	102.0(3)
C(20)-C(19)-N(2)-P(1)	-15.5(4)
C(13)-C(14)-O(1)-P(1)	11.6(4)
C(15)-C(14)-O(1)-P(1)	-164.9(3)
C(19)-C(20)-O(2)-P(1)	10.0(4)
C(21)-C(20)-O(2)-P(1)	-175.3(2)
C(14)-O(1)-P(1)-O(2)	77.9(2)
C(14)-O(1)-P(1)-N(1)	-110.2(3)
C(14)-O(1)-P(1)-N(2)	-13.5(2)
C(14)-O(1)-P(1)-N(1)#1	174.4(2)
C(14)-O(1)-P(1)-P(1)#1	-158.2(2)
C(20)-O(2)-P(1)-O(1)	-106.2(2)
C(20)-O(2)-P(1)-N(1)	80.2(2)

C(20)-O(2)-P(1)-N(2)	-16.0(2)
C(20)-O(2)-P(1)-N(1)#1	162.0(2)
C(20)-O(2)-P(1)-P(1)#1	124.08(19)
C(1)-N(1)-P(1)-O(1)	81.9(3)
P(1)#1-N(1)-P(1)-O(1)	-82.98(19)
C(1)-N(1)-P(1)-O(2)	-106.3(3)
P(1)#1-N(1)-P(1)-O(2)	88.88(15)
C(1)-N(1)-P(1)-N(2)	-12.5(3)
P(1)#1-N(1)-P(1)-N(2)	-177.32(13)
C(1)-N(1)-P(1)-N(1)#1	161.1(2)
P(1)#1-N(1)-P(1)-N(1)#1	-3.71(18)
C(1)-N(1)-P(1)-P(1)#1	164.8(4)
C(13)-N(2)-P(1)-O(1)	12.0(2)
C(19)-N(2)-P(1)-O(1)	130.6(2)
C(13)-N(2)-P(1)-O(2)	-100.9(2)
C(19)-N(2)-P(1)-O(2)	17.7(2)
C(13)-N(2)-P(1)-N(1)	145.8(2)
C(19)-N(2)-P(1)-N(1)	-95.6(2)
C(13)-N(2)-P(1)-P(1)#1	143.22(18)
C(19)-N(2)-P(1)-P(1)#1	-98.2(2)

Symmetry transformations used to generate equivalent atoms:

#1 -x,y,-z+3/2

Compound **9'**:

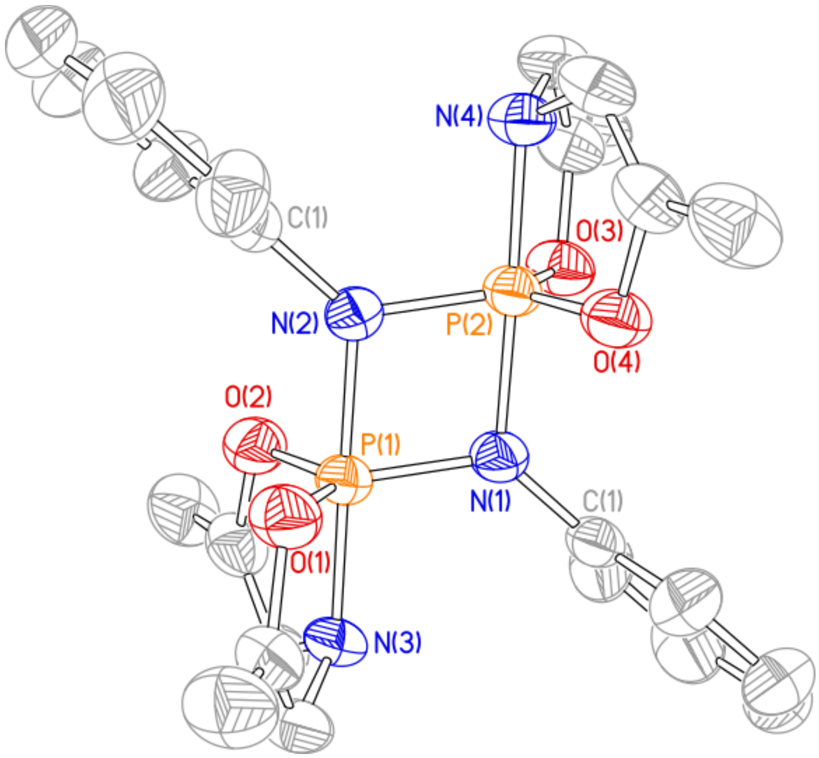

Table S31. Crystal data and structure refinement for **9'**.

Identification code	yzl1s	
Empirical formula	C38 H54 N4 O4 P2	
Formula weight	692.79	
Temperature	298(2) K	
Wavelength	0.71073 Å	
Crystal system	Monoclinic	
Space group	$P2_1/n$	
Unit cell dimensions	a = 9.2449(12) Å	α= 90°.
	b = 21.052(3) Å	β= 94.271(2)°.
	c = 20.977(3) Å	γ = 90°.
Volume	4071.3(10) $Å^3$	
Z	4	
Density (calculated)	1.130 Mg/m^3	
Absorption coefficient	0.147 mm^{-1}	
F(000)	1488	
Crystal size	0.320 x 0.260 x 0.210 mm^3	
Theta range for data collection	1.935 to 24.999°.	
Index ranges	-10<=h<=10, -24<=k<=25, -24<=l<=23	
Reflections collected	23546	
Independent reflections	7120 [R(int) = 0.0295]	
Completeness to theta = 24.999°	99.6 %	
Absorption correction	Semi-empirical from equivalents	
Max. and min. transmission	0.9697 and 0.9544	
Refinement method	Full-matrix least-squares on F^2	
Data / restraints / parameters	7120 / 0 / 446	
Goodness-of-fit on F^2	1.157	
Final R indices [I>2sigma(I)]	R1 = 0.0749, wR2 = 0.2038	
R indices (all data)	R1 = 0.0902, wR2 = 0.2112	
Extinction coefficient	n/a	
Largest diff. peak and hole	0.620 and -0.315 e.$Å^{-3}$	

Table S32. Atomic coordinates (x 10^4) and equivalent isotropic displacement parameters (Å^{2}x 10^3) for **9'**. U(eq) is defined as one third of the trace of the orthogonalized U^{ij} tensor.

	x	y	z	U(eq)
C(1)	8480(5)	568(2)	3044(2)	51(1)
C(2)	7412(5)	318(2)	3342(2)	49(1)
C(3)	7363(6)	-19(3)	3969(2)	68(1)
C(4)	6435(13)	-600(4)	3884(4)	187(6)
C(5)	6734(11)	409(5)	4447(4)	157(4)
C(6)	8879(8)	-206(5)	4217(4)	143(4)
C(7)	8589(5)	764(2)	1897(2)	50(1)
C(8)	7578(5)	630(2)	1442(2)	51(1)
C(9)	7631(6)	504(3)	739(2)	68(1)
C(10)	7216(9)	-183(4)	604(3)	118(3)
C(11)	6569(7)	940(4)	358(3)	101(2)
C(12)	9169(7)	632(4)	546(3)	106(2)
C(13)	6757(4)	2169(2)	2596(2)	41(1)
C(14)	7032(5)	2422(2)	3200(2)	55(1)
C(15)	7960(6)	2934(3)	3294(3)	72(2)
C(16)	8638(6)	3196(2)	2809(3)	72(2)
C(17)	8386(6)	2935(3)	2211(3)	76(2)
C(18)	7437(5)	2431(2)	2098(3)	59(1)
C(19)	9693(8)	3753(3)	2909(5)	126(3)
C(20)	3216(4)	363(2)	2249(2)	40(1)
C(21)	2842(5)	84(2)	1664(2)	58(1)
C(22)	1910(6)	-430(3)	1629(3)	76(2)
C(23)	1339(6)	-672(2)	2159(4)	75(2)
C(24)	1718(6)	-396(3)	2727(3)	74(2)
C(25)	2660(5)	120(2)	2781(2)	60(1)
C(26)	287(8)	-1234(3)	2108(5)	133(3)
C(27)	1493(5)	1979(2)	1756(2)	49(1)
C(28)	2563(5)	2234(2)	1463(2)	49(1)
C(29)	2624(6)	2587(3)	844(3)	69(2)
C(30)	3315(10)	2159(4)	366(3)	128(3)
C(31)	3505(10)	3185(3)	940(4)	130(3)

C(32)	1083(8)	2739(5)	585(4)	152(4)
C(33)	1388(4)	1766(2)	2894(2)	48(1)
C(34)	2383(5)	1895(2)	3354(2)	48(1)
C(35)	2340(6)	2005(3)	4056(2)	65(1)
C(36)	2961(10)	2657(3)	4230(3)	117(3)
C(37)	764(7)	1959(5)	4234(3)	140(4)
C(38)	3236(7)	1508(3)	4426(3)	89(2)
N(1)	5776(3)	1645(2)	2490(2)	38(1)
N(2)	4206(3)	890(2)	2303(2)	39(1)
N(3)	7988(3)	896(2)	2481(2)	42(1)
N(4)	1971(3)	1636(2)	2312(2)	41(1)
O(1)	6061(3)	428(1)	3019(1)	48(1)
O(2)	6170(3)	625(1)	1659(1)	47(1)
O(3)	3912(3)	2129(1)	1789(1)	45(1)
O(4)	3791(3)	1900(1)	3139(1)	45(1)
P(1)	6096(1)	889(1)	2392(1)	35(1)
P(2)	3878(1)	1650(1)	2405(1)	35(1)

Table S33. Bond lengths [Å] and angles [°] for **9'.**

C(1)-C(2)	1.318(6)
C(1)-N(3)	1.414(5)
C(1)-H(1)	0.9300
C(2)-O(1)	1.395(5)
C(2)-C(3)	1.498(7)
C(3)-C(4)	1.497(9)
C(3)-C(5)	1.498(9)
C(3)-C(6)	1.510(8)
C(4)-H(4A)	0.9600
C(4)-H(4B)	0.9600

C(4)-H(4C)	0.9600
C(5)-H(5A)	0.9600
C(5)-H(5B)	0.9600
C(5)-H(5C)	0.9600
C(6)-H(6A)	0.9600
C(6)-H(6B)	0.9600
C(6)-H(6C)	0.9600
C(7)-C(8)	1.315(6)
C(7)-N(3)	1.410(5)
C(7)-H(7)	0.9300
C(8)-O(2)	1.411(5)
C(8)-C(9)	1.504(7)
C(9)-C(10)	1.519(9)
C(9)-C(11)	1.525(8)
C(9)-C(12)	1.531(8)
C(10)-H(10A)	0.9600
C(10)-H(10B)	0.9600
C(10)-H(10C)	0.9600
C(11)-H(11A)	0.9600
C(11)-H(11B)	0.9600
C(11)-H(11C)	0.9600
C(12)-H(12A)	0.9600
C(12)-H(12B)	0.9600
C(12)-H(12C)	0.9600
C(13)-C(18)	1.375(6)
C(13)-C(14)	1.381(6)
C(13)-N(1)	1.435(5)
C(14)-C(15)	1.382(7)
C(14)-H(14)	0.9300
C(15)-C(16)	1.353(8)
C(15)-H(15)	0.9300
C(16)-C(17)	1.372(8)
C(16)-C(19)	1.529(7)
C(17)-C(18)	1.385(7)
C(17)-H(17)	0.9300
C(18)-H(18)	0.9300

C(19)-H(19A)	0.9600
C(19)-H(19B)	0.9600
C(19)-H(19C)	0.9600
C(20)-C(25)	1.362(6)
C(20)-C(21)	1.382(6)
C(20)-N(2)	1.436(5)
C(21)-C(22)	1.380(7)
C(21)-H(21)	0.9300
C(22)-C(23)	1.365(8)
C(22)-H(22)	0.9300
C(23)-C(24)	1.349(8)
C(23)-C(26)	1.531(7)
C(24)-C(25)	1.391(7)
C(24)-H(24)	0.9300
C(25)-H(25)	0.9300
C(26)-H(26A)	0.9600
C(26)-H(26B)	0.9600
C(26)-H(26C)	0.9600
C(27)-C(28)	1.318(6)
C(27)-N(4)	1.413(5)
C(27)-H(27)	0.9300
C(28)-O(3)	1.395(5)
C(28)-C(29)	1.499(6)
C(29)-C(31)	1.505(9)
C(29)-C(32)	1.520(8)
C(29)-C(30)	1.524(9)
C(30)-H(30A)	0.9600
C(30)-H(30B)	0.9600
C(30)-H(30C)	0.9600
C(31)-H(31A)	0.9600
C(31)-H(31B)	0.9600
C(31)-H(31C)	0.9600
C(32)-H(32A)	0.9600
C(32)-H(32B)	0.9600
C(32)-H(32C)	0.9600
C(33)-C(34)	1.310(6)

C(33)-N(4)	1.400(5)
C(33)-H(33)	0.9300
C(34)-O(4)	1.409(5)
C(34)-C(35)	1.496(6)
C(35)-C(38)	1.511(8)
C(35)-C(36)	1.521(9)
C(35)-C(37)	1.533(8)
C(36)-H(36A)	0.9600
C(36)-H(36B)	0.9600
C(36)-H(36C)	0.9600
C(37)-H(37A)	0.9600
C(37)-H(37B)	0.9600
C(37)-H(37C)	0.9600
C(38)-H(38A)	0.9600
C(38)-H(38B)	0.9600
C(38)-H(38C)	0.9600
N(1)-P(1)	1.635(3)
N(1)-P(2)	1.751(3)
N(2)-P(2)	1.646(3)
N(2)-P(1)	1.744(3)
N(3)-P(1)	1.746(3)
N(4)-P(2)	1.759(3)
O(1)-P(1)	1.637(3)
O(2)-P(1)	1.641(3)
O(3)-P(2)	1.641(3)
O(4)-P(2)	1.636(3)
C(2)-C(1)-N(3)	112.7(4)
C(2)-C(1)-H(1)	123.7
N(3)-C(1)-H(1)	123.7
C(1)-C(2)-O(1)	112.2(4)
C(1)-C(2)-C(3)	132.8(4)
O(1)-C(2)-C(3)	114.9(4)
C(4)-C(3)-C(5)	109.0(7)
C(4)-C(3)-C(2)	109.7(5)
C(5)-C(3)-C(2)	109.9(5)
C(4)-C(3)-C(6)	109.7(7)

C(5)-C(3)-C(6)	108.9(6)
C(2)-C(3)-C(6)	109.7(5)
C(3)-C(4)-H(4A)	109.5
C(3)-C(4)-H(4B)	109.5
H(4A)-C(4)-H(4B)	109.5
C(3)-C(4)-H(4C)	109.5
H(4A)-C(4)-H(4C)	109.5
H(4B)-C(4)-H(4C)	109.5
C(3)-C(5)-H(5A)	109.5
C(3)-C(5)-H(5B)	109.5
H(5A)-C(5)-H(5B)	109.5
C(3)-C(5)-H(5C)	109.5
H(5A)-C(5)-H(5C)	109.5
H(5B)-C(5)-H(5C)	109.5
C(3)-C(6)-H(6A)	109.5
C(3)-C(6)-H(6B)	109.5
H(6A)-C(6)-H(6B)	109.5
C(3)-C(6)-H(6C)	109.5
H(6A)-C(6)-H(6C)	109.5
H(6B)-C(6)-H(6C)	109.5
C(8)-C(7)-N(3)	111.6(4)
C(8)-C(7)-H(7)	124.2
N(3)-C(7)-H(7)	124.2
C(7)-C(8)-O(2)	113.0(4)
C(7)-C(8)-C(9)	132.6(4)
O(2)-C(8)-C(9)	114.4(4)
C(8)-C(9)-C(10)	108.9(5)
C(8)-C(9)-C(11)	110.0(4)
C(10)-C(9)-C(11)	109.3(6)
C(8)-C(9)-C(12)	109.1(5)
C(10)-C(9)-C(12)	110.2(5)
C(11)-C(9)-C(12)	109.3(5)
C(9)-C(10)-H(10A)	109.5
C(9)-C(10)-H(10B)	109.5
H(10A)-C(10)-H(10B)	109.5
C(9)-C(10)-H(10C)	109.5

H(10A)-C(10)-H(10C)	109.5
H(10B)-C(10)-H(10C)	109.5
C(9)-C(11)-H(11A)	109.5
C(9)-C(11)-H(11B)	109.5
H(11A)-C(11)-H(11B)	109.5
C(9)-C(11)-H(11C)	109.5
H(11A)-C(11)-H(11C)	109.5
H(11B)-C(11)-H(11C)	109.5
C(9)-C(12)-H(12A)	109.5
C(9)-C(12)-H(12B)	109.5
H(12A)-C(12)-H(12B)	109.5
C(9)-C(12)-H(12C)	109.5
H(12A)-C(12)-H(12C)	109.5
H(12B)-C(12)-H(12C)	109.5
C(18)-C(13)-C(14)	118.6(4)
C(18)-C(13)-N(1)	120.7(4)
C(14)-C(13)-N(1)	120.7(4)
C(13)-C(14)-C(15)	120.1(5)
C(13)-C(14)-H(14)	120.0
C(15)-C(14)-H(14)	120.0
C(16)-C(15)-C(14)	122.0(5)
C(16)-C(15)-H(15)	119.0
C(14)-C(15)-H(15)	119.0
C(15)-C(16)-C(17)	117.8(5)
C(15)-C(16)-C(19)	122.4(6)
C(17)-C(16)-C(19)	119.8(6)
C(16)-C(17)-C(18)	121.8(5)
C(16)-C(17)-H(17)	119.1
C(18)-C(17)-H(17)	119.1
C(13)-C(18)-C(17)	119.7(5)
C(13)-C(18)-H(18)	120.1
C(17)-C(18)-H(18)	120.1
C(16)-C(19)-H(19A)	109.5
C(16)-C(19)-H(19B)	109.5
H(19A)-C(19)-H(19B)	109.5
C(16)-C(19)-H(19C)	109.5

H(19A)-C(19)-H(19C)	109.5
H(19B)-C(19)-H(19C)	109.5
C(25)-C(20)-C(21)	118.8(4)
C(25)-C(20)-N(2)	120.2(4)
C(21)-C(20)-N(2)	120.9(4)
C(22)-C(21)-C(20)	119.7(5)
C(22)-C(21)-H(21)	120.2
C(20)-C(21)-H(21)	120.2
C(23)-C(22)-C(21)	121.8(5)
C(23)-C(22)-H(22)	119.1
C(21)-C(22)-H(22)	119.1
C(24)-C(23)-C(22)	117.9(5)
C(24)-C(23)-C(26)	121.1(6)
C(22)-C(23)-C(26)	121.0(6)
C(23)-C(24)-C(25)	121.8(5)
C(23)-C(24)-H(24)	119.1
C(25)-C(24)-H(24)	119.1
C(20)-C(25)-C(24)	120.0(5)
C(20)-C(25)-H(25)	120.0
C(24)-C(25)-H(25)	120.0
C(23)-C(26)-H(26A)	109.5
C(23)-C(26)-H(26B)	109.5
H(26A)-C(26)-H(26B)	109.5
C(23)-C(26)-H(26C)	109.5
H(26A)-C(26)-H(26C)	109.5
H(26B)-C(26)-H(26C)	109.5
C(28)-C(27)-N(4)	113.2(4)
C(28)-C(27)-H(27)	123.4
N(4)-C(27)-H(27)	123.4
C(27)-C(28)-O(3)	112.3(4)
C(27)-C(28)-C(29)	133.1(4)
O(3)-C(28)-C(29)	114.5(4)
C(28)-C(29)-C(31)	110.7(5)
C(28)-C(29)-C(32)	108.6(5)
C(31)-C(29)-C(32)	110.8(6)
C(28)-C(29)-C(30)	108.7(5)

C(31)-C(29)-C(30)	109.5(6)
C(32)-C(29)-C(30)	108.5(6)
C(29)-C(30)-H(30A)	109.5
C(29)-C(30)-H(30B)	109.5
H(30A)-C(30)-H(30B)	109.5
C(29)-C(30)-H(30C)	109.5
H(30A)-C(30)-H(30C)	109.5
H(30B)-C(30)-H(30C)	109.5
C(29)-C(31)-H(31A)	109.5
C(29)-C(31)-H(31B)	109.5
H(31A)-C(31)-H(31B)	109.5
C(29)-C(31)-H(31C)	109.5
H(31A)-C(31)-H(31C)	109.5
H(31B)-C(31)-H(31C)	109.5
C(29)-C(32)-H(32A)	109.5
C(29)-C(32)-H(32B)	109.5
H(32A)-C(32)-H(32B)	109.5
C(29)-C(32)-H(32C)	109.5
H(32A)-C(32)-H(32C)	109.5
H(32B)-C(32)-H(32C)	109.5
C(34)-C(33)-N(4)	112.8(4)
C(34)-C(33)-H(33)	123.6
N(4)-C(33)-H(33)	123.6
C(33)-C(34)-O(4)	112.5(4)
C(33)-C(34)-C(35)	133.5(4)
O(4)-C(34)-C(35)	114.0(4)
C(34)-C(35)-C(38)	110.1(4)
C(34)-C(35)-C(36)	109.8(5)
C(38)-C(35)-C(36)	108.6(5)
C(34)-C(35)-C(37)	108.9(5)
C(38)-C(35)-C(37)	109.0(5)
C(36)-C(35)-C(37)	110.4(6)
C(35)-C(36)-H(36A)	109.5
C(35)-C(36)-H(36B)	109.5
H(36A)-C(36)-H(36B)	109.5
C(35)-C(36)-H(36C)	109.5

H(36A)-C(36)-H(36C)	109.5
H(36B)-C(36)-H(36C)	109.5
C(35)-C(37)-H(37A)	109.5
C(35)-C(37)-H(37B)	109.5
H(37A)-C(37)-H(37B)	109.5
C(35)-C(37)-H(37C)	109.5
H(37A)-C(37)-H(37C)	109.5
H(37B)-C(37)-H(37C)	109.5
C(35)-C(38)-H(38A)	109.5
C(35)-C(38)-H(38B)	109.5
H(38A)-C(38)-H(38B)	109.5
C(35)-C(38)-H(38C)	109.5
H(38A)-C(38)-H(38C)	109.5
H(38B)-C(38)-H(38C)	109.5
C(13)-N(1)-P(1)	130.5(3)
C(13)-N(1)-P(2)	128.9(3)
P(1)-N(1)-P(2)	100.50(17)
C(20)-N(2)-P(2)	129.6(3)
C(20)-N(2)-P(1)	129.5(3)
P(2)-N(2)-P(1)	100.36(17)
C(7)-N(3)-C(1)	120.5(3)
C(7)-N(3)-P(1)	111.2(3)
C(1)-N(3)-P(1)	110.1(3)
C(33)-N(4)-C(27)	120.2(3)
C(33)-N(4)-P(2)	110.3(3)
C(27)-N(4)-P(2)	109.4(3)
C(2)-O(1)-P(1)	114.8(3)
C(8)-O(2)-P(1)	113.8(3)
C(28)-O(3)-P(2)	114.5(3)
C(34)-O(4)-P(2)	114.2(3)
N(1)-P(1)-O(1)	117.50(17)
N(1)-P(1)-O(2)	117.93(17)
O(1)-P(1)-O(2)	123.82(16)
N(1)-P(1)-N(2)	79.84(15)
O(1)-P(1)-N(2)	90.40(15)
O(2)-P(1)-N(2)	90.66(15)

N(1)-P(1)-N(3)	99.64(16)
O(1)-P(1)-N(3)	89.91(15)
O(2)-P(1)-N(3)	89.52(15)
N(2)-P(1)-N(3)	179.47(17)
O(4)-P(2)-O(3)	123.29(16)
O(4)-P(2)-N(2)	117.23(16)
O(3)-P(2)-N(2)	118.74(17)
O(4)-P(2)-N(1)	91.40(15)
O(3)-P(2)-N(1)	90.34(15)
N(2)-P(2)-N(1)	79.31(15)
O(4)-P(2)-N(4)	89.50(15)
O(3)-P(2)-N(4)	90.04(15)
N(2)-P(2)-N(4)	99.34(16)
N(1)-P(2)-N(4)	178.61(17)

Symmetry transformations used to generate equivalent atoms:

Table S34. Anisotropic displacement parameters (Å^{2}x 10^3) for **9'**. The anisotropic displacement factor exponent takes the form: $-2\pi^2[h^2 a^{*2}U^{11} + ... + 2 h k a^* b^* U^{12}]$

	U^{11}	U^{22}	U^{33}	U^{23}	U^{13}	U^{12}
C(1)	36(2)	58(3)	57(3)	-2(2)	-8(2)	10(2)
C(2)	47(3)	46(2)	54(3)	2(2)	-4(2)	10(2)
C(3)	68(3)	75(4)	58(3)	16(3)	-7(3)	5(3)
C(4)	270(13)	135(8)	140(8)	95(6)	-86(8)	-103(8)
C(5)	231(11)	177(9)	72(5)	32(5)	58(6)	77(8)
C(6)	101(6)	227(10)	97(5)	79(6)	-9(4)	54(6)
C(7)	33(2)	53(3)	64(3)	-1(2)	6(2)	6(2)
C(8)	45(3)	53(3)	56(3)	-3(2)	16(2)	7(2)
C(9)	68(3)	81(4)	56(3)	-11(3)	17(3)	13(3)
C(10)	164(8)	110(6)	81(5)	-39(4)	15(5)	-7(5)
C(11)	100(5)	145(6)	59(4)	12(4)	11(3)	31(5)
C(12)	84(5)	163(7)	76(4)	-22(4)	33(4)	7(5)
C(13)	28(2)	35(2)	59(3)	5(2)	1(2)	2(2)

C(14)	52(3)	56(3)	58(3)	-2(2)	-1(2)	-8(2)
C(15)	64(3)	62(3)	86(4)	-14(3)	-21(3)	-4(3)
C(16)	49(3)	47(3)	120(5)	-4(3)	-1(3)	-10(2)
C(17)	67(4)	56(3)	112(5)	11(3)	36(3)	-11(3)
C(18)	59(3)	49(3)	70(3)	-1(2)	24(2)	-8(2)
C(19)	95(5)	74(5)	205(9)	-8(5)	-8(5)	-42(4)
C(20)	32(2)	34(2)	54(3)	0(2)	6(2)	1(2)
C(21)	56(3)	62(3)	55(3)	-7(2)	2(2)	-12(2)
C(22)	66(4)	66(3)	92(4)	-22(3)	-13(3)	-11(3)
C(23)	47(3)	50(3)	129(5)	-13(3)	11(3)	-10(2)
C(24)	68(3)	56(3)	100(5)	8(3)	31(3)	-13(3)
C(25)	67(3)	50(3)	63(3)	-1(2)	19(2)	-11(2)
C(26)	103(6)	84(5)	212(10)	-23(5)	15(6)	-49(4)
C(27)	35(2)	61(3)	49(3)	2(2)	-5(2)	4(2)
C(28)	42(2)	55(3)	50(3)	6(2)	1(2)	7(2)
C(29)	64(3)	80(4)	61(3)	24(3)	0(3)	6(3)
C(30)	186(9)	137(7)	66(4)	14(4)	49(5)	10(6)
C(31)	184(9)	89(5)	112(6)	56(5)	-14(6)	-36(5)
C(32)	89(5)	251(11)	114(6)	108(7)	-13(4)	31(6)
C(33)	33(2)	58(3)	54(3)	6(2)	10(2)	8(2)
C(34)	43(2)	52(3)	50(3)	3(2)	11(2)	10(2)
C(35)	59(3)	90(4)	48(3)	-2(3)	12(2)	19(3)
C(36)	182(8)	96(5)	73(4)	-30(4)	3(5)	23(5)
C(37)	79(5)	279(12)	65(4)	-3(6)	31(4)	38(6)
C(38)	100(5)	108(5)	59(4)	13(3)	0(3)	10(4)
N(1)	28(2)	34(2)	52(2)	4(2)	2(1)	-2(1)
N(2)	30(2)	35(2)	51(2)	0(2)	2(1)	-3(1)
N(3)	26(2)	46(2)	54(2)	0(2)	-1(2)	6(1)
N(4)	29(2)	46(2)	49(2)	5(2)	1(1)	-1(1)
O(1)	42(2)	45(2)	56(2)	14(1)	-1(1)	-1(1)
O(2)	37(2)	53(2)	52(2)	-8(1)	5(1)	1(1)
O(3)	36(2)	48(2)	52(2)	15(1)	4(1)	1(1)
O(4)	33(2)	55(2)	48(2)	-3(1)	3(1)	4(1)
P(1)	27(1)	35(1)	43(1)	1(1)	1(1)	1(1)
P(2)	28(1)	36(1)	41(1)	3(1)	2(1)	1(1)

Table S35. Hydrogen coordinates (x 10^4) and isotropic displacement parameters ($Å^2$x 10^3) for **9'**.

	x	y	z	U(eq)
H(1)	9453	533	3189	61
H(4A)	5495	-483	3695	280
H(4B)	6335	-794	4292	280
H(4C)	6880	-895	3609	280
H(5A)	7236	809	4458	236
H(5B)	6839	216	4862	236
H(5C)	5724	478	4327	236
H(6A)	9303	-470	3907	214
H(6B)	8840	-437	4610	214
H(6C)	9458	169	4290	214
H(7)	9577	772	1840	60
H(10A)	7870	-458	851	177
H(10B)	7273	-271	157	177
H(10C)	6243	-256	718	177
H(11A)	5609	872	490	152
H(11B)	6585	846	-90	152
H(11C)	6845	1374	433	152
H(12A)	9422	1067	634	159
H(12B)	9210	551	97	159
H(12C)	9840	358	784	159
H(14)	6593	2248	3545	66
H(15)	8123	3103	3703	86
H(17)	8864	3100	1874	92
H(18)	7260	2271	1686	71
H(19A)	10055	3869	2508	188
H(19B)	9199	4109	3078	188
H(19C)	10488	3631	3204	188
H(21)	3216	240	1296	70
H(22)	1665	-615	1233	91
H(24)	1339	-555	3094	88

H(25)	2910	298	3179	71
H(26A)	803	-1618	2223	199
H(26B)	-134	-1268	1678	199
H(26C)	-466	-1169	2393	199
H(27)	522	2020	1612	58
H(30A)	4296	2065	520	192
H(30B)	3312	2371	-39	192
H(30C)	2773	1771	317	192
H(31A)	3070	3454	1242	195
H(31B)	3532	3404	540	195
H(31C)	4475	3078	1100	195
H(32A)	530	2353	543	228
H(32B)	1102	2940	175	228
H(32C)	644	3020	875	228
H(33)	399	1762	2950	58
H(36A)	3917	2692	4082	176
H(36B)	3012	2710	4685	176
H(36C)	2348	2979	4031	176
H(37A)	194	2281	4010	210
H(37B)	730	2018	4686	210
H(37C)	382	1548	4116	210
H(38A)	2823	1096	4339	134
H(38B)	3244	1596	4875	134
H(38C)	4212	1517	4297	134

Table S36. Torsion angles [°] for **9'.**

N(3)-C(1)-C(2)-O(1)	1.3(6)
N(3)-C(1)-C(2)-C(3)	-174.3(5)
C(1)-C(2)-C(3)-C(4)	-134.5(7)
O(1)-C(2)-C(3)-C(4)	50.0(8)
C(1)-C(2)-C(3)-C(5)	105.7(7)
O(1)-C(2)-C(3)-C(5)	-69.8(7)
C(1)-C(2)-C(3)-C(6)	-13.9(9)
O(1)-C(2)-C(3)-C(6)	170.6(6)
N(3)-C(7)-C(8)-O(2)	-2.5(5)
N(3)-C(7)-C(8)-C(9)	175.0(5)
C(7)-C(8)-C(9)-C(10)	113.4(7)
O(2)-C(8)-C(9)-C(10)	-69.1(6)
C(7)-C(8)-C(9)-C(11)	-126.8(6)
O(2)-C(8)-C(9)-C(11)	50.8(6)
C(7)-C(8)-C(9)-C(12)	-6.9(8)
O(2)-C(8)-C(9)-C(12)	170.6(5)
C(18)-C(13)-C(14)-C(15)	0.8(7)
N(1)-C(13)-C(14)-C(15)	-178.9(4)
C(13)-C(14)-C(15)-C(16)	-0.9(8)
C(14)-C(15)-C(16)-C(17)	-0.4(8)
C(14)-C(15)-C(16)-C(19)	-178.6(5)
C(15)-C(16)-C(17)-C(18)	1.9(8)
C(19)-C(16)-C(17)-C(18)	-179.9(6)
C(14)-C(13)-C(18)-C(17)	0.7(7)
N(1)-C(13)-C(18)-C(17)	-179.7(4)
C(16)-C(17)-C(18)-C(13)	-2.1(8)
C(25)-C(20)-C(21)-C(22)	0.6(7)
N(2)-C(20)-C(21)-C(22)	178.7(4)
C(20)-C(21)-C(22)-C(23)	0.0(8)
C(21)-C(22)-C(23)-C(24)	-0.3(9)
C(21)-C(22)-C(23)-C(26)	179.0(6)
C(22)-C(23)-C(24)-C(25)	0.0(9)
C(26)-C(23)-C(24)-C(25)	-179.3(6)
C(21)-C(20)-C(25)-C(24)	-0.9(7)

N(2)-C(20)-C(25)-C(24)	-179.1(4)
C(23)-C(24)-C(25)-C(20)	0.6(8)
N(4)-C(27)-C(28)-O(3)	-2.8(6)
N(4)-C(27)-C(28)-C(29)	174.3(5)
C(27)-C(28)-C(29)-C(31)	131.8(7)
O(3)-C(28)-C(29)-C(31)	-51.2(7)
C(27)-C(28)-C(29)-C(32)	10.0(9)
O(3)-C(28)-C(29)-C(32)	-173.0(6)
C(27)-C(28)-C(29)-C(30)	-107.9(7)
O(3)-C(28)-C(29)-C(30)	69.1(6)
N(4)-C(33)-C(34)-O(4)	2.9(5)
N(4)-C(33)-C(34)-C(35)	-173.7(5)
C(33)-C(34)-C(35)-C(38)	118.6(6)
O(4)-C(34)-C(35)-C(38)	-57.9(6)
C(33)-C(34)-C(35)-C(36)	-122.0(6)
O(4)-C(34)-C(35)-C(36)	61.5(6)
C(33)-C(34)-C(35)-C(37)	-1.0(9)
O(4)-C(34)-C(35)-C(37)	-177.5(5)
C(18)-C(13)-N(1)-P(1)	78.2(5)
C(14)-C(13)-N(1)-P(1)	-102.2(4)
C(18)-C(13)-N(1)-P(2)	-99.4(5)
C(14)-C(13)-N(1)-P(2)	80.2(5)
C(25)-C(20)-N(2)-P(2)	-77.4(5)
C(21)-C(20)-N(2)-P(2)	104.5(4)
C(25)-C(20)-N(2)-P(1)	92.5(5)
C(21)-C(20)-N(2)-P(1)	-85.7(5)
C(8)-C(7)-N(3)-C(1)	126.9(4)
C(8)-C(7)-N(3)-P(1)	-4.0(5)
C(2)-C(1)-N(3)-C(7)	-128.6(4)
C(2)-C(1)-N(3)-P(1)	2.9(5)
C(34)-C(33)-N(4)-C(27)	-125.7(4)
C(34)-C(33)-N(4)-P(2)	3.0(5)
C(28)-C(27)-N(4)-C(33)	127.3(4)
C(28)-C(27)-N(4)-P(2)	-1.9(5)
C(1)-C(2)-O(1)-P(1)	-5.4(5)
C(3)-C(2)-O(1)-P(1)	171.1(3)

C(7)-C(8)-O(2)-P(1)	8.6(5)
C(9)-C(8)-O(2)-P(1)	-169.5(3)
C(27)-C(28)-O(3)-P(2)	6.8(5)
C(29)-C(28)-O(3)-P(2)	-170.9(3)
C(33)-C(34)-O(4)-P(2)	-8.1(5)
C(35)-C(34)-O(4)-P(2)	169.1(3)
C(13)-N(1)-P(1)-O(1)	97.0(4)
P(2)-N(1)-P(1)-O(1)	-84.9(2)
C(13)-N(1)-P(1)-O(2)	-92.6(4)
P(2)-N(1)-P(1)-O(2)	85.53(19)
C(13)-N(1)-P(1)-N(2)	-177.9(4)
P(2)-N(1)-P(1)-N(2)	0.22(16)
C(13)-N(1)-P(1)-N(3)	2.0(4)
P(2)-N(1)-P(1)-N(3)	-179.85(17)
C(2)-O(1)-P(1)-N(1)	-95.0(3)
C(2)-O(1)-P(1)-O(2)	95.2(3)
C(2)-O(1)-P(1)-N(2)	-173.7(3)
C(2)-O(1)-P(1)-N(3)	5.9(3)
C(8)-O(2)-P(1)-N(1)	91.7(3)
C(8)-O(2)-P(1)-O(1)	-98.5(3)
C(8)-O(2)-P(1)-N(2)	170.6(3)
C(8)-O(2)-P(1)-N(3)	-8.9(3)
C(20)-N(2)-P(1)-N(1)	-172.3(4)
P(2)-N(2)-P(1)-N(1)	-0.23(18)
C(20)-N(2)-P(1)-O(1)	-54.4(4)
P(2)-N(2)-P(1)-O(1)	117.66(18)
C(20)-N(2)-P(1)-O(2)	69.4(4)
P(2)-N(2)-P(1)-O(2)	-118.51(18)
C(7)-N(3)-P(1)-N(1)	-110.8(3)
C(1)-N(3)-P(1)-N(1)	113.0(3)
C(7)-N(3)-P(1)-O(1)	131.3(3)
C(1)-N(3)-P(1)-O(1)	-4.9(3)
C(7)-N(3)-P(1)-O(2)	7.4(3)
C(1)-N(3)-P(1)-O(2)	-128.7(3)
C(34)-O(4)-P(2)-O(3)	97.8(3)
C(34)-O(4)-P(2)-N(2)	-92.1(3)

C(34)-O(4)-P(2)-N(1)	-170.8(3)
C(34)-O(4)-P(2)-N(4)	8.1(3)
C(28)-O(3)-P(2)-O(4)	-96.0(3)
C(28)-O(3)-P(2)-N(2)	94.2(3)
C(28)-O(3)-P(2)-N(1)	172.1(3)
C(28)-O(3)-P(2)-N(4)	-6.5(3)
C(20)-N(2)-P(2)-O(4)	86.2(4)
P(1)-N(2)-P(2)-O(4)	-85.82(19)
C(20)-N(2)-P(2)-O(3)	-103.3(4)
P(1)-N(2)-P(2)-O(3)	84.7(2)
C(20)-N(2)-P(2)-N(1)	172.2(4)
P(1)-N(2)-P(2)-N(1)	0.22(16)
C(20)-N(2)-P(2)-N(4)	-8.1(4)
P(1)-N(2)-P(2)-N(4)	179.89(16)
C(13)-N(1)-P(2)-O(4)	-64.6(4)
P(1)-N(1)-P(2)-O(4)	117.23(18)
C(13)-N(1)-P(2)-O(3)	58.7(4)
P(1)-N(1)-P(2)-O(3)	-119.45(18)
C(13)-N(1)-P(2)-N(2)	177.9(4)
P(1)-N(1)-P(2)-N(2)	-0.23(17)
C(33)-N(4)-P(2)-O(4)	-6.4(3)
C(27)-N(4)-P(2)-O(4)	128.1(3)
C(33)-N(4)-P(2)-O(3)	-129.7(3)
C(27)-N(4)-P(2)-O(3)	4.8(3)
C(33)-N(4)-P(2)-N(2)	111.2(3)
C(27)-N(4)-P(2)-N(2)	-114.4(3)

Symmetry transformations used to generate equivalent atoms:

Compound **15**:

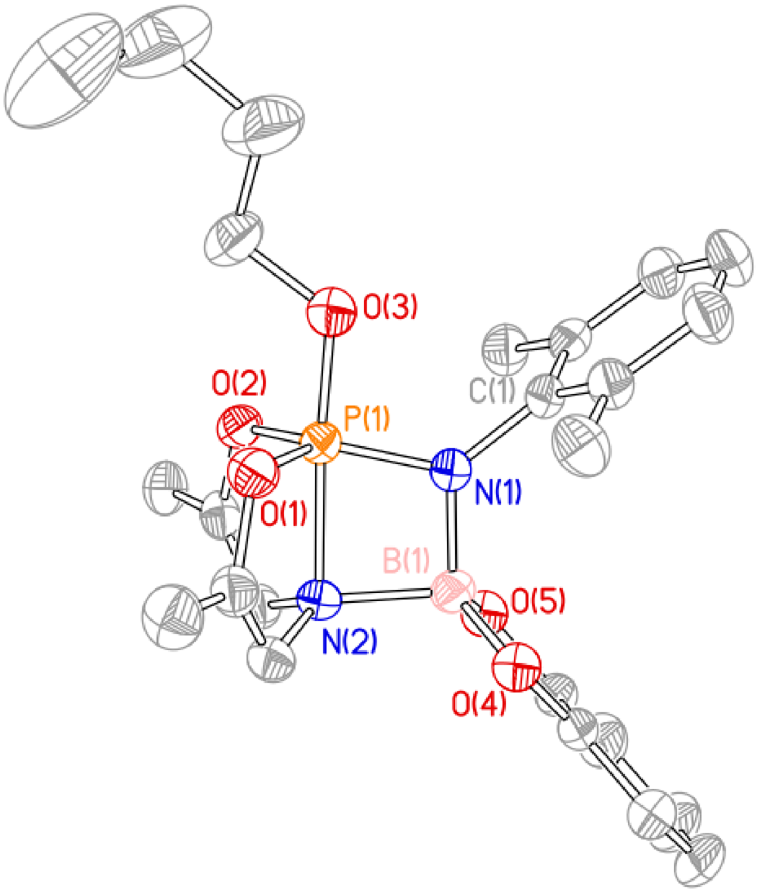

Table S37. Crystal data and structure refinement for **15**.

Identification code	yzl11o	
Empirical formula	C34 H50 B N2 O5 P	
Formula weight	608.54	
Temperature	193(2) K	
Wavelength	0.71073 Å	
Crystal system	Orthorhombic	
Space group	Pbca	
Unit cell dimensions	a = 19.707(5) Å	α= 90°.
	b = 17.611(4) Å	β= 90°.
	c = 20.439(5) Å	γ = 90°.
Volume	7094(3) Å^3	
Z	8	
Density (calculated)	1.140 Mg/m^3	
Absorption coefficient	0.117 mm^{-1}	
F(000)	2624	
Crystal size	0.230 x 0.190 x 0.060 mm^3	
Theta range for data collection	1.843 to 25.000°.	
Index ranges	-23<=h<=23, -20<=k<=20, -24<=l<=24	
Reflections collected	44609	
Independent reflections	6249 [R(int) = 0.0429]	
Completeness to theta = 25.000°	100.0 %	
Absorption correction	Semi-empirical from equivalents	
Max. and min. transmission	0.9930 and 0.9735	
Refinement method	Full-matrix least-squares on F^2	
Data / restraints / parameters	6249 / 0 / 388	
Goodness-of-fit on F^2	0.960	
Final R indices [I>2sigma(I)]	R1 = 0.0535, wR2 = 0.2029	
R indices (all data)	R1 = 0.0670, wR2 = 0.2269	
Extinction coefficient	n/a	
Largest diff. peak and hole	0.878 and -0.448 e.Å^{-3}	

Table S38. Atomic coordinates (x 10^4) and equivalent isotropic displacement parameters ($Å^2$x 10^3) for **15**. U(eq) is defined as one third of the trace of the orthogonalized U^{ij} tensor.

	x	y	z	U(eq)
C(1)	3283(1)	9637(1)	3629(1)	31(1)
C(2)	1185(1)	8754(1)	2891(1)	33(1)
C(3)	672(1)	9021(2)	2398(1)	41(1)
C(4)	513(2)	8362(2)	1930(2)	67(1)
C(5)	31(1)	9284(2)	2738(2)	65(1)
C(6)	980(2)	9675(2)	2006(1)	58(1)
C(7)	1876(1)	7593(1)	3990(1)	34(1)
C(8)	1730(1)	7930(1)	4546(1)	36(1)
C(9)	1556(1)	7617(2)	5208(1)	47(1)
C(10)	2118(2)	7835(2)	5688(2)	77(1)
C(11)	1503(2)	6752(2)	5166(2)	71(1)
C(12)	873(2)	7946(2)	5424(2)	74(1)
C(13)	3421(1)	7767(1)	2463(1)	34(1)
C(14)	3692(1)	7552(2)	1872(1)	49(1)
C(15)	4211(2)	7008(2)	1890(2)	59(1)
C(16)	4434(1)	6707(2)	2472(2)	58(1)
C(17)	4151(1)	6932(2)	3070(1)	45(1)
C(18)	3644(1)	7464(1)	3049(1)	33(1)
C(19)	1526(1)	8116(1)	2942(1)	33(1)
C(20)	3729(1)	9613(1)	4164(1)	37(1)
C(21)	4257(1)	10141(2)	4180(1)	48(1)
C(22)	4320(1)	10685(2)	3707(1)	52(1)
C(23)	3872(1)	10711(2)	3190(1)	45(1)
C(24)	3339(1)	10187(1)	3141(1)	35(1)
C(25)	2855(1)	10239(2)	2567(1)	42(1)
C(26)	3212(2)	10097(2)	1913(1)	68(1)
C(27)	2482(2)	10996(2)	2570(2)	67(1)
C(28)	3658(1)	9043(2)	4721(1)	44(1)
C(29)	3482(2)	9458(2)	5355(1)	66(1)
C(30)	4295(2)	8560(2)	4803(2)	61(1)
C(31)	1408(1)	10328(2)	4407(2)	55(1)

C(32)	1589(2)	10985(2)	4792(2)	91(1)
C(33)	989(3)	11409(3)	5066(2)	108(2)
C(34)	577(3)	10985(4)	5526(2)	144(3)
B(1)	2803(1)	8297(1)	3274(1)	30(1)
N(1)	2752(1)	9073(1)	3589(1)	29(1)
N(2)	2013(1)	8115(1)	3464(1)	29(1)
O(1)	1744(1)	8721(1)	4529(1)	35(1)
O(2)	1347(1)	9303(1)	3360(1)	34(1)
O(4)	3288(1)	7770(1)	3563(1)	34(1)
O(3)	2005(1)	9952(1)	4152(1)	36(1)
O(5)	2916(1)	8284(1)	2568(1)	35(1)
P(1)	1977(1)	9121(1)	3849(1)	28(1)

Table S39. Bond lengths [Å] and angles [°] for **15**.

C(1)-C(24)	1.396(3)
C(1)-C(20)	1.404(3)
C(1)-N(1)	1.444(3)
C(2)-C(19)	1.314(3)
C(2)-O(2)	1.398(3)
C(2)-C(3)	1.503(3)
C(3)-C(5)	1.515(3)
C(3)-C(6)	1.529(4)
C(3)-C(4)	1.535(4)
C(4)-H(4A)	0.9800
C(4)-H(4B)	0.9800
C(4)-H(4C)	0.9800
C(5)-H(5A)	0.9800
C(5)-H(5B)	0.9800
C(5)-H(5C)	0.9800
C(6)-H(6A)	0.9800
C(6)-H(6B)	0.9800
C(6)-H(6C)	0.9800

C(7)-C(8)	1.314(3)
C(7)-N(2)	1.441(3)
C(7)-H(7)	0.9500
C(8)-O(1)	1.395(3)
C(8)-C(9)	1.499(3)
C(9)-C(11)	1.530(4)
C(9)-C(12)	1.529(4)
C(9)-C(10)	1.530(4)
C(10)-H(10A)	0.9800
C(10)-H(10B)	0.9800
C(10)-H(10C)	0.9800
C(11)-H(11A)	0.9800
C(11)-H(11B)	0.9800
C(11)-H(11C)	0.9800
C(12)-H(12A)	0.9800
C(12)-H(12B)	0.9800
C(12)-H(12C)	0.9800
C(13)-O(5)	1.366(3)
C(13)-C(14)	1.375(3)
C(13)-C(18)	1.383(3)
C(14)-C(15)	1.401(4)
C(14)-H(14)	0.9500
C(15)-C(16)	1.375(4)
C(15)-H(15)	0.9500
C(16)-C(17)	1.399(4)
C(16)-H(16)	0.9500
C(17)-C(18)	1.371(3)
C(17)-H(17)	0.9500
C(18)-O(4)	1.374(3)
C(19)-N(2)	1.435(3)
C(19)-H(19)	0.9500
C(20)-C(21)	1.396(3)
C(20)-C(28)	1.524(3)
C(21)-C(22)	1.366(4)
C(21)-H(21)	0.9500
C(22)-C(23)	1.378(4)

C(22)-H(22)	0.9500
C(23)-C(24)	1.402(3)
C(23)-H(23)	0.9500
C(24)-C(25)	1.514(3)
C(25)-C(27)	1.523(4)
C(25)-C(26)	1.530(4)
C(25)-H(25)	1.0000
C(26)-H(26A)	0.9800
C(26)-H(26B)	0.9800
C(26)-H(26C)	0.9800
C(27)-H(27A)	0.9800
C(27)-H(27B)	0.9800
C(27)-H(27C)	0.9800
C(28)-C(30)	1.525(4)
C(28)-C(29)	1.529(4)
C(28)-H(28)	1.0000
C(29)-H(29A)	0.9800
C(29)-H(29B)	0.9800
C(29)-H(29C)	0.9800
C(30)-H(30A)	0.9800
C(30)-H(30B)	0.9800
C(30)-H(30C)	0.9800
C(31)-C(32)	1.443(4)
C(31)-O(3)	1.447(3)
C(31)-H(31A)	0.9900
C(31)-H(31B)	0.9900
C(32)-C(33)	1.507(6)
C(32)-H(32A)	0.9900
C(32)-H(32B)	0.9900
C(33)-C(34)	1.450(7)
C(33)-H(33A)	0.9900
C(33)-H(33B)	0.9900
C(34)-H(34A)	0.9800
C(34)-H(34B)	0.9800
C(34)-H(34C)	0.9800
B(1)-O(4)	1.456(3)

B(1)-O(5)	1.460(3)
B(1)-N(1)	1.515(3)
B(1)-N(2)	1.637(3)
N(1)-P(1)	1.6195(18)
N(2)-P(1)	1.9392(19)
O(1)-P(1)	1.6242(16)
O(2)-P(1)	1.6252(16)
O(3)-P(1)	1.5906(16)
C(24)-C(1)-C(20)	121.9(2)
C(24)-C(1)-N(1)	119.6(2)
C(20)-C(1)-N(1)	118.5(2)
C(19)-C(2)-O(2)	114.86(19)
C(19)-C(2)-C(3)	131.6(2)
O(2)-C(2)-C(3)	113.4(2)
C(2)-C(3)-C(5)	110.4(2)
C(2)-C(3)-C(6)	108.6(2)
C(5)-C(3)-C(6)	110.0(2)
C(2)-C(3)-C(4)	108.6(2)
C(5)-C(3)-C(4)	110.3(2)
C(6)-C(3)-C(4)	108.9(2)
C(3)-C(4)-H(4A)	109.5
C(3)-C(4)-H(4B)	109.5
H(4A)-C(4)-H(4B)	109.5
C(3)-C(4)-H(4C)	109.5
H(4A)-C(4)-H(4C)	109.5
H(4B)-C(4)-H(4C)	109.5
C(3)-C(5)-H(5A)	109.5
C(3)-C(5)-H(5B)	109.5
H(5A)-C(5)-H(5B)	109.5
C(3)-C(5)-H(5C)	109.5
H(5A)-C(5)-H(5C)	109.5
H(5B)-C(5)-H(5C)	109.5
C(3)-C(6)-H(6A)	109.5
C(3)-C(6)-H(6B)	109.5
H(6A)-C(6)-H(6B)	109.5
C(3)-C(6)-H(6C)	109.5

H(6A)-C(6)-H(6C)	109.5
H(6B)-C(6)-H(6C)	109.5
C(8)-C(7)-N(2)	113.5(2)
C(8)-C(7)-H(7)	123.3
N(2)-C(7)-H(7)	123.3
C(7)-C(8)-O(1)	115.2(2)
C(7)-C(8)-C(9)	131.6(2)
O(1)-C(8)-C(9)	113.2(2)
C(8)-C(9)-C(11)	109.3(2)
C(8)-C(9)-C(12)	108.9(2)
C(11)-C(9)-C(12)	109.5(3)
C(8)-C(9)-C(10)	108.7(2)
C(11)-C(9)-C(10)	109.5(3)
C(12)-C(9)-C(10)	110.9(3)
C(9)-C(10)-H(10A)	109.5
C(9)-C(10)-H(10B)	109.5
H(10A)-C(10)-H(10B)	109.5
C(9)-C(10)-H(10C)	109.5
H(10A)-C(10)-H(10C)	109.5
H(10B)-C(10)-H(10C)	109.5
C(9)-C(11)-H(11A)	109.5
C(9)-C(11)-H(11B)	109.5
H(11A)-C(11)-H(11B)	109.5
C(9)-C(11)-H(11C)	109.5
H(11A)-C(11)-H(11C)	109.5
H(11B)-C(11)-H(11C)	109.5
C(9)-C(12)-H(12A)	109.5
C(9)-C(12)-H(12B)	109.5
H(12A)-C(12)-H(12B)	109.5
C(9)-C(12)-H(12C)	109.5
H(12A)-C(12)-H(12C)	109.5
H(12B)-C(12)-H(12C)	109.5
O(5)-C(13)-C(14)	127.2(2)
O(5)-C(13)-C(18)	110.65(19)
C(14)-C(13)-C(18)	122.1(2)
C(13)-C(14)-C(15)	116.6(3)

C(13)-C(14)-H(14)	121.7
C(15)-C(14)-H(14)	121.7
C(16)-C(15)-C(14)	121.3(2)
C(16)-C(15)-H(15)	119.3
C(14)-C(15)-H(15)	119.3
C(15)-C(16)-C(17)	121.3(3)
C(15)-C(16)-H(16)	119.3
C(17)-C(16)-H(16)	119.3
C(18)-C(17)-C(16)	117.1(3)
C(18)-C(17)-H(17)	121.4
C(16)-C(17)-H(17)	121.4
C(17)-C(18)-O(4)	128.1(2)
C(17)-C(18)-C(13)	121.5(2)
O(4)-C(18)-C(13)	110.39(19)
C(2)-C(19)-N(2)	113.7(2)
C(2)-C(19)-H(19)	123.1
N(2)-C(19)-H(19)	123.1
C(21)-C(20)-C(1)	117.7(2)
C(21)-C(20)-C(28)	119.4(2)
C(1)-C(20)-C(28)	122.9(2)
C(22)-C(21)-C(20)	121.3(3)
C(22)-C(21)-H(21)	119.4
C(20)-C(21)-H(21)	119.4
C(21)-C(22)-C(23)	120.5(2)
C(21)-C(22)-H(22)	119.8
C(23)-C(22)-H(22)	119.8
C(22)-C(23)-C(24)	120.9(2)
C(22)-C(23)-H(23)	119.5
C(24)-C(23)-H(23)	119.5
C(1)-C(24)-C(23)	117.7(2)
C(1)-C(24)-C(25)	123.1(2)
C(23)-C(24)-C(25)	119.2(2)
C(24)-C(25)-C(27)	110.7(2)
C(24)-C(25)-C(26)	112.2(2)
C(27)-C(25)-C(26)	111.6(3)
C(24)-C(25)-H(25)	107.4

C(27)-C(25)-H(25)	107.4
C(26)-C(25)-H(25)	107.4
C(25)-C(26)-H(26A)	109.5
C(25)-C(26)-H(26B)	109.5
H(26A)-C(26)-H(26B)	109.5
C(25)-C(26)-H(26C)	109.5
H(26A)-C(26)-H(26C)	109.5
H(26B)-C(26)-H(26C)	109.5
C(25)-C(27)-H(27A)	109.5
C(25)-C(27)-H(27B)	109.5
H(27A)-C(27)-H(27B)	109.5
C(25)-C(27)-H(27C)	109.5
H(27A)-C(27)-H(27C)	109.5
H(27B)-C(27)-H(27C)	109.5
C(30)-C(28)-C(20)	111.9(2)
C(30)-C(28)-C(29)	111.1(2)
C(20)-C(28)-C(29)	109.8(2)
C(30)-C(28)-H(28)	108.0
C(20)-C(28)-H(28)	108.0
C(29)-C(28)-H(28)	108.0
C(28)-C(29)-H(29A)	109.5
C(28)-C(29)-H(29B)	109.5
H(29A)-C(29)-H(29B)	109.5
C(28)-C(29)-H(29C)	109.5
H(29A)-C(29)-H(29C)	109.5
H(29B)-C(29)-H(29C)	109.5
C(28)-C(30)-H(30A)	109.5
C(28)-C(30)-H(30B)	109.5
H(30A)-C(30)-H(30B)	109.5
C(28)-C(30)-H(30C)	109.5
H(30A)-C(30)-H(30C)	109.5
H(30B)-C(30)-H(30C)	109.5
C(32)-C(31)-O(3)	111.3(3)
C(32)-C(31)-H(31A)	109.4
O(3)-C(31)-H(31A)	109.4
C(32)-C(31)-H(31B)	109.4

O(3)-C(31)-H(31B)	109.4
H(31A)-C(31)-H(31B)	108.0
C(31)-C(32)-C(33)	113.9(4)
C(31)-C(32)-H(32A)	108.8
C(33)-C(32)-H(32A)	108.8
C(31)-C(32)-H(32B)	108.8
C(33)-C(32)-H(32B)	108.8
H(32A)-C(32)-H(32B)	107.7
C(34)-C(33)-C(32)	115.2(5)
C(34)-C(33)-H(33A)	108.5
C(32)-C(33)-H(33A)	108.5
C(34)-C(33)-H(33B)	108.5
C(32)-C(33)-H(33B)	108.5
H(33A)-C(33)-H(33B)	107.5
C(33)-C(34)-H(34A)	109.5
C(33)-C(34)-H(34B)	109.5
H(34A)-C(34)-H(34B)	109.5
C(33)-C(34)-H(34C)	109.5
H(34A)-C(34)-H(34C)	109.5
H(34B)-C(34)-H(34C)	109.5
O(4)-B(1)-O(5)	106.95(18)
O(4)-B(1)-N(1)	116.48(19)
O(5)-B(1)-N(1)	116.44(18)
O(4)-B(1)-N(2)	113.73(18)
O(5)-B(1)-N(2)	112.09(18)
N(1)-B(1)-N(2)	90.68(15)
C(1)-N(1)-B(1)	126.58(17)
C(1)-N(1)-P(1)	128.98(15)
B(1)-N(1)-P(1)	104.42(14)
C(19)-N(2)-C(7)	115.49(18)
C(19)-N(2)-B(1)	117.34(17)
C(7)-N(2)-B(1)	118.73(17)
C(19)-N(2)-P(1)	106.00(14)
C(7)-N(2)-P(1)	105.88(14)
B(1)-N(2)-P(1)	87.30(12)
C(8)-O(1)-P(1)	117.37(14)

C(2)-O(2)-P(1)	117.33(14)
C(18)-O(4)-B(1)	105.93(17)
C(31)-O(3)-P(1)	122.22(16)
C(13)-O(5)-B(1)	106.06(17)
O(3)-P(1)-N(1)	98.16(9)
O(3)-P(1)-O(1)	94.26(9)
N(1)-P(1)-O(1)	121.59(9)
O(3)-P(1)-O(2)	94.88(8)
N(1)-P(1)-O(2)	121.95(9)
O(1)-P(1)-O(2)	113.38(9)
O(3)-P(1)-N(2)	175.75(8)
N(1)-P(1)-N(2)	77.59(8)
O(1)-P(1)-N(2)	87.83(8)
O(2)-P(1)-N(2)	87.67(8)

Symmetry transformations used to generate equivalent atoms:

Table S40. Anisotropic displacement parameters ($Å^2$x 10^3) for **15**. The anisotropic displacement factor exponent takes the form: $-2\pi^2[h^2 a^{*2}U^{11} + ... + 2 h k a^* b^* U^{12}]$

	U^{11}	U^{22}	U^{33}	U^{23}	U^{13}	U^{12}
C(1)	24(1)	31(1)	38(1)	-6(1)	6(1)	-1(1)
C(2)	28(1)	39(1)	31(1)	-1(1)	2(1)	-4(1)
C(3)	33(1)	51(2)	38(1)	3(1)	-3(1)	0(1)
C(4)	69(2)	74(2)	57(2)	-4(2)	-28(2)	-10(2)
C(5)	34(1)	101(3)	59(2)	17(2)	-1(1)	15(2)
C(6)	51(2)	68(2)	54(2)	18(1)	-9(1)	-4(2)
C(7)	34(1)	27(1)	42(1)	4(1)	1(1)	-2(1)
C(8)	30(1)	35(1)	41(1)	4(1)	0(1)	-1(1)
C(9)	49(2)	50(2)	42(1)	9(1)	5(1)	0(1)
C(10)	88(3)	98(3)	45(2)	15(2)	-13(2)	-18(2)
C(11)	98(3)	57(2)	59(2)	19(2)	14(2)	-5(2)

C(12)	73(2)	81(2)	70(2)	27(2)	38(2)	14(2)
C(13)	31(1)	32(1)	41(1)	-6(1)	6(1)	-3(1)
C(14)	45(2)	56(2)	45(2)	-12(1)	10(1)	-3(1)
C(15)	50(2)	65(2)	61(2)	-22(2)	17(1)	8(2)
C(16)	39(2)	50(2)	85(2)	-21(2)	12(1)	10(1)
C(17)	34(1)	36(1)	64(2)	-1(1)	1(1)	2(1)
C(18)	26(1)	28(1)	44(1)	-7(1)	5(1)	-3(1)
C(19)	33(1)	34(1)	33(1)	-5(1)	2(1)	-3(1)
C(20)	31(1)	38(1)	42(1)	-5(1)	1(1)	-2(1)
C(21)	37(1)	52(2)	54(2)	-6(1)	-4(1)	-11(1)
C(22)	37(1)	48(2)	70(2)	-8(1)	7(1)	-16(1)
C(23)	38(1)	38(1)	60(2)	3(1)	14(1)	-5(1)
C(24)	30(1)	30(1)	45(1)	-3(1)	8(1)	3(1)
C(25)	36(1)	41(1)	50(1)	11(1)	1(1)	2(1)
C(26)	65(2)	91(3)	47(2)	10(2)	2(1)	14(2)
C(27)	54(2)	48(2)	100(2)	7(2)	-12(2)	11(2)
C(28)	45(2)	48(2)	38(1)	0(1)	-8(1)	-8(1)
C(29)	86(2)	71(2)	42(2)	-2(2)	0(2)	1(2)
C(30)	59(2)	57(2)	67(2)	7(2)	-14(2)	1(2)
C(31)	47(2)	54(2)	64(2)	-18(1)	9(1)	11(1)
C(32)	88(3)	78(3)	106(3)	-48(2)	2(2)	12(2)
C(33)	131(4)	102(4)	92(3)	-39(3)	3(3)	37(3)
C(34)	185(6)	187(6)	61(2)	14(3)	30(3)	84(5)
B(1)	30(1)	29(1)	31(1)	-1(1)	2(1)	2(1)
N(1)	25(1)	28(1)	33(1)	-3(1)	2(1)	0(1)
N(2)	30(1)	25(1)	32(1)	-2(1)	0(1)	-1(1)
O(1)	39(1)	35(1)	32(1)	-3(1)	6(1)	-3(1)
O(2)	30(1)	32(1)	39(1)	-6(1)	-2(1)	3(1)
O(4)	33(1)	34(1)	36(1)	-2(1)	2(1)	6(1)
O(3)	33(1)	32(1)	41(1)	-9(1)	4(1)	2(1)
O(5)	38(1)	35(1)	33(1)	-1(1)	4(1)	7(1)
P(1)	26(1)	28(1)	29(1)	-3(1)	2(1)	0(1)

Table S41. Hydrogen coordinates (x 10^4) and isotropic displacement parameters (Å^{2}x 10 ^{3}) for **15**.

	x	y	z	U(eq)
H(4A)	179	8529	1606	100
H(4B)	930	8205	1707	100
H(4C)	329	7934	2180	100
H(5A)	-298	9456	2410	97
H(5B)	-163	8862	2989	97
H(5C)	139	9704	3034	97
H(6A)	651	9854	1682	87
H(6B)	1098	10092	2303	87
H(6C)	1391	9498	1782	87
H(7)	1890	7057	3940	41
H(10A)	2010	7634	6122	115
H(10B)	2551	7622	5538	115
H(10C)	2155	8390	5710	115
H(11A)	1390	6546	5597	107
H(11B)	1148	6613	4852	107
H(11C)	1939	6542	5021	107
H(12A)	755	7745	5856	112
H(12B)	906	8501	5446	112
H(12C)	522	7803	5108	112
H(14)	3536	7761	1471	58
H(15)	4413	6844	1492	71
H(16)	4786	6340	2469	69
H(17)	4305	6725	3473	54
H(19)	1457	7695	2659	40
H(21)	4579	10122	4526	57
H(22)	4675	11048	3735	62
H(23)	3925	11089	2863	54
H(25)	2508	9830	2623	51
H(26A)	2882	10136	1556	102
H(26B)	3570	10476	1850	102

H(26C)	3413	9588	1914	102
H(27A)	2172	11018	2195	101
H(27B)	2222	11045	2976	101
H(27C)	2812	11411	2539	101
H(28)	3272	8695	4613	52
H(29A)	3072	9764	5290	99
H(29B)	3403	9087	5704	99
H(29C)	3860	9791	5479	99
H(30A)	4229	8202	5164	91
H(30B)	4382	8278	4398	91
H(30C)	4682	8890	4899	91
H(31A)	1114	10487	4039	66
H(31B)	1149	9968	4682	66
H(32A)	1857	11337	4515	109
H(32B)	1883	10821	5158	109
H(33A)	1156	11873	5286	130
H(33B)	697	11571	4697	130
H(34A)	201	11304	5677	216
H(34B)	396	10531	5311	216
H(34C)	856	10833	5901	216

Table S42. Torsion angles [°] for **15**.

C(19)-C(2)-C(3)-C(5)	126.9(3)
O(2)-C(2)-C(3)-C(5)	-56.5(3)
C(19)-C(2)-C(3)-C(6)	-112.4(3)
O(2)-C(2)-C(3)-C(6)	64.1(3)
C(19)-C(2)-C(3)-C(4)	5.9(4)
O(2)-C(2)-C(3)-C(4)	-177.6(2)
N(2)-C(7)-C(8)-O(1)	-1.0(3)
N(2)-C(7)-C(8)-C(9)	179.9(2)

C(7)-C(8)-C(9)-C(11)	-4.7(4)
O(1)-C(8)-C(9)-C(11)	176.1(2)
C(7)-C(8)-C(9)-C(12)	-124.3(3)
O(1)-C(8)-C(9)-C(12)	56.5(3)
C(7)-C(8)-C(9)-C(10)	114.8(3)
O(1)-C(8)-C(9)-C(10)	-64.4(3)
O(5)-C(13)-C(14)-C(15)	180.0(2)
C(18)-C(13)-C(14)-C(15)	-0.3(4)
C(13)-C(14)-C(15)-C(16)	0.1(4)
C(14)-C(15)-C(16)-C(17)	0.0(5)
C(15)-C(16)-C(17)-C(18)	0.1(4)
C(16)-C(17)-C(18)-O(4)	179.7(2)
C(16)-C(17)-C(18)-C(13)	-0.2(4)
O(5)-C(13)-C(18)-C(17)	-179.9(2)
C(14)-C(13)-C(18)-C(17)	0.4(4)
O(5)-C(13)-C(18)-O(4)	0.2(3)
C(14)-C(13)-C(18)-O(4)	-179.5(2)
O(2)-C(2)-C(19)-N(2)	-1.3(3)
C(3)-C(2)-C(19)-N(2)	175.2(2)
C(24)-C(1)-C(20)-C(21)	-2.8(3)
N(1)-C(1)-C(20)-C(21)	177.2(2)
C(24)-C(1)-C(20)-C(28)	176.9(2)
N(1)-C(1)-C(20)-C(28)	-3.1(3)
C(1)-C(20)-C(21)-C(22)	2.9(4)
C(28)-C(20)-C(21)-C(22)	-176.8(2)
C(20)-C(21)-C(22)-C(23)	-1.9(4)
C(21)-C(22)-C(23)-C(24)	0.7(4)
C(20)-C(1)-C(24)-C(23)	1.6(3)
N(1)-C(1)-C(24)-C(23)	-178.4(2)
C(20)-C(1)-C(24)-C(25)	-178.5(2)
N(1)-C(1)-C(24)-C(25)	1.5(3)
C(22)-C(23)-C(24)-C(1)	-0.6(4)
C(22)-C(23)-C(24)-C(25)	179.6(2)
C(1)-C(24)-C(25)-C(27)	118.5(3)
C(23)-C(24)-C(25)-C(27)	-61.7(3)
C(1)-C(24)-C(25)-C(26)	-116.1(3)

C(23)-C(24)-C(25)-C(26)	63.7(3)
C(21)-C(20)-C(28)-C(30)	-58.0(3)
C(1)-C(20)-C(28)-C(30)	122.3(3)
C(21)-C(20)-C(28)-C(29)	65.8(3)
C(1)-C(20)-C(28)-C(29)	-113.9(3)
O(3)-C(31)-C(32)-C(33)	179.4(4)
C(31)-C(32)-C(33)-C(34)	63.3(6)
C(24)-C(1)-N(1)-B(1)	91.8(3)
C(20)-C(1)-N(1)-B(1)	-88.2(3)
C(24)-C(1)-N(1)-P(1)	-86.8(3)
C(20)-C(1)-N(1)-P(1)	93.2(2)
O(4)-B(1)-N(1)-C(1)	63.1(3)
O(5)-B(1)-N(1)-C(1)	-64.6(3)
N(2)-B(1)-N(1)-C(1)	-179.81(19)
O(4)-B(1)-N(1)-P(1)	-118.01(18)
O(5)-B(1)-N(1)-P(1)	114.29(18)
N(2)-B(1)-N(1)-P(1)	-0.91(15)
C(2)-C(19)-N(2)-C(7)	114.0(2)
C(2)-C(19)-N(2)-B(1)	-98.2(2)
C(2)-C(19)-N(2)-P(1)	-2.9(2)
C(8)-C(7)-N(2)-C(19)	-113.1(2)
C(8)-C(7)-N(2)-B(1)	99.6(2)
C(8)-C(7)-N(2)-P(1)	3.8(2)
O(4)-B(1)-N(2)-C(19)	-133.1(2)
O(5)-B(1)-N(2)-C(19)	-11.7(3)
N(1)-B(1)-N(2)-C(19)	107.37(19)
O(4)-B(1)-N(2)-C(7)	13.6(3)
O(5)-B(1)-N(2)-C(7)	135.1(2)
N(1)-B(1)-N(2)-C(7)	-105.9(2)
O(4)-B(1)-N(2)-P(1)	120.23(17)
O(5)-B(1)-N(2)-P(1)	-118.30(17)
N(1)-B(1)-N(2)-P(1)	0.74(12)
C(7)-C(8)-O(1)-P(1)	-3.3(3)
C(9)-C(8)-O(1)-P(1)	176.05(16)
C(19)-C(2)-O(2)-P(1)	6.1(2)
C(3)-C(2)-O(2)-P(1)	-171.06(15)

C(17)-C(18)-O(4)-B(1)	-179.3(2)
C(13)-C(18)-O(4)-B(1)	0.7(2)
O(5)-B(1)-O(4)-C(18)	-1.2(2)
N(1)-B(1)-O(4)-C(18)	-133.44(19)
N(2)-B(1)-O(4)-C(18)	123.07(19)
C(32)-C(31)-O(3)-P(1)	166.2(3)
C(14)-C(13)-O(5)-B(1)	178.8(2)
C(18)-C(13)-O(5)-B(1)	-1.0(2)
O(4)-B(1)-O(5)-C(13)	1.3(2)
N(1)-B(1)-O(5)-C(13)	133.6(2)
N(2)-B(1)-O(5)-C(13)	-123.94(19)
C(31)-O(3)-P(1)-N(1)	173.9(2)
C(31)-O(3)-P(1)-O(1)	-63.3(2)
C(31)-O(3)-P(1)-O(2)	50.6(2)
C(1)-N(1)-P(1)-O(3)	-0.6(2)
B(1)-N(1)-P(1)-O(3)	-179.47(14)
C(1)-N(1)-P(1)-O(1)	-100.8(2)
B(1)-N(1)-P(1)-O(1)	80.37(16)
C(1)-N(1)-P(1)-O(2)	100.4(2)
B(1)-N(1)-P(1)-O(2)	-78.47(16)
C(1)-N(1)-P(1)-N(2)	179.6(2)
B(1)-N(1)-P(1)-N(2)	0.79(13)
C(8)-O(1)-P(1)-O(3)	-171.79(16)
C(8)-O(1)-P(1)-N(1)	-69.49(18)
C(8)-O(1)-P(1)-O(2)	91.02(17)
C(8)-O(1)-P(1)-N(2)	4.50(16)
C(2)-O(2)-P(1)-O(3)	170.35(15)
C(2)-O(2)-P(1)-N(1)	67.57(18)
C(2)-O(2)-P(1)-O(1)	-92.87(15)
C(2)-O(2)-P(1)-N(2)	-6.24(15)

Symmetry transformations used to generate equivalent atoms:

VII. Multinuclear NMR Spectra

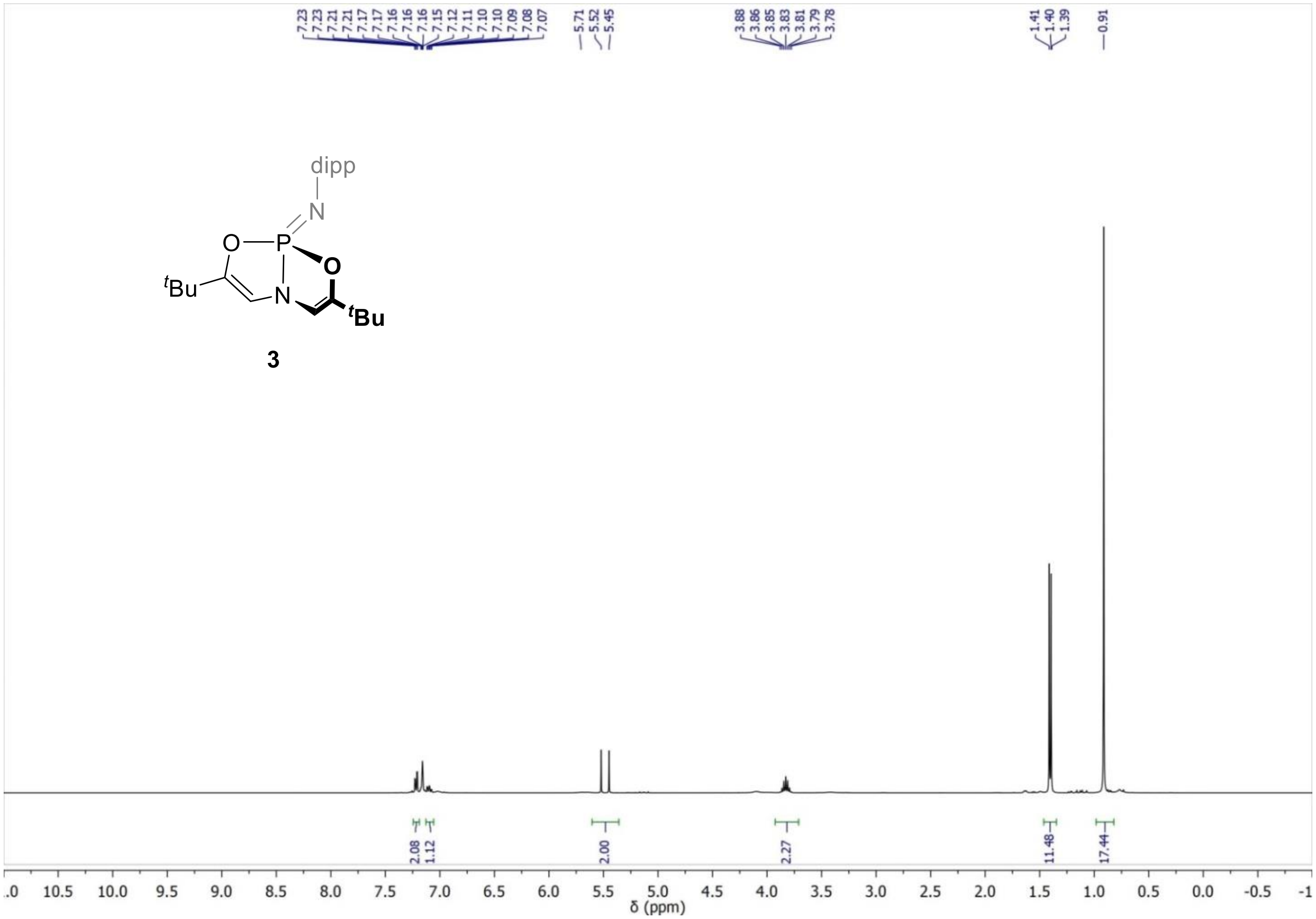

Figure S1. ^{1}H NMR of **3**.

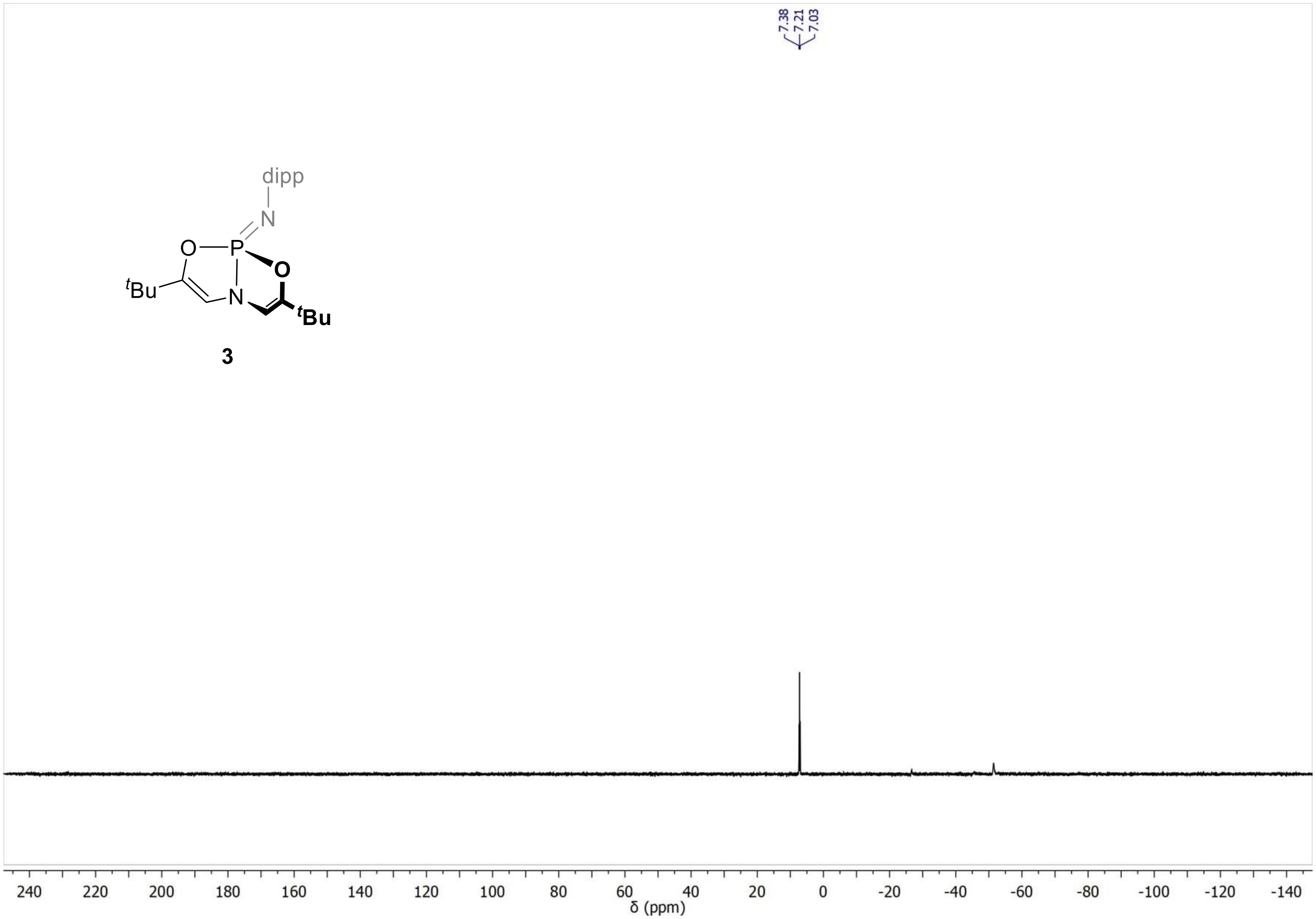

Figure S2. ^{31}P NMR of **3**.

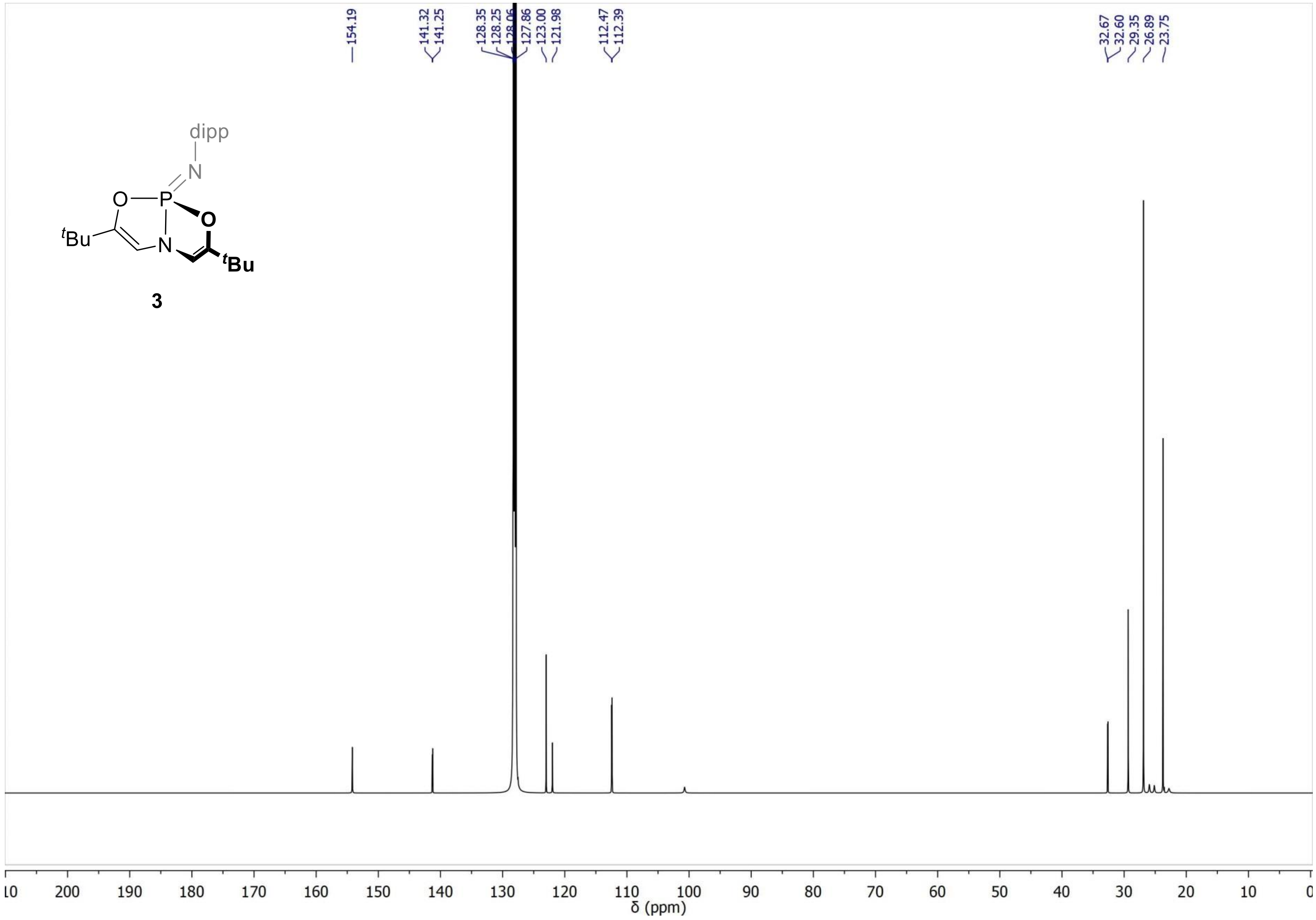

Figure S3. ^{13}C NMR of **3**.

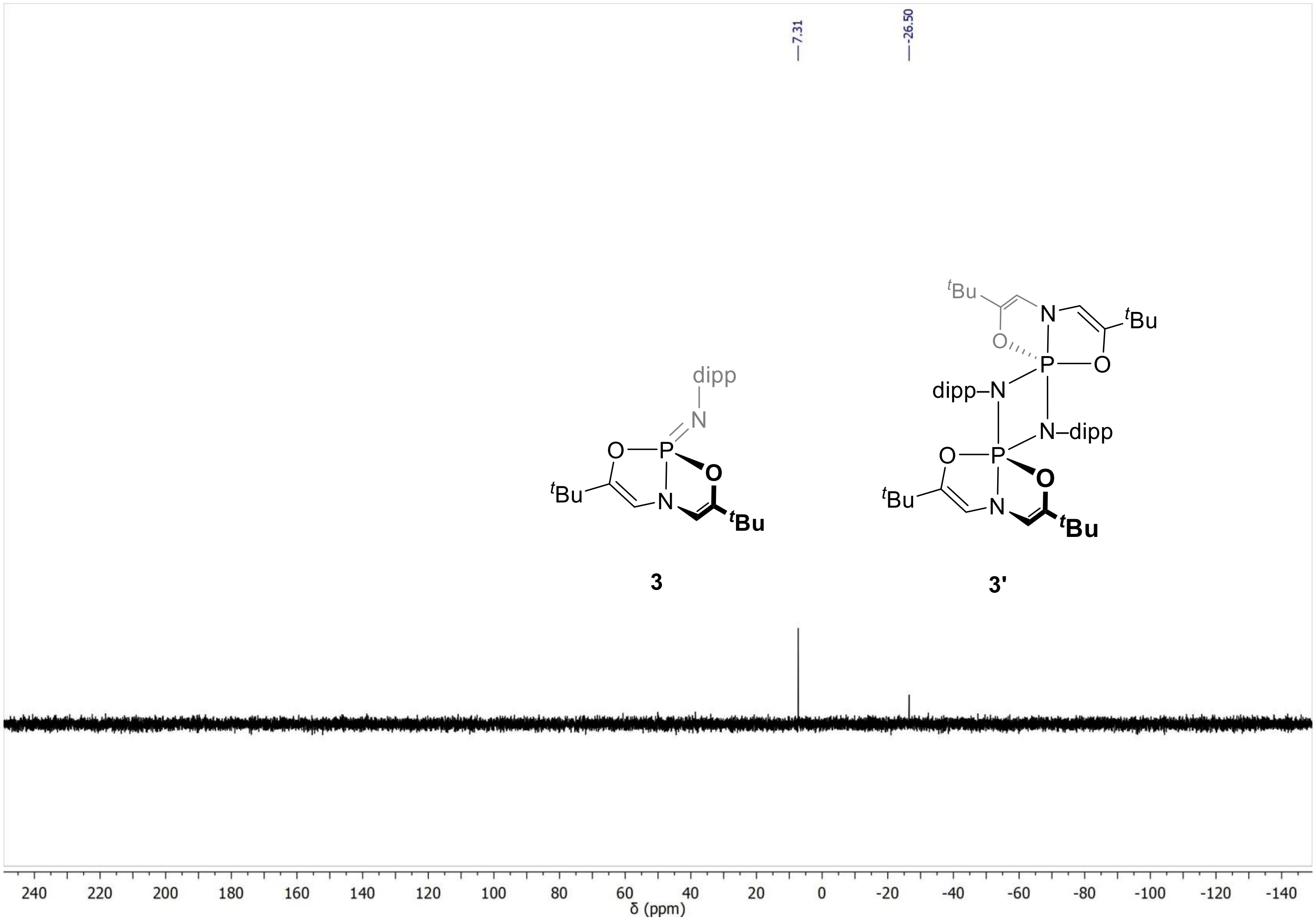

Figure S4. ^{31}P NMR of mixture of **3** and **3’**.

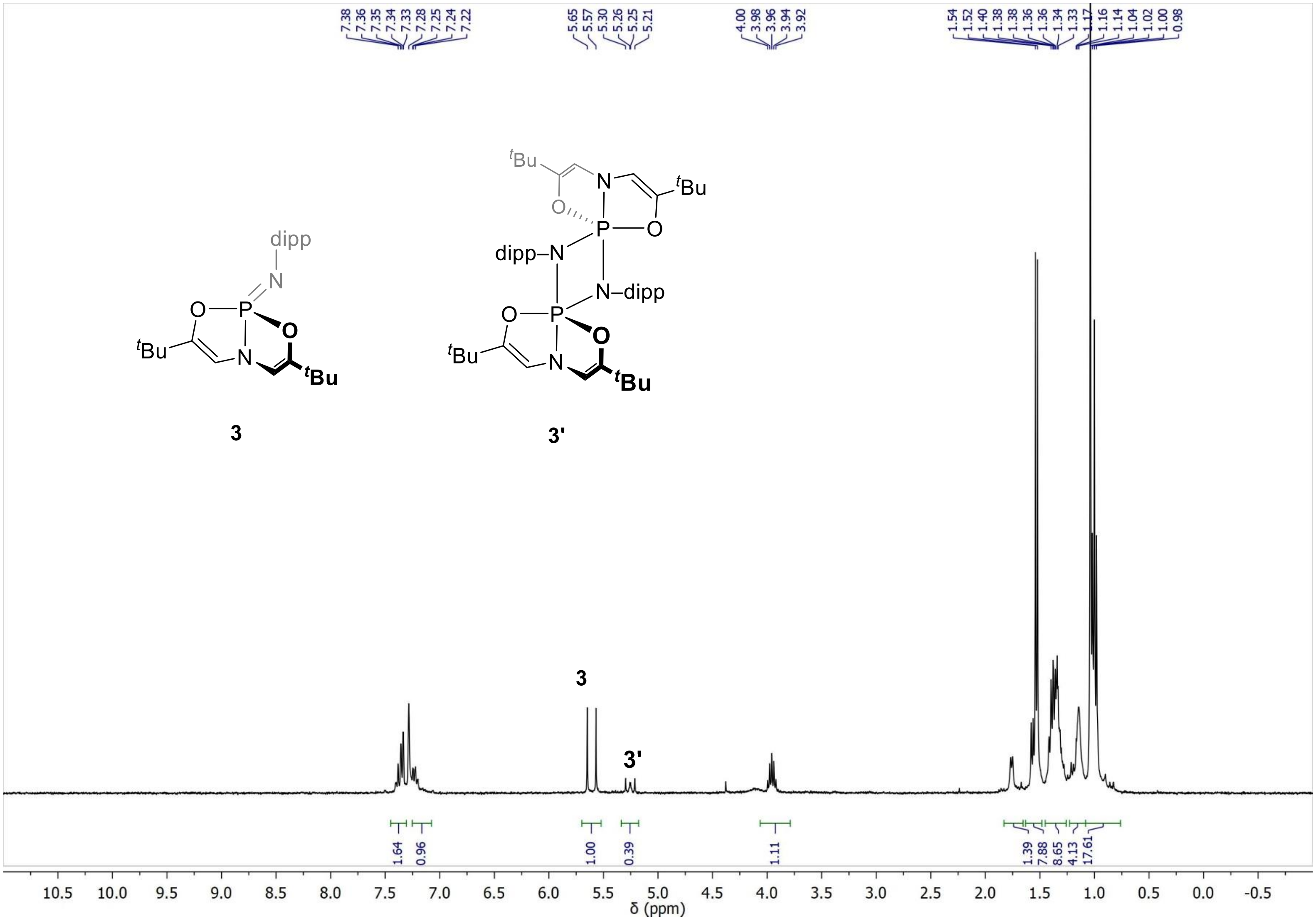

Figure S5. ^{1}H NMR of mixture of **3** and **3'**.

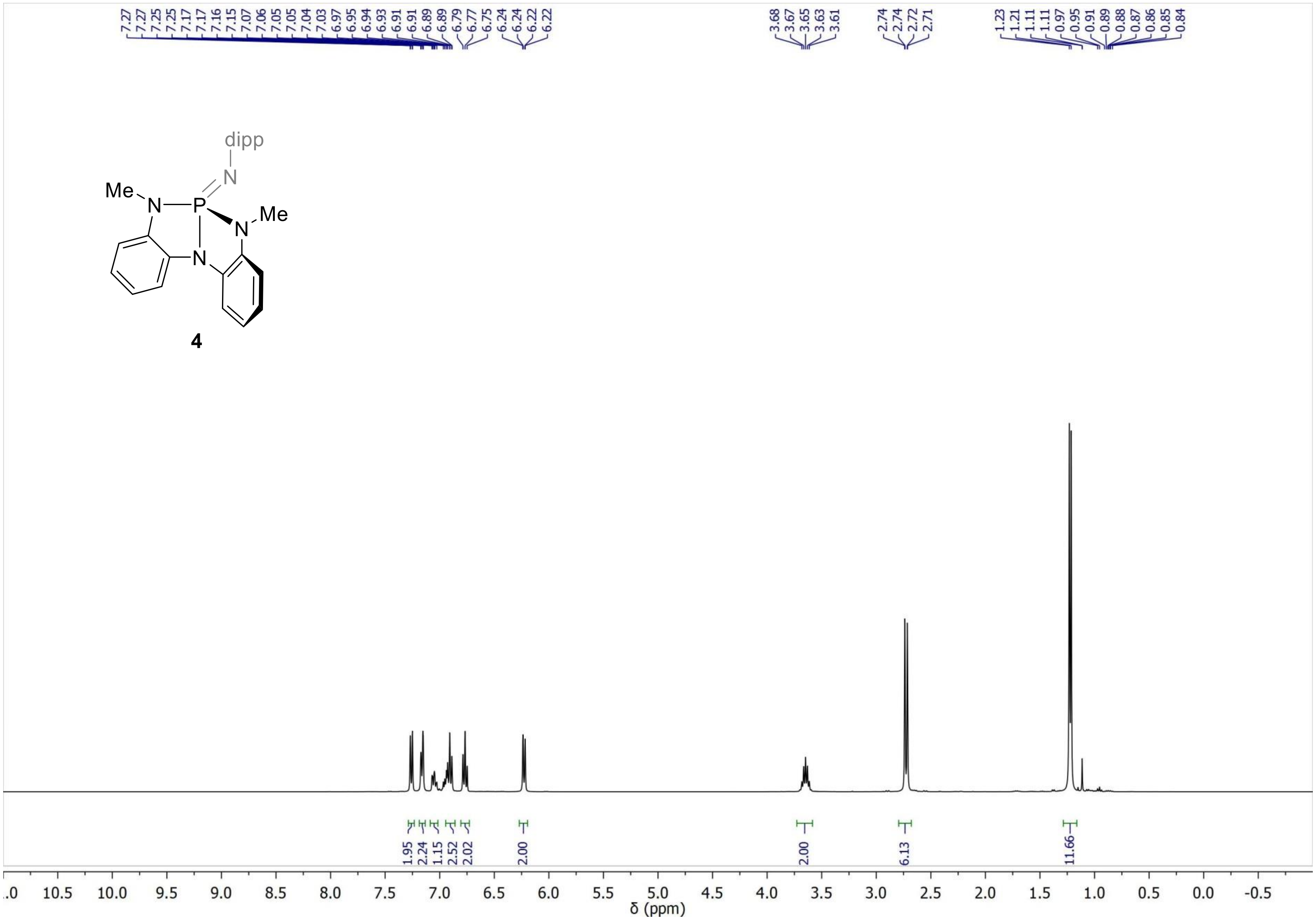

Figure S6. ^{1}H NMR of **4**.

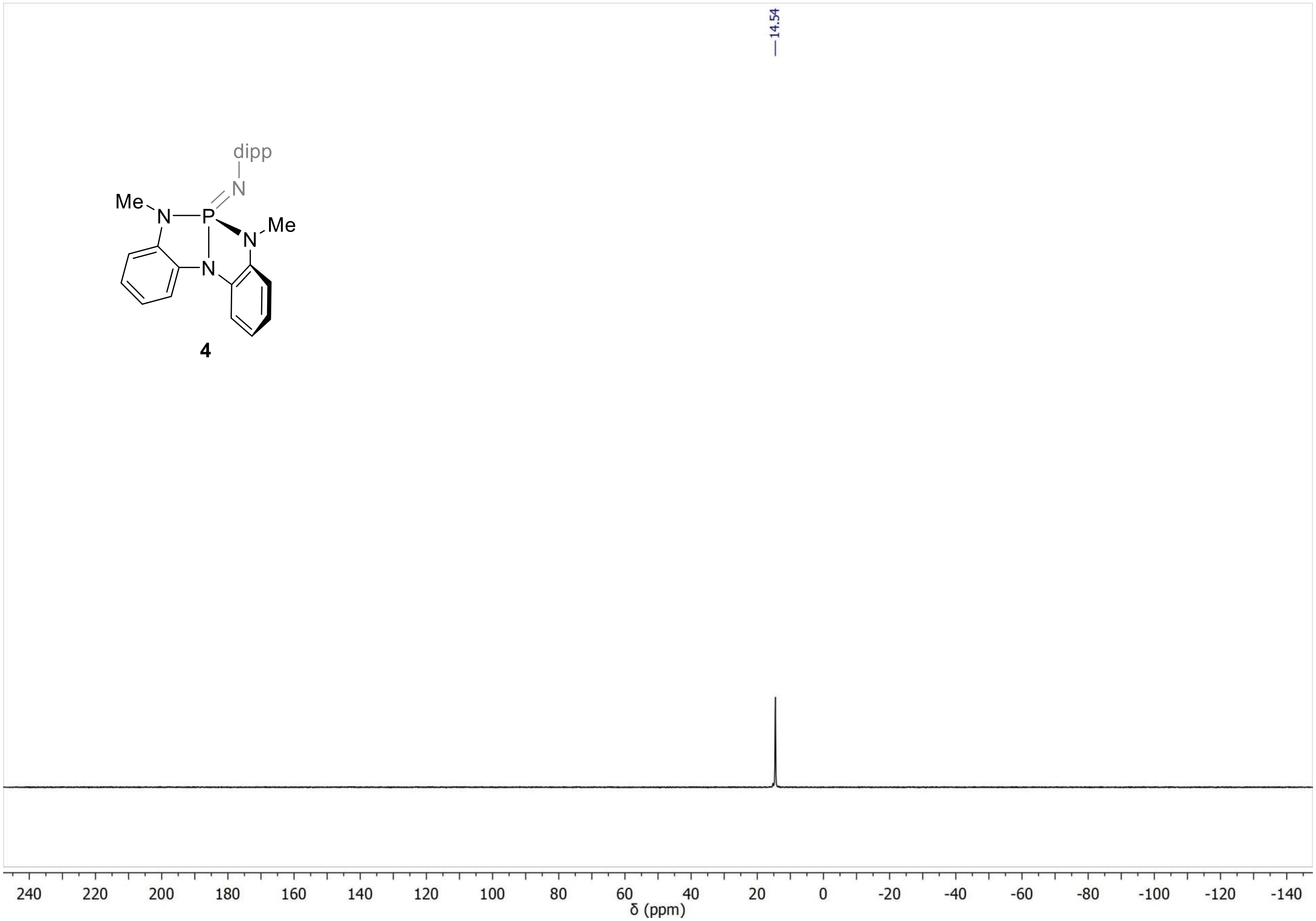

Figure S7. ^{31}P NMR of **4**.

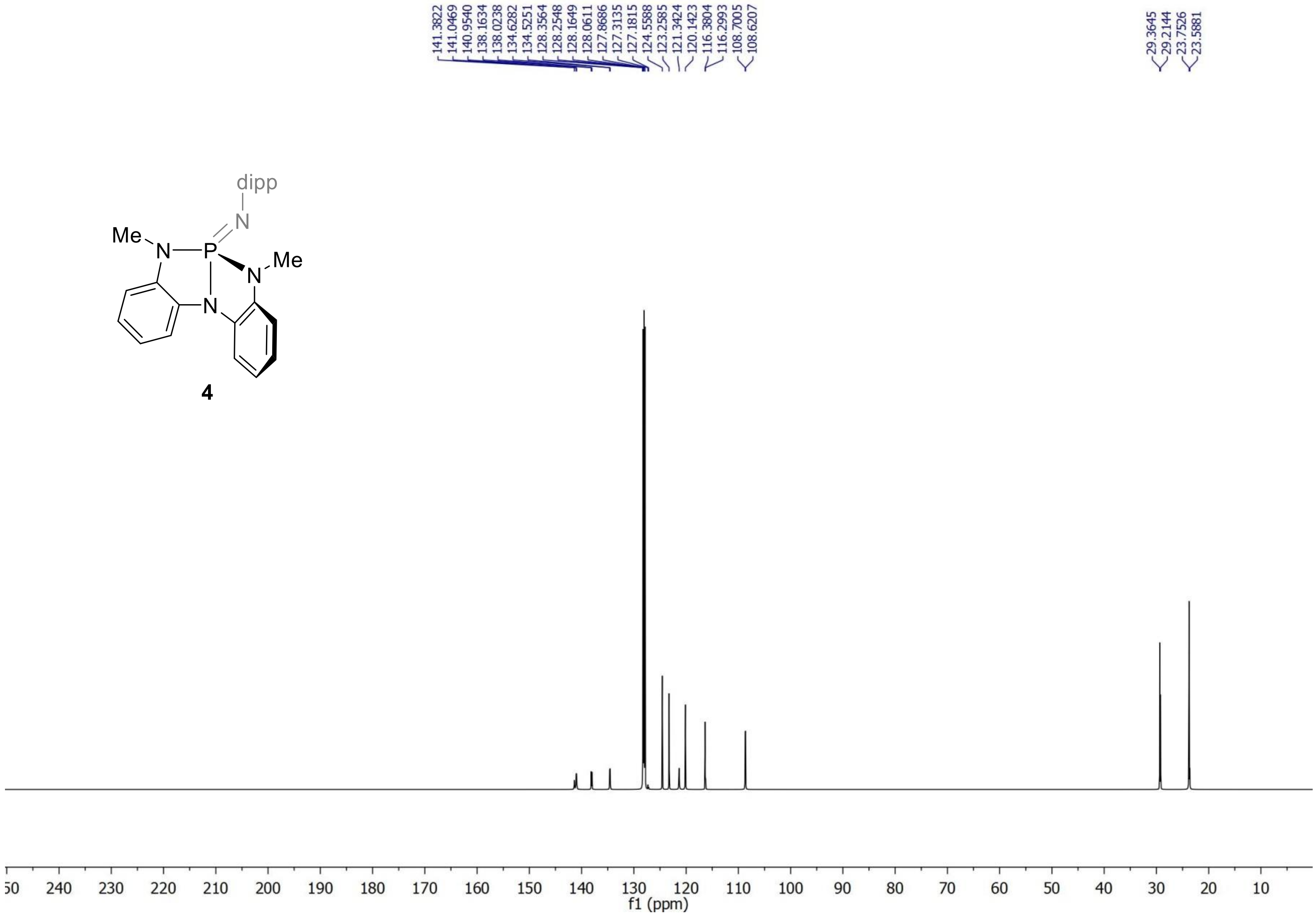

Figure S8. ^{13}C NMR of **4**.

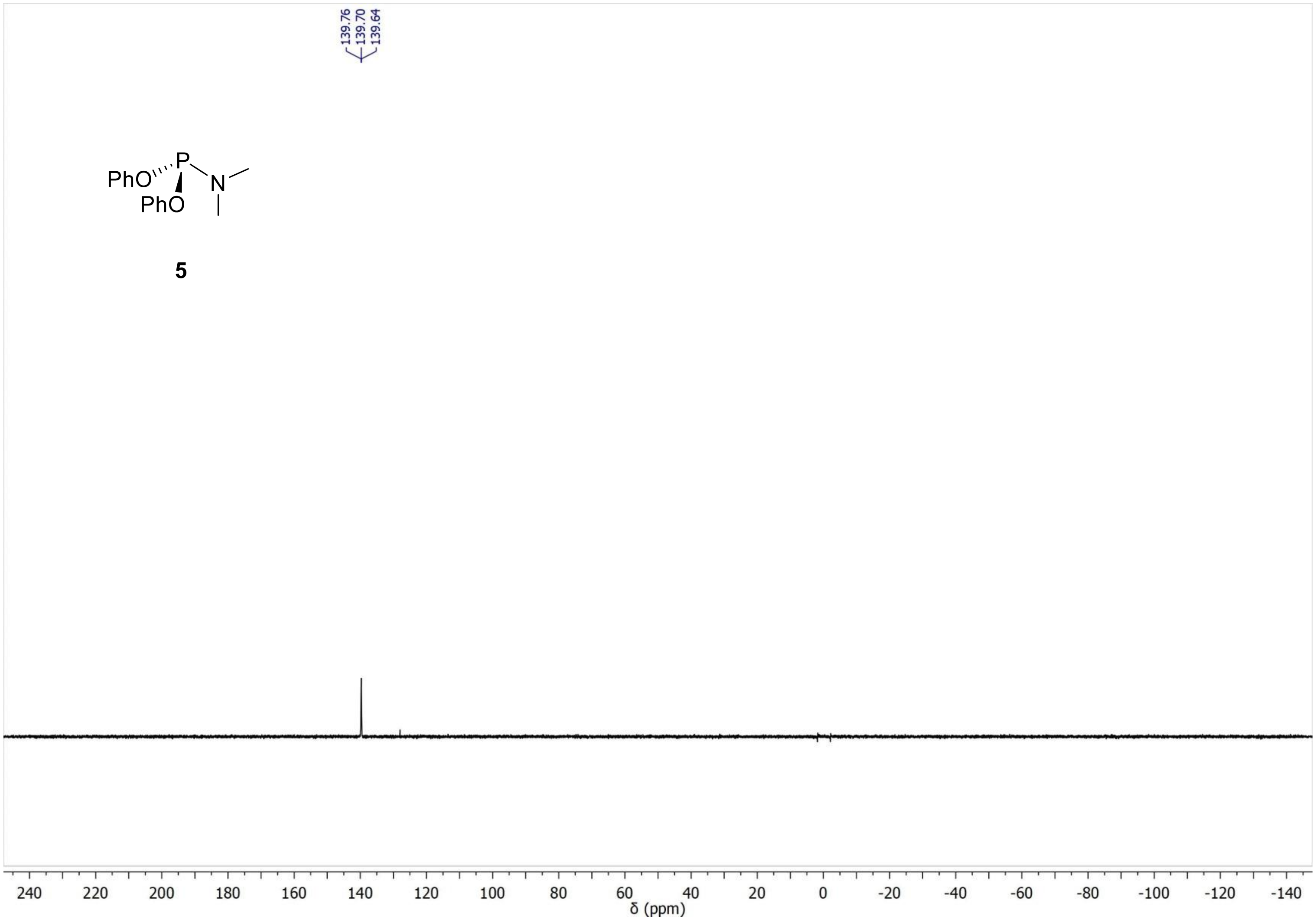

Figure S9. ^{31}P NMR of **5**.

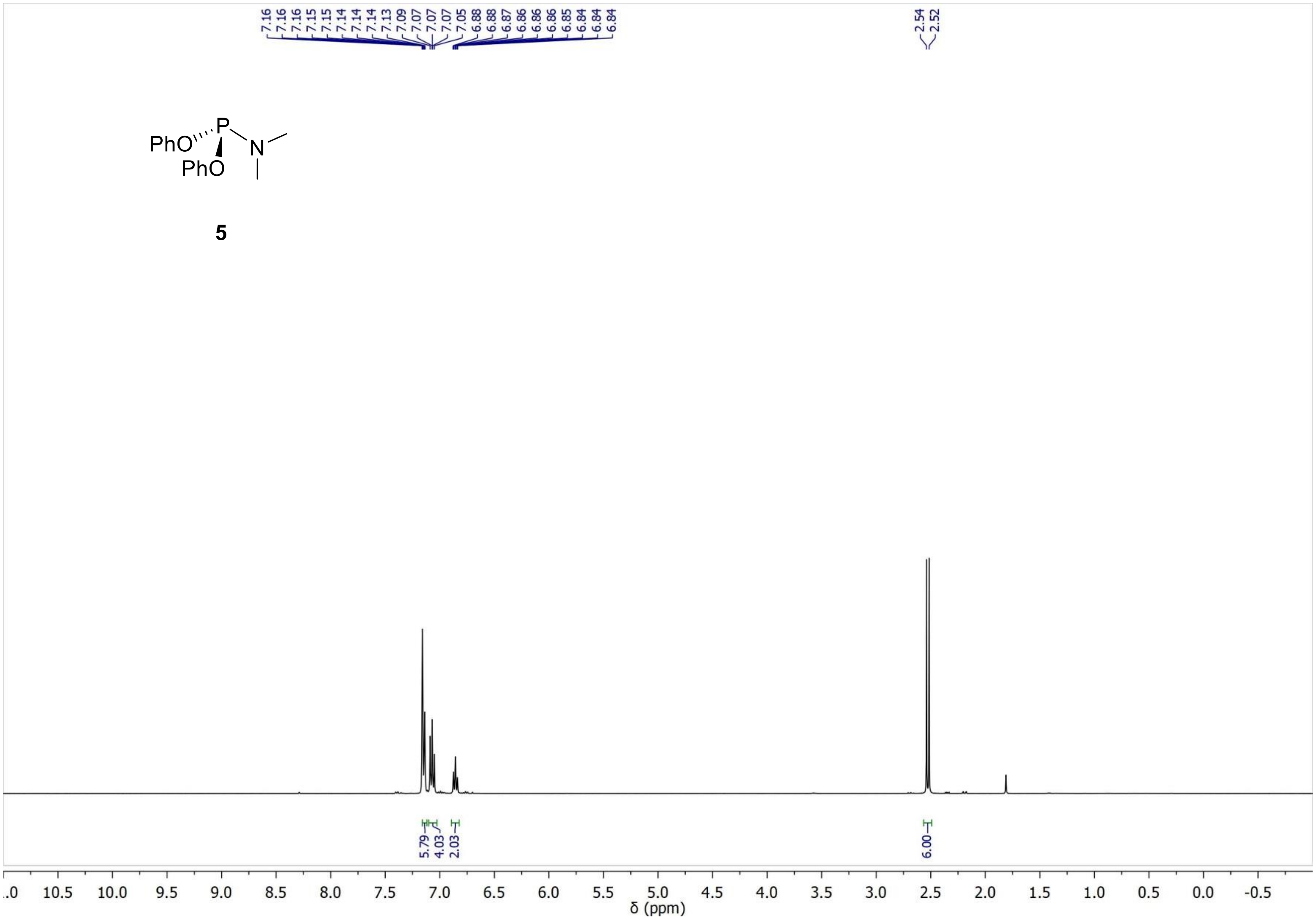

Figure S10. ^{1}H NMR of **5**.

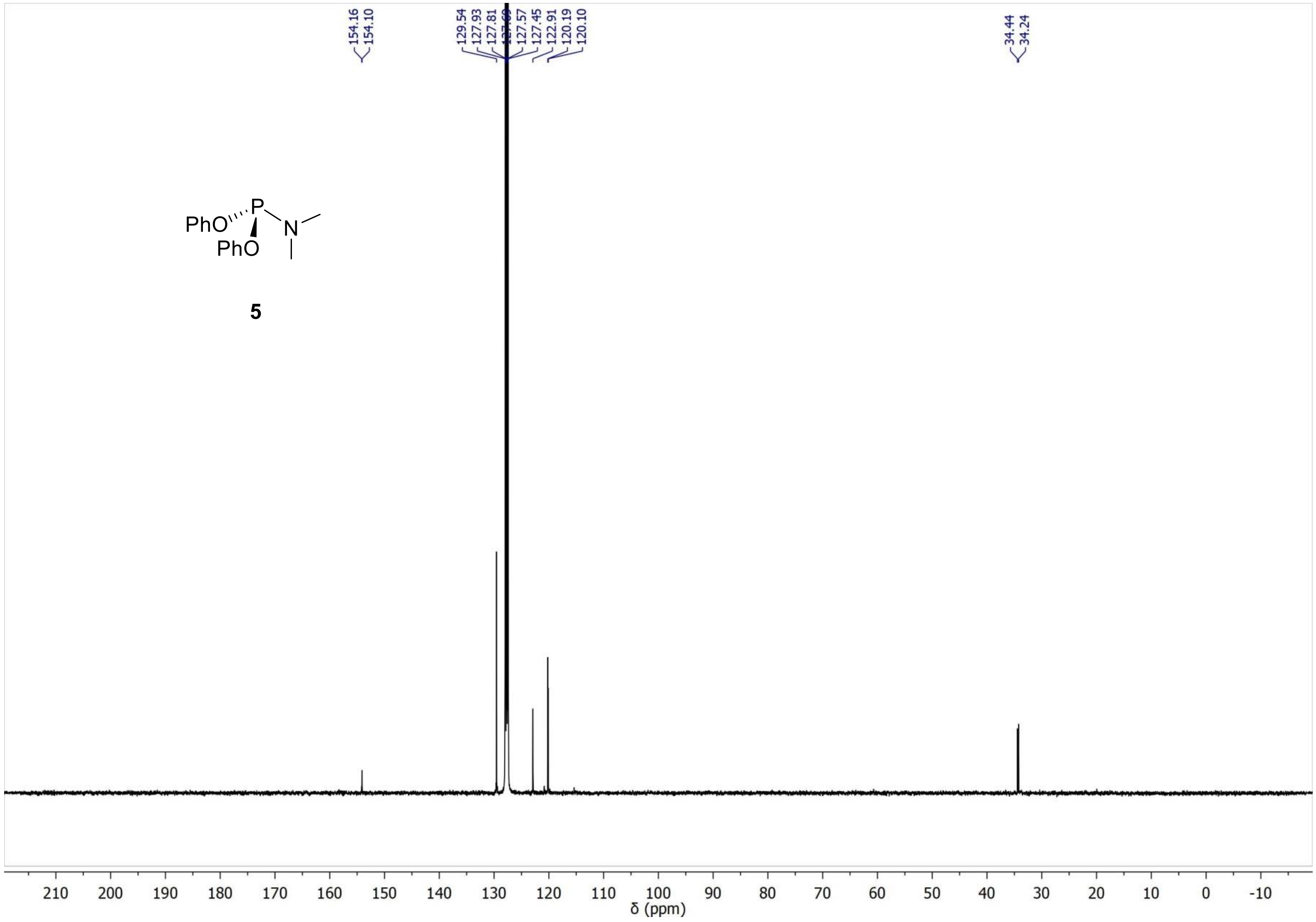

Figure S11. ^{13}C NMR of **5**.

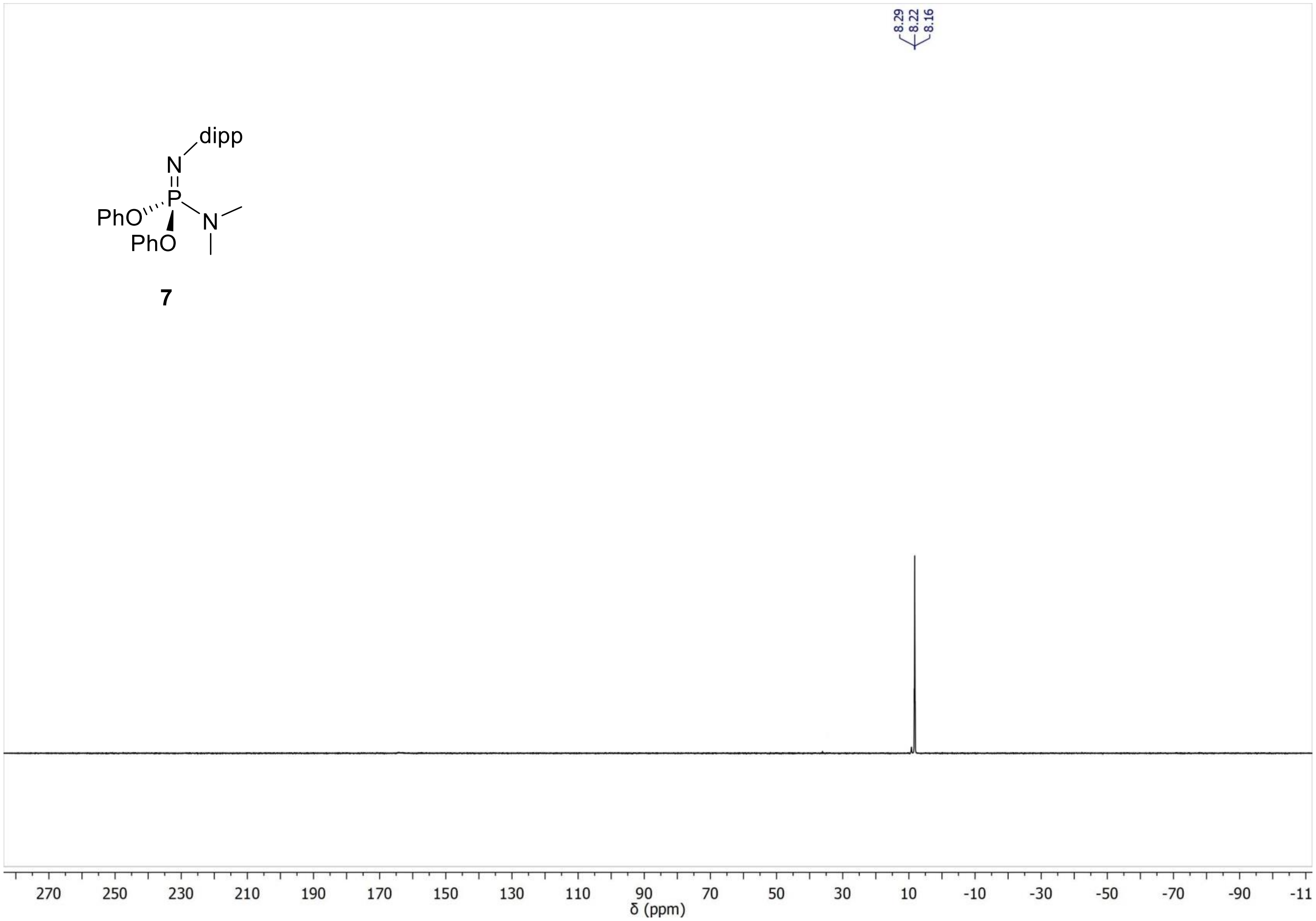

Figure S12. ^{31}P NMR of **7**.

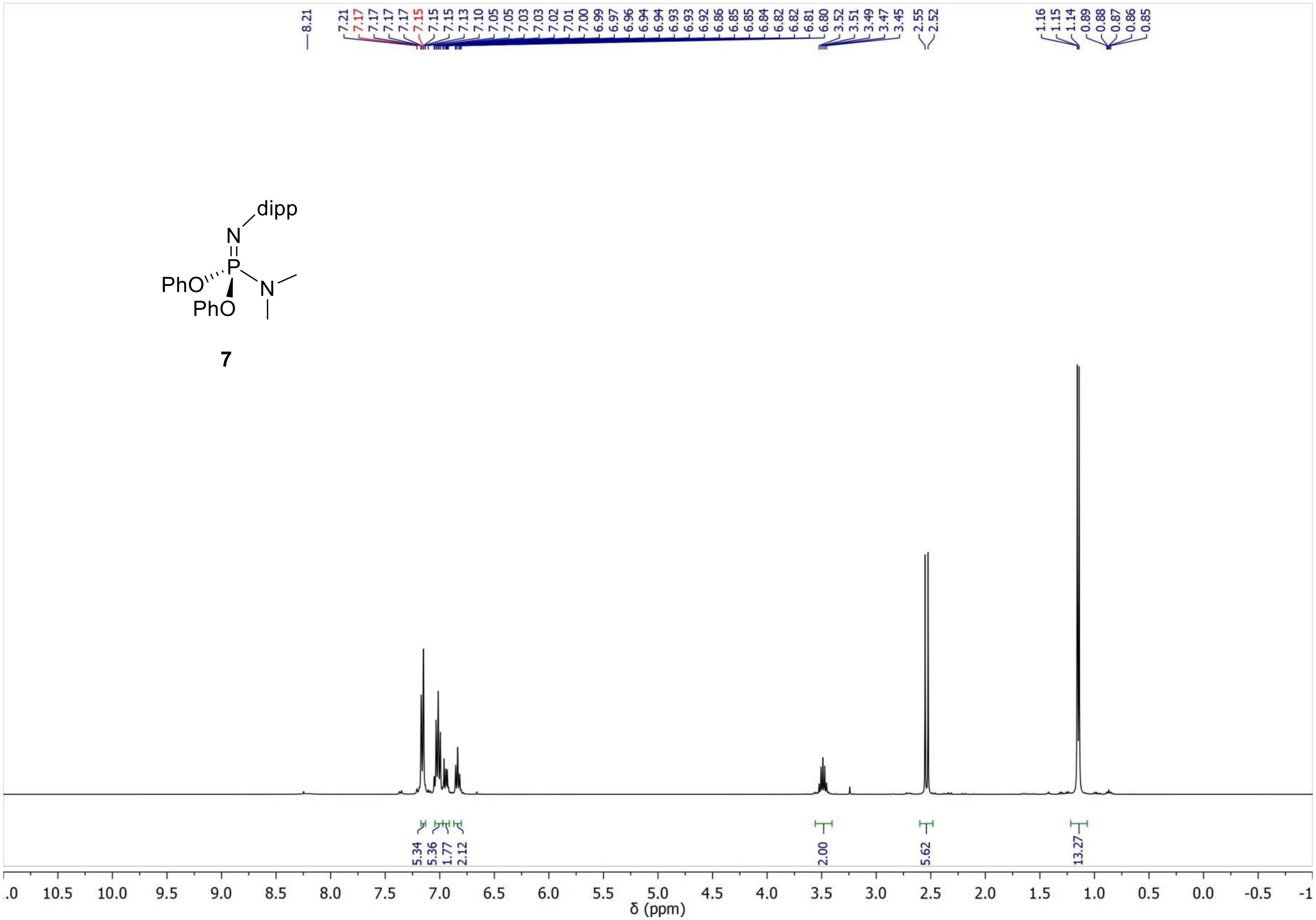

Figure S13. ^{1}H NMR of **7**.

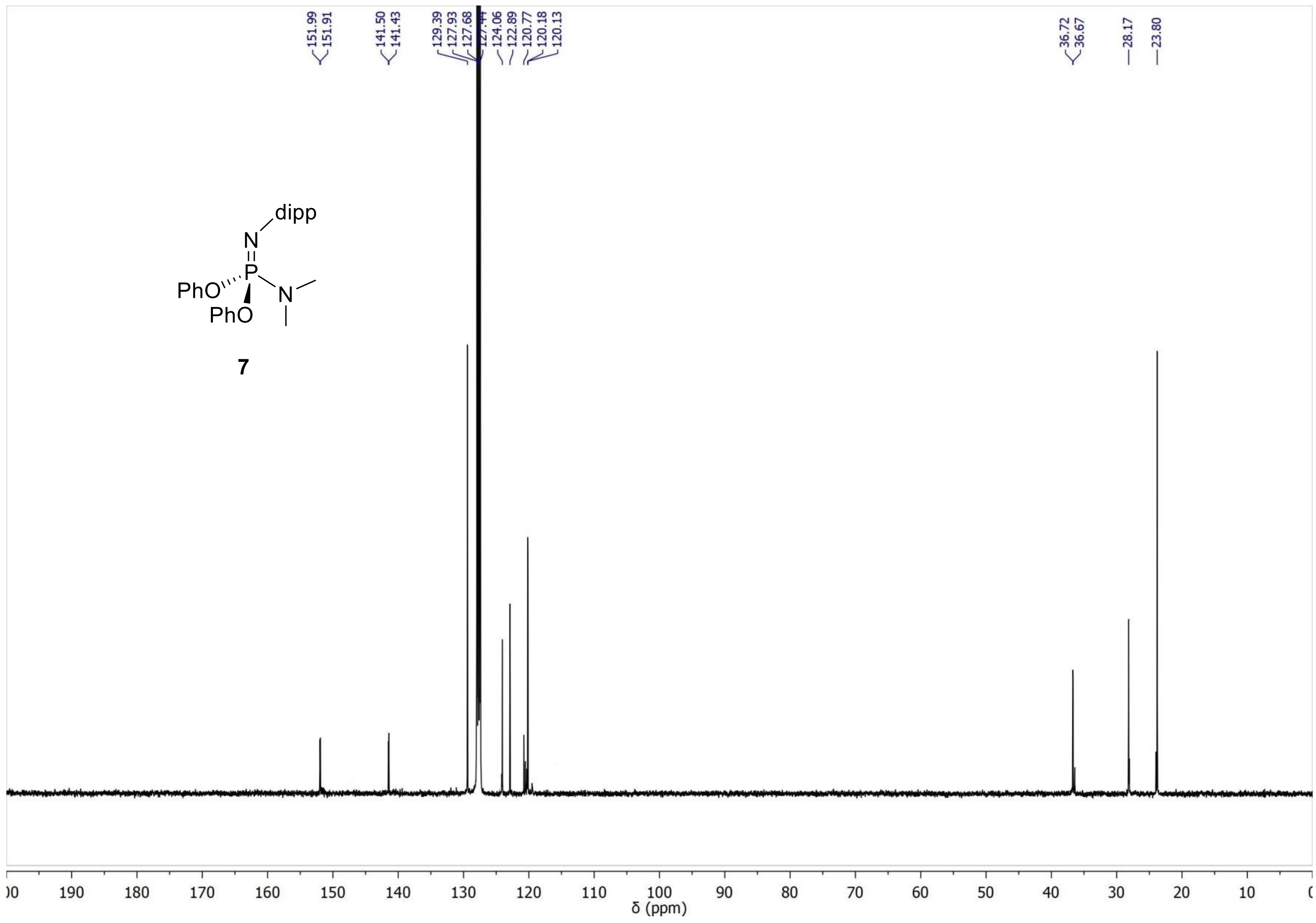

Figure S14. ^{13}C NMR of **7**.

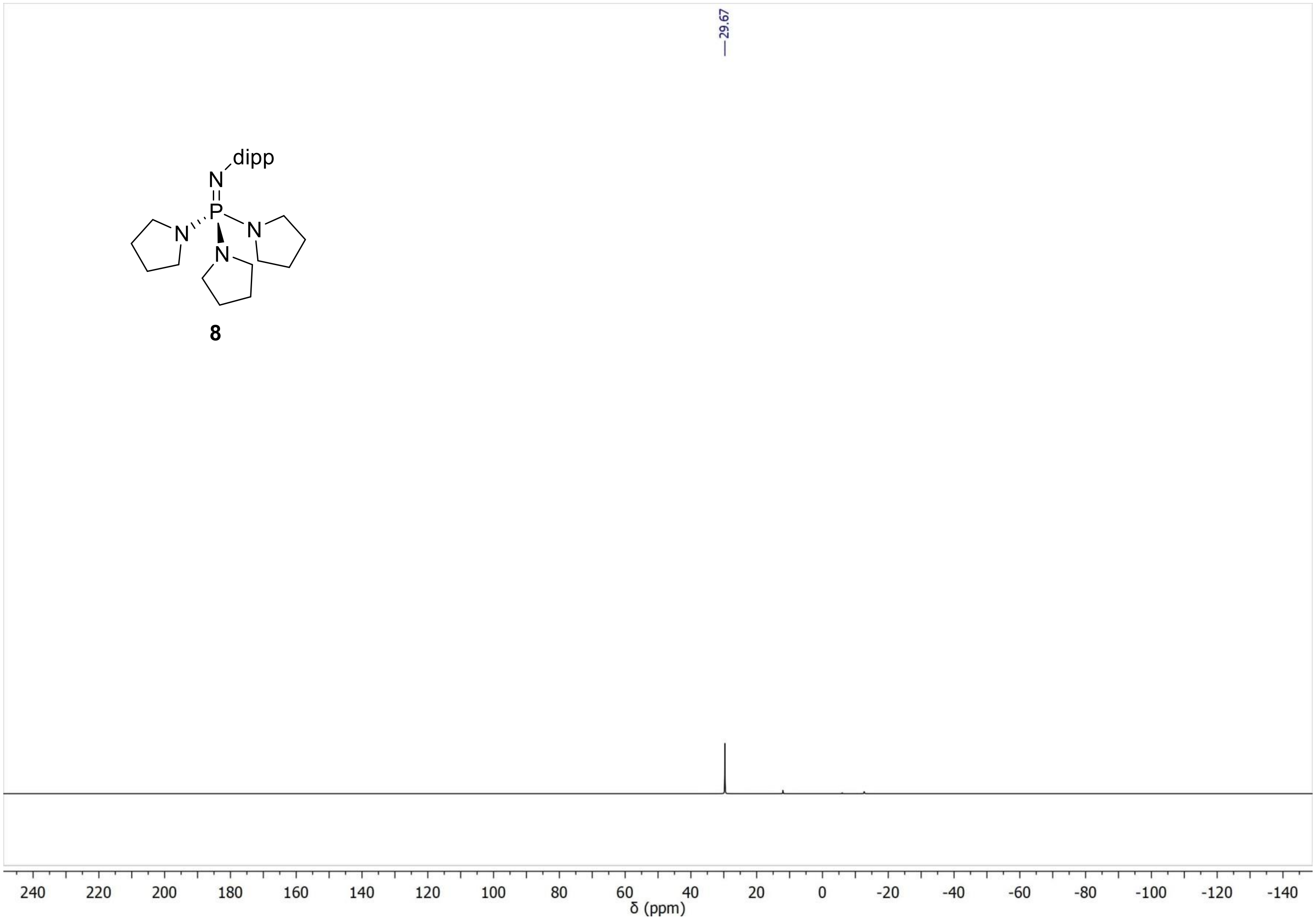

Figure S15. ^{31}P NMR of **8**.

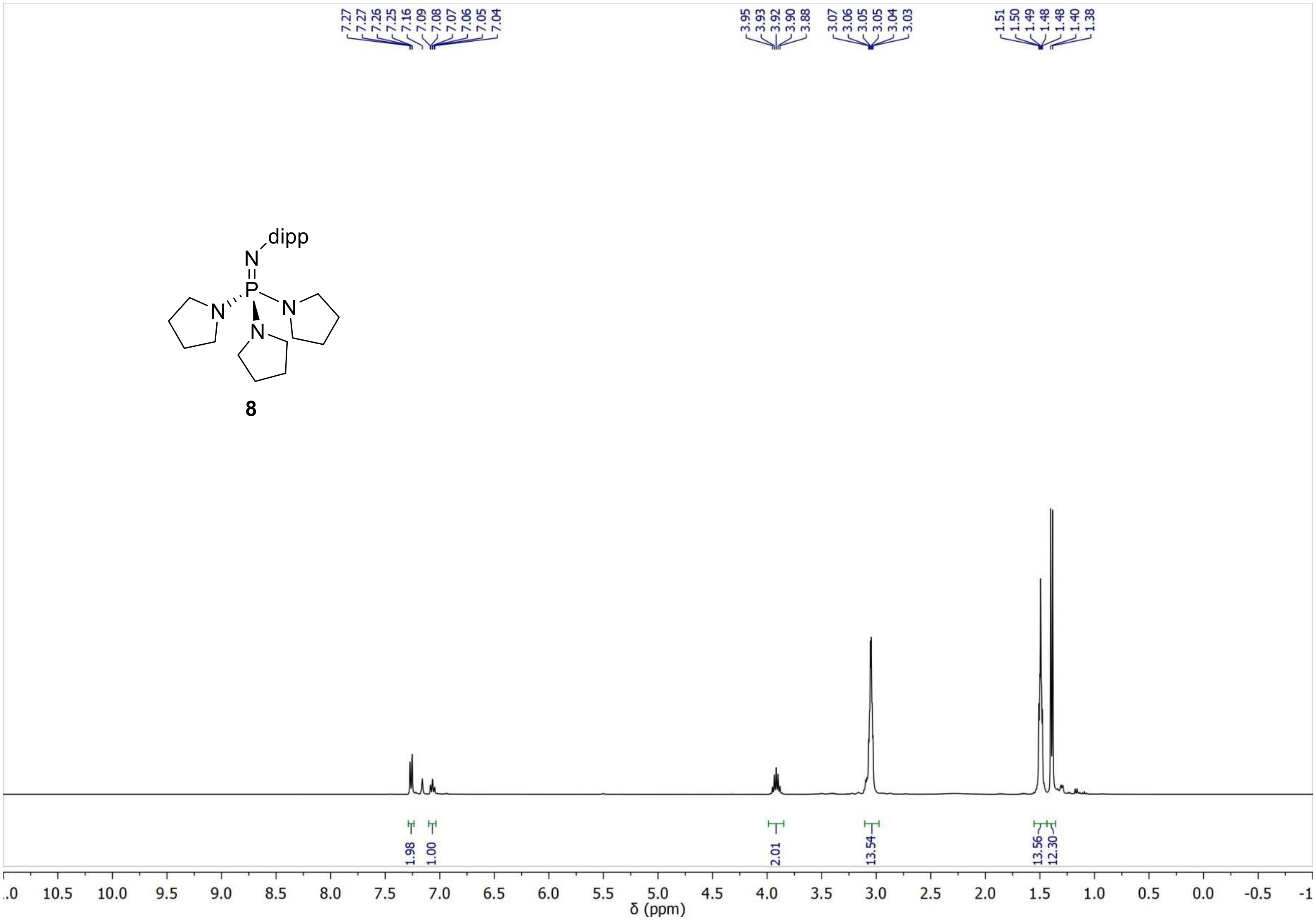

Figure S16. ^{1}H NMR of **8**.

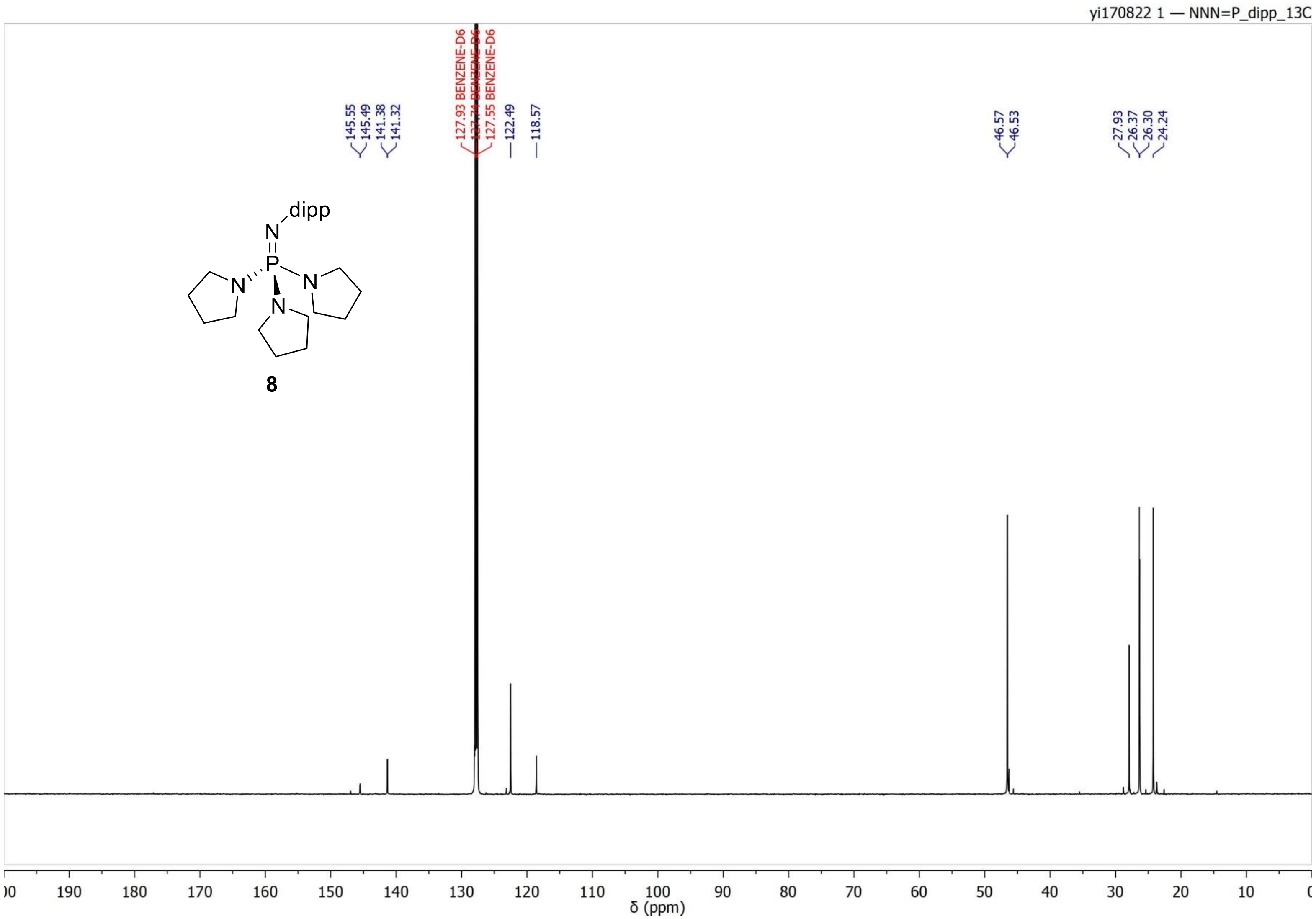

Figure S17. ^{13}C NMR of **8**.

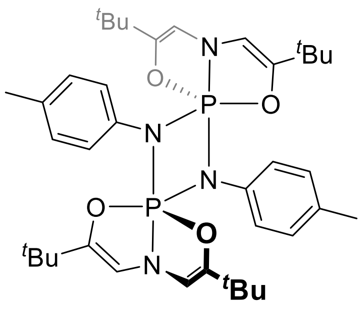

9'

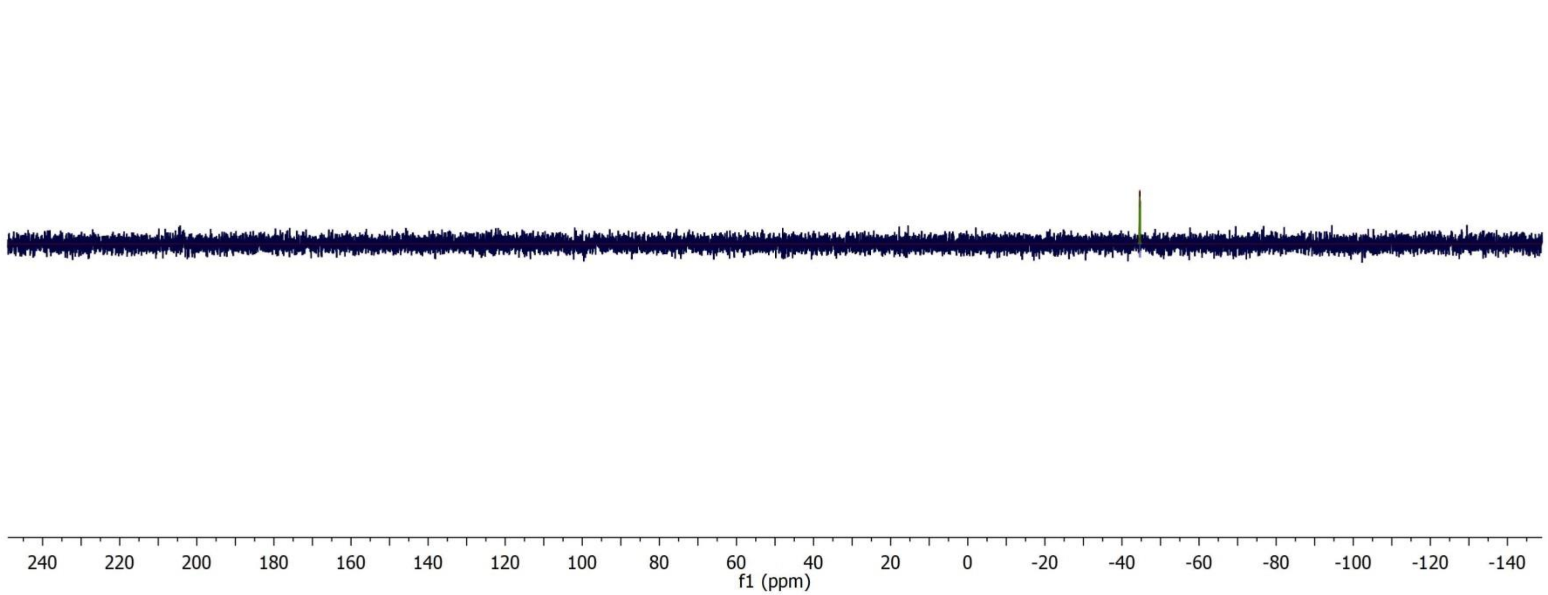

Figure S18. ^{31}P NMR of **9’**.

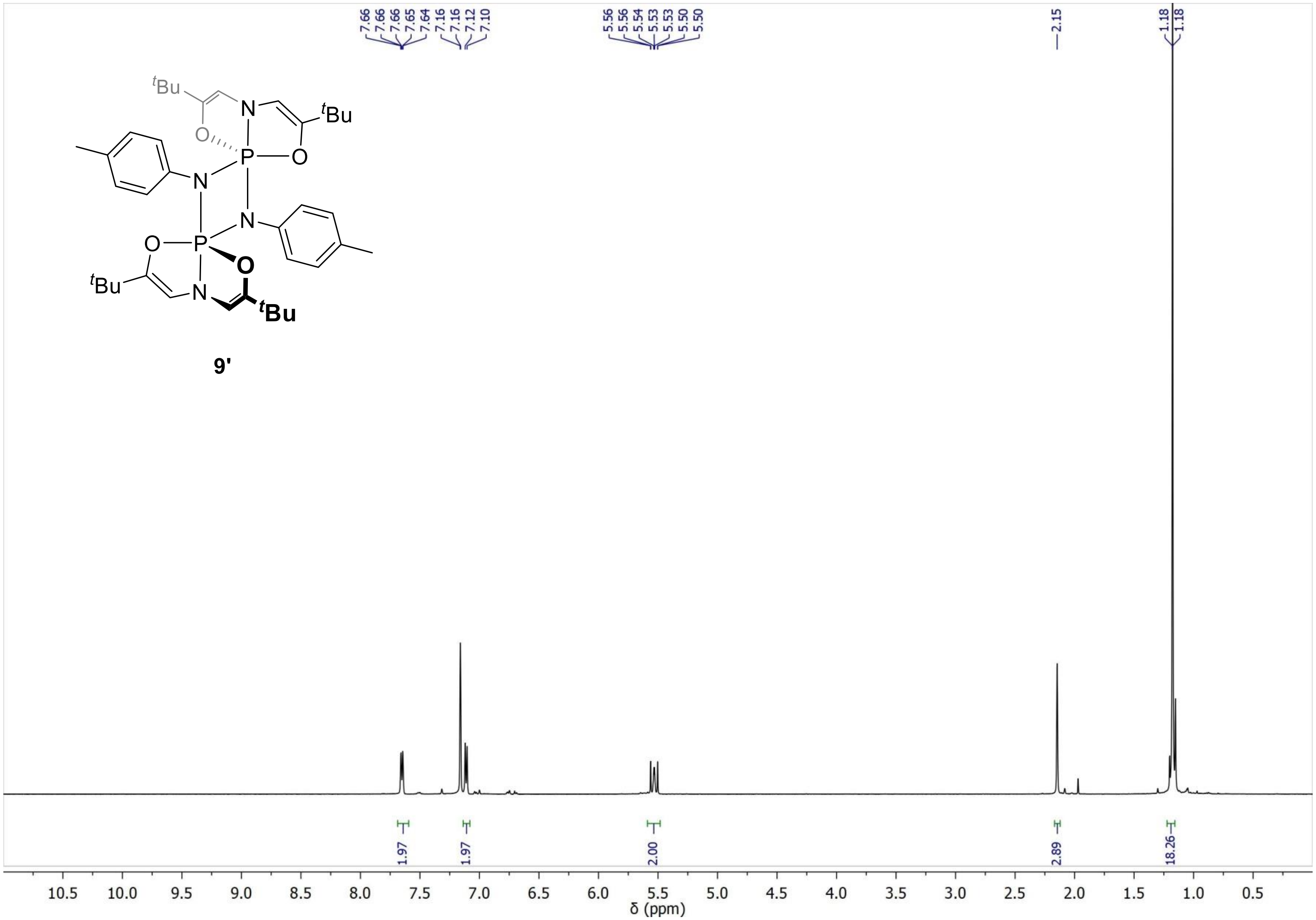

Figure S19. ¹H NMR of **9’**.

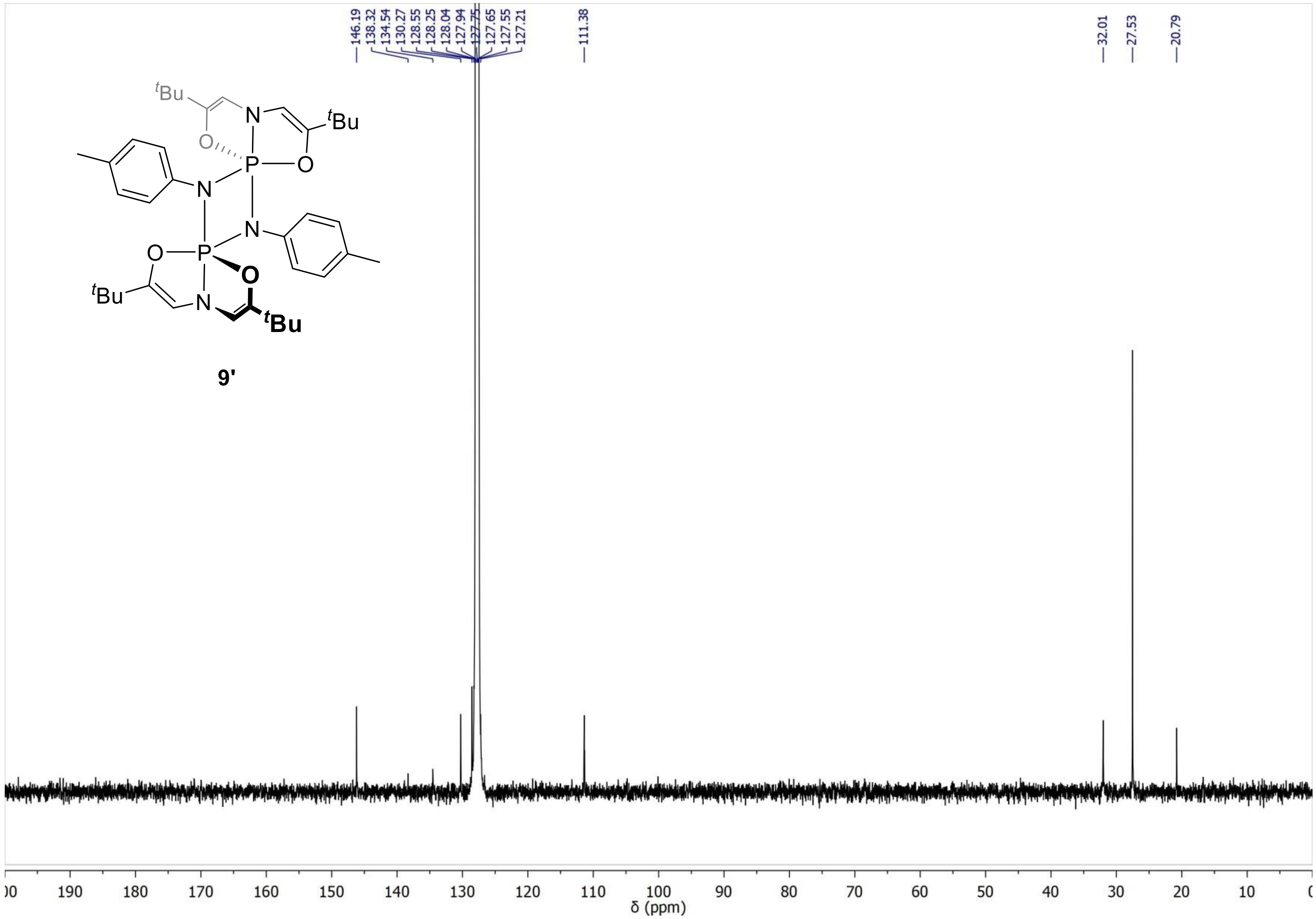

Figure S20. ^{13}C NMR of **9'**.

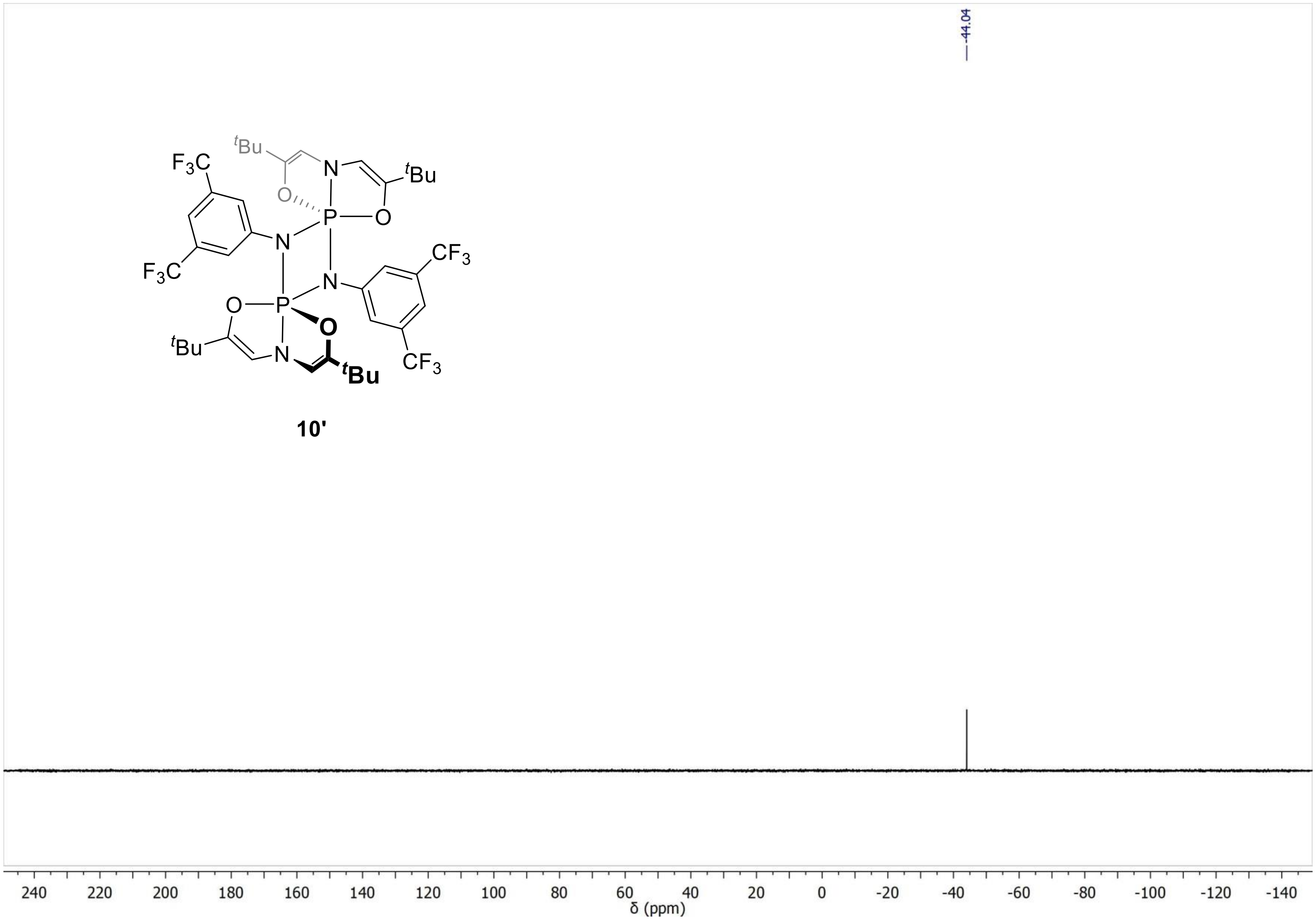

Figure S21. ^{31}P NMR of **10'**.

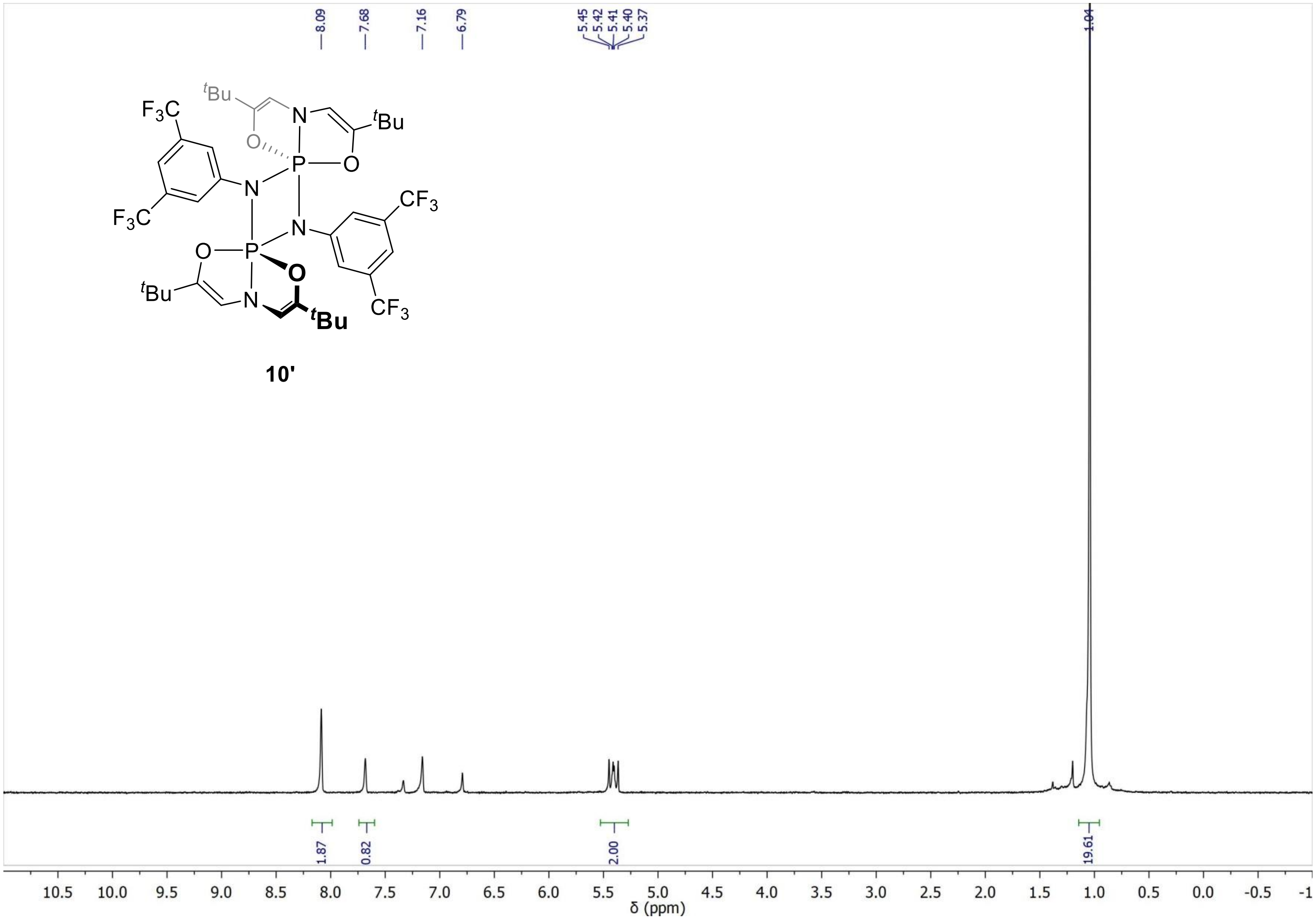

Figure S22. ^{1}H NMR of **10’**.

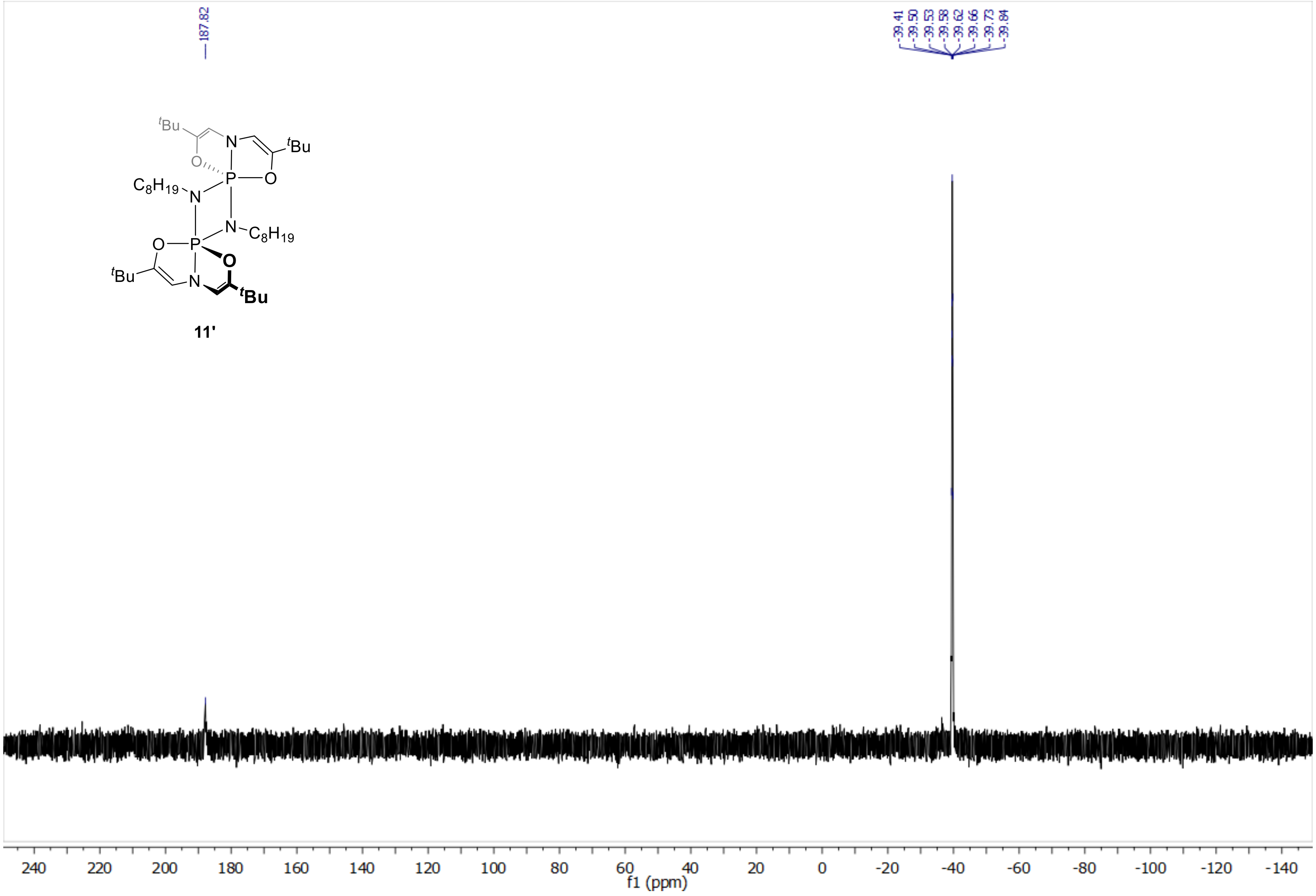

Figure S23. ^{31}P NMR of **11'**.

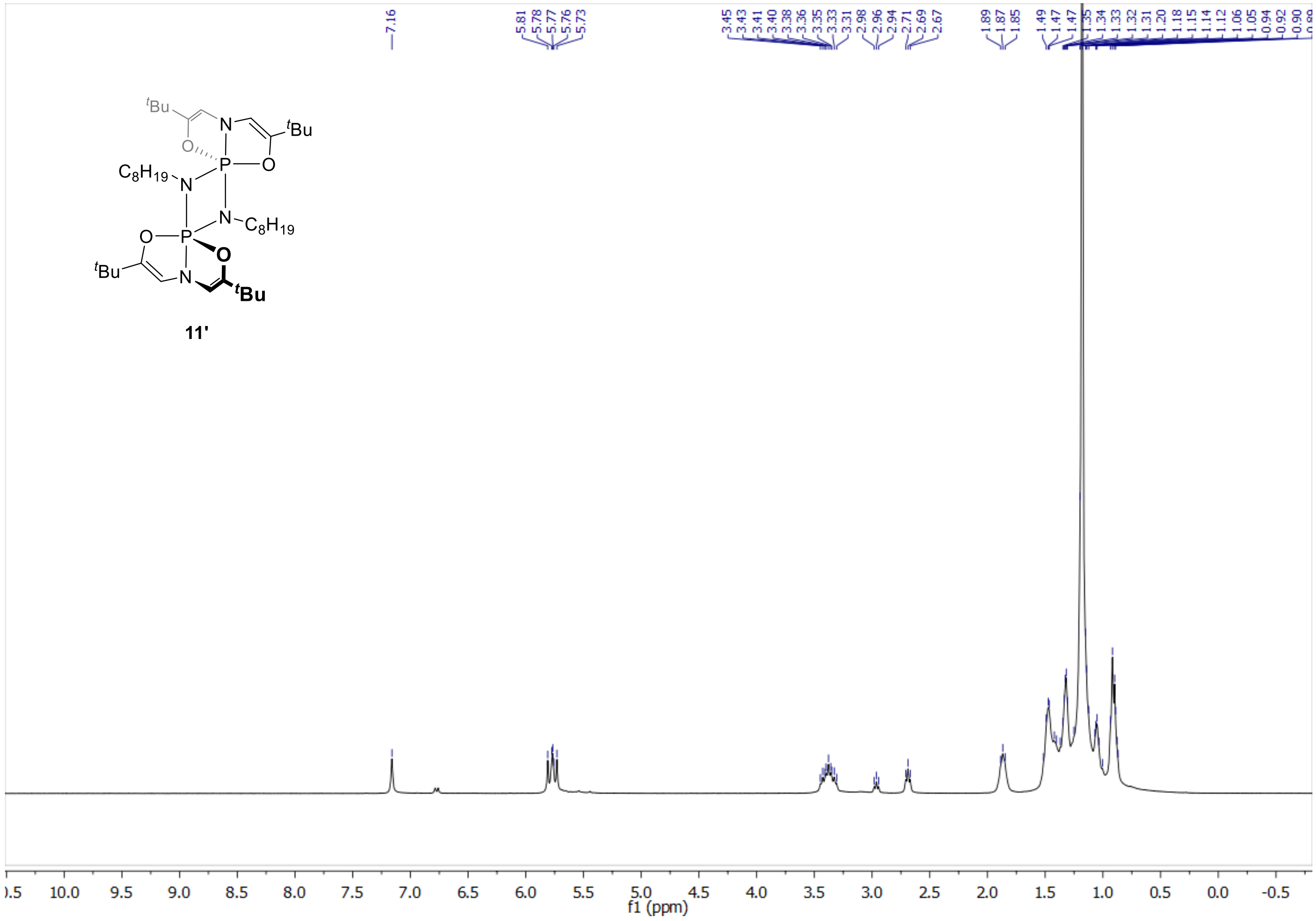

Figure S24. ^{1}H NMR of **11'**.

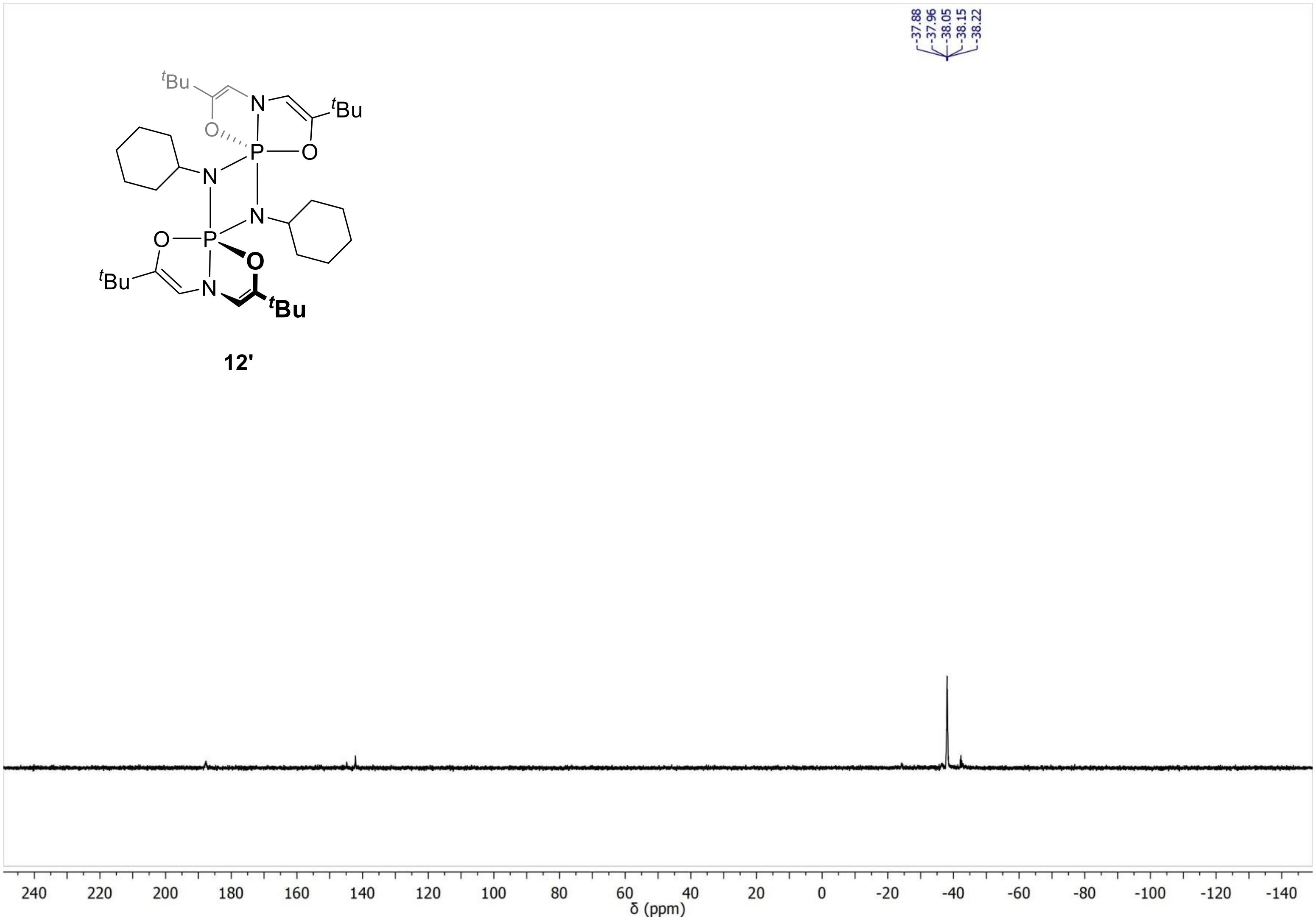

Figure S25. ^{31}P NMR of **12’**.

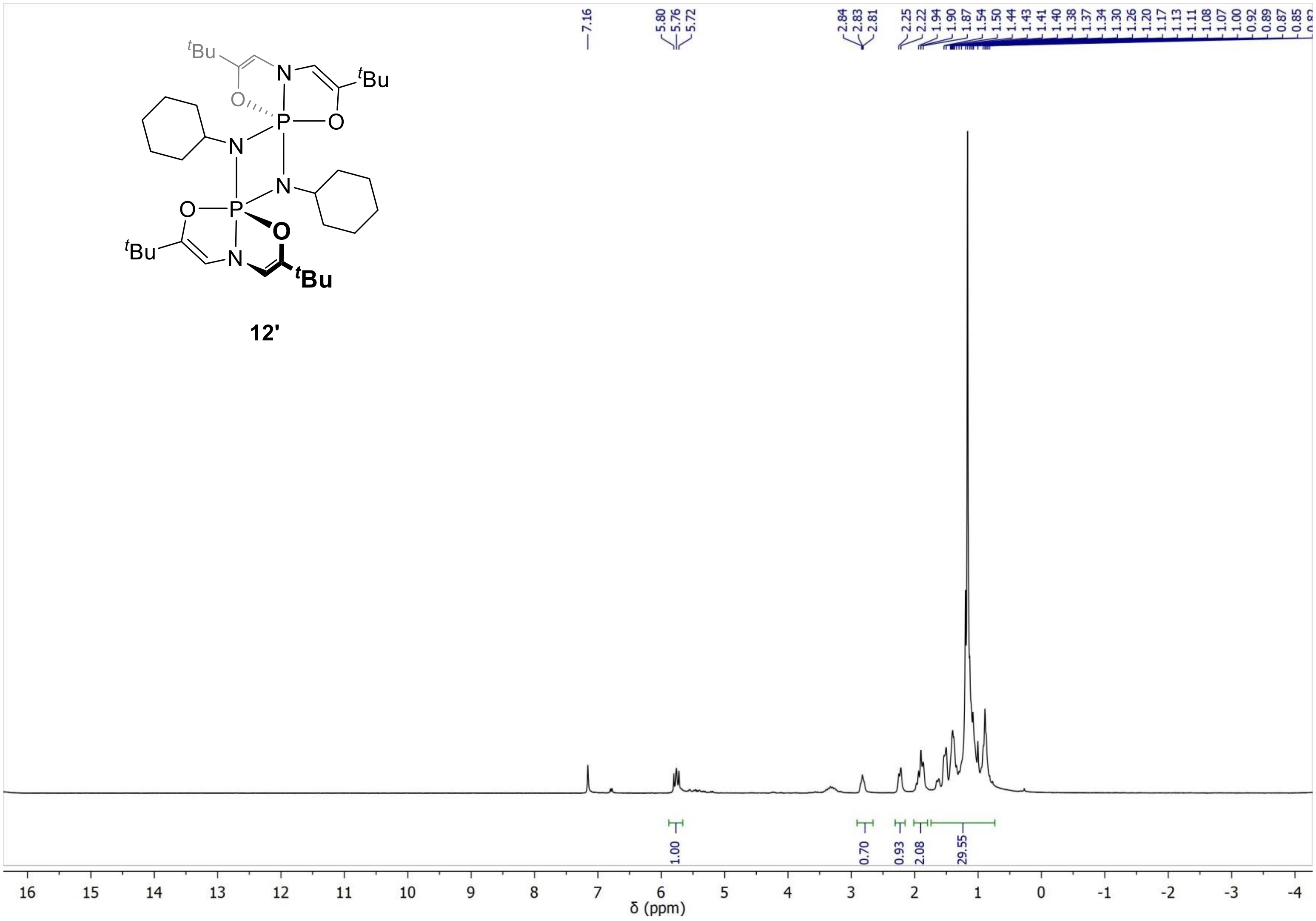

Figure S26. ^{1}H NMR of **12’**.

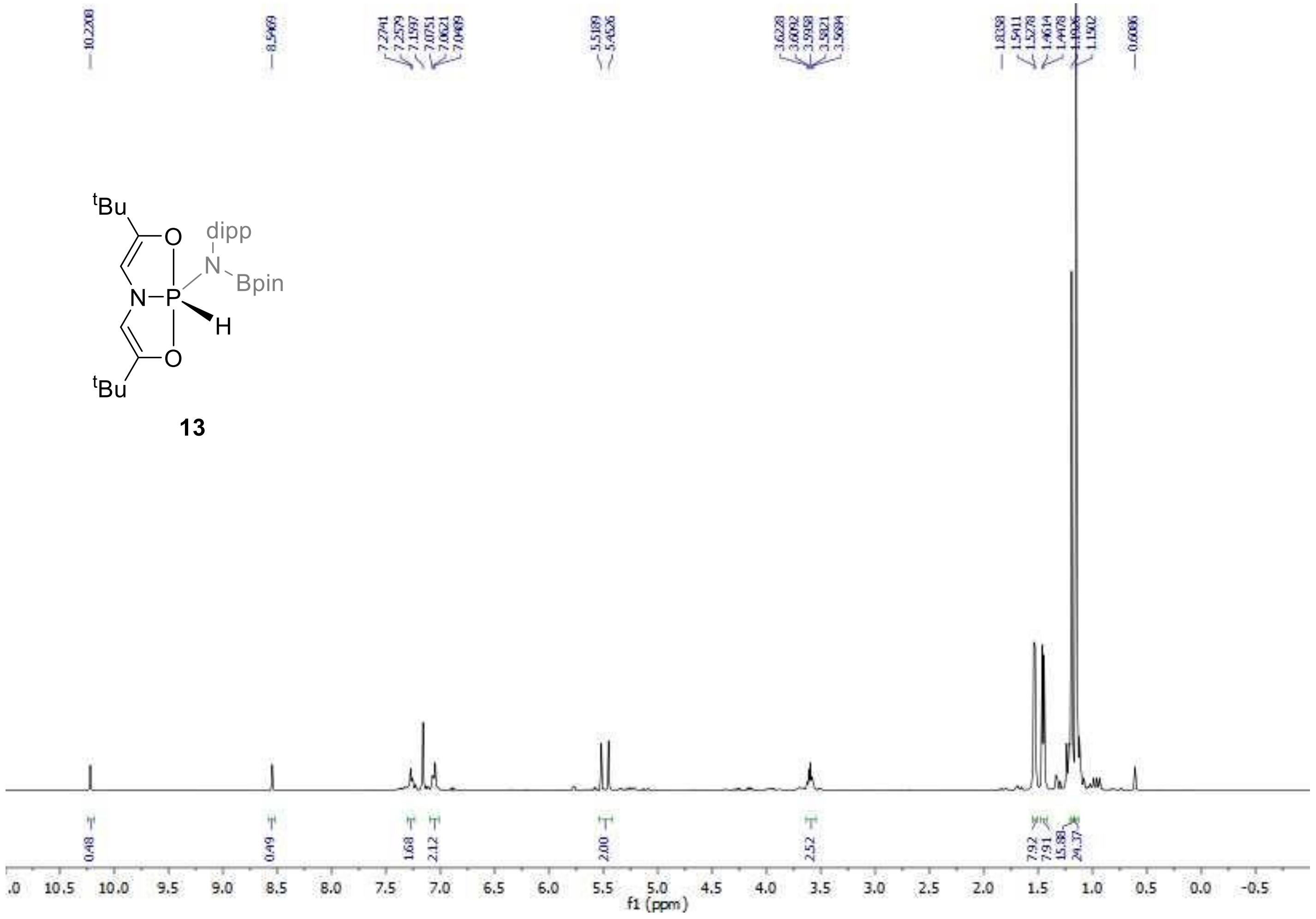

Figure S27. ^{1}H NMR of **13**.

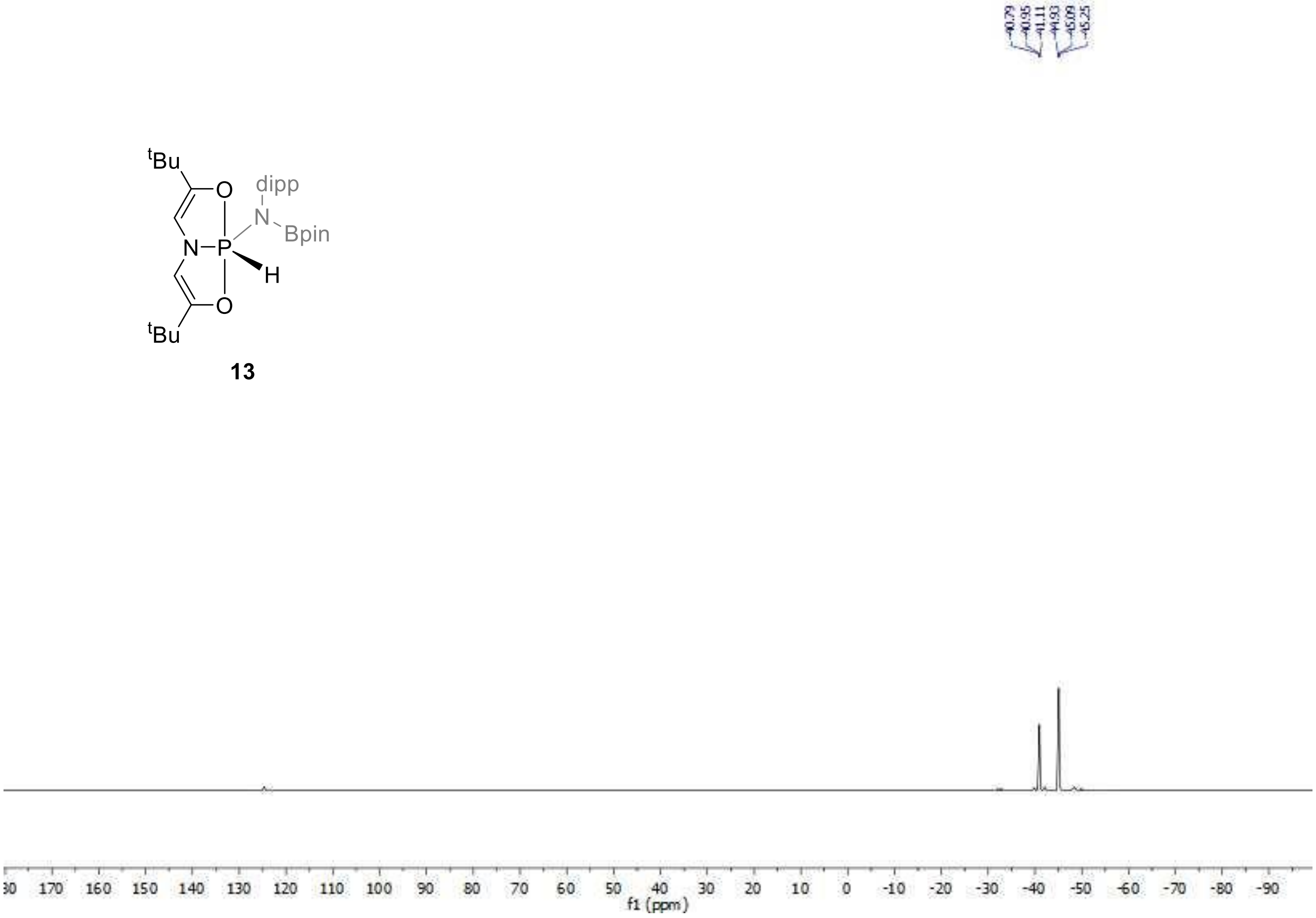

Figure S28. ^{31}P NMR of **13**.

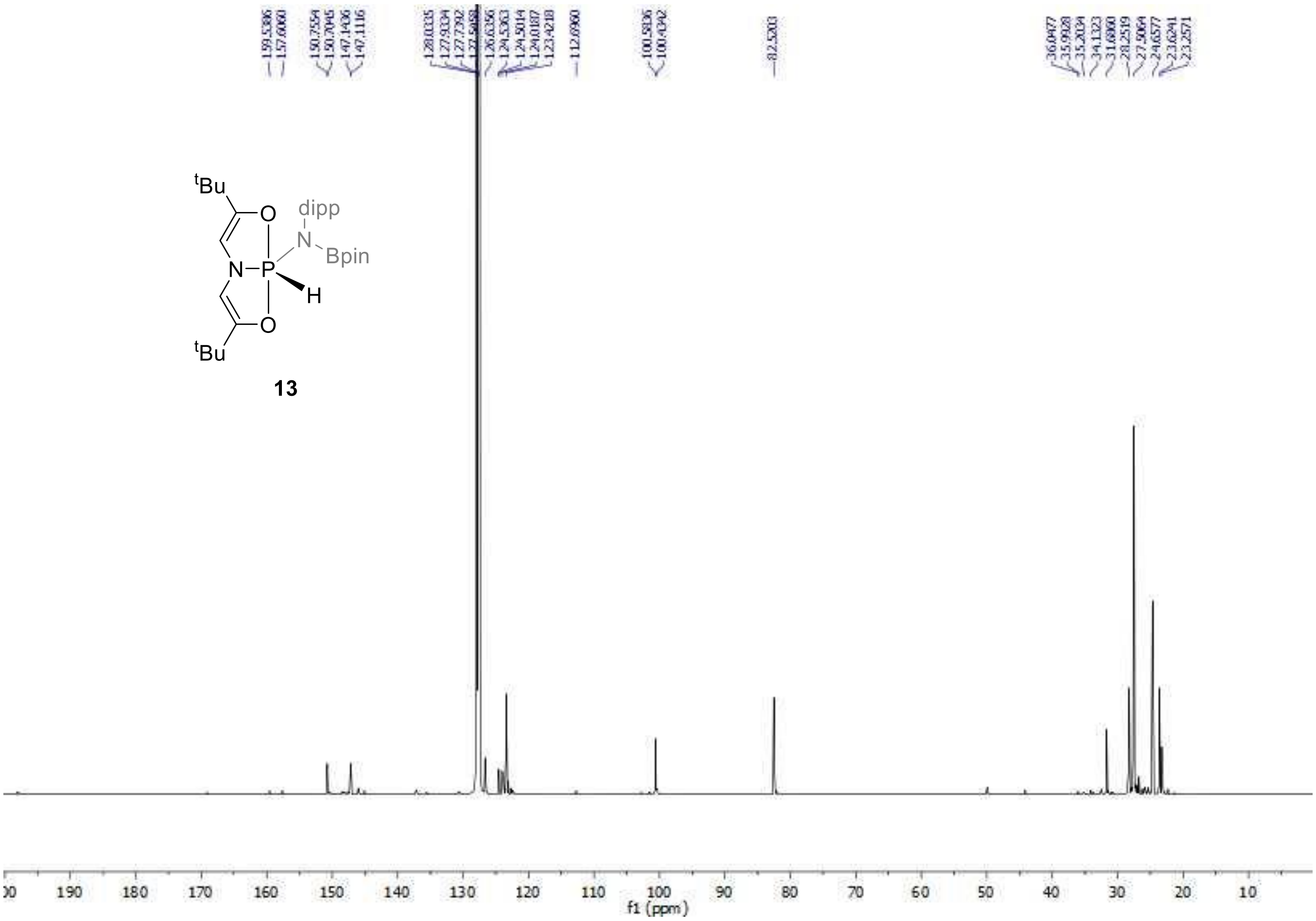

Figure S29. ^{13}C NMR of **13**.

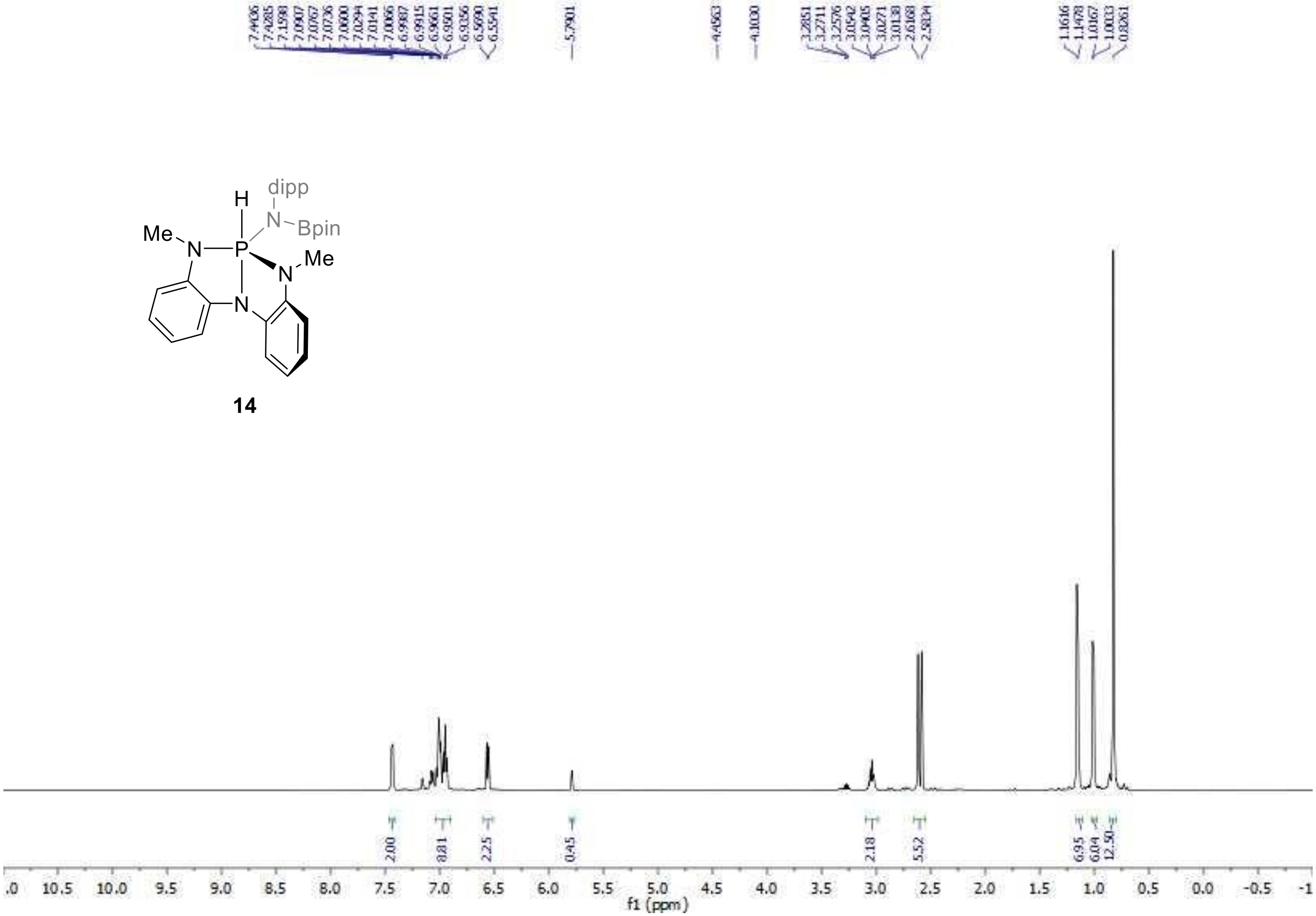

Figure S30. ^{1}H NMR of **14**.

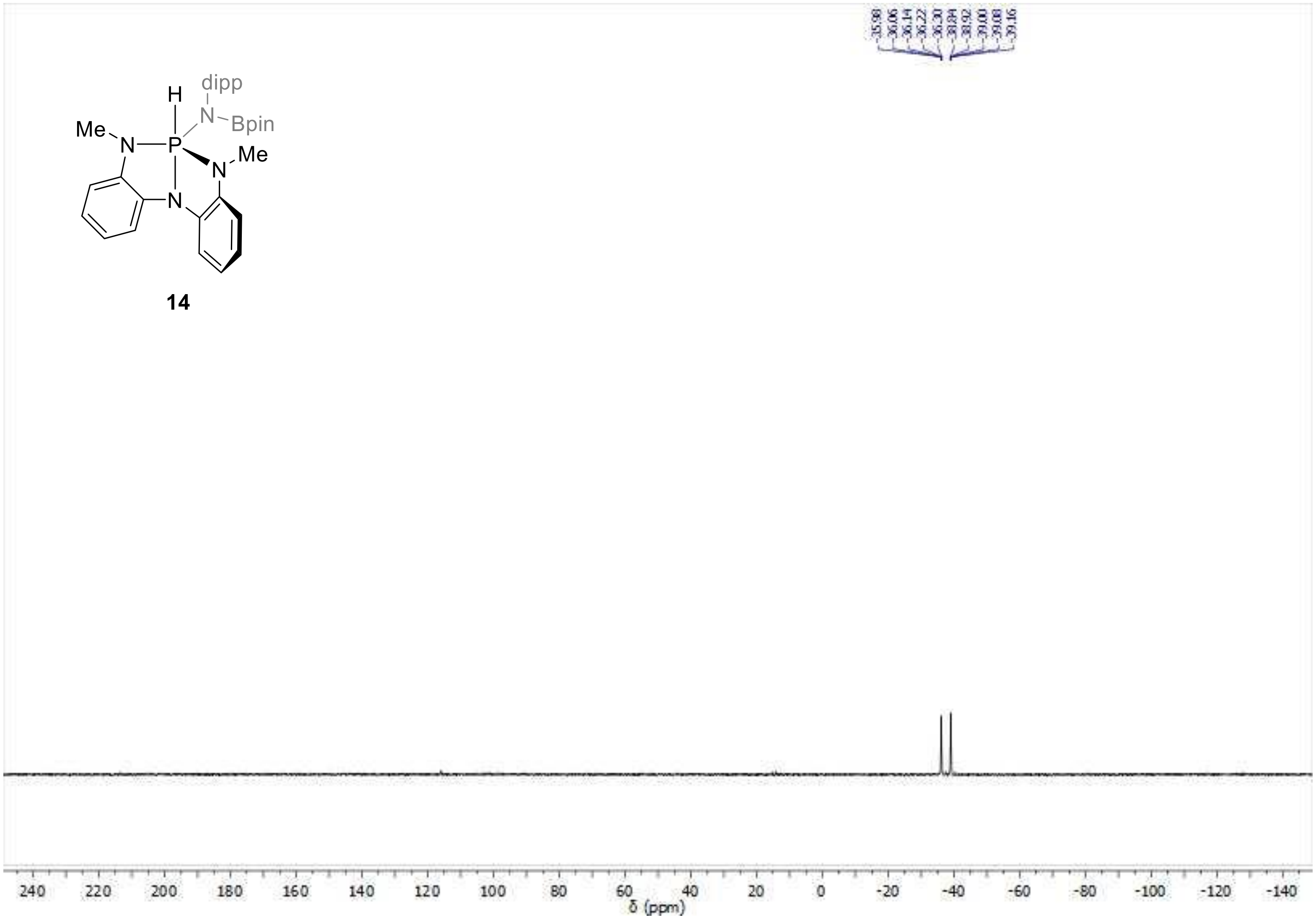

Figure S31. ^{31}P NMR of **14**.

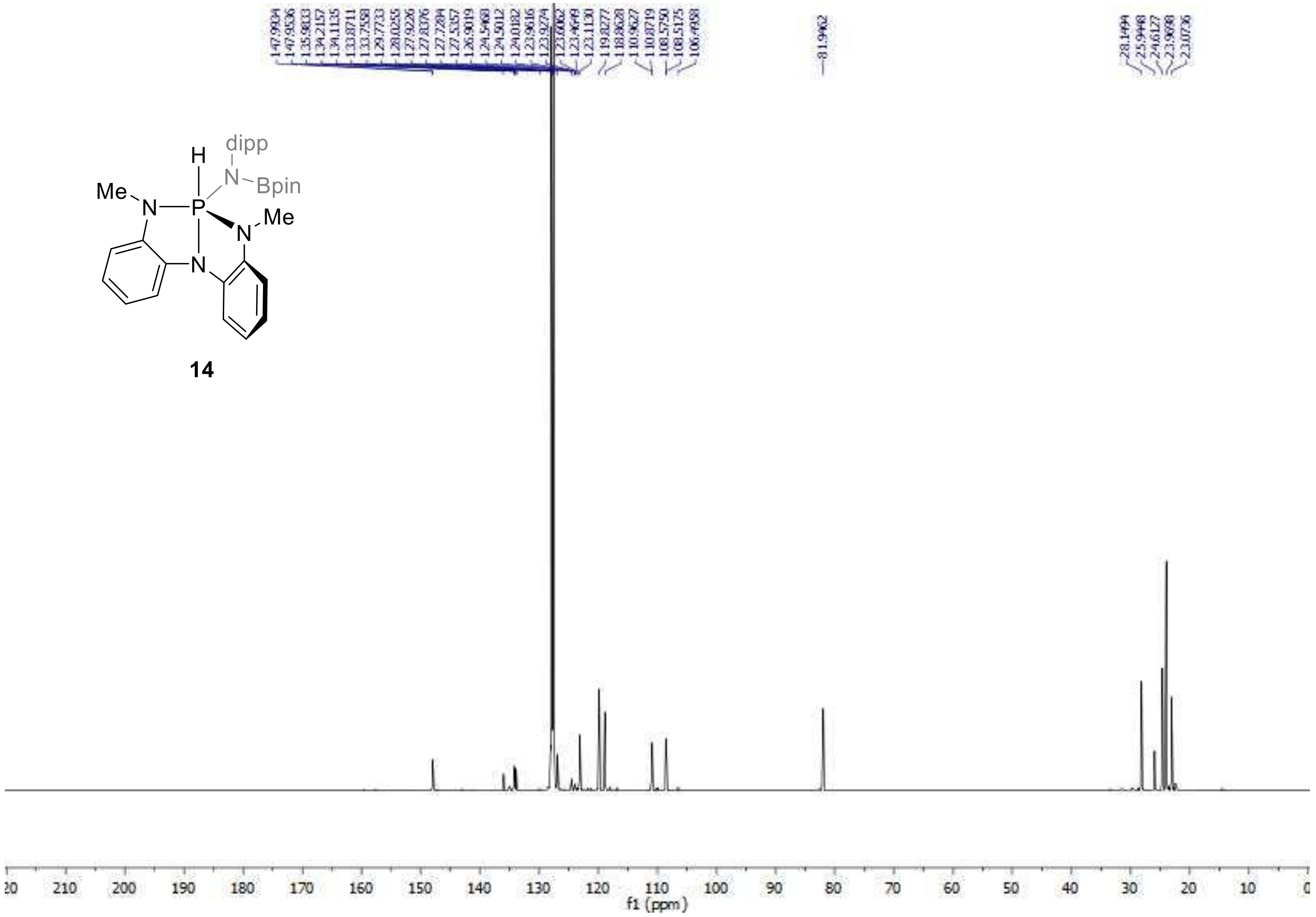

Figure S32. ^{13}C NMR of **14**.

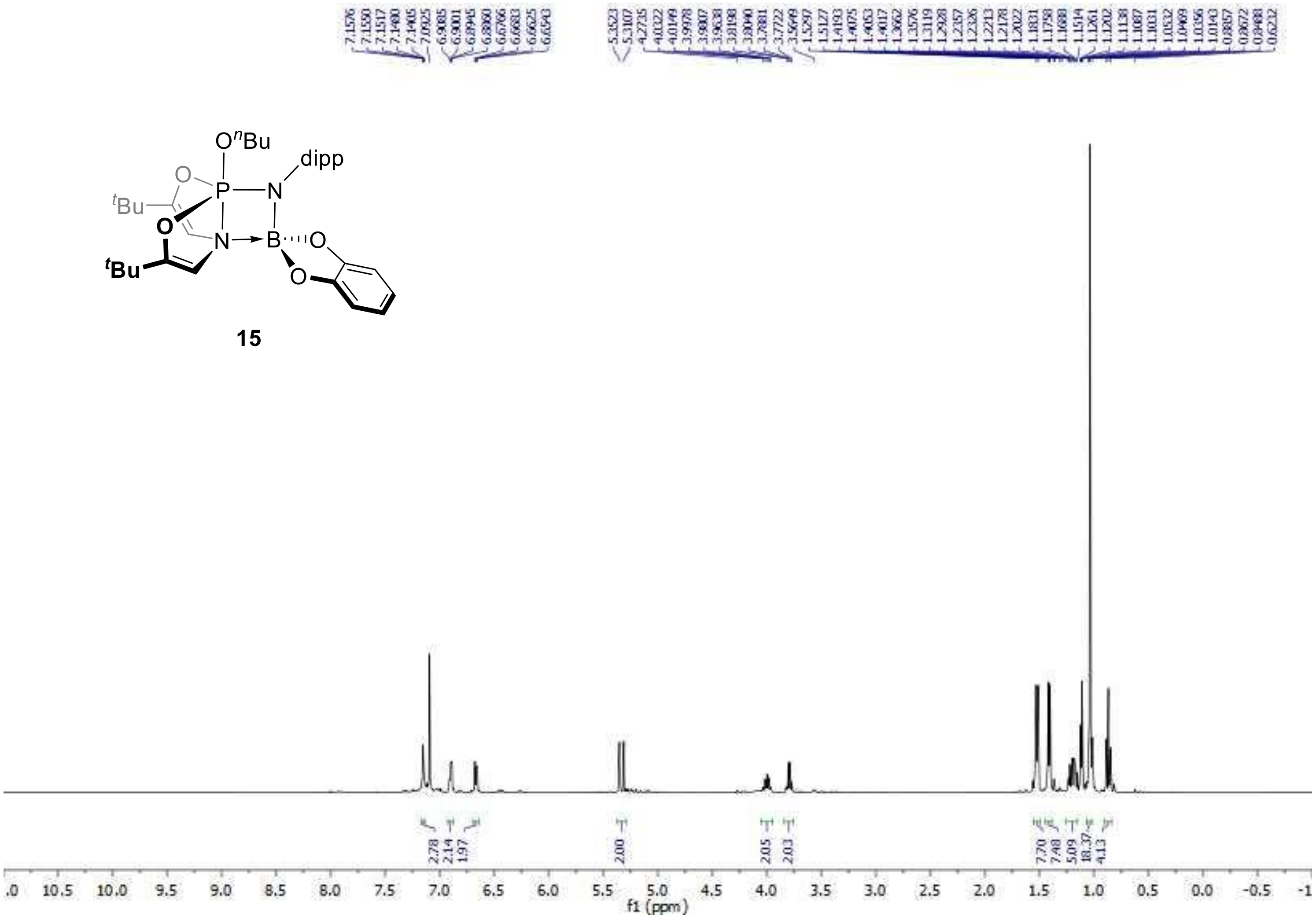

Figure S33. ^{1}H NMR of **15**.

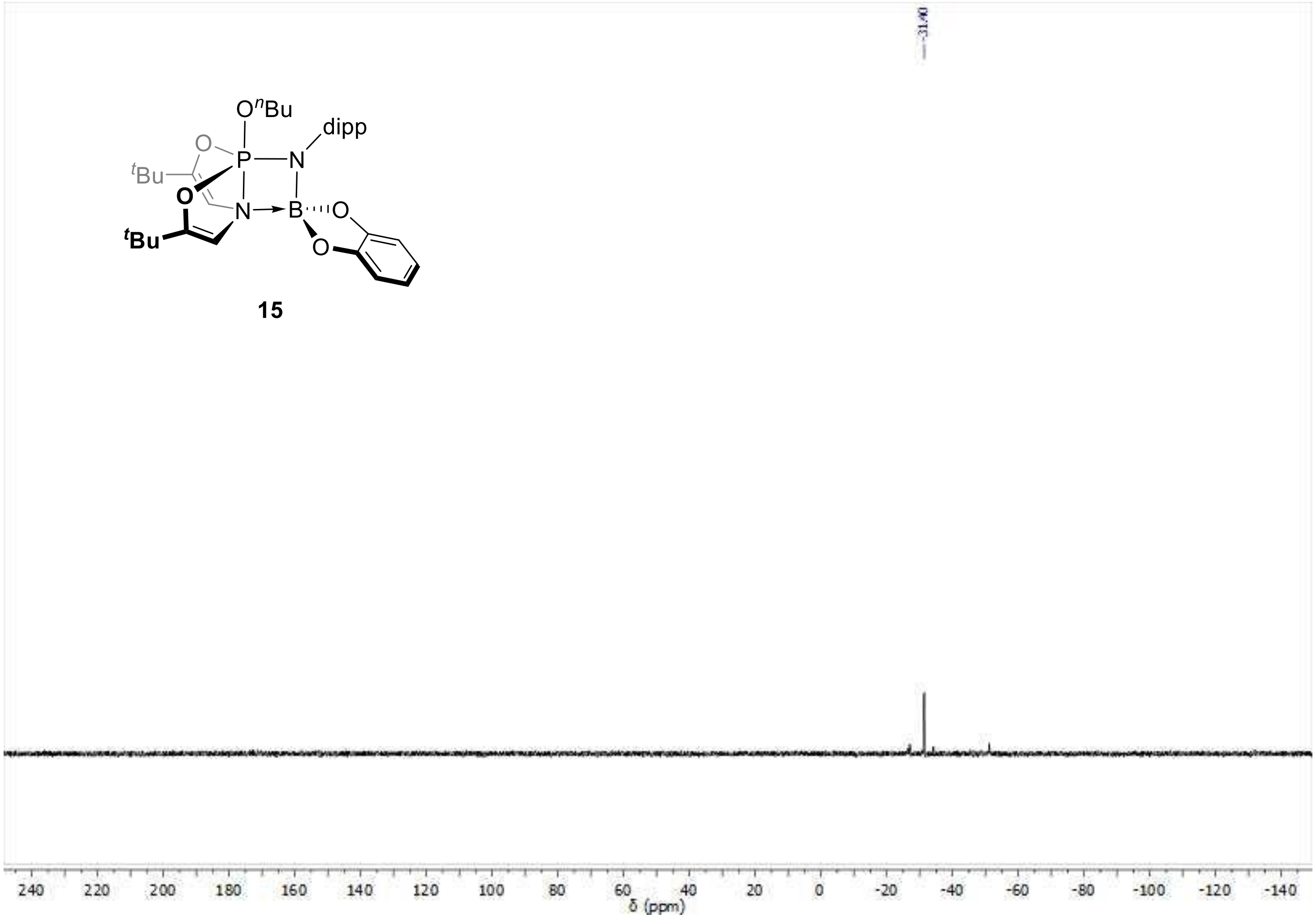

15

Figure S34. ^{31}P NMR of **15**.

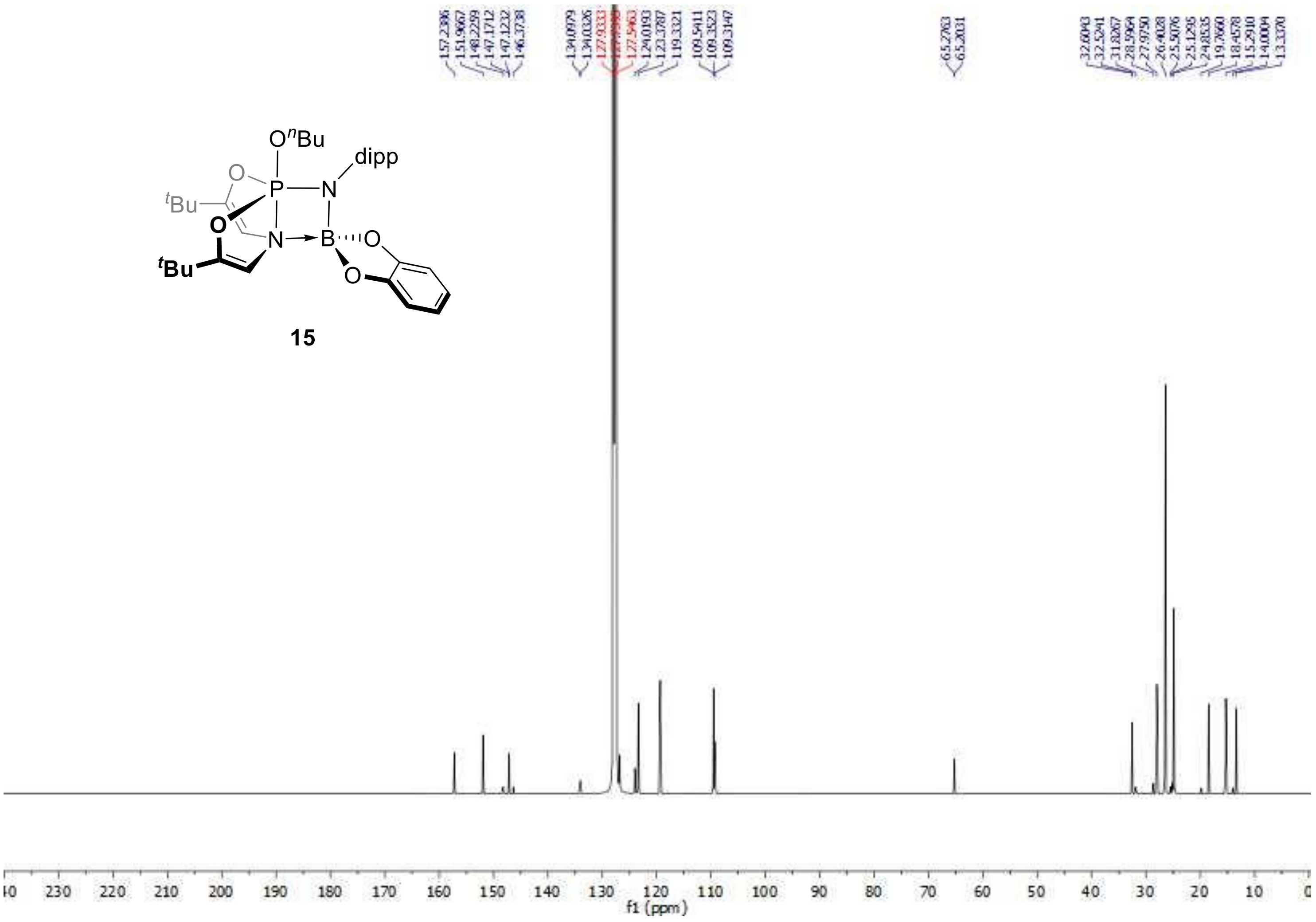

Figure S35. ^{13}C NMR of **15**.

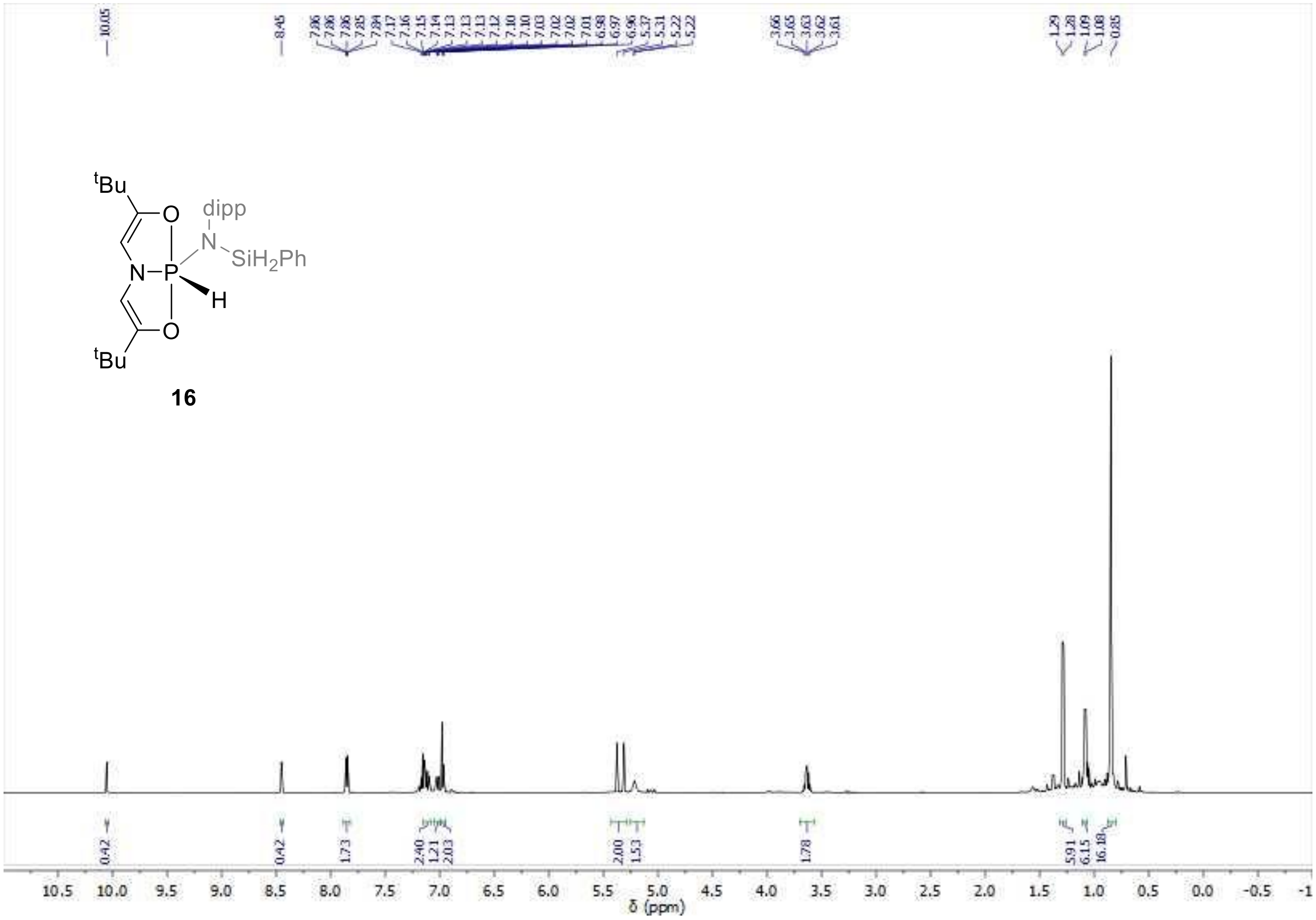

Figure S36. ^{1}H NMR of **16**.

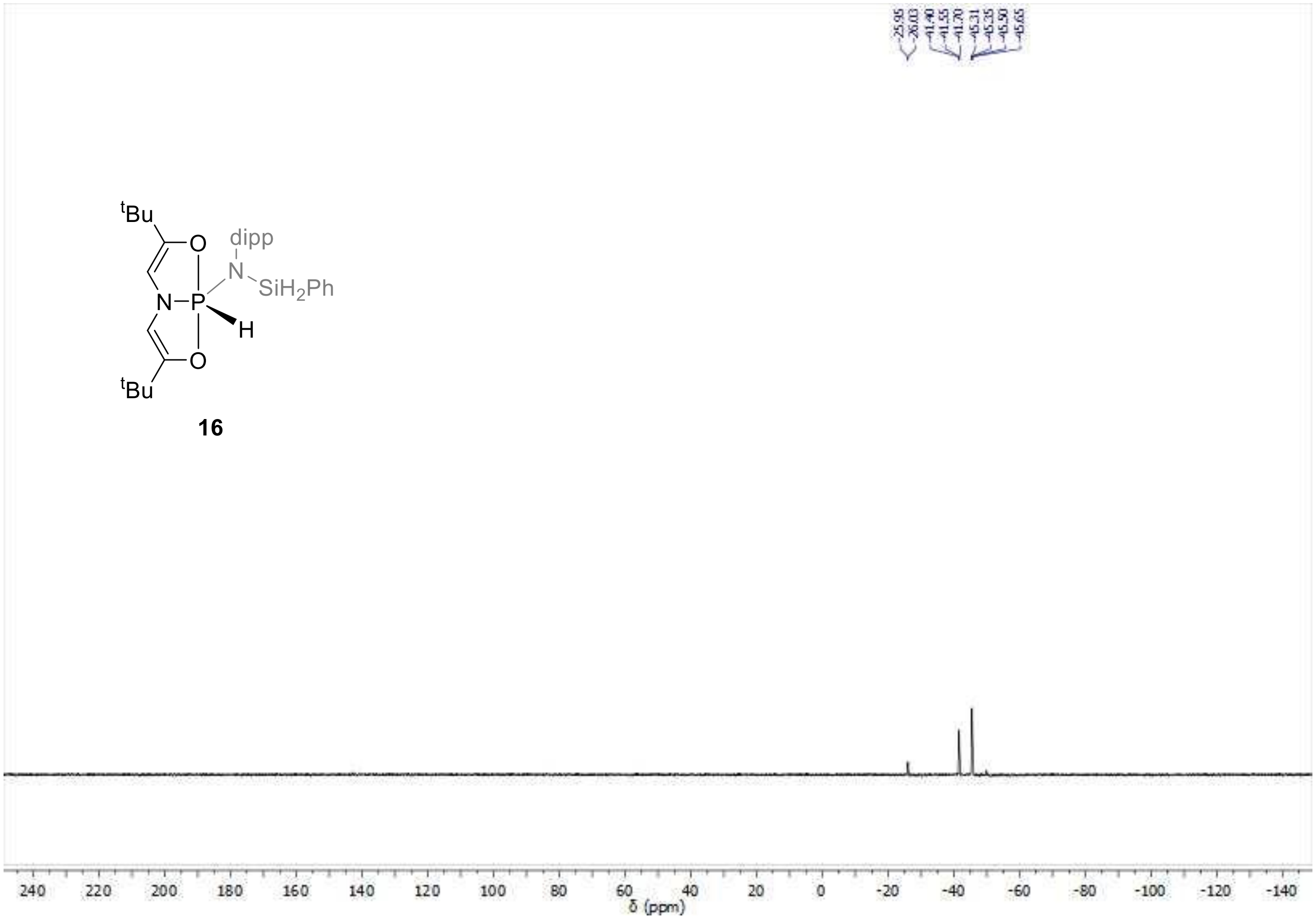

Figure S37. ^{31}P NMR of **16**.

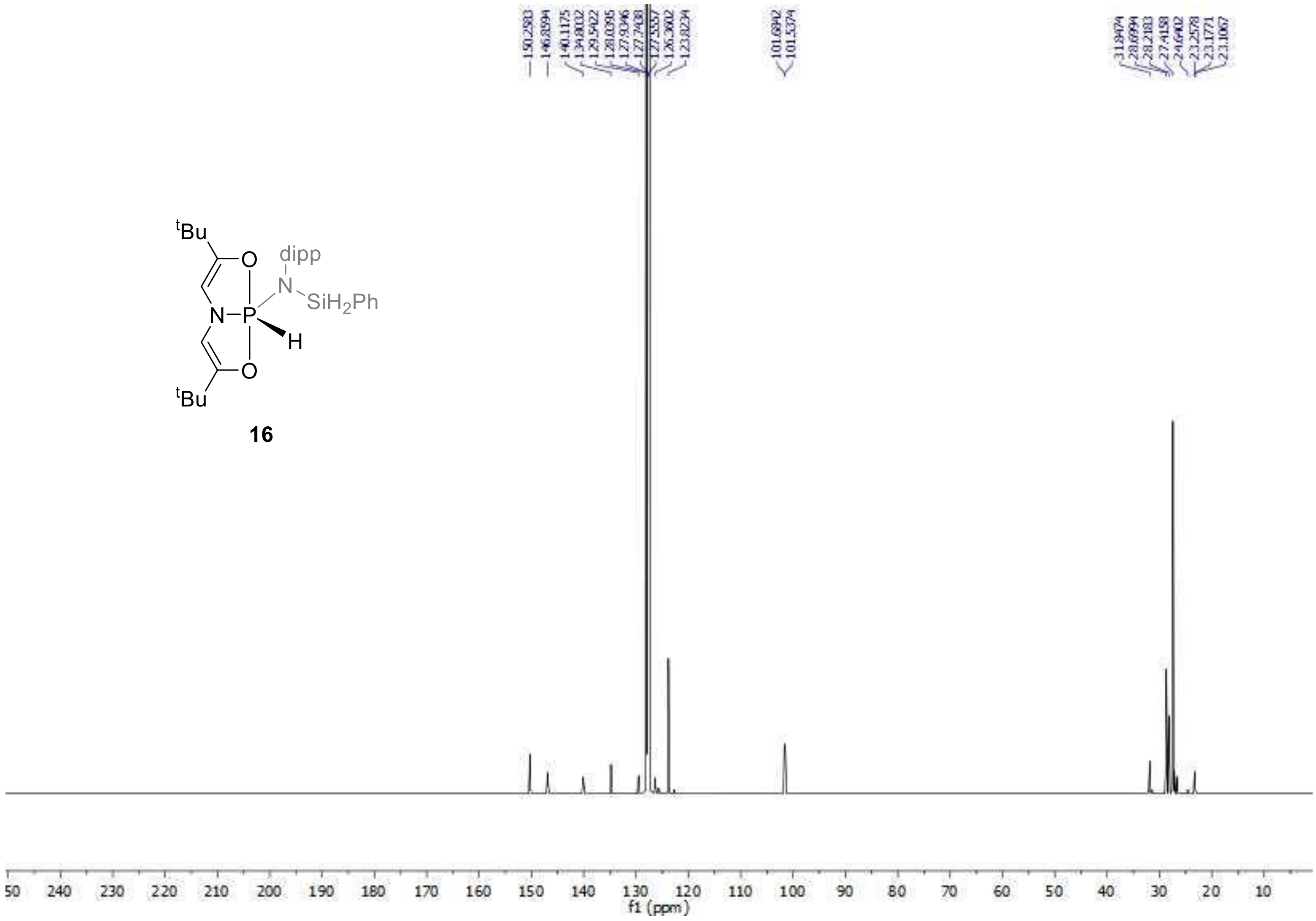

16

Figure S38. ^{13}C NMR of **16**.

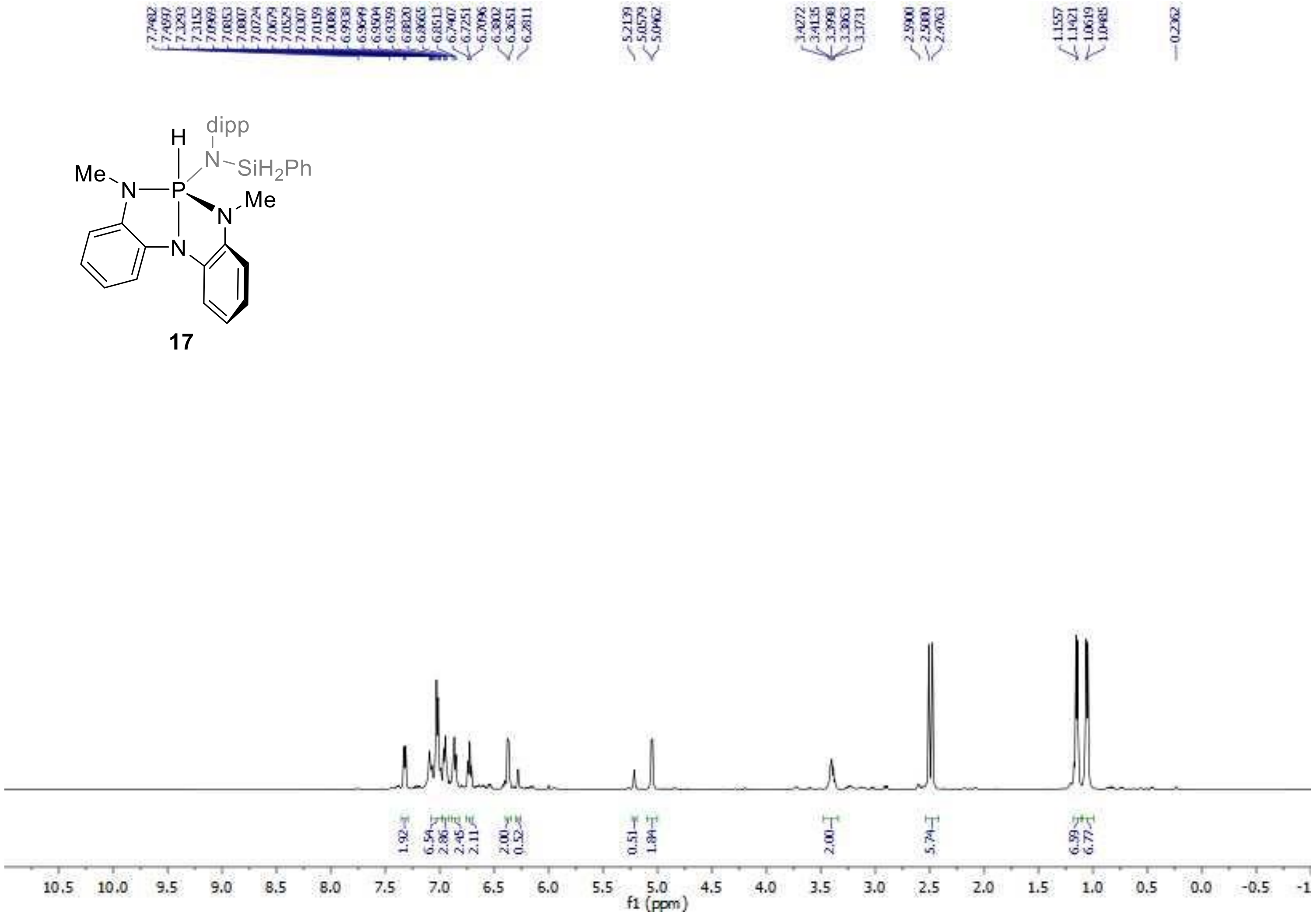

17

Figure S39. ^{1}H NMR of **17**.

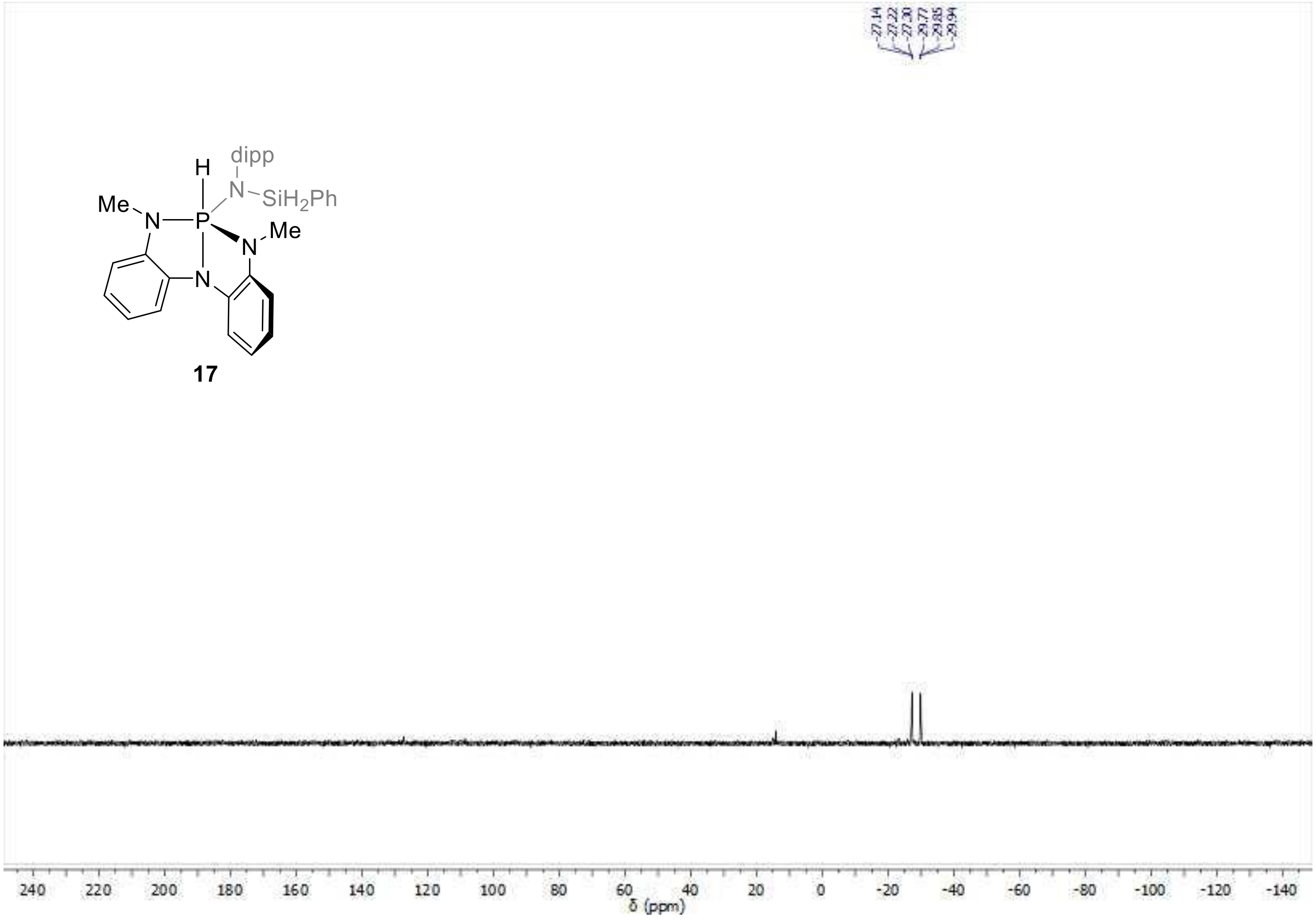

Figure S40. ^{31}P NMR of **17**.

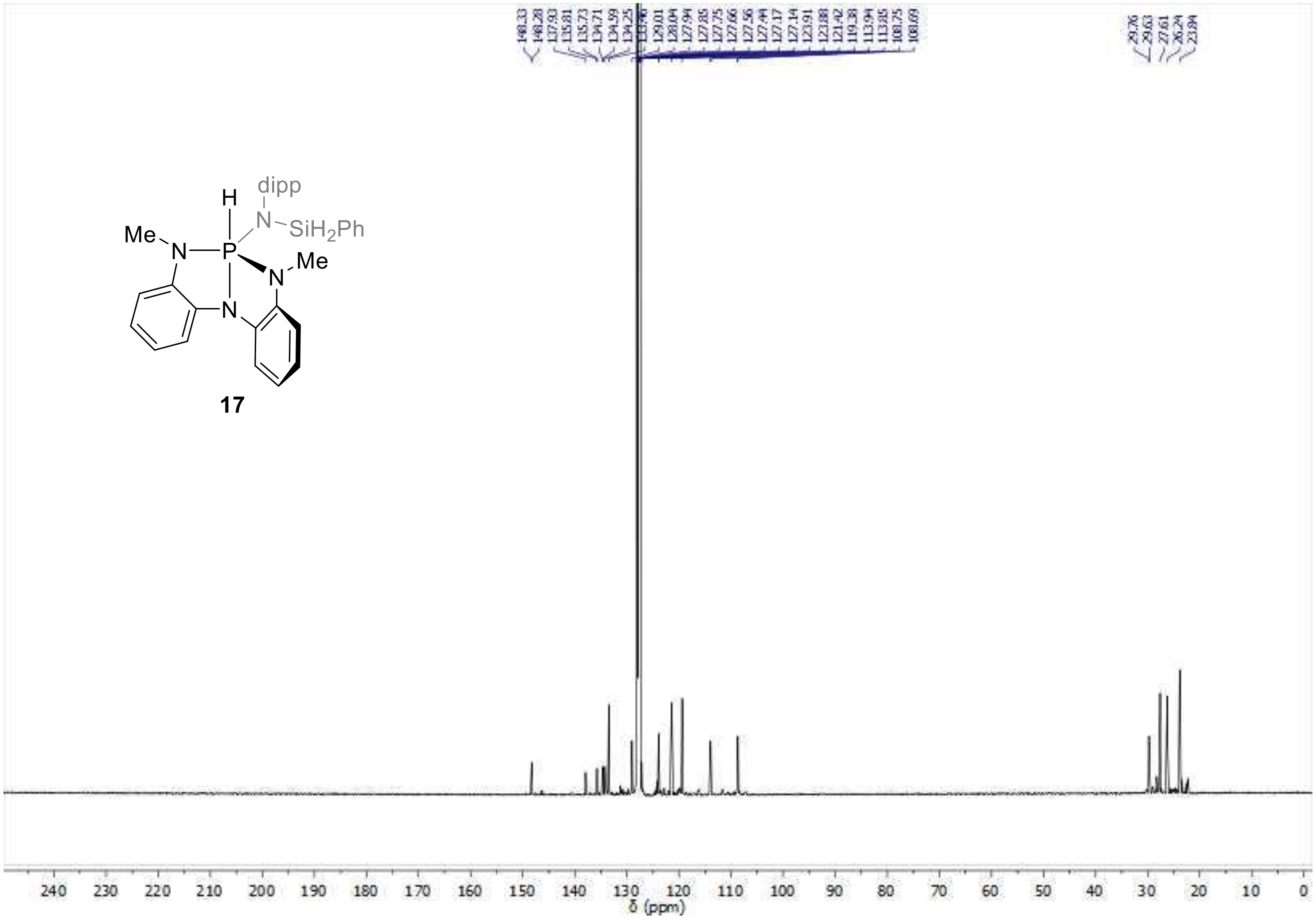

Figure S41. ^{13}C NMR of **17**.

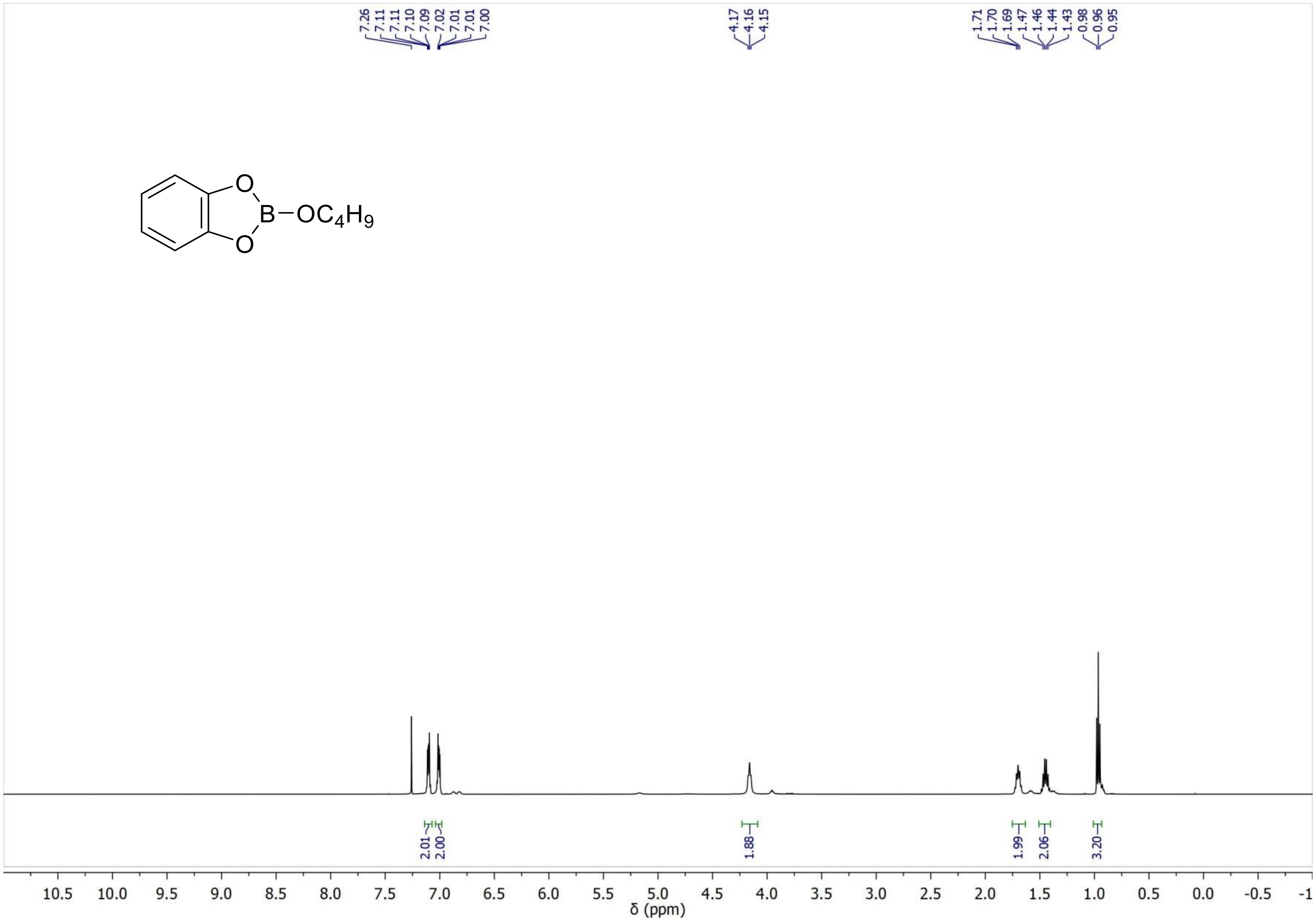

Figure S42. ^{1}H NMR of butoxy catecholborane.

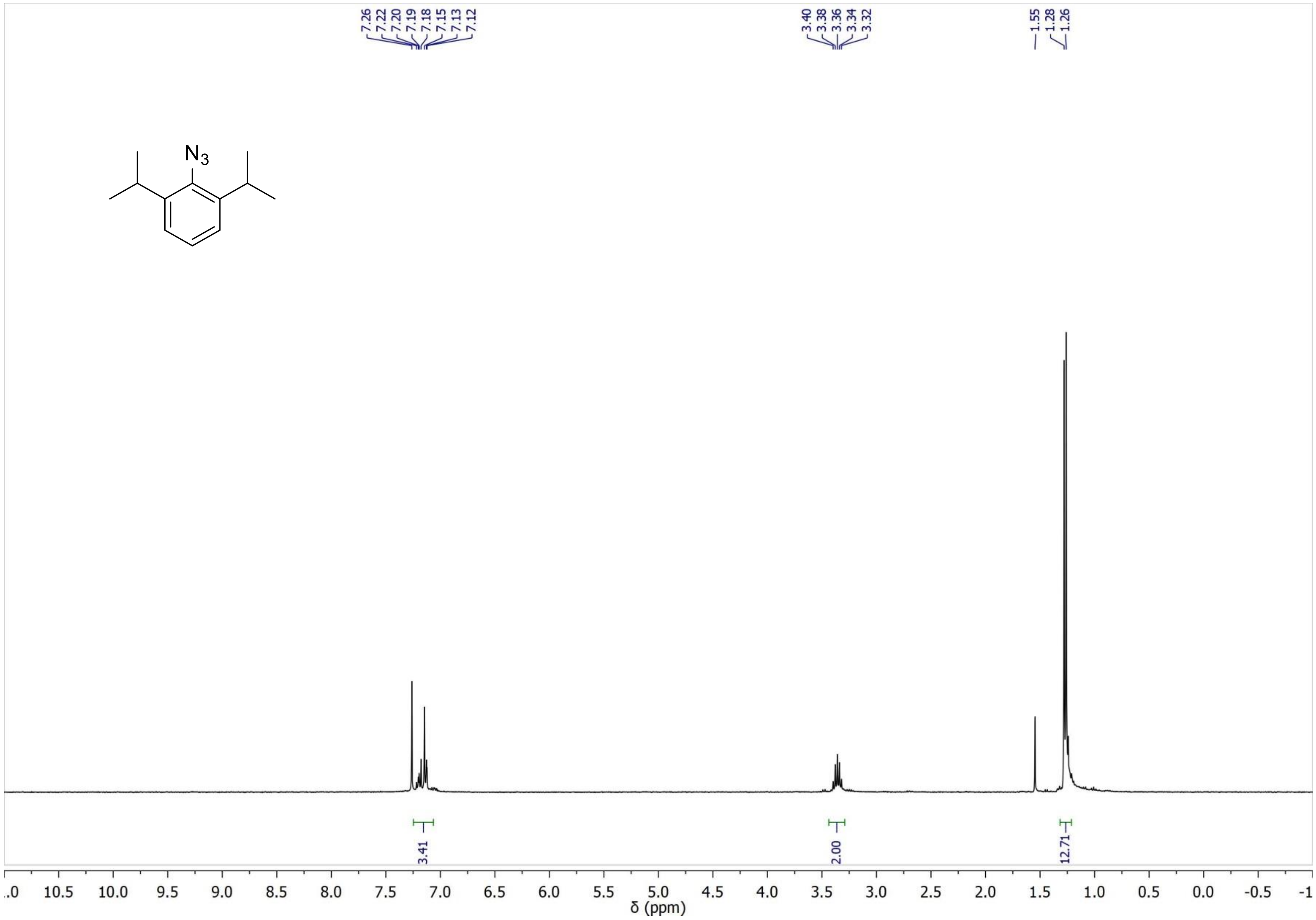

Figure S43. ^{1}H NMR of 2,6-diisopropylphenyl azide.

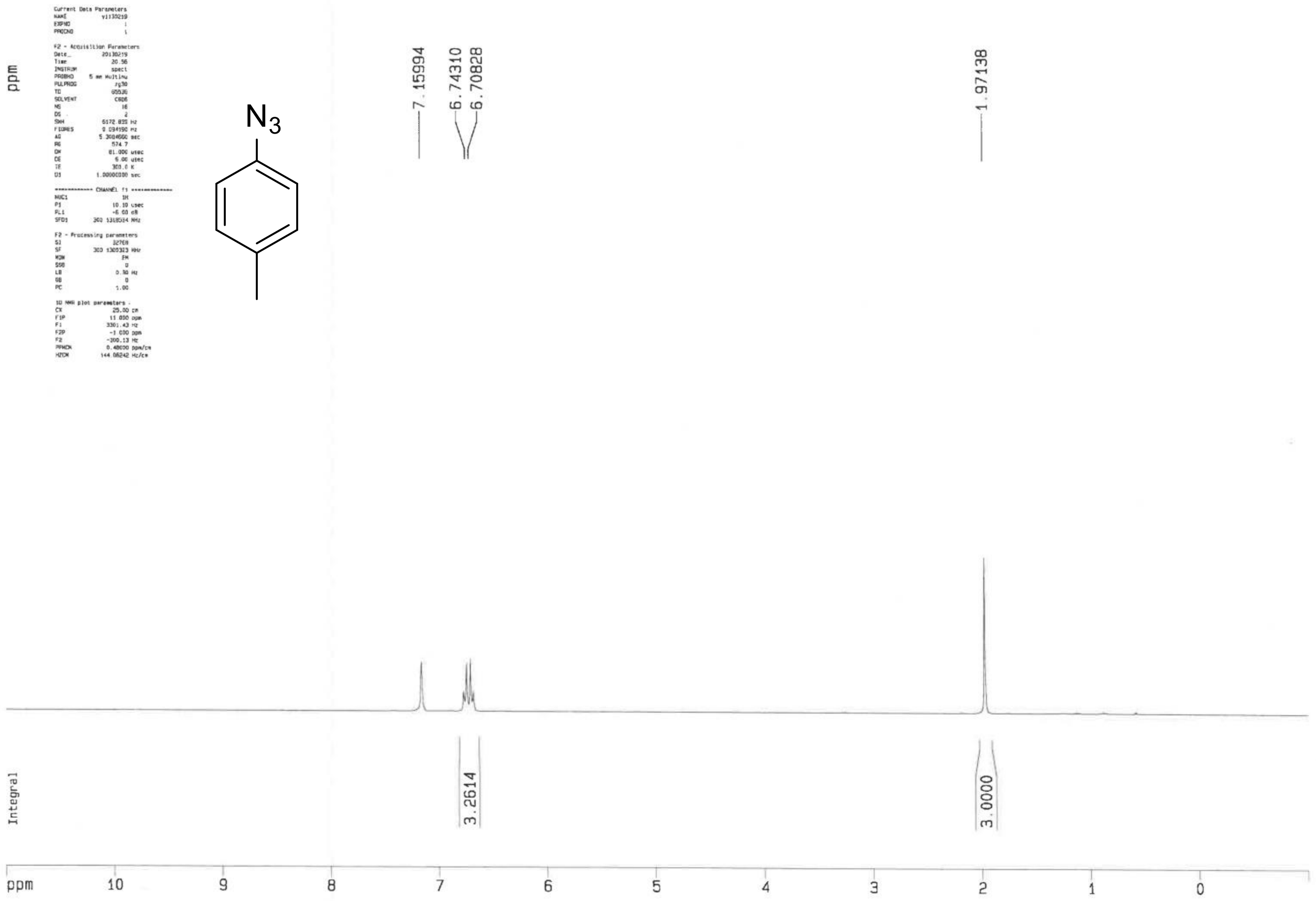

Figure S44. ^{1}H NMR of 4-methylphenyl azide.

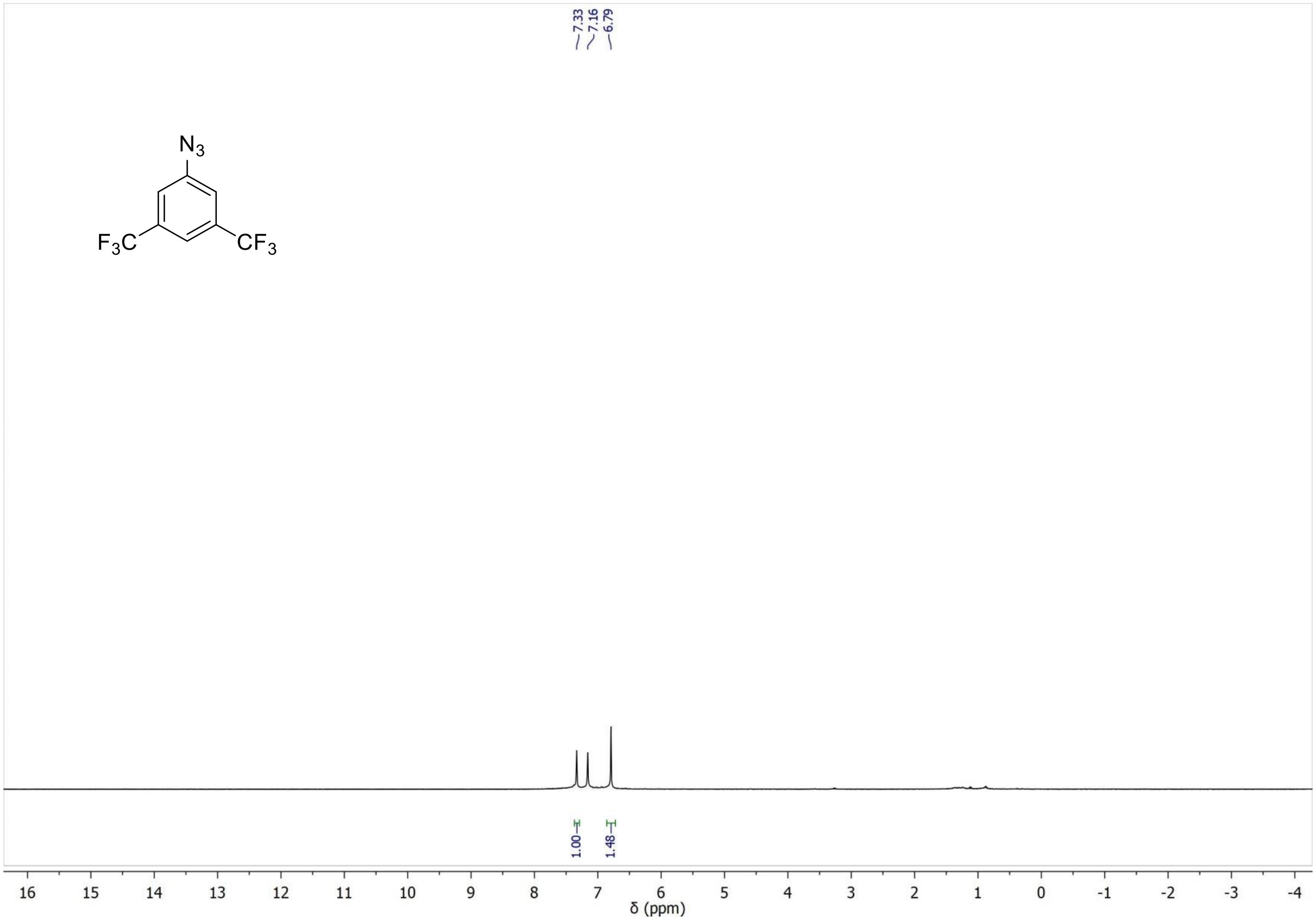

Figure S45. ^{1}H NMR of 3,5-bis(trifluoromethyl)phenyl azide.

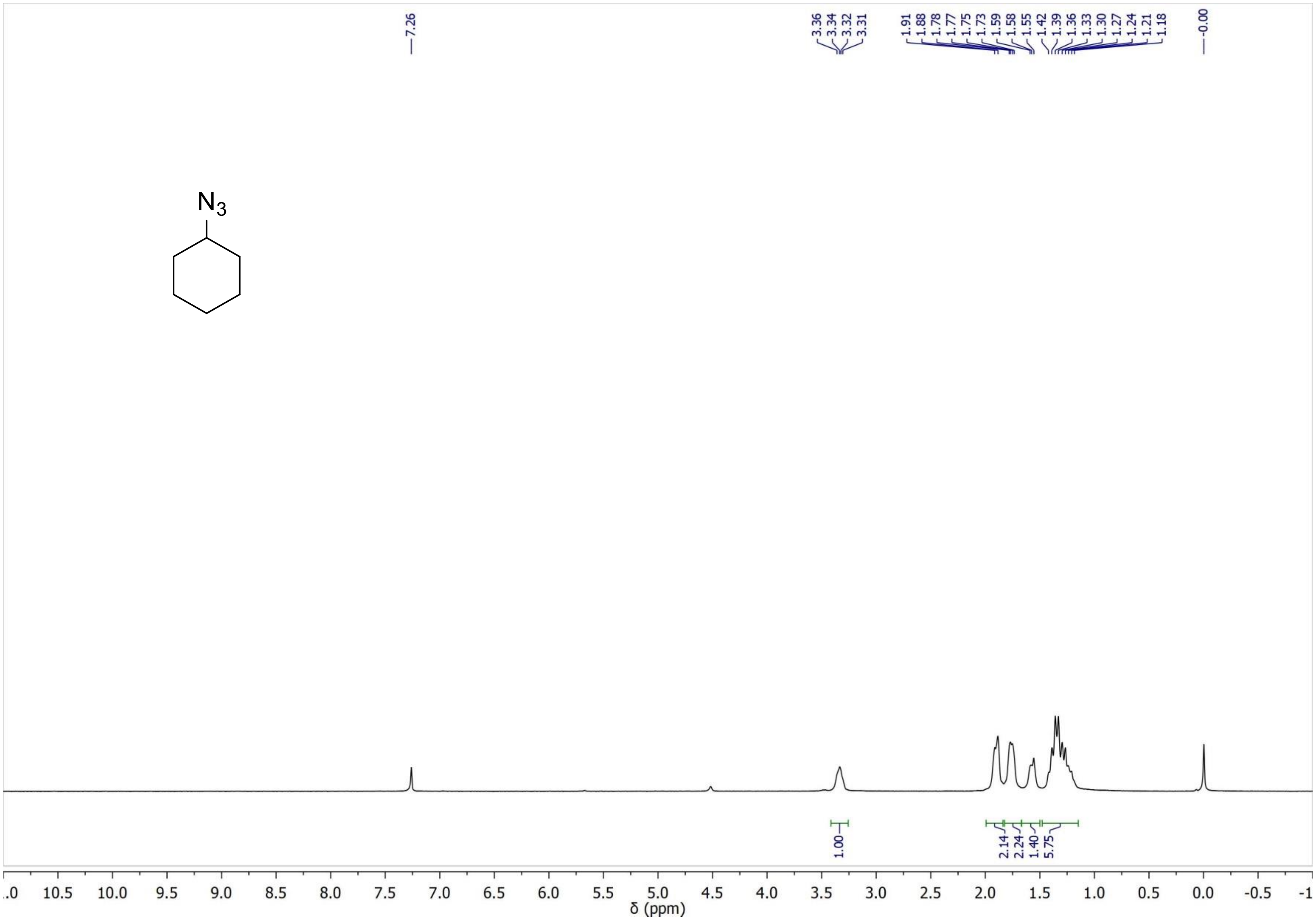

Figure S46. ^{1}H NMR of cyclohexylazide.

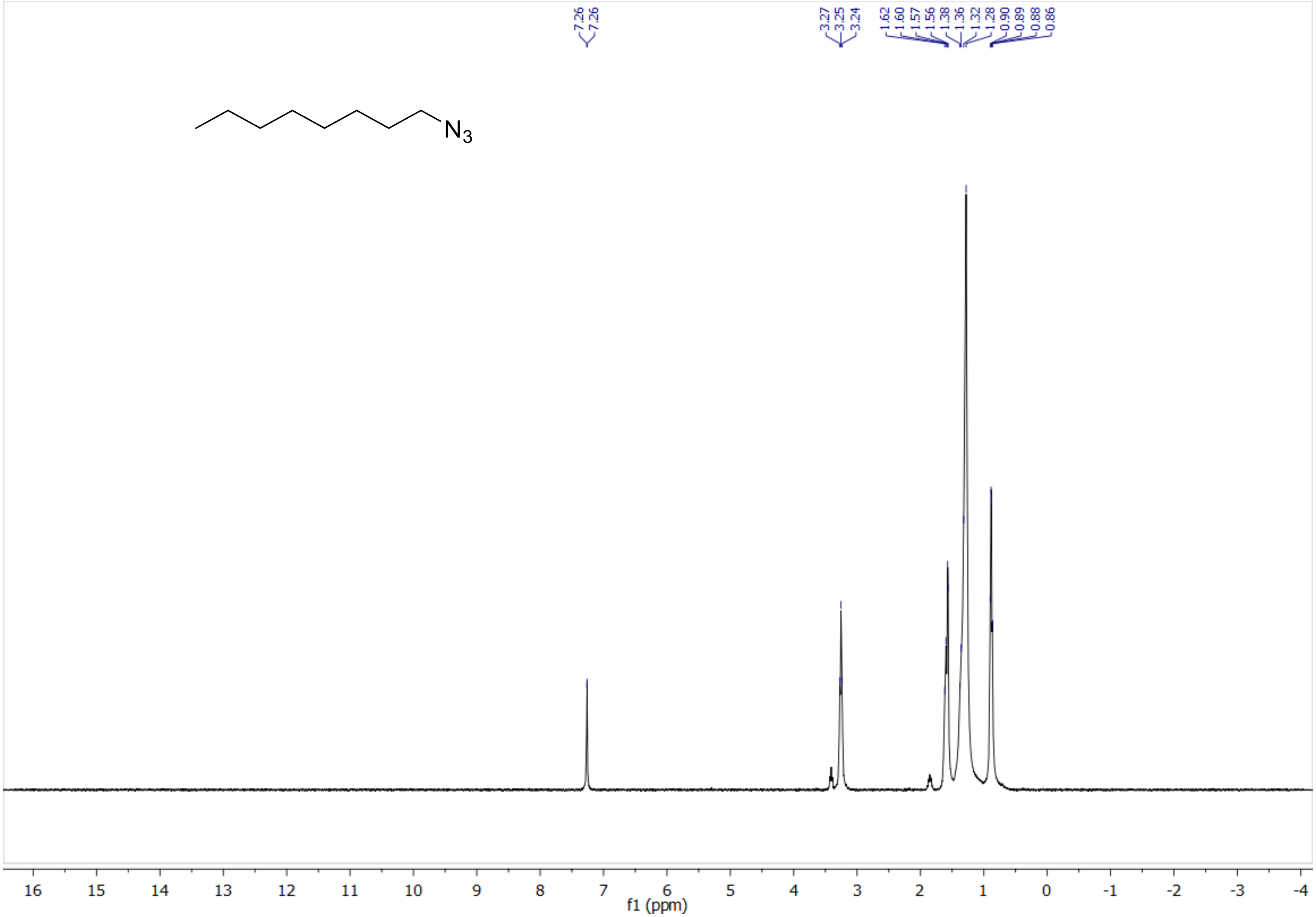

Figure S47. ^{1}H NMR of *n*-octyl azide.

VIII. DFT calculations.

H_3P=NH scans:

DFT calculations and geometry optimizations were carried out using the ORCA 4.0.0 software package at the M06-2X/def2-TZVP level of theory. Single point energies for the minimal model system H_3P=NH were scanned across the distortion coordinate α as in Figure 2. Reproduced here are single-point, HOMO, and LUMO energies for each of the geometries scanned, as well as the Cartesian coordinates for each scan.

H-P-H bond angle (deg)	HOMO (eV)	LUMO (eV)	HOMO-LUMO gap (eV)	Electronic energy (Hartrees)	Relative electronic energy (kcal/mol)
90	-7.87	1.12	9.00	-398.44	1.873
100	-7.82	1.12	8.94	-398.44	0.000
110	-7.76	0.95	8.71	-398.44	0.805
120	-7.69	0.57	8.26	-398.43	4.421
130	-7.67	0.06	7.73	-398.42	9.938
140	-7.67	-0.50	7.17	-398.41	17.181
150	-7.70	-1.03	6.67	-398.40	25.703
160	-7.82	-1.52	6.30	-398.38	34.901
170	-7.94	-1.93	6.01	-398.37	44.498
180	-8.08	-2.29	5.79	-398.35	54.853

α = 90°

P	-0.02850	-0.47220	-0.06890
H	1.09310	0.39370	0.07680
N	0.16300	-2.06450	-0.16410
H	-0.97000	0.01180	0.91850
H	-0.79730	0.15290	-1.10840
H	0.98874	-2.34635	0.36156

α = 100°

P	-0.02850	-0.47220	-0.06890
H	0.88760	0.33375	0.66608
N	0.16300	-2.06450	-0.16410
H	-1.30951	-0.06211	0.46622
H	-0.06376	0.19575	-1.33982
H	0.13550	-2.47330	0.76850

α = 110°

P	-0.02850	-0.47220	-0.06890
H	1.09310	0.39370	0.07680
N	0.16300	-2.06450	-0.16410
H	-0.82520	-0.10190	1.08170
H	-0.62800	0.06260	-1.25930
H	0.46001	-2.43246	0.73812

α = 120°

P	-0.02850	-0.47220	-0.06890
H	0.99056	0.35719	0.48123
N	0.16300	-2.06450	-0.16410
H	-1.10300	-0.19410	0.86045
H	-0.07243	0.06671	-1.39945
H	0.13550	-2.47330	0.76850

α = 130°

P	-0.02850	-0.47220	-0.06890
H	1.09310	0.39370	0.07680
N	0.16300	-2.06450	-0.16410
H	-0.64820	-0.23290	1.21730
H	-0.43150	-0.04900	-1.38080
H	0.29707	-2.45382	0.76761

α = 140°

P	-0.02850	-0.47220	-0.06890
H	1.05989	0.37750	0.28098
N	0.16300	-2.06450	-0.16410
H	-0.76996	-0.31963	1.16502
H	-0.09549	-0.07883	-1.44859
H	0.13550	-2.47330	0.76850

α = 150°

P	-0.02850	-0.47220	-0.06890
H	1.09310	0.39370	0.07680
N	0.16300	-2.06450	-0.16410
H	-0.45640	-0.36810	1.31010
H	-0.22730	-0.17090	-1.45900
H	0.29707	-2.45382	0.76761

α = 160°

P	-0.02850	-0.47220	-0.06890
H	1.09350	0.39407	0.07142
N	0.16300	-2.06450	-0.16410
H	-0.34912	-0.43654	1.34230
H	-0.12876	-0.23627	-1.48210
H	0.13550	-2.47330	0.76850

α = 170°

P	-0.02850	-0.47220	-0.06890
H	1.09350	0.39407	0.07142
N	0.16300	-2.06450	-0.16410
H	-0.24532	-0.50580	1.36198
H	-0.02330	-0.30265	-1.49513
H	0.13550	-2.47330	0.76850

α = 180°

P	-0.02846	-0.47222	-0.06887
H	1.09305	0.39374	0.07680
N	0.16295	-2.06453	-0.16406
H	-0.13206	-0.57458	1.37311
H	0.08785	-0.37648	-1.49782
H	0.13548	-2.47327	0.76850

Geometry Optimizations and Frequency Calculations of **3**, **4**, **7**, and **8**

Geometries were optimized using the ORCA 4.0.0 software package at the M06-2X/def2-TZVP level of theory using compounds **3**, **4**, **7**, and **8** in their full, untruncated forms. Frequency calculations were performed on equilibrium geometries, and the absence of imaginary modes confirmed that each compound was at a true minimum on the potential energy surface. Zero-point energy and thermal corrections were applied to the electronic energy to obtain a corrected thermal energy. Reproduced here are Cartesian coordinates, electronic energies, and zero-point-energy- and thermally-corrected enthalpies.

**Compound**	**Electronic energy (Hartrees)**	**Corrected thermal energy (Hartrees)**
**3**	-1538.09896	-1537.49050
**4**	-1569.76671	-1569.21138
**7**	-1611.94184	-1611.36463
**8**	-1499.55072	-1498.88666

Compound **3**:

C	3.11444105599789	6.40289367640790	7.42973776778926
C	2.30402812790470	7.22574103986335	8.23643109410630
C	0.66726955128317	8.22021926272465	6.62073973342478
H	1.00534715683695	9.24277932344891	6.80093318973045
H	-0.38289153107181	8.25748051104335	6.32341319285811
H	1.24666539516489	7.80375477741420	5.79912321587283
C	0.83505741412014	7.38569260859596	7.89663134849553
H	0.44125204143303	6.38488028602812	7.69155089398228
C	-0.00099433933213	7.98396292527735	9.02370498259911
H	0.26213079610982	9.02861571998328	9.20118965518819
H	0.12682820167157	7.43751149766982	9.95855007506465
H	-1.05706435774955	7.95575314323102	8.75367959331825
C	2.89345030204570	7.88175828157004	9.31043681587357
H	2.28869202868553	8.51265179328310	9.94837038917951
C	4.24552049147842	7.74198523874066	9.58446646662467
H	4.68460965931063	8.26224629971400	10.42564426099288
C	5.03207423930242	6.93715929143859	8.77643335509162
H	6.08741685966170	6.83525365975996	8.99428660009095
C	4.48694096415823	6.26130089311414	7.69150927822571

C	5.30457863406228	5.33381243141064	6.81914882030325
H	4.89325822680420	5.40211062977137	5.80974433683854
C	5.11994437825522	3.88962374858453	7.30010140237557
H	5.55076351250596	3.77091458342094	8.29700101582991
H	5.60595287844672	3.18254922031089	6.62465302025534
H	4.06244692637302	3.63115792573635	7.36618404867447
C	6.78583088415296	5.68965313605011	6.75187725555248
H	6.93144770811814	6.73183543477031	6.46599864130382
H	7.28661692676028	5.05883155154460	6.01659479224136
H	7.28138202788562	5.52841532506404	7.71084641750210
C	1.65230402742262	3.72004391169930	3.39549489824057
H	1.92484388561468	3.21073134196843	2.48624631156942
C	0.66029318038124	4.59570199618430	3.55105400605406
C	-0.32154423268141	5.20995196554311	2.60026991997682
C	-1.72469716285786	5.14462776826679	3.22057185519112
H	-2.02655045328107	4.11241529702198	3.40305882910947
H	-2.44294348753351	5.59893947835712	2.53655516421177
H	-1.75821164368952	5.68686537371556	4.16423457928213
C	0.06006850573629	6.68334425710498	2.38593986289717

H	0.06380244520567	7.22129424628527	3.33372512804785
H	-0.66454640194075	7.15815221589280	1.72255781872352
H	1.04923983434151	6.76714257423303	1.93513648310114
C	-0.29966663899302	4.45868362767579	1.27263445274194
H	0.68718217846211	4.49925987188661	0.80951421336691
H	-1.01135708251750	4.91518553571341	0.58404229621090
H	-0.57936091285976	3.41334872195539	1.41095254814157
C	2.37990805830058	2.22701335913069	5.21320224098505
H	2.83823991628188	1.38094736471857	4.72880405781460
C	1.78257205508121	2.23075899904978	6.40406403565472
C	1.59219443791894	1.16895191353545	7.44315792242819
C	0.10199721180173	1.09541270804428	7.80439544272103
H	-0.24692017237315	2.04572770741502	8.20658789701288
H	-0.05214936735304	0.32470462212033	8.56065136056247
H	-0.50100776960710	0.84803810889969	6.92982207795049
C	2.07058977225945	-0.17553548417010	6.90184695145229
H	1.51122630983384	-0.46205990332378	6.00996775434906
H	1.92230441265551	-0.94631108742863	7.65833556455128
H	3.13295306314458	-0.14562718312890	6.65635121969758

C	2.39718048579742	1.54652956329435	8.69618101596613
H	3.46626762229917	1.56490798327079	8.48249887215010
H	2.21380921616937	0.81321303065819	9.48290076226555
H	2.10300808366032	2.52923026434947	9.06505796316183
N	2.41955546185748	3.51684118577520	4.58926117243806
N	2.56102733254700	5.75743451569102	6.33512893143319
O	1.29997455855485	3.48288583439596	6.77997047167098
O	0.57570934251716	5.10603873742603	4.84120135511670
P	1.81010776746699	4.61476536080100	5.74504290436538

Compound **4**:

C	7.89484650954377	7.95914841097848	2.12001406663669
C	7.06070364044706	8.38586315667775	3.17934501435792
C	5.85926707366917	7.72816555332843	3.40221267086278
H	5.21658880611107	8.04581795364551	4.21326338413289
C	5.47143382682981	6.65627474210251	2.60907265977221
H	4.54207298478799	6.14241018379747	2.81749353061415
C	6.27455396511341	6.26371914347455	1.55312974359123
H	5.96282005448145	5.44072004091082	0.91968464662814

C	7.48455952328393	6.90333693313068	1.28132418857810
C	8.31053833462297	6.44984477361507	0.09043896342336
H	9.24100375436912	7.00847769450650	0.09883950441617
C	8.68402621878907	4.96540419326022	0.14611222220434
H	9.09670678067579	4.69504830975212	1.11788301548941
H	9.42955464174573	4.73996987160973	-0.61784909391443
H	7.81331623355754	4.33642174958755	-0.05091645164838
C	7.60970530749164	6.74168478256633	-1.24266539475431
H	6.66975958194854	6.19066865752507	-1.31226160064479
H	8.24473313660003	6.42636070625169	-2.07391524581146
H	7.37842546725584	7.80259747536488	-1.35635806157756
C	7.52613382019655	9.51045121571274	4.08395154061563
H	8.05353198076101	10.23131899379701	3.45533906252088
C	6.38924777282988	10.24252810257554	4.79511112104640
H	5.63585126379019	10.59772942620265	4.09157967887916
H	6.78500967029940	11.10067324254754	5.33948248352377
H	5.89374886867331	9.60082094090331	5.52721975165440
C	8.52890870453596	8.96711086591361	5.10769387912732
H	8.02438117873321	8.26265857471335	5.77217399882185

H	8.94748850350395	9.77185839437227	5.71714137319481
H	9.34787641123889	8.44214615744967	4.61578347068206
C	10.30580120716170	9.17312502966806	-0.91923573107086
H	10.89097964164212	9.88263816498056	-1.50645625585722
H	9.44518781888564	9.68622096089354	-0.49051860499918
H	9.93612871834977	8.38566044194298	-1.58021743718206
C	12.00515282062748	7.57128759994637	-0.08341460776828
C	12.49020883774390	7.15810956036915	-1.31020869908022
H	12.21253124418478	7.68091845289254	-2.21582774343502
C	13.34780560209366	6.05868448318964	-1.35475672457588
H	13.72601325317810	5.72168455150028	-2.31109764912247
C	13.71626602928790	5.39794041840602	-0.19571309510403
H	14.38233168770008	4.54933182936470	-0.24776950591196
C	13.22495271135849	5.81367170068402	1.04220584866790
H	13.49770573583541	5.29318478760516	1.94804044957422
C	12.37865981185200	6.89820478216970	1.09432010102829
C	12.51717278119810	7.96431419167362	3.34597163685431
C	13.37867150997405	7.26986027871229	4.16836681499202
H	13.53692687007861	6.20965425772759	4.03261730977189

C	14.04204982289487	7.95990147123034	5.18627091895908
H	14.72491038544406	7.42642562661461	5.83156538540622
C	13.82003123971146	9.31036374590653	5.37854857320898
H	14.34238683118290	9.83691598324254	6.16536873696540
C	12.93457067299332	10.01680444593349	4.56372667221320
H	12.76595040047368	11.07283944568524	4.72705934983259
C	12.27886193402512	9.33529105794180	3.55079266631509
C	10.76352335398040	11.17151585950118	2.84813266034237
H	10.46784080007199	11.32759268767453	3.88936064749930
H	9.87545405411633	11.26805965759904	2.22660078521260
H	11.47962972178872	11.94382182235726	2.56068184624809
N	9.10913388964637	8.61570671749097	1.96070195782440
N	11.09775137439865	8.60693381725764	0.15449188675819
N	11.76060174068664	7.46358096033612	2.24502530393363
N	11.33072524938594	9.85111475657116	2.66204303062211
P	10.60371723216145	8.62926720665818	1.76368234945453

Compound **7**:

C	3.15942069332129	5.03246645494906	10.44739790395816

C	4.07826936148324	5.97006178574488	10.96167959803012
C	5.30668977736280	6.12073351217018	10.32756228994426
H	6.02378569897380	6.82875996636132	10.71996294722557
C	5.63693880893720	5.38202556712789	9.20444331285124
H	6.59968167048349	5.51469857384291	8.72904994972680
C	4.72407677436715	4.47446424492133	8.69434938730195
H	4.98238753861010	3.90067755048378	7.81431203415447
C	3.48859977097722	4.28449353357575	9.29771829722953
C	2.46287197429119	3.33519584570131	8.71766746428804
H	1.97308665288440	2.84703201309790	9.56328008556195
C	1.39972503306350	4.13133243667022	7.95182663126015
H	1.84529223739528	4.58745275948695	7.06552417352525
H	0.99102145394265	4.92971443226247	8.57115464266857
H	0.58301866746588	3.48289356000164	7.62588081428938
C	3.04783085116464	2.23828892296292	7.83390622161995
H	2.26454467331596	1.53345657482748	7.55140953365002
H	3.83516836131994	1.68886157119281	8.35136794247318
H	3.46534818331653	2.64248430494633	6.91031006156729
C	3.69393156287844	6.84250354609040	12.13881450127999

H	3.20347346863358	6.21342695117202	12.88455618773222
C	4.88159432842828	7.49988995283690	12.83634457346827
H	5.35244492014376	8.25169719146303	12.20074422752365
H	5.63785511582844	6.76560875179911	13.11552525671097
H	4.54353443618076	8.00562824422888	13.74135074421665
C	2.69310110691396	7.90985059887650	11.68051252367798
H	3.18780249425320	8.60469883006900	10.99861793654219
H	2.30506068262758	8.47463003128746	12.53030484249960
H	1.85069238098222	7.46242147640574	11.15409402482245
C	2.90184431264279	3.01847646441982	13.45135682247365
C	2.80310864712031	2.04447300474016	12.46603994733536
H	2.00972759243036	2.08108861241199	11.73305287130900
C	3.74435750335777	1.02439898490311	12.43394178008997
H	3.67484844040359	0.26959725070493	11.66139154819359
C	4.75902464381238	0.96531686563766	13.37645294464585
H	5.48600882441720	0.16553448591556	13.34309209417383
C	4.83542040605253	1.94003443890338	14.36213220614521
H	5.62179866951034	1.90243360862070	15.10413868582764
C	3.90987922097821	2.97001242098336	14.40238798196343

H	3.95279494473746	3.74557023073656	15.15520656877701
C	-0.52316371714755	6.60784241587508	12.71129751670516
C	-1.22940256137543	7.26547095108031	13.70984982855584
H	-0.86323288230251	7.21594010139586	14.72631600659170
C	-2.38284761557106	7.96148831414458	13.38439006599294
H	-2.93232915338678	8.47520135669521	14.16233933429316
C	-2.83643097856014	7.99676492077130	12.07224847906406
H	-3.73968356853889	8.53652730040596	11.82254349003144
C	-2.11870579833177	7.34135749702998	11.08303104413931
H	-2.45691861602844	7.37318237129042	10.05555650120387
C	-0.95405464985095	6.64812299279712	11.39044166339728
H	-0.37792195644912	6.15830316171624	10.61488967904265
C	-0.65986083215952	3.23826463243277	10.87496080235202
H	-0.80493434700970	2.15463687742023	10.87292090280006
H	0.03359033486953	3.50868078576266	10.08525657990401
H	-1.62470071117213	3.71763330734192	10.67825084012421
C	-1.03868292330939	3.48260574658070	13.28137517920515
H	-1.92345843455292	4.12100097347646	13.18390038837496
H	-0.53572047266605	3.72181492116903	14.21609687251865

H	-1.35986718078423	2.44017034357541	13.32221233350298
N	1.90440701892196	4.83424962121144	11.01713379232525
N	-0.13617575554208	3.66674447172652	12.15648218043915
O	2.00526445047719	4.06660212381628	13.57063480964885
O	0.61289002471164	5.93157763864692	13.10588018845831
P	1.17277744074885	4.63821762110420	12.32400896059461

Compound **8**:

C	1.36053991371109	6.62568255360153	13.06801110575154
C	1.37945998050644	5.95216810419890	14.31137158124937
C	1.23883180749700	4.56970606171817	14.34343433481617
H	1.23071505753038	4.05792479360626	15.29714228426177
C	1.12427944628546	3.82829013380086	13.17826610976627
H	1.00678094540112	2.75355137588656	13.22203537299614
C	1.20819909219310	4.47612716848264	11.95707106967959
H	1.17339901194241	3.89214879162409	11.04629746049073
C	1.34210819338197	5.85813428585719	11.87997495564935
C	1.56658284186236	6.55112186009339	10.55404002750994
H	1.03423857778919	7.49976863725308	10.58891312689636

C	1.06948591046424	5.77703190503950	9.33901954826032
H	1.66354897691282	4.87787574780856	9.16508156282083
H	1.15083673152916	6.39647108084863	8.44464423130279
H	0.02652910040329	5.47646839546745	9.45418753848831
C	3.05610621726352	6.88683118131739	10.41307789789187
H	3.39195406401520	7.48053715982945	11.26294006475312
H	3.24385821307777	7.44962585334967	9.49563048408569
H	3.64491832179563	5.96794807101684	10.37767199674120
C	1.67762848534918	6.73493289961298	15.57397279635136
H	1.13875824281926	7.67983935802922	15.51941624027796
C	1.25616948528918	6.04526647708186	16.86744727229073
H	0.20806247706875	5.74223991607067	16.83970892427124
H	1.39232820716989	6.72570088517995	17.71022387694208
H	1.86065866879554	5.15892419676739	17.06700528528590
C	3.17880984166220	7.05354458860766	15.60230355318223
H	3.75211725236714	6.13018897674920	15.70623752989357
H	3.42766480890713	7.70765940808106	16.44215118114314
H	3.48487311441536	7.53939656371813	14.67552353842363
C	-1.75614744899544	8.29120239475155	11.77988213853687

H	-0.93993327150612	7.93577274953772	11.14304070187698
H	-2.20634381190937	9.16267814599757	11.30074495839044
C	-2.76252033081600	7.15865641909680	12.00983183039067
H	-2.84202866059134	6.50218807798503	11.14523968182972
H	-3.75149914994777	7.57258437083375	12.21632514918749
C	-2.21757741907200	6.46353824633714	13.25757869377578
H	-1.34781021431108	5.85101045215713	13.00727309911514
H	-2.95315384784173	5.83748688537501	13.75998615584528
C	-1.78132780079778	7.65332170807976	14.10640380630381
H	-2.64115442528113	8.07892307598256	14.63496685369298
H	-1.02459414015511	7.39385116292042	14.84769268478676
C	1.18689151067226	10.16967747732197	10.82841449335460
H	0.59312249746366	9.52867926657534	10.16640644828246
H	2.18020999355611	9.73495275200415	10.92085479387975
C	1.17606327941722	11.60013194047753	10.29505519631995
H	2.01295926333981	12.16207131711155	10.71324442094013
H	1.24186936215316	11.63712337604635	9.20924602867242
C	-0.14585338109186	12.13952612943864	10.84419876512982
H	-0.22115937500284	13.22498656492923	10.82803191664942

H	-0.97602382952791	11.72960403133480	10.26464632955826
C	-0.17801334520815	11.56851688552942	12.26431888625605
H	-1.19671282834148	11.40049784341586	12.62328670720728
H	0.32230000694694	12.24140652238738	12.96875688700033
C	-0.69614861895842	10.46193033553828	15.45944742181911
H	-1.66695550562623	10.02607192228266	15.24184223034399
H	-0.73179813992246	11.52815863972423	15.19726440168128
C	-0.25194266012461	10.28960943164319	16.91772193206667
H	-0.64173361784049	11.07603807330436	17.56154548036996
H	-0.61815990751057	9.33377655547907	17.29306994219601
C	1.29349729560673	10.27928974790690	16.84893369452488
H	1.69842342284305	9.41477367231910	17.37351387633457
H	1.72879256763362	11.17034739455814	17.29788393282482
C	1.62445517268414	10.20250679384861	15.34351448234732
H	2.41539819191843	9.49626995607199	15.10186613338250
H	1.92099628096914	11.18890075800153	14.96430877572006
N	1.43608233105826	8.01735440927635	12.97696223861670
N	0.36742223668509	9.78417451202159	14.73660286820193
N	-1.26076413770660	8.63403858077227	13.13087928218319

N 0.56092895276404 10.30758947149297 12.14543519381891

P 0.34507751496898 9.08903151943357 13.23312053511235

Fluoride Ion Affinity Calculations:

The gas phase structures of the anionic fluoride ion adducts of **3**, **4**, **7**, and **8** and the structures of CF_2O and CF_3O^- were optimized at the M06-2X/def2-TZVP level of theory in the ORCA 4.0.0 software package. Structures were confirmed to represent minima on potential energy surface by the absence of any imaginary modes in frequency calculations. Zero-point energy and thermal corrections were applied to the electronic energy to obtain a corrected thermal energy. Reproduced here are Cartesian coordinates, electronic energies, and zero-point-energy- and thermally-corrected enthalpies. Fluoride ion affinities were computed as the reaction enthalpy of the following reaction:[11]

$CF_2O + [R_3P(F)=NR']^- \rightarrow CF_3O^- + R_3P=NR'$ $\Delta H_{rxn} = FIA$

Fluoride ion affinities were benchmarked to the experimentally measured FIA of CF_2O (50.0 kcal/mol).[0.02]

Compound	Electronic energy (Hartrees)	Corrected thermal energy (Hartrees)
**3**•F^-	-1638.06752	-1637.45757
**4**•F^-	-1669.71576	-1669.15762
**7**•F^-	-1711.87384	-1711.29550
**8**•F^-	-1599.439311	-1598.77389
CF_3O^-	-412.9846735	-412.96424
CF_2O	-313.0476679	-313.02968

Compound **3**•F^-:

C	2.27335525239917	6.10462051022193	7.33921069412564
C	1.54414843198910	7.17707987876629	7.91822326442389
C	-0.82181823926590	6.89486563745876	7.19841524149163
H	-0.87201301453381	7.89589317245615	6.75967037564187
H	-1.82989173895943	6.60240463037528	7.50316525451953
H	-0.47955325755686	6.20256724147312	6.43242999219337
C	0.13308635630259	6.91178947495809	8.39887498879444
H	0.13391790966815	5.91092648902586	8.83651590132646
C	-0.37106879304598	7.90764873374890	9.44091489621946
H	-0.51951442260926	8.89871563403757	9.00421449057368
H	0.32542584497574	8.00770222291732	10.27557243861875
H	-1.33626358171169	7.58241809260997	9.83417734326691
C	2.10810260762953	8.44353828196445	7.98337711060349
H	1.55192637814731	9.25232257879083	8.44353289359477
C	3.36930603631130	8.70099609747745	7.46706457603220
H	3.79601800001915	9.69449218975035	7.52667045340491
C	4.07118026277615	7.66637870327054	6.86534672031654

H	5.04944967581364	7.87192233851980	6.44844127270364
C	3.55356968719017	6.37899481242772	6.79123343313952
C	4.34081650618910	5.23975384871253	6.17379295306520
H	3.66654441561689	4.70338712359395	5.50062404501852
C	4.80480369094594	4.25722054405285	7.25630941671796
H	5.53924075473558	4.74478634915598	7.90329828793324
H	5.27254560809442	3.37794321382829	6.80691018979411
H	3.96312413142205	3.93108792137405	7.86368609106381
C	5.53192499428093	5.69286136190352	5.33471856995003
H	5.23738200233328	6.42302881527196	4.57895653656806
H	5.97079809840037	4.83176889434769	4.82710534403360
H	6.31040730508813	6.14292968948550	5.95599242556136
N	1.78079702909791	4.83181044771592	7.42112398884178
P	1.24023532839112	3.82307683787843	6.35776826018006
C	1.77296548309652	3.11506178338341	3.86602807561178
H	2.00568993716389	2.44652175370201	3.05417295311799
C	1.47062657987188	4.41445894243742	3.81199745342146
C	1.40655287228457	5.38858402661670	2.67429844805244
C	2.53994469707593	6.40760648582052	2.86676745719412

H	2.47939262390482	6.85600865777732	3.86016739702021
H	2.47608314718289	7.19883475437165	2.11504221474699
H	3.50946182355715	5.91423635576223	2.77490086343639
C	1.55686616865695	4.66853323838482	1.34031435616483
H	2.51779427120476	4.15451772428701	1.28055932988799
H	1.50243966069618	5.38552025155978	0.51830254244227
H	0.76118135180378	3.93267620568856	1.20748585767299
C	0.05764561504952	6.11922293479448	2.72956085147356
H	-0.76693204150549	5.41434486393129	2.60657806391932
H	0.00135840592511	6.86660658901658	1.93408306258315
H	-0.06248337044293	6.61718169570140	3.69081813038949
C	1.57971527106490	1.33942432969524	5.60305709133865
H	1.65184515685563	0.52695620962850	4.89938976137912
C	1.25975931812823	1.29529824979085	6.89788459886157
C	0.93507228177642	0.16031736367463	7.82192434097255
C	-0.44570354981012	0.42131907591640	8.44258027063579
H	-0.44561012163714	1.37744832846021	8.96415088938260
H	-0.69940766632631	-0.37094210348912	9.15160030884620
H	-1.21469250495933	0.46071393555698	7.66916359902224

C	0.93114983370445	-1.16605499342132	7.07155348201520
H	0.16380200762167	-1.16894954736700	6.29496086431490
H	0.72301660561675	-1.98451466457876	7.76409963890225
H	1.89943955611013	-1.35230334395578	6.60379192010779
C	1.98364436007314	0.12963781999712	8.94340379696823
H	2.97229034001077	-0.09118419894102	8.53797005765209
H	1.72885537221764	-0.63493595060035	9.68183436223718
H	2.02576132445162	1.09883998696557	9.43904414466517
N	1.80576786823365	2.66709911674984	5.19807074180049
O	1.24635495052381	2.51386627528423	7.47351132246334
O	1.26018227805759	4.95477168833356	5.02443476504718
F	-0.34651316737380	3.75124439149398	6.26765953653401

Compound **4**•F^-:

C	8.36178310803812	7.74120974331615	2.18504166540951
C	7.51380982174023	8.89318410981793	2.27775220986021
C	6.28975617292377	8.89557396436714	1.63086523838077
H	5.67530858350102	9.78839434980333	1.65398470423836
C	5.82467038366885	7.77870903616617	0.94909276354677

H	4.86254200187926	7.79956780710977	0.45293375929346
C	6.61029867799504	6.64195478137002	0.91513000662007
H	6.24497624974109	5.76967343896811	0.38310077649096
C	7.86666557067915	6.59583112025633	1.51322656504282
C	8.71704779950346	5.34498497629444	1.36505752537148
H	9.58394325952445	5.47518166591420	2.01322823353675
C	7.96675517079013	4.07601152424796	1.77602393348889
H	7.64077161854978	4.10854247434601	2.81667116586741
H	8.60968286986459	3.20454151426585	1.63026943869454
H	7.08095904308190	3.93349872002993	1.15513748079710
C	9.20258356020653	5.15861808450905	-0.07838708583808
H	8.36513373322813	4.87640180613427	-0.72320731337311
H	9.95176490163492	4.36647609456041	-0.14844924943646
H	9.64814176651743	6.07107674593647	-0.46685271244756
C	7.97086317606341	10.12071083005021	3.05437220039698
H	9.05265339241816	10.10431028545811	3.08390063182467
C	7.56549103298345	11.46283766304882	2.44092986589264
H	7.84880901461012	11.52755819871663	1.38868150541268
H	8.06871218429501	12.27168299478683	2.97364207293694

H	6.48952644735523	11.63949778979163	2.52667573457102
C	7.48720548606246	10.08241779410156	4.50946683345054
H	6.43042244770425	10.36289670829738	4.57217026695369
H	8.05339288769886	10.79387427601967	5.11434972286655
H	7.61124817838205	9.09037310559745	4.94193320517613
C	9.68831930721382	8.84553837824637	-0.20289001215614
H	10.09586332798849	9.39867865277901	-1.05620861505930
H	9.14926785048256	9.54783515774414	0.42708662451323
H	8.96128367498103	8.11614368159541	-0.57499068526580
C	11.83579945097476	7.75651779866681	-0.14840738827384
C	11.91368746766057	7.40970250737015	-1.49528585539287
H	11.04692413914729	7.51097441658214	-2.13513607149417
C	13.11151480597202	6.90608840174184	-2.00093190220030
H	13.17259770755663	6.63126803654760	-3.04700624183661
C	14.21101643781116	6.73475670552686	-1.18152373603491
H	15.13122127508150	6.33515275048177	-1.58650504637051
C	14.13790864500217	7.06831200142298	0.17704086792395
H	14.98594100949759	6.91128692494392	0.82963464111454
C	12.96498301092238	7.58811185767113	0.67823619413311

C	13.54860040884582	8.56687788081490	2.87105294324588
C	14.81932802718418	9.05527633373092	2.64138845880887
H	15.28124830099794	8.95939503713258	1.66955327572148
C	15.47757500789438	9.74836823009341	3.66529319752012
H	16.46503531005019	10.15205540783023	3.48125986521670
C	14.85873379717630	9.95479067443422	4.88397988322309
H	15.37053948540709	10.50571836722930	5.66316854189284
C	13.55237644306769	9.50867133346325	5.10425774100207
H	13.04260357846758	9.74053223979173	6.02933299948560
C	12.89206199122521	8.82046416525385	4.09601212736976
C	10.73014342265583	8.76235333496961	5.14369279485659
H	11.14342059619240	8.37740535557695	6.08075426026040
H	9.75900313568434	8.31292583308163	4.97245036805330
H	10.61684869521107	9.85236962984376	5.24543219708483
N	9.59614212071537	7.75765574638120	2.78753909761053
N	10.77244029358884	8.24444769757303	0.54116230365175
N	12.66081078065865	7.95678434976033	1.98374440767029
N	11.59203917491610	8.40218823994179	4.04705657014287
P	10.98217955770697	8.36327933534135	2.35475092274012

F 10.98402322142130 9.96858193315394 2.27186412581690

Compound **7**•F$^-$:

C 2.47096034876397 4.83429214362595 9.90527662563907

C 3.57291408086544 5.43594884370432 10.55838200175028

C 4.86590506906207 5.10212765536193 10.18571823456432

H 5.70365488171703 5.54834090218898 10.70880998557546

C 5.10845187143757 4.18368581978056 9.17418743031213

H 6.12239638055806 3.91872444044563 8.90049717343797

C 4.03240923672724 3.60938860255749 8.51007356175670

H 4.22579002119309 2.89953477067180 7.71506495809799

C 2.72229873377268 3.92047476533256 8.84854660322680

C 1.54082833681210 3.33368384866414 8.09999184183288

H 0.82232185865824 3.00304884700468 8.85430030215988

C 0.86165549428140 4.41468335940596 7.24939878078504

H 1.54350888905531 4.74367920107122 6.46030486028467

H 0.59407793856609 5.26983655050944 7.86781260211231

H -0.04163911966180 4.02093038165019 6.77415335470349

C 1.88337129325311 2.13216063976491 7.22461678265373

H	0.97071626407449	1.71848384055107	6.78969555759345
H	2.38058128907032	1.34343260075724	7.79179672968651
H	2.53603271523073	2.41850770820173	6.39608282309786
C	3.29284818412442	6.44932976042687	11.64610057872419
H	2.36581341449588	6.14679450486058	12.13702191809264
C	4.36419102997454	6.51915758891967	12.72680305384472
H	5.30262489301607	6.92855022108673	12.34433693063782
H	4.55881525215285	5.52969809054454	13.14320491192765
H	4.02525776386052	7.16951224706377	13.53594184352296
C	3.05757766308951	7.82238984863756	11.00830950744943
H	3.97613014932576	8.17218046994240	10.52993381135061
H	2.75573422352187	8.55890077645158	11.75759935569116
H	2.27984710234948	7.75438727091601	10.24555430287633
C	2.20608355570347	3.36136675800627	12.75874022935887
C	2.11458057429892	3.78958879863163	14.08634390385630
H	1.18605328515757	4.21351258906706	14.43808833071576
C	3.20675370119752	3.64796042214609	14.93353848068533
H	3.11953430850441	3.97631812291356	15.96233188641007
C	4.39485598248701	3.08983711392837	14.48554760796895

H	5.23939310121425	2.98649341918528	15.15437110989317
C	4.48725618139824	2.67227690211765	13.16078427378727
H	5.41033101703698	2.24809959408229	12.78432377891261
C	3.41027278496495	2.80701940325401	12.30570820145290
H	3.47304019720387	2.50931209314659	11.26566583341602
C	-0.62022090539756	6.37516869829411	13.05015842703849
C	-0.16728721096984	7.54526454531741	12.44535843043998
H	0.54713987769197	7.46939815304193	11.63735640588604
C	-0.67910708100066	8.76392580943373	12.85635688201377
H	-0.32667679500154	9.67059707375445	12.37991552459517
C	-1.64388647584796	8.83352367157989	13.85657798590412
H	-2.04557966195027	9.78983772266583	14.16599043867597
C	-2.09699194150283	7.66170947285996	14.44373892814558
H	-2.85903639409948	7.69756200987294	15.21272922036031
C	-1.58830788847587	6.43393635993913	14.04431440028853
H	-1.93820320823476	5.50701407633459	14.47990153858910
C	-1.71132789945386	3.35322977116197	9.61051928927473
H	-1.43753967719506	2.50384243996833	8.96971798654592
H	-1.51636385881561	4.26515527304916	9.05644091745467

H	-2.78759894724601	3.28856701294815	9.80824872220134
C	-0.93066548396925	1.99000612544429	11.42976705166163
H	-1.94076740017178	1.57363692694213	11.35551979358615
H	-0.65157932687478	2.02771508453357	12.47784016362688
H	-0.24296974552882	1.30591587087751	10.91499941621056
N	1.19381412571896	5.20430303504378	10.22732152149784
N	-0.94764856092216	3.31058034175036	10.84352607713745
O	1.16582564148148	3.34778389881094	11.91519850606674
O	-0.10681039430239	5.15411698554258	12.74273760805802
P	0.13497915888712	4.56459780796888	11.20064602607346
F	-1.01911989533425	5.62036091628604	10.67955867882223

Compound **8**•F^-:

C	1.22910968108767	7.02518327393299	13.20263270889412
C	2.08358436942563	6.34469582777949	14.14803119640225
C	2.93999100842376	5.33522542884455	13.76118043071010
H	3.59504674090417	4.88668705368550	14.50170617894663
C	2.99014480579824	4.88180509428848	12.44768554522807
H	3.68217718621417	4.10673450644034	12.14543606711366

C	2.08544652572466	5.42416601738660	11.55393410748905
H	2.06467758138369	5.03824098941590	10.53899228350600
C	1.18502175453813	6.43201917523662	11.88847154473429
C	0.17706691187317	6.80580897043869	10.80599647866953
H	-0.51872147920447	7.53759405091326	11.20261896604506
C	-0.65124497262027	5.58364155645788	10.37798060564638
H	-0.03436893721147	4.85738678338074	9.84403185764688
H	-1.46277670625252	5.88488613916982	9.70955297804310
H	-1.08481223678969	5.07765604866905	11.24124043695695
C	0.82762995563198	7.42737550992332	9.56773886557820
H	1.36037144260341	8.33507894494937	9.83600874625934
H	0.06873087072926	7.68541078648310	8.82352443232949
H	1.53100195117735	6.73165183888572	9.10357157019378
C	2.06190270351212	6.80743541287110	15.58860328595775
H	1.12867617491792	7.35521610181947	15.70775979815858
C	2.08022728767458	5.65590528679268	16.59383364373223
H	1.28835985972389	4.93810781326025	16.37582939075635
H	1.93390220433875	6.03262532863394	17.60928340023221
H	3.03147555311505	5.11890367378318	16.58013707577501

C	3.21210764365544	7.77809549118461	15.86478485050714
H	4.16986417296317	7.25270283355880	15.81074883182903
H	3.12410995432603	8.23319849206382	16.85591149660416
H	3.22492178891616	8.57273081558800	15.11899049039600
C	-3.04070409087465	8.93208343333942	12.30717309247598
H	-2.99795559635506	8.94369837876202	11.21022604878670
H	-3.28616675078352	9.94262891789289	12.62271678795847
C	-4.07632824409218	7.91965397817395	12.78816227636230
H	-4.95448012266129	7.87733522497674	12.14216385051483
H	-4.40493666609963	8.18179018176237	13.79755442183109
C	-3.27051122064112	6.62795958503397	12.82794857286505
H	-3.13296052320571	6.25183830627252	11.81024264746844
H	-3.72386604257642	5.83620720799716	13.42474759498895
C	-1.92649056131439	7.09199956795146	13.38836903451168
H	-1.93358616919107	7.07878057239287	14.48436820594698
H	-1.11978166853294	6.43704365910300	13.07277806735919
C	2.33799233008331	9.52794756243645	12.07937458779897
H	2.68054738042828	8.58052772471854	11.65043688160424
H	2.53941657894197	9.48465160806789	13.14764198743229

C	3.05395652738746	10.70099856360090	11.40996709941648
H	3.06577688445427	11.56335733084528	12.08182808163241
H	4.08240531588285	10.46122341447017	11.13912366350037
C	2.15225582643245	11.00197746308542	10.21888855775209
H	2.30393667394355	11.99377394647873	9.79000144542054
H	2.31338547652798	10.26360270313677	9.43072369240566
C	0.76454820397478	10.84702960137361	10.83526255152383
H	0.00159949812023	10.61191287179644	10.09195643149129
H	0.47141049290025	11.78462285156931	11.31766048158917
C	-1.41267939139656	11.23341409921884	14.64945974057225
H	-2.35658271145648	10.72567847463645	14.43698319863832
H	-1.39655978223696	12.14838799736645	14.05171743496286
C	-1.27907378387460	11.54160598046537	16.14591615331268
H	-1.66322130993907	12.52869836253761	16.40512730588771
H	-1.83051971632079	10.80111755876683	16.73012202322321
C	0.21638926976973	11.36616559762589	16.39933012664304
H	0.46821329576432	11.22530149875296	17.45079772014444
H	0.76617982637756	12.23184216729711	16.02203278647630
C	0.52469376272323	10.14183315005341	15.54136805121990

H	0.21853583974281	9.22905468654855	16.06619344610804
H	1.58151784062499	10.04077296035539	15.30718997946093
N	0.57067324059017	8.10038651004833	13.66364390407308
N	-0.25987508290038	10.38137936131641	14.33839918193485
N	-1.77123151103513	8.46095546515038	12.88129379138659
N	0.90701680357732	9.75812448360520	11.81593009083296
P	-0.28328505304989	9.32932938713714	12.99656551233849
F	-1.19737886628960	10.54213835804243	12.21549622580595

CF_3O^-:

F	0.93083875100715	0.75206943760599	0.49568133284004
C	-0.17929666931632	-0.10934456511806	0.45463639593927
O	-0.56719288958903	-0.56401931964403	1.51115291230858
F	-1.08878086355194	0.66830814669814	-0.28202605957205
F	0.23809167145013	-1.03235369954203	-0.52104458151583

CF_2O:

F	1.02053735747867	0.05184805214319	0.27277202022499
C	-0.23765107788953	-0.02128160512980	0.60607653671309

O -0.65748003022811 -0.06149231004403 1.69028967787889

F -0.93418624936102 -0.04412413696937 -0.49581823481698

Proton affinity calculations:

The gas phase structures of the cationic N-protonated adducts of **3**, **4**, **7**, and **8** were optimized at the M06-2X/def2-TZVP level of theory in the ORCA 4.0.0 software package. Structures were confirmed to represent minima on potential energy surface by the absence of any imaginary modes in frequency calculations. Zero-point energy and thermal corrections were applied to the electronic energy to obtain a corrected thermal energy. Reproduced here are Cartesian coordinates, electronic energies, and zero-point-energy- and thermally-corrected enthalpies. Proton affinities were calculated as the enthalpy of the following reaction:

$[R_3P=N(H)R']^+ \rightarrow H^+ + R_3P=NR'$ $\Delta H_{rxn} = PA$

Compound	Electronic energy (Hartrees)	Corrected thermal energy (Hartrees)
**3**•H$^+$	-1538.49008	-1537.86796
**4**•H$^+$	-1570.17090	-1569.60173
**7**•H$^+$	-1612.33442	-1611.74378
**8**•H$^+$	-1499.973864	-1499.29595
H$^+$ (5/2*R*T)	--	-0.002360459

Compound **3**•H$^+$:

C 3.04921734162251 6.24450502556918 7.27462976700267

C 2.12488285144781 7.19835370972058 7.70227176587878

C	-0.33470745329747	7.42254944377565	7.30536070515630
H	-0.30579016681090	8.51308762929690	7.29608506010928
H	-1.32333151133674	7.12035508034689	7.65229035521684
H	-0.20613421742269	7.07722518311399	6.27828711270512
C	0.74817312260099	6.85597102025118	8.22622116515645
H	0.63617070292473	5.77068004970674	8.24339824611437
C	0.55960627680094	7.35910283937613	9.65758278878184
H	0.60713957602508	8.44818246457961	9.69560472542616
H	1.32665478616210	6.96603560898668	10.32507005451297
H	-0.41632305371159	7.05669660953769	10.03800103181276
C	2.49684489872283	8.53810516543560	7.60209142969503
H	1.80687270691802	9.30448617343032	7.93071183433000
C	3.72282050421975	8.90150292656318	7.07879813952107
H	3.98966529680432	9.94736933100784	7.00681333276142
C	4.60720625622128	7.93099949209834	6.63086202840345
H	5.55291156375410	8.23285405453386	6.20426878053045
C	4.28811269043562	6.58140992042844	6.71617313808468
C	5.25308344231543	5.51136809502066	6.25639425246501
H	4.67847962635692	4.71053669864376	5.78253841198000

C	5.98235444247310	4.91142164482792	7.46011074310342
H	6.57006992450356	5.68308037953757	7.95928381400293
H	6.65947583179104	4.11882685800501	7.14119838694999
H	5.29143823864433	4.49478358887822	8.19380263793605
C	6.25721248535289	6.00031576632524	5.22059386147362
H	5.76596767803133	6.49848597575185	4.38334405412158
H	6.82045446804734	5.15375728319976	4.82847521472915
H	6.97700522149645	6.69367426131759	5.65706993278136
C	2.77278329089608	3.84948383785529	3.81396381623141
H	3.32732472994482	3.48463734961748	2.96647799855532
C	2.09948207571749	4.99423541610927	3.89460202381040
C	1.88878889005726	6.14068134802102	2.96981593640574
C	2.50181927466338	7.39764630485577	3.60038164915680
H	2.07298393374214	7.60391216897627	4.58338991639882
H	2.30653581782848	8.25473475003857	2.95519127872557
H	3.58131567621306	7.29200601529195	3.71444113435937
C	2.55365120254780	5.83833628847422	1.63098738892364
H	3.63075698429922	5.70267272179334	1.73998723500073
H	2.38960306966491	6.67594554504291	0.95354378897664

H	2.12751725009138	4.94683147202198	1.16755179122011
C	0.38183281710009	6.34769236607942	2.77158003102258
H	-0.08772827874163	5.45915644002310	2.34716679540180
H	0.22436749681292	7.17729252438979	2.08225764468150
H	-0.11477143396238	6.59260833420849	3.71047972159962
C	1.93120901898744	1.87945690649162	5.01734817617799
H	2.15103598009267	1.10691253139432	4.30048504426967
C	0.96463439030813	1.86647763601527	5.92806075614126
C	-0.12993432589351	0.91523290080136	6.25986864428848
C	-0.06165511565557	-0.27175072057470	5.30430380336073
H	-0.18693772372832	0.04657351585676	4.26812293428782
H	-0.86702606153952	-0.96689642719908	5.53984556241785
H	0.88237300418539	-0.81008710408931	5.39894541155089
C	0.01235325547782	0.44978680842433	7.71249728092867
H	0.94948471693594	-0.08662989153658	7.86424524405075
H	-0.81043332123056	-0.22161209243185	7.95919416703774
H	-0.02504314475812	1.29359681998892	8.40209414177867
C	-1.46821389906696	1.64675125314731	6.08372978861705
H	-1.55418977744365	2.48969473261189	6.76959158551678

H	-2.28413675825048	0.95554382985435	6.29661743627739
H	-1.58809566387968	2.01103390149623	5.06233098864742
N	2.71899046199917	3.07887088052309	5.02641868435440
N	2.68619311823527	4.83908050571544	7.35994301215485
O	0.97948325694168	3.04361302461317	6.72560431017697
O	1.49331781004252	5.17454491550677	5.16441340123193
P	1.99983680474620	4.07216416141793	6.14986556340000
H	2.84225764552603	4.34504674990750	8.22992314211988

Compound **4**•H^+:

C	7.98639112129728	7.92081097880175	2.43823287852347
C	6.94430962026309	8.76217605992521	2.89202980733756
C	5.64597241225335	8.46072416490630	2.50761695438054
H	4.83117639468746	9.08166991115450	2.85146657625103
C	5.37630530904732	7.37652017866648	1.68353443742521
H	4.36284765178016	7.15809535765173	1.37214414979423
C	6.40436070844911	6.54475210543452	1.29777447666662
H	6.18021554082927	5.66640490901404	0.70809255616159
C	7.72542879747468	6.76795055244263	1.69722148299157

C	8.74087546161474	5.67330673770955	1.43369222254442
H	9.70325529076677	5.96696687847136	1.85808997333073
C	8.28681889769132	4.39643102324797	2.16022163832900
H	8.03824842575929	4.58807787339825	3.20415193411173
H	9.06473326661801	3.63621305405931	2.10953074024397
H	7.39727505692169	3.98795556222017	1.68004161180909
C	8.91802719060860	5.37869194905185	-0.05495109751643
H	8.00023758467908	4.96066125188512	-0.47004882904457
H	9.71301451208656	4.65060152468737	-0.21641115728835
H	9.16496024980189	6.26944030238506	-0.62705683721842
C	7.21620202964006	9.89463781814558	3.86895986387306
H	8.10582966742549	10.43619150246449	3.53140224355322
C	6.08499667917553	10.91492689954774	3.96283960491800
H	5.78855352068745	11.30065668277274	2.98731012852345
H	6.40174710681386	11.75374268757535	4.58254388692968
H	5.20731775999769	10.47667354727194	4.44059751522921
C	7.48167734897627	9.31328331567056	5.27298488647016
H	6.55837877582730	8.88156161225187	5.66340802236444
H	7.81958378562455	10.09075055253084	5.95857783597986

H	8.21503264803506	8.50462902504715	5.29037735712193
C	9.46413013671602	9.04984540442973	-0.27698522578887
H	9.93574605908175	9.76100381388927	-0.95649640356866
H	8.81482457573548	9.58708712570812	0.41447469810131
H	8.84341828598414	8.35508606942087	-0.84178483269300
C	11.49208616625724	7.56088825996147	-0.16540497628888
C	11.64079983496903	7.35498096303481	-1.52214328292735
H	10.94491261487029	7.78571530886220	-2.22986793747070
C	12.72489564928822	6.58878222431620	-1.95244917233457
H	12.87631540094437	6.41784021062689	-3.00956870990917
C	13.61640226238123	6.05126222793908	-1.03910577206524
H	14.45419210861742	5.46871405123927	-1.39251270400400
C	13.45605784955094	6.25285554490019	0.33081042568273
H	14.15943688224753	5.83740838319360	1.03493988852154
C	12.38505611229012	7.00749412735887	0.75961356970339
C	13.07573391552380	7.92898399908939	2.98529034403813
C	14.21428545562083	7.30697178527624	3.45214001716561
H	14.43638272717380	6.28168120925206	3.19575895945944
C	15.07262398311918	8.03726267649216	4.27507354356991

H	15.97080840250202	7.56788984985826	4.64969326569326
C	14.78466617296001	9.34632132917412	4.62384986967473
H	15.46677223890201	9.89892938356269	5.25446443422128
C	13.61942411970844	9.96855428908623	4.18062143951175
H	13.38719548210300	10.98466597982797	4.46987843870932
C	12.77248912661650	9.23979939740603	3.36932871175381
C	11.00062961289018	10.99543936499645	3.14226915771793
H	10.88010760799199	11.16577848535809	4.21400589961345
H	10.03544219211588	11.11756508761621	2.65437116747278
H	11.68356157208980	11.74238850197684	2.73875166470490
N	9.33081905539949	8.29499938167495	2.83361843712541
N	10.48117565454690	8.32711476651123	0.46947400788724
N	12.07224904373940	7.40267521487594	2.10613295638323
N	11.51557052996029	9.65901148895783	2.87410260981851
P	10.75461564037377	8.43779189148353	2.06980593464671
H	9.37050071289601	8.67500818425189	3.77157471207809

Compound **7**•H^+:

C	2.72542128291611	4.82417623716335	10.31981806998698

C	3.64667162569345	5.49621698489395	11.13243461726081
C	4.98113959531593	5.49476347587585	10.72919551419441
H	5.71781647780284	5.99731742195517	11.34098453814336
C	5.37568779543627	4.86985176976897	9.56234043266595
H	6.41385840543363	4.89449674792875	9.25702803820017
C	4.44264860262778	4.23038802065587	8.76420785710638
H	4.76670292562125	3.75986228064633	7.84635962098297
C	3.09838208979689	4.19404066196729	9.11958041121313
C	2.07534749203889	3.58448206483365	8.17389491580409
H	1.25754203809172	3.18027185910061	8.77254609747425
C	1.49956219281211	4.67296081295058	7.25901607694422
H	2.30358848920228	5.15463897059342	6.70034932060355
H	0.97443522559377	5.45212609151559	7.81622033430727
H	0.80007393838173	4.23738814995019	6.54463274949702
C	2.63042275479911	2.43702492995772	7.33204524151810
H	1.81363825300038	1.93556564407384	6.81188361567155
H	3.15501132942973	1.70438851551769	7.94586478360750
H	3.31883353089144	2.79913316159063	6.56834239839145
C	3.26102210277730	6.28016962660936	12.37583356629906

H	2.34092479086851	5.87668711094707	12.79666927078526
C	4.29837114717180	6.21947640413179	13.49601422900240
H	5.15726687893365	6.85113979586922	13.26800186337125
H	4.66020705966505	5.20970878442313	13.68505645278851
H	3.84706200850296	6.59837258301910	14.41465538106704
C	3.01643829539858	7.74387245492183	11.99359044613053
H	3.90777637772359	8.14406015107970	11.50669416137530
H	2.82074283703704	8.34350263225022	12.88310105168909
H	2.17542368590793	7.85471117678089	11.30794452559624
C	2.81453950780245	3.05755655259913	13.30169018574530
C	3.95801453335001	2.67497999215965	12.62549035194490
H	3.92685061207080	2.49867069652609	11.55736179116524
C	5.12844112214996	2.51301814546937	13.35279088857027
H	6.02880509688708	2.20672637448271	12.83707728063211
C	5.13879819702767	2.72977331178782	14.72614658201589
H	6.05094735476963	2.58508866900364	15.28854235892721
C	3.97564341891392	3.11616664363470	15.37764016419986
H	3.98170464314520	3.28675321445193	16.44555155234887
C	2.79286137037945	3.28392139871766	14.66556061295324

H	1.87060792984130	3.56462572213884	15.15745907057187
C	-0.37895367486757	6.38214392790481	12.68992392881930
C	0.12601500254147	7.48141490494098	13.35788555874819
H	0.88234650850907	7.34404983918855	14.11962716823670
C	-0.33671948258793	8.74246098880943	13.00782740156311
H	0.06544127734276	9.61062258528582	13.51226118188708
C	-1.29149715617934	8.88989300349310	12.00974964200478
H	-1.64404796626683	9.87336743600246	11.72986601794448
C	-1.80360613379949	7.77028248736185	11.36945111058984
H	-2.55920645846536	7.87423137400497	10.60327617376143
C	-1.34823207825966	6.50372409745469	11.70555815847965
H	-1.74642344044454	5.63462252409221	11.20073725123156
C	-0.53352723270501	2.08383794517232	10.64797151019621
H	-0.82696095741776	1.20030758989410	11.21618483782612
H	0.50272525542608	1.95976750493186	10.33918561115246
H	-1.16410093440247	2.16495868835892	9.76283829980927
C	-1.95083072340017	3.23709522879135	12.25762868928313
H	-2.77534671790162	3.14698417948942	11.54897987179038
H	-2.08240199413733	4.13974556449230	12.84335679985808

H	-1.96863048149439	2.37309214784129	12.92433685326921
N	1.31906237061109	4.79248360478424	10.67472903521726
N	-0.70049548546998	3.27251645036842	11.49567736099787
O	1.60833743677236	3.09867644165810	12.58080321993932
O	0.13200598639295	5.11384007901065	13.03784606156825
P	0.61427570907535	4.09011877092143	11.94732623249072
H	0.68893835591906	5.21308739380337	10.00315560258307

Compound **8**•H^+:

C	1.66152558934346	6.57306076406756	13.16969561160603
C	1.80831149813719	6.00666077683689	14.44421356900211
C	1.65112157018156	4.62891601593659	14.56611523606868
H	1.75961698033515	4.16189995559702	15.53502171890568
C	1.34168783237658	3.84703881307425	13.46545888085433
H	1.21362736251233	2.77902995911278	13.58183865201712
C	1.19666637097730	4.42677341109589	12.21587358600440
H	0.95723377873524	3.80264435258392	11.36598372889791
C	1.35938629422246	5.79757023904458	12.04143964893180
C	1.26746755378934	6.42787667445871	10.66674385139035

H	0.85894857199420	7.42801057658418	10.79777449043340
C	0.34858622499039	5.69070854653307	9.69832371260777
H	0.75518594520005	4.72153258643872	9.40858859277866
H	0.23394515580883	6.27614115990434	8.78539546219373
H	-0.64117033612138	5.52814083352667	10.12847440212153
C	2.67025469231483	6.58141179929445	10.06962466073439
H	3.32325946853496	7.15833847193801	10.72641773753010
H	2.62531895326574	7.08193727844580	9.10114858813692
H	3.12729710628194	5.60155261632323	9.92499998078284
C	2.15244515488074	6.86799145940202	15.64567311739454
H	1.58357762414069	7.79271365733019	15.54634648191684
C	1.76113170306638	6.25265574067661	16.98631233936754
H	0.71690297572873	5.93828176190209	16.99647495095595
H	1.90366594634299	6.98650877817306	17.78024643304936
H	2.38100409082126	5.39001305389249	17.23213006741538
C	3.64469809568716	7.21899598634014	15.64552561020762
H	4.24437251240356	6.31675390776981	15.77175001925204
H	3.88020345057076	7.90086166282925	16.46468835598887
H	3.96084151614168	7.68895197247697	14.71258984657378

C	-1.74119255661788	8.27855678578491	11.67722194725349
H	-1.19708172052988	8.01003608905516	10.77102504748796
H	-2.09774288407100	9.30567886938282	11.57472073746915
C	-2.89795393080616	7.30357039392409	11.93915679504140
H	-2.67776789986074	6.33974161377130	11.47920869238210
H	-3.83429955693280	7.66800541513205	11.52374443502828
C	-2.92501830552439	7.15289458268410	13.46073856859613
H	-3.43334361511596	6.24989834059462	13.79041808909267
H	-3.41558354982466	8.01318269672935	13.92027935298043
C	-1.44143439950770	7.14409759161225	13.81073817683415
H	-1.22490803325585	7.41425447572982	14.84512042122087
H	-0.99868334371511	6.16415366542750	13.61421673590021
C	1.21657629677621	10.02273388308301	10.64631551303143
H	0.54150508705169	9.43479470829904	10.01695906517783
H	2.18667944911795	9.52938572367249	10.66486509630387
C	1.26400328092097	11.46358451352575	10.14977585408483
H	2.14491346292751	11.96855769018418	10.54822209258056
H	1.29329579723385	11.52099724142847	9.06436625044976
C	-0.00936084013320	12.05578780685068	10.75377377598697

H	-0.02116005356891	13.14279184628539	10.77217328376001
H	-0.87837889877089	11.71564858644396	10.18773447827546
C	-0.03896637711407	11.45821877737702	12.16253130770552
H	-1.05838250189309	11.31157582425373	12.52468236661167
H	0.48946210133756	12.09271307572222	12.87713700768581
C	-0.85223636053809	10.16301886279596	15.26036842125362
H	-1.71407821095926	9.54648866756757	15.01506987466391
H	-1.06631152235005	11.19456732404330	14.95914632895368
C	-0.44087260220160	10.10775103077191	16.73160893109092
H	-1.03805392203380	10.77367709549262	17.35004619916433
H	-0.57136757766860	9.09080312929927	17.10206072044768
C	1.05574894344967	10.48333400013415	16.71776589222933
H	1.63122108158969	9.83340865749439	17.37369132264200
H	1.21461764559986	11.50719037230616	17.04926059172460
C	1.50237032286061	10.32413451348481	15.24649822200720
H	2.40132788635354	9.71765528783988	15.13832637049676
H	1.69325360586127	11.30101441204805	14.79255613405103
N	1.76530384601528	8.00819414368078	13.00162121878209
N	0.35776575032726	9.67756030536946	14.59260299371358

N	-0.89259148665216	8.15292731632287	12.88371545345406
N	0.67771866750689	10.16856203316872	12.01280931088113
P	0.46870999428743	8.99718855782867	13.11738516168883
H	2.69068324776447	8.41539128180817	13.00427242869494

References

[1] A. B. Pangborn, M. A. Giardello, R. H. Grubbs, R. K. Rosen and F. J. Timmers, *Organometallics*, 1996, **15**, 1518–1520.
[2] P. J. Alaimo, D. W. Peters, J. Arnold and R. G. Bergman, *J. Chem. Educ.*, 2001, **78**, 64.
[3] Bruker, *SAINT*, 2012, Bruker AXS Inc., Madison, Wisconsin, USA.
[4] Bruker, *SADABS*, 2001, Bruker AXS Inc., Madison, Wisconsin, USA.
[5] G. M. Sheldrick, *Acta. Cryst.*, 2008, **A64**, 112-122.
[6] L. J. Farrugia, *J. Appl. Cryst.*, 2012, **45**, 849-854.
[7] S. M. McCarthy, Y.-C. Lin, D. Devarajan, J. W. Chang, H. P. Yennawar, R. M. Rioux, D. H. Ess and A. T. Radosevich, *J. Am. Chem. Soc.*, 2014, **136**, 4640–4650.
[8] W. Zhao, S. M. McCarthy, T. Y. Lai, H. P. Yennawar and A. T. Radosevich, *J. Am. Chem. Soc.*, 2014, **136**, 17634–17644.
[9] L. A. Hussain, A. J. Elias and M. N. S. Rao. *Tet. Lett.*, 1988, **29**, 5983-5986.
[10] D. L. Herring, *J. Org. Chem.*, 1961, **26**, 3998-3999.
[11] K. O. Christe, D. A. Dixon, D. McLemore, W. W. Wilson, J. A. Sheehy and J. A. Boatz, *J. Fluorine Chem.*, 2000, **101**, 151–153.
